# Residents Are Coming: A Faculty Development Curriculum to Prepare a Community Site For New Learners

**DOI:** 10.21980/J87D2N

**Published:** 2022-07-15

**Authors:** Keith Willner, Essie Reed-Schrader, Stephen Mohney

**Affiliations:** * Geisinger Wyoming Valley Medical Center, Department of Emergency Medicine, Wilkes-Barre, PA

## Abstract

**Audience:**

This curriculum is designed for emergency medicine attendings in varying years of community practice to prepare them for Emergency Medicine (EM) residents

**Length of Curriculum:**

15 months

**Introduction:**

Emergency medicine is a growing field with new residencies approved every year. A strong, competent cadre of clinical educators is essential to the success of any residency, and new programs have the challenge of developing their clinical faculty into outstanding teachers. There is minimal literature guidance for navigating this transition. Our site is a community tertiary care center in the process of applying for an EM residency. We focus on our experience designing a faculty development curriculum to accommodate the needs of a diverse group of physicians in all stages of their careers. We will demonstrate that a curriculum satisfying all stakeholders can easily be implemented in a way that allows for robust participation without excessive additional administrative burden.

**Educational Goals:**

Our goal is to prepare community-based EM attendings to be outstanding educators to future residents by augmenting their knowledge of current educational practice and adult learning theory, literature review, and biostatistics.

**Educational Methods:**

The educational strategies used in this curriculum included lectures, guided discussion, small group discussion, and asynchronous learning.

**Research Methods:**

This curriculum was implemented in the Geisinger Wyoming Valley Medical center targeted at staff physicians. This educational study was deemed exempt by the institutional review board (IRB). We electronically collected retrospective survey data using a 5-point Likert scale as well as free text responses. The primary measure was agreement with the statement, “Faculty development time makes me feel more prepared to be a clinical educator.” We also surveyed whether this was felt to be an appropriate use of time, self-reported growth in key educational and biostatistical domains, and likeliness to change practice based on the material.

**Results:**

Responses collected from core faculty after the sessions indicated a uniformly positive review of the series itself with the primary outcome receiving a 4.6 rating on a 5-point Likert scale (strong agreement). Faculty reported that these brief sessions improved the quality of the departmental staff meetings (average rating 4.7/5). Journal club sessions were rated as positive (4.7/5) and attendees self-reported growth in statistical literacy and security in clinical practice.

**Discussion:**

We demonstrated successful implementation of a faculty development curriculum that was favorably assessed by all key stakeholders. Faculty self-reported growth in all educational and clinical domains evaluated. It was successfully implemented without substantially increasing the time burden for physicians with robust clinical and administrative schedules. We feel this is generalizable to other sites seeking to start an EM residency and is useful for sites with existing residencies to efficiently deliver content to junior faculty.

**Topics:**

Emergency medicine, faculty development, journal club, virtual learning.

## User Guide

**Table:** 

**List of Resources:**	
Abstract	1
User Guide	3
Appendix 1: Curriculum Timeline	7
Appendix 2: Journal Club Discussion Questions and Answer Key	11
Appendix 3: Assessment PowerPoint	31
Appendix 4: Feedback PowerPoint	32
Appendix 5: Stroke Alert Update PowerPoint	33
Appendix 6: Bedside Clinical Teaching PowerPoint	34
Appendix 7: COVID Updates PowerPoint	35
Appendix 8: Push-dose Vasopressors PowerPoint	36
Appendix 9: Loop Drainage of Abscess and Bedside Procedure Teaching PowerPoint	37
Appendix 10: Atrial Fibrillation and “When You and Your Trainee Disagree” PowerPoint	38
Appendix 11: How to Give an Amazing “Chalk Talk” PowerPoint	39
Appendix 12: Bedside Teaching for the New Attending PowerPoint	40
Appendix 13: Status Epilepticus PowerPoint	41

**Learner Audience:**

Attending Faculty

**Length of Curriculum:**

15 months

**Topics:**

Emergency medicine, faculty development, journal club, virtual learning.

**Objectives:**

**Goal 1:** EM faculty will become proficient in interpreting the primary literature as it pertains to the daily practice of EM before the arrival of residents and will be able to decide whether and how to modify their practice based on review of selected studies in the primary literature.

Learning Objectives for Goal 1:

1. EM faculty will be able to discuss key basic biostatistical concepts from 2 pre-selected studies every other month
2. EM faculty will be able to cite sources of bias, both external and internal, in studies demonstrated in the primary literature.
3. EM faculty will be able to explain how a patient population included in a clinical trial is either similar to or different from the patients they serve.
4. EM faculty will describe how methodological issues or flaws in study’s design and reporting will influence their decision to act on its results in daily practice.

**Goal 2:** EM faculty will apply modern educational and adult learning theory to their clinical and bedside teaching.

Learning objectives for Goal 2:

1. EM faculty will be able to deliver excellent clinical teaching given the constraints of a busy emergency department
2. EM faculty will provide timely and effective feedback to their learners and produce robust and useful assessments on their progress

### Brief introduction

Emergency medicine (EM) is a growing field with new residencies approved every year. A strong, competent cadre of clinical educators is essential to the success of any residency, and new programs have the challenge of developing their clinical faculty into outstanding teachers. Furthermore, aspiring faculty often have multiple other demands on their time making a concise, integrable delivery format imperative. The COVID-19 pandemic has increased the challenge with institutional limitations on in-person meetings and the physical and emotional demands of working in an overstretched healthcare system.

There is minimal literature guidance for navigating this transition. It is recommended that prospective programs implement a faculty development curriculum approximately 18 months prior to the arrival of new learners.^1^ How that is implemented is institution dependent. Journal clubs are widely used to teach biostatistical literacy and promote critical appraisal of the medical literature.^2^,^3^ Medical podcasts are a popular way for clinicians in all stages of their training and career to stay informed about current literature, access practice changing updates, and stay engaged with their specialty community.^4^ These are especially appealing as a curricular supplement because they can be used while engaged in other tasks.

Our site is a community tertiary care center in the process of applying for an EM residency. We focus on our experience designing a faculty development curriculum to accommodate the needs of a diverse group of physicians in all stages of their careers. We will demonstrate that a curriculum satisfying all stakeholders can easily be implemented in a way that allows for robust participation without excessive additional administrative burden.

### Problem identification, general and targeted needs assessment

We reviewed the EM-specific Accreditation Council for Graduate Medical Education (ACGME) requirements with particular attention to faculty requirements to initiate a general needs assessment. The ACGME requires all program faculty devote time to practice-based learning and improvement. They also require a core faculty with training in teaching, evaluation, and feedback.^5^ Formal targeted needs assessment determined particular knowledge gaps in educational theory as well as biostatistical literacy. To this end we utilized two separate instructional methods. We implemented a bimonthly journal club to improve biostatistical knowledge and increase faculty review of the primary EM literature. We supplemented articles with podcast FOAMed resources intended to increase compliance with article review and to prepare faculty to help future residents determine how to incorporate these resources into their own practice. We utilized a brief lecture format on some aspect of educational theory or clinical medicine delivered at monthly staff meetings to introduce topics related to bedside teaching, assessment, and feedback. Participation in journal club sessions was voluntary. Staff meeting attendance is required, though meetings were conducted virtually during the pandemic.

### Goals of the curriculum

This curriculum seeks to prepare attending emergency physicians in community practice for the arrival of resident learners and augment their teaching of medical students by improving their knowledge of current educational practice and adult learning theory, literature review, and biostatistical literacy.

### Objectives of the curriculum

#### Goal 1

EM faculty will become proficient in interpreting the primary literature as it pertains to the daily practice of EM before the arrival of residents and will be able to decide whether and how to modify their practice based on review of selected studies in the primary literature.

Learning Objectives for Goal 1:

1. EM faculty will be able to discuss key basic biostatistical concepts from 2 pre-selected studies every other month
2. EM faculty will be able to cite sources of bias, both external and internal, in studies demonstrated in the primary literature.
3. EM faculty will be able to explain how a patient population included in a clinical trial is either similar to or different from the patients they serve.
4. EM faculty will describe how methodological issues or flaws in study’s design and reporting will influence their decision to act on its results in daily practice.

#### Goal 2

EM faculty will apply modern educational and adult learning theory to their clinical and bedside teaching.

Learning objectives for Goal 2:

1. EM faculty will be able to deliver excellent clinical teaching given the constraints of a busy emergency department
2. EM faculty will provide timely and effective feedback to their learners and produce robust and useful assessments on their progress

### Educational Strategies

See Curriculum Chart

Educational strategies used include virtual lecture supplemented by group discussion and small group discussion in journal club. We delivered a 10–15 minute virtual lecture on a clinical and/or educational topic which was augmented by real-time discussion of challenges and strategies where appropriate via Microsoft Teams. PowerPoint and supplemental materials were available for review via this application. Journal club was delivered in a hybrid format due to constraints on group gatherings due to the COVID-19 pandemic. The authors curated 2 articles for each session. These articles were supplemented with discussion questions to guide review as well as suggested podcasts. We hoped this would improve compliance with article review. Additionally, recent studies have shown many learners use podcasts as a top method of knowledge acquisition. We wanted our faculty to be familiar with this medium so they could prepare to discuss the validity of these resources with trainees. The journal club content is relevant to all learners, and clinical and educational topics are relevant to anyone who participates in medical education at the graduate or undergraduate levels.

### Results and tips for successful implementation

We implemented our curriculum during scheduled staff meetings to avoid increasing time demands. Journal club was held at a rotating time based on the preference of prospective core faculty. The curriculum is still ongoing; we are continuously reviewing to ensure delivery of high-yield content. The target is a 10-person core faculty; however, our department has 48 physicians and APPs, as well as rotating medical students. Quality improvement surveys were delivered to all learners via email for faculty development sessions. Pre- and post-surveys were distributed in person and virtually for Journal club. Basic statistics were performed using Microsoft Excel.

Seven out of nine core faculty responded to the survey (the lead author is a member of the core faculty but recused himself). Reviews for the faculty development sessions were uniformly positive. The primary question of interest, “Faculty development time makes me feel more prepared to be a clinical educator,” was rated 4.6 on a 5-point Likert scale where 5 represented “strongly agree.” They also indicated it was a valuable use of time during staff meetings (4.7/5). Similar approval was found for journal club (4.7/5). When surveyed regarding faculty development lectures, 4 out of 7 respondents agreed that the sessions led them to make changes to their clinical and educational practice, and the rest were neutral.

To obtain more specific information regarding faculty’s growth in response to journal club, we included a written pre- and posttest with one of the sessions. Three separate faculty members responded. They were asked 3 questions regarding biostatistical literacy both before and after the session. As shown in figure 1, all questions showed a nonsignificant trend toward improvement (mean difference 1.33 [p = 0.18], 0.67 [p = 0.18], and 2.33 [p = 0.09], respectively). We also assessed changes to clinical practice, as shown in figure 2, in response to the session. Again, we found a nonsignificant trend toward improvement (mean difference 0.67 [ p = 0.22] and 0.67 [p = 0.19], respectively).

Our ongoing review indicated that faculty prefer a combination of academic theory and clinical practice topics. We suspect this will be the case at other similar sites and recommend inserting topics that are relevant to the department in the lectures indicated in the appendices. Potential sources include interesting cases, departmental Quality Improvement projects, practice issues identified in morbidity and mortality conferences, and requests from faculty. We think the last is especially valuable to increase investment in the curriculum. We have included our chosen topics for reference of depth and breadth of instruction though these can be easily substituted based on local needs.

### Evaluation and Feedback

The only element of this curriculum that was poorly reviewed was the virtual journal club sessions. We hypothesize that a certain quorum must be present in-person to sustain critical mass for engaged discussion, less than what social distancing dictated was acceptable. Also, the social benefits including interaction with consultants from other departments outside of the clinical arena are lost. Now that distancing requirements have eased, we have returned to a hybrid model. This allows more robust attendance and dramatically improved participation.

The major weakness of this curriculum study is that its effectiveness in preparing faculty to teach residents was not evaluated because as of this writing, our graduate medical trainees have not started. EM is a required clerkship at our affiliated medical school and student evaluations of our site are consistently high, suggesting good efficacy.

### Associated Content

Curriculum Chart

Appendix 1: Curriculum Timeline

Appendix 2: Journal Club Discussion Questions and Answer Key

Appendix 3: Assessment PowerPoint

Appendix 4: Feedback PowerPoint

Appendix 5: Stroke Alert Update PowerPoint

Appendix 6: Bedside Clinical Teaching PowerPoint

Appendix 7: COVID Updates PowerPoint

Appendix 8: Push-dose Vasopressors PowerPoint

Appendix 9: Loop Drainage of Abscess and Bedside Procedure Teaching PowerPoint

Appendix 10: Atrial Fibrillation and “When You and Your Trainee Disagree” PowerPoint

Appendix 11: How to Give an Amazing “Chalk Talk” PowerPoint

Appendix 12: Bedside Teaching for the New Attending PowerPoint

Appendix 13: Status Epilepticus PowerPoint

## Figures and Tables

**Chart 1:**
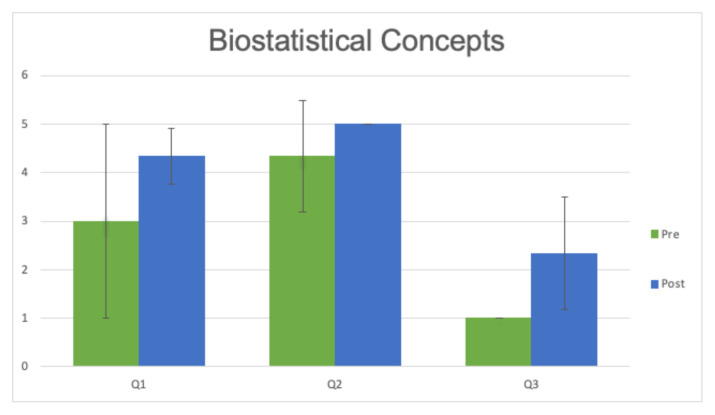
Sample pre- and post-Journal Club session self-assessment of biostatistical literacy rated on a 5-point Likert scale (No idea [1] to Know Cold [5]). Q1: I can explain the difference between a derivation and validation study and why it’s important. Q2: I can explain the difference between sensitivity/specificity and positive/negative predictive value. Q3: I know what it means to perform a recursive partitioning analysis.

**Chart 2:**
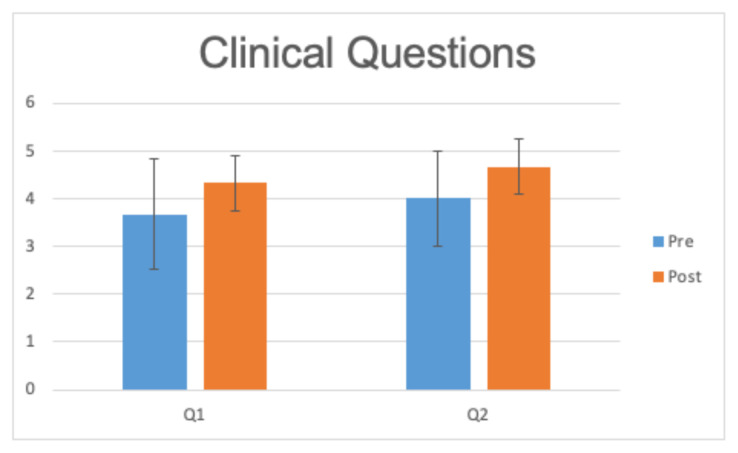
Pre- and post-session self-assessment of clinical concepts rated on a 5-point Likert scale (Strongly disagree [1] to Strongly Agree [5]). Q1: I feel like I know the current local standard evaluation of a well-appearing febrile infant. Q2: I am more confident in evaluating well-appearing febrile infants after this exercise.

## DIDACTICS AND HANDS-ON CURRICULUM

### Appendix 1 Curriculum Timeline

**Table:**

Topic	Recommended Educational Strategy	Educational Content	Objectives	Learners	Timing, Resources Needed (Space, Instructors, Equipment, Citations of JETem pubs or other literature)	Recommended Assessment, Milestones Addressed
Biostatistical literacy	Journal Club, topics as follows: -Neonatal fever -Adjusted D-Dimer Values to Rule out PE -Antibiotic only treatment of appendicitis - Round Table Discussion: “Pregnancy Adapted YEARSPE”	1) 2 primary literature articles assigned every other month. 2) Associated online resources provided with articles. 3) Small group discussion regarding articles, ideally includes a member of the applicable specialty team. 4) Summary sent electronically.	1) EM faculty will be able to discuss key basic biostatistical concepts from 2 pre-selected studies every other month. 2) EM faculty will be able to cite sources of bias, both external and internal, in studies demonstrated in the primary literature. 3) EM faculty will be able to explain how a patient population included in a clinical trial is either similar to or different from the patients they serve. 4) EM faculty will describe how methodological issues or flaws in a study’s design and reporting will influence their decision to act on its results in daily practice.	Attending physicians and available to advanced practitioners and rotating learners.	Sessions last approximately 1 hour. Month 1: Neonatal Fever (Appendix 2, page 1). Month 7: Adjusted Ddimer (Appendix 2, page 9). Month 10: Antibiotic only treatment of appendicitis (Appendix 2, page 11). Month 15: Round table discussion “Pregnancy-Adapted YEARS-PE” (Appendix 2, page 16).	Self-assessment.
Educational theory	Monthly brief lectures delivered during existing meeting time. List of covered topics: - Assessment - Feedback - Bedside clinical teaching - Clinical procedure topic and Bedside Procedure Teaching - Clinical topic and “When you and your trainee disagree” -How to give an excellent chalk talk	1) Standard PowerPoint lecture. 2) Learner-focused group discussion during and after.	1) EM faculty will be able to deliver excellent clinical teaching given the constraints of a busy emergency department. 2) EM faculty will provide timely and effective feedback to their learners and produce robust and useful assessments on their progress.	Attending physicians but delivered to advanced practitioners participating in meetings.	10–15 minutes, during existing department meetings. Month 2: Assessment. Month 3: Feedback. Month 5: Bedside Clinical teaching. Month 9: Bedside procedure teaching. Month 11: Clinical topic and “when you and your trainee disagree. Month 12: How to give an excellent chalk talk. Month 13: Bedside teaching for the new attending (spaced repetition).	Self-assessment.
Sample Clinical Education Topics	Monthly brief lectures delivered during existing meeting time. List of covered topics: - Stroke Alert Update - COVID Updates - Push-dose vasopressors - Status epilepticus	1) Standard PowerPoint lecture. 2) Learner-focused group discussion during and after.	1) EM Faculty will improve their practice-based learning by receiving timely updates on clinical topics relevant to departmental needs.	Attending physicians but delivered to advanced practitioners participating in meetings.	10–15 minutes, during existing department meetings. Month 4: Relevant clinical topic. Month 6: Relevant clinical topic. Month 8: Relevant clinical topic. Month 14: Relevant clinical topic.	Self-assessment

**Table:**

Educational Theory Topic	Objectives	Timing	Relevant Appendix
Assessment	1) Explain how COVID-19 has altered the sub-internship season for EM applicants and how these changes affect students and programs. 2) Discuss the role individual faculty play in the assessment of learners. 3) Recognize how to assess learners in a way that is beneficial to their growth while simultaneously informing their summative evaluations.	Month 2	3
Feedback	1) Define feedback. 2) Discuss how to make feedback effective. 3) Describe and utilize the ARTful feedback method.	Month 3	4
Bedside Clinical Teaching	1) Understand how to teach effectively in a brief amount of time. 2) Appreciate different strategies that can be used to engage learners. 3) Describe how to maximize the educational value of a clinical encounter.	Month 5	6
Loop Drainage of Abscess (or other clinical topic) and Bedside Procedure Teaching	1) Explain the pitfalls of physical exam in evaluating abscess. 2) Describe the loop vessel technique and which populations it might be most useful for. 3) Appreciate how to guide novice learners through a new procedure at bedside.	Month 9	9
Atrial Fibrillation (or other clinical topic) and “When you and your trainee disagree”	1) Explain which patients with atrial fibrillation are candidates for cardioversion. 2) Discuss which patients we discharge after cardioversion should be started on anticoagulation. 3) Evaluate strategies to manage the unstable patient with atrial fibrillation. 4) Resolve disagreements with trainees with regards to patient management.	Month 11	10
How to give an amazing chalk talk	1) Explain how to design and deliver a high quality-chalk talk. 2) Discuss evidence-based strategies for preparing lectures for adult learners. 3) Understand how to adapt lectures to a virtual format.	Month 12	11
Bedside Procedure Teaching	1) Discuss the importance of patient-centered procedure teaching. 2) Describe multiple modern frameworks for procedural teaching. 3) Discuss how to maintain learner autonomy during critical procedures.	Month 13	12

**Table:**

Sample Clinical Education Topic*	Objectives	Timing	Relevant Appendix
A New Stroke Alert Pathway	1) Understand the different stroke treatment options available depending on time of symptom onset. 2) Appreciate the role of the telestroke neurologist. 3) Explain which patients are candidates for intervention outside the tPA window. 4) Apply the RACE score to evaluate for potential large vessel occlusion (LVO) strokes.	Month 4	5
COVID Updates	1) Updates to recommendations for care of elderly people, athletes, and general return to work. 2) Most recent literature for pharmacologic therapy. 3) Critical care insights. 4) Discussion of MIS-C.	Month 6	7
Push-Dose Vasopressors	1) Understand the evidence (or lack thereof) behind use of bolus-dose vasopressors in the ED. 2) Describe pharmacology, indications, and dosing of common agents. 3) Explain common safety issues and how to prevent them. 4) Discuss how to educate novice learners on this topic.	Month 7	8
Status Epilepticus	1) Discuss adequate dosing of benzodiazepines. 2) Describe “second-line” agents and their indications and dosing. 3) Explain which induction agents and general anesthetics are useful. 4) Discuss exceptions in pediatric patients.	Month 14	13

* Please note that these topics are not mandatory. We chose them based on our departments needs as identified by morbidity and mortality conference, quality improvement projects, and most importantly, feedback from our faculty. We are providing what we did to give a sense of scale, but we recommend you substitute based on local needs as determined above.

### Appendix 2 Journal Club Discussion Questions and Answer Key

#### Month 1: Approach to Neonatal Fever

##### Article 1: Step-By-Step Approach

Gomez B, Mintegi S, Bressan S, Da Dalt L, Gervaix A, Lacroix L. Validation of the “Step-by-Step” Approach in the Management of Young Febrile Infants. *Pediatrics*. August 2016; 138 (2): e20154381. Doi: 10.1542/peds.2015-4381

##### Recommended podcasts

- Validation of the “step-by-step” Approach in The Management of Young Febrile Infants. December 2016. https://www.emrap.org/episode/ema-2016-12/abstract20
- EM:RAP: Step-by-step again. January 2017. https://www.emrap.org/episode/brokenankles/paperchase3step
- EM:RAP: Pediatric Pearls: Pediatric fever step by step. December 2017. https://www.emrap.org/episode/pneumoniainthe/pediatricpearls1

##### Discussion Questions

Describe the study? Which patients were included? Which were excluded?

What was the comparison to the tool they propose?

Describe the difference between sensitivity/specificity and positive/negative predictive value. Which characteristics do we care about as emergency physicians?

Explain the difference between “derivation” and “validation” in determining a clinical decision rule. Why is it important to do both? What characteristics of a validation study make a rule more generalizable?

What was the prevalence of the outcome of interest in the study population? How do you think this affects the usefulness of the rule?

How many patients with IBI (invasive bacterial infection) did the rule miss? What are some common characteristics of these patients?

##### Article 2: PECARN Approach

Kuppermann N, Dan PS, Levine DA, et al. A clinical prediction rule to identify febrile infants 60 days and younger at low risk for serious bacterial infections. *JAMA Pediatr*. Published ahead of print February 18, 2019. doi:10.1001/jamapediatrics.2018.5501

##### Recommended podcasts

- EM:RAP EMA: Abstract 1: Predicting Febrile < 60 days As Low Risk For SBI. December 2019. https://www.emrap.org/episode/ema2019/abstract1
- EM:RAP: Pediatric Pearls: Fever in the first 60 days – the latest tool. July 2019. https://www.emrap.org/episode/emrap2019july/pediatricpearls

##### Discussion Questions

Describe the study? Which patients were included? Which were excluded? How is this different from the first article?

Explain recursive partitioning analysis.

Discuss their statistical methods. Do they strike you as appropriate?

Discuss the results. Do you agree with their conclusions? Any limitations?

##### Summary Questions

Did you listen to the podcasts? Does their assessment of the articles agree with yours? Why or why not?

Will you change your practice as a result of this discussion? Why or why not? If yes, how? Specifically think about which patients need LP (lumbar puncture) as part of their workup.

There are some standard characteristics of the care provided. How might this differ from our local practice?

#### Pre-Discussion Reflection

Please rate your confidence with the following biostatistical concepts PRIOR TO READING THE ARTICLES AND DISCUSSION QUESTIONS on a scale from 1 (No idea) to 5 (know cold and could teach to others):

I can explain the difference between a derivation and validation study and why it’s important.

**Table:**

1	2	3	4	5

I can explain the difference between sensitivity/specificity and positive/negative predictive value.

**Table:**

1	2	3	4	5

I know what it means to perform a recursive partitioning analysis.

**Table:**

1	2	3	4	5

Please respond to these clinical questions on a scale of 1 (strongly disagree) to 5 (strongly agree). 3 is neutral.

I feel like I know the current local standard evaluation of a well-appearing febrile infant.

**Table:**

1	2	3	4	5

My practice is similar to that of my peers.

**Table:**

1	2	3	4	5

Did you listen to any of the podcasts?

**Table:**

Yes	No

If no, why not (check all that apply)

**Table:**

__No time
__Don’t have subscription
__Not interested
Other (please explain) _______________________________

If yes, did you agree with their interpretation of the articles?

**Table:**

__Yes to both
__No to both
__Yes to one, no to one

Please explain your answer to the above question in 1–2 sentences here.

#### Post-Discussion Reflection

After reading the papers, discussion questions, and participating in the group discussions, please rate your confidence with the following biostatistical concepts:

I can explain the difference between a derivation and validation study and why it’s important.

**Table:**

1	2	3	4	5

I can explain the difference between sensitivity/specificity and positive/negative predictive value.

**Table:**

1	2	3	4	5

I know what it means to perform a recursive partitioning analysis.

**Table:**

1	2	3	4	5

Please respond to these clinical questions on a scale of 1 (strongly disagree) to 5 (strongly agree). 3 is neutral.

I feel like I know the current local standard evaluation of a well-appearing febrile infant.

**Table:**

1	2	3	4	5

I am more confident in evaluating well-appearing febrile infants after this exercise.

**Table:**

1	2	3	4	5

I will make changes to my practice based on what I learned here.

**Table:**

1	2	3	4	5

My practice is similar to that of my peers.

**Table:**

1	2	3	4	5

Did you perceive commercial bias?

**Table:**

Yes	No

Do you feel that the group agreed with the podcasts? Why or why not?

Will you change your practice as a result of this activity?

**Table:**

Yes	No	Not sure

(Turn over)

If no, why not?

**Table:**

__I’m an early adopter; I was doing this already
__This sounds promising, but I worry it’s not settled standard of care
__Other (please explain)

If yes, please explain what changes you will make specifically?

Please rate the following statements from 1 (strongly disagree) to 5 (strongly agree). 3 is neutral.

Journal club provides me with valuable information and is worth my time.

**Table:**

1	2	3	4	5

I have grown as a clinician thanks to journal club.

**Table:**

1	2	3	4	5

Journal club makes me feel more prepared to be a clinical educator.

**Table:**

1	2	3	4	5

Do you have any feedback for how this can be made more beneficial to you?

Do you have any suggestions for topics you would like to see covered in the future?

#### Month 1: Approach to Neonatal Fever Answer Key

##### Summary

These are two well-written articles which potentially allow us to do less invasive testing on our youngest and potentially most stress-inducing patients. The group consensus is that we are not ready to abandon the lumbar puncture (LP) in patients less than 28 days old; however, if family has significant hesitancy about this procedure, this is an avenue for shared decision making. We generally agreed with the podcasts’ take on the articles. Our guest pediatrician reminds us that if we forego the LP we should avoid antibiotics (remember these are well-appearing neonates) so as not to cloud the picture for meningitis in the future. Also, 10% of babies with a documented respiratory virus (Flu, RSV) have a urinary tract infection, so you still need a urine sample.

##### *Author’s note*

Potentially sick children are a major source of stress for emergency providers. Community emergency physicians don’t perform many neonatal LPs, so this is an additional stressor. Furthermore, these articles highlight the process of deriving and validating a clinical decision rule and the PECARN article includes some advanced statistical analysis. Our group conducted this session before the COVID-19 pandemic, and before the American Academy of Pediatrics (AAP) changed their guidelines on this population, so your discussion may vary, and including the new AAP guidelines as a reference would be helpful. We also included a sample curricular assessment tool.

#### Month 7: New Pulmonary Embolism (PE) Rule Out Strategies

##### Article 1: Pretest Probability-Adjusted Dimer

Kearon C, de Wit K, Parpia S, et al. Diagnosis of pulmonary embolism with d-dimer adjusted to clinical probability. *N Engl J Med*. 2019;381(22):2125–2134.

##### Recommended podcast

- Abstract 1: Diagnosis of pulmonary embolism with d-dimer adjusted. EM:RAP EMA. March 2020. https://www.emrap.org/episode/ema2020march/abstract1

##### Discussion questions

Describe the study. What were the inclusion and exclusion criteria? Is this similar to our patient population? Describe the algorithm they used.

Describe their methods. Do you think it was okay to use a convenience sample? Did they prespecify their secondary outcomes?

Discuss the results. Can anyone explain table 3?

Most of the patients in this sample are low pretest probability. Is this acceptable?

Patients in whom imaging was ordered against protocol were excluded. Do you agree with their discussion of why this is okay? All those lost to follow up were low pretest. Discuss how this affects results, or not.

If you listened to the podcast, explain how they agreed/disagreed with your interpretation.

Will you change your practice based on this study. Why or why not?

##### Article 2: Pregnancy Adjusted YEARS

van der Pol LM, Tromeur C, Bistervels IM, et al. Pregnancy-Adapted YEARS Algorithm for Diagnosis of Suspected Pulmonary Embolism. *N Engl J Med*. 2019 Mar 21;380(12):1139–1149.

##### Recommended podcast

- EM:RAP EMA: Abstract 21: Pregnancy-adapted YEARS algorithm. August 2019. At : https://www.emrap.org/episode/ema2019august/abstract21

##### Discussion Questions

Describe the study. What were the inclusion and exclusion criteria? Is this similar to our patient population? Describe the algorithm.

Describe the methods.

What is the difference between a per protocol and an intention to treat analysis?
What ultrasound strategy did they use? What do we use? How should this affect results?

Discuss the results of the study.

How did results differ by trimester? Do you agree with their explanation?

If you listened to the podcast, explain how they agreed/disagreed with your interpretation.

Will you change your practice as a result of this study?

##### Supplemental Resources

- Arora S and Menchine M. Abstract 3: Multicenter Evaluation of YEARS Criteria For PE. EM:RAP. March 2019. At: https://www.emrap.org/episode/ema2019march/abstract3
- Kabrhel C, Van Hylckama Vlieg A, et al. Multicenter Evaluation of The YEARS Criteria in Emergency Department Patients Evaluated for Pulmonary Embolism. *Acad Emerg Med;* 25(9):987, September 2018.
- Mattu A, Swaminathan A. Cardiology Corner: Pulmonary embolism updates. EM:RAP. November 2020. At: https://www.emrap.org/episode/emrap202014/cardiology
- Mason J, Herbert M, Swadron S. C3: Pulmonary Embolism. EM:RAP. December 2019. At: https://www.emrap.org/c3/playlist/cardiovascular/episode/c3pulmonary/c3pulmonary
- Farkas J. Submassive and Massive PE. Internet Book of Critical Care. September 2021. At: https://emcrit.org/ibcc/pe/

#### Month 7: New Pulmonary Embolism (PE) Rule Out Strategies Answer Key

##### Article 1: Pretest Probability-Adjusted Dimer

Kearon C, de Wit K, Parpia S, et al. Diagnosis of pulmonary embolism with d-dimer adjusted to clinical probability. *N Engl J Med*. 2019;381(22):2125–2134.

##### Recommended podcast

- Abstract 1: Diagnosis of pulmonary embolism with d-dimer adjusted. EM:RAP EMA. March 2020. https://www.emrap.org/episode/ema2020march/abstract1

This was an overall well-done study looking at the accuracy of using a higher D-dimer threshold to exclude PE in patients with a low pretest probability of disease, defined as a Wells score < 4. The study was conducted in adult outpatients (ie, ED patients) presenting to a university hospital in Canada. Patients with recent major surgery, active anticoagulation, life expectancy < 3 months, and a dimer known to the treating provider prior to assessment of the pretest probability were excluded. Strengths included < 5% loss to follow up and that the study was conducted on ED patients. This was a convenience sample, which is not ideal. The algorithm performed very well with no patients who had a low or moderate pretest probability and a negative dimer who were “ruled out” having venous thromboembolism (VTE) at 90 day follow up. They also report a substantial reduction in use of computerized tomography (CT) imaging compared to standard dimer threshold as well as YEARS and age-adjusted dimer approaches.

Overall, we liked the article and thought it was potentially practice changing, but no one in the group was ready to start using this in their clinical practice without some external validation or total buy-in from the rest of the group. At the very least, this study can be used in shared decision making with patients who might be anxious about receiving radiation or contrast. Entry into this study required some suspicion for PE beyond typical chief complaint so be wary of using this to indiscriminately order D-dimers on all comers.

Below is a summary of their algorithm

Clinical suspicion for PE → Apply Wells score

##### Article 2: Pregnancy Adjusted YEARS

van der Pol LM, Tromeur C, Bistervels IM, et al. Pregnancy-Adapted YEARS Algorithm for Diagnosis of Suspected Pulmonary Embolism. *N Engl J Med*. 2019 Mar 21;380(12):1139–1149.

##### Recommended podcast

- EM:RAP EMA: Abstract 21: Pregnancy-adapted YEARS algorithm. August 2019. At : https://www.emrap.org/episode/ema2019august/abstract21

This is another well-done article answering the uncommon, but always frustrating, question of what to do with pregnant patients whose clinical presentation raises concern for a diagnosis of PE. This study used a modified YEARS protocol to evaluate pregnant patients with a complaint of NEW/WORSE chest pain and/or shortness of breath presenting to the emergency department. They excluded the critically ill, those on anticoagulation, with contrast allergy, or with inability to follow up. Overall, the study performed very well in excluding VTE with only one missed deep vein thromboembolism (DVT) discovered at 90 day follow up.

There are a few items of note here. First is that this technically wasn’t a randomized controlled trial. Second is that all the patients lost to follow up were in the low-risk group but this would not have affected the results. They also used only 2-point compression US for DVT studies, which is theoretically LESS sensitive than the multi-site compression with doppler we get in our formal studies. The algorithm was equally safe in all trimesters but was less effective as a rule as trimester increased. This makes physiological sense because we know that D-dimer increases throughout pregnancy, and third trimester is when reflux and some of the physiologic breathlessness symptoms of normal pregnancy are at a peak. Again, this was not a screening tool for all comers; there had to be some suspicion for PE based on attending assessment. The prevalence of PE in this study was similar to the other one, highlighting that, in general, active pregnancy isn’t a huge independent risk factor. The postpartum period is, however, and none of us would think twice about radiation then.

Group consensus was more favorable for changing our practice based on this article versus the first, simply because there seems to be no standard way of ruling these patients out and this provides a formal algorithm to do so.

##### *Author’s Note*

This session was conducted virtually, and it did not go particularly well. This was done earlier in the pandemic so there may have been some virtual talk novelty at play. If implementing this again, we would have focused more on discussion of challenging cases and barriers to implementation to start discussion rather than leading with the formal biostatistics material. This is an example of important, recent, and potentially practice-changing articles which we recommend as journal club material.

#### Month 9: Antibiotic Only Strategy for Appendicitis

Does all appendicitis need urgent surgery? Every emergency physician has encountered the patient whose appendicitis couldn’t have occurred at a worse time for them or is understandably nervous about surgery. Which patients might even be candidates for nonoperative management of their appendicitis? We’ll be discussing the seminal paper on this topic (APPAC) and the most recent American paper (CODA).

##### Article 1: APPAC Trial (2015)

Salminen P, Paajanen H, Rautio T, et al. Antibiotic Therapy vs. Appendectomy for Treatment of Uncomplicated Acute Appendicitis: The APPAC Randomized Clinical Trial. *JAMA*. 2015;313(23):2340–2348. doi:10.1001/jama.2015.6154

##### Recommended Podcast

- Antibiotic Therapy Vs. Appendectomy for Treatment Of Uncomplicated Acute Appendicitis: The APPAC Randomized Trial. EMA. November 2015. At: https://www.emrap.org/episode/ema-2015-11/abstract20

##### Discussion questions

Describe the study. Where was it conducted? What were the inclusion and exclusion criteria? Discuss their protocol.

Describe the results.

What is a non-inferiority trial? When would you conduct such a thing?

Discuss secondary outcomes. Do these make sense?

If you listened to the podcast, does your interpretation agree with theirs?

Would you change your practice based on this study?

##### Article 2: CODA Trial (2020)

Flum DR, Davidson GH, Monsell SE, et al. A Randomized Trial Comparing Antibiotics with Appendectomy for Appendicitis. *N Engl J Med*. 2020 Nov 12;383(20):1907–1919. Epub 2020 Oct 5. PMID: 33017106. At: doi: 10.1056/NEJMoa2014320

##### Recommended Podcasts

- Arora S, Menchine M. Abstract 7: A Randomized Trial Comparing Antibiotics with Appendectomy. February 2021. At: https://www.emrap.org/episode/ema2021february/abstract7a
- Talan D, Herbert M. The CODA Trial. October 2020. At: https://www.emrap.org/episode/thecodatrial/thecodatrial

##### Discussion questions

Describe the study. What were the exclusion and inclusion criteria? What was their study protocol? How are these different from the APPAC study?

Describe the results.

What is the difference between intention to treat and per protocol analysis? When should you use which?

What is a pragmatic study design? What are strengths and weaknesses of this approach?

Notice table 1 in each study. What do you think of the fever and inflammatory markers?

If you listened to the podcast, does your interpretation agree with theirs?

Would you change your practice based on this study? If your answer was different here than with the APPAC study, why is that?

##### Supplemental Material

- **Five Year Follow Up**
  ○ Salminen P, Tuominen R, Paajanen H, et al. Five-Year Follow-up of Antibiotic Therapy for Uncomplicated Acute Appendicitis in the APPAC Randomized Clinical Trial. *JAMA*. 2018 Sep 25;320(12):1259–1265. Erratum in: *JAMA*. 2018 Oct 23;320(16):1711. PMID: 30264120. PMCID: PMC6233612. doi: 10.1001/jama.2018.13201
  ○ Arora S, Menchine M. Abstract 11: APPAC - 5-Yr Follow-Up Of Abx Therapy For Appendicitis. March 2019. At: https://www.emrap.org/episode/ema2019march/abstract11appac
- **Quality of Life at Follow Up**
  ○ Arora S, Menchine M. Abstract 1: 7-year follow-up of antibiotic therapy for acute appendicitis. June 2020. https://www.emrap.org/episode/ema2020june/abstract17year
  ○ Sippola S, Haijanen J, Viinikainen L, et al. Quality of Life and Patient Satisfaction at 7-Year Follow-up of Antibiotic Therapy vs. Appendectomy for Uncomplicated Acute Appendicitis: A Secondary Analysis of a Randomized Clinical Trial. *JAMA Surg*. 2020 Apr 1;155(4):283–289. PMID: 32074268 doi: 10.1001/jamasurg
- **Oral Antibiotics for acute appendicitis**
  ○ Sippola S, Haijanen J, Grönroos J, et al. Effect of Oral Moxifloxacin vs. Intravenous Ertapenem Plus Oral Levofloxacin for Treatment of Uncomplicated Acute Appendicitis: The APPAC II Randomized Clinical Trial. *JAMA*. 2021;325(4):353–362. doi:10.1001/jama.2020.23525

#### Month 9: Antibiotic Only Strategy for Appendicitis Answer Key

##### Article 1: APPAC Trial (2015)

Salminen P, Paajanen H, Rautio T, et al. Antibiotic Therapy vs. Appendectomy for Treatment of Uncomplicated Acute Appendicitis: The APPAC Randomized Clinical Trial. *JAMA*. 2015;313(23):2340–2348. doi:10.1001/jama.2015.6154

##### Recommended Podcast

- Antibiotic Therapy Vs. Appendectomy for Treatment Of Uncomplicated Acute Appendicitis: The APPAC Randomized Trial. EMA. November 2015. At: https://www.emrap.org/episode/ema-2015-11/abstract20

This was a randomized, open label noninferiority trial comparing Finnish patients randomized to open appendectomy vs. antibiotic only therapy. Included patients ages 18–60 who presented to the ED and had CT-confirmed uncomplicated acute appendicitis. Exclusion criteria included complicated appendicitis (appendicolith, perforation, abscess, suspected tumor), contraindication to CT (including active metformin use, renal insufficiency, pregnant/lactating, iodine allergy), peritonitis on exam, systemic illness. The surgery group was treated with open (in almost all cases) appendectomy with only antibiotics being perioperative cefuroxime and metronidazole. The antibiotics only group was hospitalized for 3 days and received 1g/day IV ertapenem, then transitioned to oral levofloxacin/metronidazole.

All but one patient in the surgical group had successful appendectomy. In the antibiotics group, 27% of patients ultimately had appendectomy within 1 year of presentation. 5 of these were negative on histopathology.

A noninferiority trial tests whether a treatment is not worse than some comparator by the pre-specified noninferiority margin. It is not the opposite of the common “superiority” trial and does not check for equality because in a big enough sample there is always some minute difference between groups. It can show equivalence, however. The primary reasons to use this are ethical (there’s a treatment so using a placebo would be unethical), cost (these are generally less expensive than placebo studies), and safety (there is a safety or other patient-centered benefit to the new treatment). In general, these are done when there’s a new/different treatment strategy that has some benefit over the standard of care (like avoiding surgery in this case).

Overall complications were higher in the surgery group driven by post-operative complications such as wound infections, hernias, and obstructive symptoms. People in the antibiotics group stayed longer, but that length of stay was specified in trial protocol. Pain at discharge and at 1 week was less in the antibiotics group but the same by 2 months out. The patients who got only antibiotics also had much less sick leave.

##### Article 2: CODA Trial (2020)

Flum DR, Davidson GH, Monsell SE, et al. A Randomized Trial Comparing Antibiotics with Appendectomy for Appendicitis. *N Engl J Med*. 2020 Nov 12;383(20):1907–1919. Epub 2020 Oct 5. PMID: 33017106. At: doi: 10.1056/NEJMoa2014320

##### Recommended Podcasts

- Arora S, Menchine M. Abstract 7: A Randomized Trial Comparing Antibiotics with Appendectomy. February 2021. At: https://www.emrap.org/episode/ema2021february/abstract7a
- Talan D, Herbert M. The CODA Trial. October 2020. At: https://www.emrap.org/episode/thecodatrial/thecodatrial

This is a pragmatic, open label noninferiority randomized trial comparing antibiotic therapy with laparoscopic appendectomy conducted at several sites in the US. Consecutive adults older than 18 attending an ED and who had appendicitis confirmed by imaging. Exclusion criteria included septic shock, diffuse peritonitis, recurrent appendicitis, severe phlegmon determined by surgeon to require more extensive resection, walled-off abscess, free air, neoplasm, or more than minimal free fluid. Notably, they included patients with an appendicolith and sorted them into a pre-specified subgroup. They also included small perforations where the other exclusion criteria were absent so this is a sicker cohort than was included in APPAC.

The antibiotics protocol required only 24 hours of IV antibiotic therapy followed by oral medications for a total of 10 days. They could be discharged after 24 hours of therapy or after receiving a 24-hour IV dose provided they met the usual clinical discharge criteria of tolerating oral intake, pain control, and improvement. Care post-randomization was not standardized. Surgical treatment was not standardized, but 96% had a laparoscopic procedure. Primary outcome was scored on the EQ-5D questionnaire.

Mean scores on the questionnaire were essentially the same. This was true whether there was an appendicolith or not. Appendectomy was performed in 29% by 90 day follow up (41% of those with appendicolith). Secondary outcomes as follows: Time to symptom resolution and duration of hospitalization were essentially the same. Return visits to ED or repeat hospitalization were more frequent in antibiotics group, driven by need for appendectomy. Missed work for patient and caregiver favors the antibiotics group.

##### *Author’s Note*

This was a very successful session held in a hybrid format which significantly helped attendance. These articles are potentially paradigm shifting and have garnered some coverage in the lay press. Important biostatistics points include intention to treat versus per protocol analysis and noninferiority study design. The CODA trial particularly allowed discussion of pragmatic RCT designs which are increasingly common and therefore important in bolstering faculty’s biostatistical literacy.

#### Month 15: Multispecialty Discussion of the Pregnancy-Adapted YEARS PE Algorithm

##### Article 1: Pregnancy Adjusted YEARS

van der Pol LM, Tromeur C, Bistervels IM, et al. Pregnancy-Adapted YEARS Algorithm for Diagnosis of Suspected Pulmonary Embolism. *N Engl J Med*. 2019 Mar 21;380(12):1139–1149.

##### Background on the paper

One of the most important parts of understanding a paper is understanding who it DOESN’T apply to. In order to be included, patients had to present to the ED with new or worse chest pain or shortness of breath, and it wasn’t applied to all-comers, so there had to be some suspicion for PE by the attending physician. They EXCLUDED the critically ill, those on anticoagulation, with contrast allergy, or with inability to follow up. In general, the methodology was excellent, there was minimal loss to follow up, and the algorithm performed very well. This wasn’t technically a trial, but it is about as close as we’ll get in this population. Rate of PE/DVT was similar to other studies, so this population probably isn’t as high risk as we sometimes imagine it to be.

##### Specialist Input

We were joined by two of our colleagues from radiology. Their sense of current practice is that we adequately avoid radiation in these patients. They use special CT protocols in pregnant patients to limit radiation exposure to both mom and fetus. Sometimes a shield is used but if used incorrectly will actually increase the radiation dose the fetus receives. We should be getting chest X-ray (CXR) in these patients because sometimes this will reveal a diagnosis and is useful in follow up. They also highlighted that CT imaging in a coordinated healthcare system such as ours can be useful in monitoring non-acute findings.

##### Discussion and Bottom Line

Almost all participants felt that using a risk-adjusted dimer would be appropriate. Important considerations include challenges brought about by long wait times because very low risk patients don’t even require dimer testing, and this can be a nuanced decision requiring information potentially not available during the medical screening exam (MSE). Another issue is that our lab will flag a result as abnormal even though it’s still “negative” at the high threshold which is a potential challenge with patients having access to their lab results.

##### *Author’s Note*

We had excellent engagement when we invited our specialist colleagues to discuss and join us in a moderated discussion. This article was covered already in a previous journal club, so a simple review of the basic biostatistics elements was all that was required. These were presented ahead of time and attendees were encouraged to bring questions to ask our radiology colleagues. We chose this particular paper because of poor engagement with it in the previous section and out of a hope that the material was novel and practice changing. It is presented here as an example of how one might set up a multi-specialty discussion panel. A paper relevant to a recent interesting or morbidity/mortality case would also work. We would recommend including a sample case from your department relevant to the discussion. An example from this section below:

24-year-old G1P0 female currently 29 weeks pregnant presents with shortness of breath for the past 3 days. It is associated with a “sickness” in her chest, congestion, and sore throat. No relief with albuterol inhaler, prompting visit. No fever or cough. No COVID exposure. Normal fetal movement, no bleeding, no contractions

Vitals: 117/99, heart rate 99, respiratory rate 18, oxygen saturation 98%, temperature 37.2°C

Exam: Appears anxious, nontoxic

Clear lungs

No extremity swelling

Group discussion of how they would work this up, input from specialists.

Case resolution

CXR: Left lower lobe infiltrate

Dimer: Not ordered

CT PE: Ordered, negative

Final Dx: COVID Pneumonia

### Appendix 3 Assessment PowerPoint

Please see associated PowerPoint file

### Appendix 4 Feedback PowerPoint

Please see associated PowerPoint file

### Appendix 5 Stroke Alert Update PowerPoint

Please see associated PowerPoint file

### Appendix 6 Bedside Clinical Teaching PowerPoint

Please see associated PowerPoint file

### Appendix 7 COVID Updates PowerPoint

Please see associated PowerPoint file

### Appendix 8 Push-dose Vasopressors PowerPoint

Please see associated PowerPoint file

### Appendix 9 Loop Drainage of Abscess and Bedside Procedure Teaching PowerPoint

Please see associated PowerPoint file

### Appendix 10 Atrial Fibrillation and “When You and Your Trainee Disagree” PowerPoint

Please see associated PowerPoint file

### Appendix 11 Highlights from CORD PowerPoint

Please see associated PowerPoint file

### Appendix 12 Bedside Teaching for the New Attending PowerPoint

Please see associated PowerPoint file

### Appendix 13 Status Epilepticus PowerPoint

Please see associated PowerPoint file
