# Supplementary material for: Residents Are Coming: A Faculty Development Curriculum to Prepare a Community Site For New Learners
Source: J Educ Teach Emerg Med. 2022 Jul 15;7(3):C1–C41. doi: 10.21980/J87D2N (PMC10332697; doi:10.21980/J87D2N)
Supplement: Supplementary file 1 — Please see associated PowerPoint file [file jetem-7-3-c1-appendix3.pptx]

## Slide 1
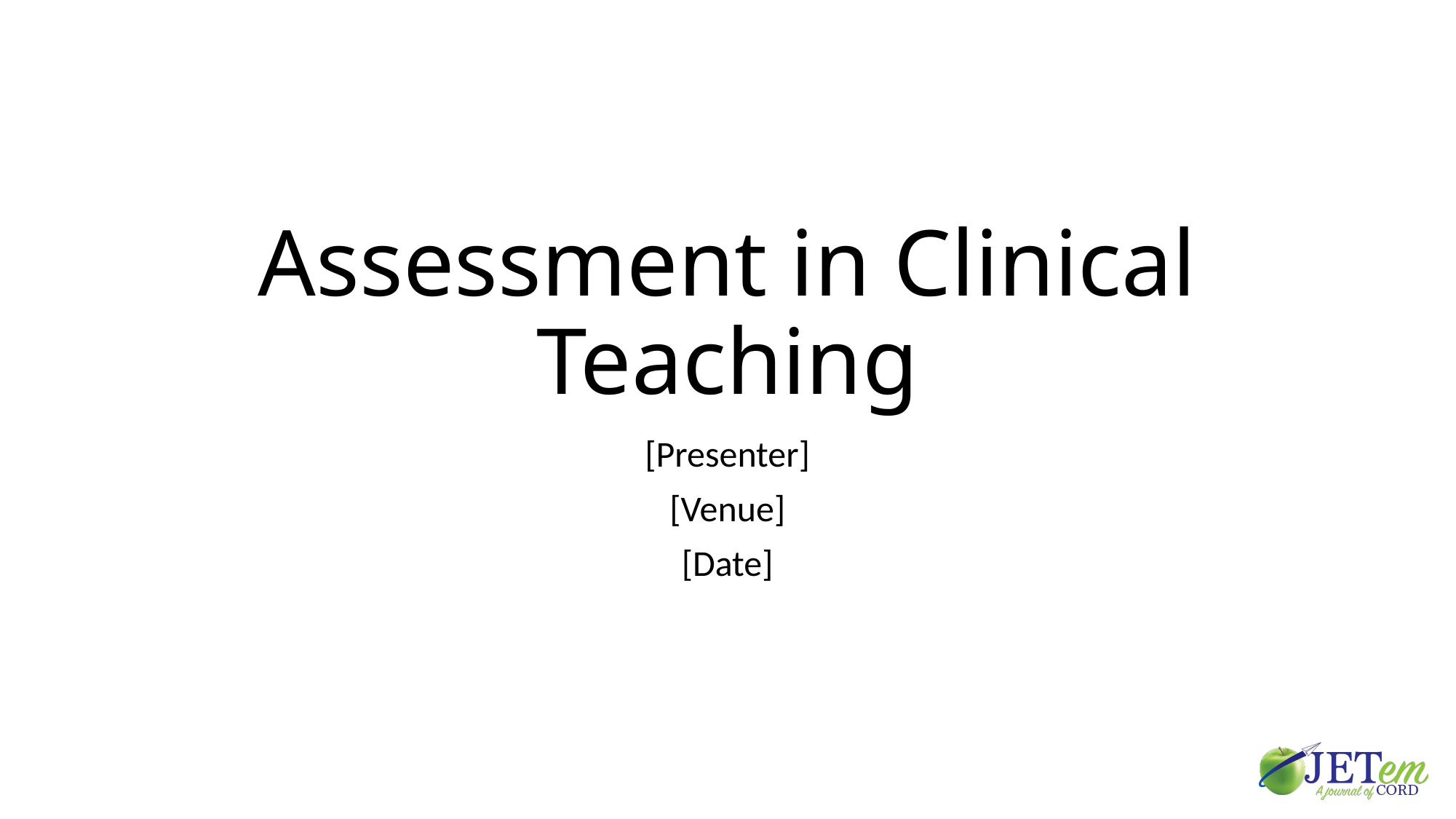

# Assessment in Clinical Teaching
[Presenter]
[Venue]
[Date]

## Slide 2
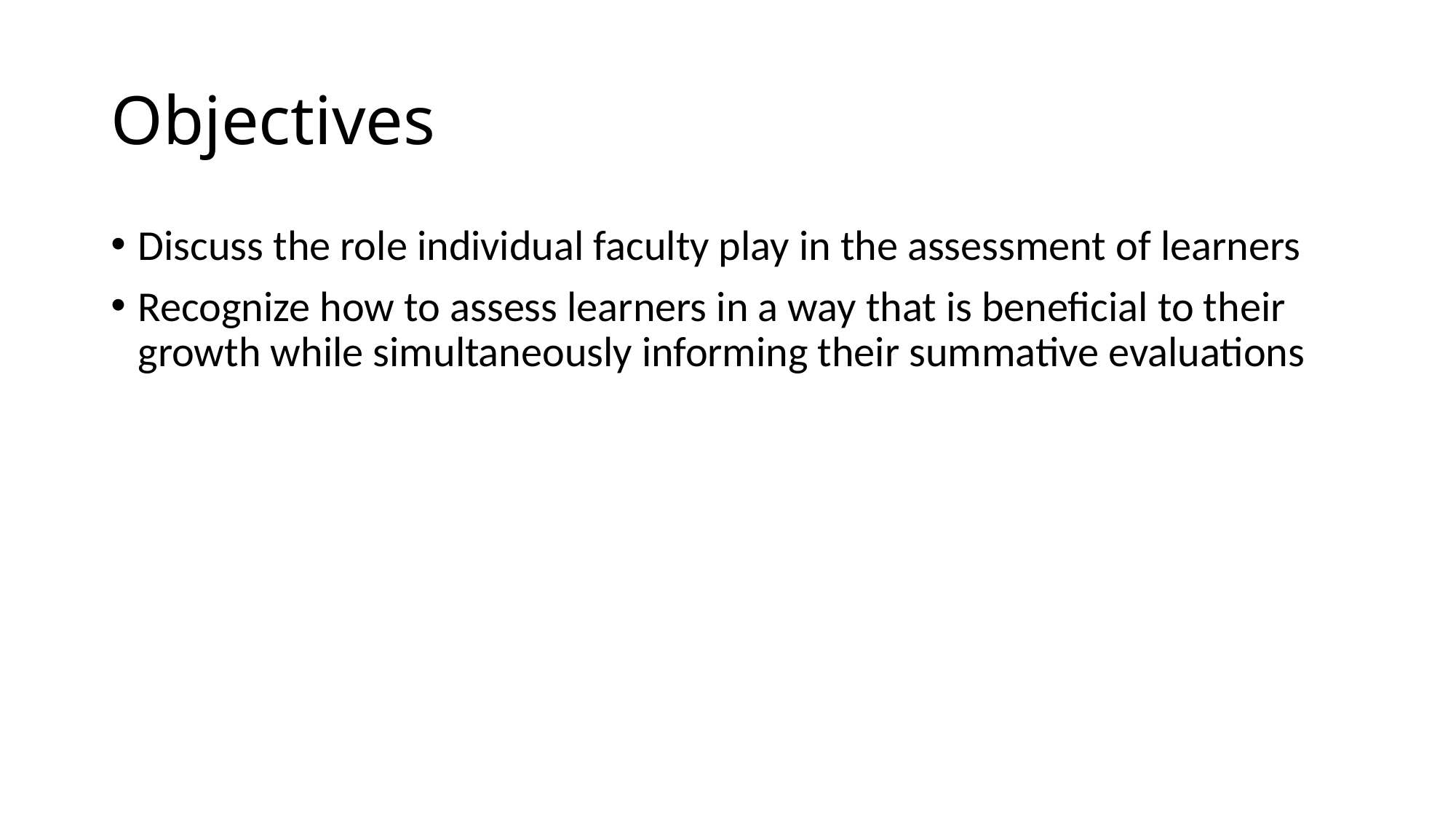

# Objectives
Discuss the role individual faculty play in the assessment of learners
Recognize how to assess learners in a way that is beneficial to their growth while simultaneously informing their summative evaluations

## Slide 3
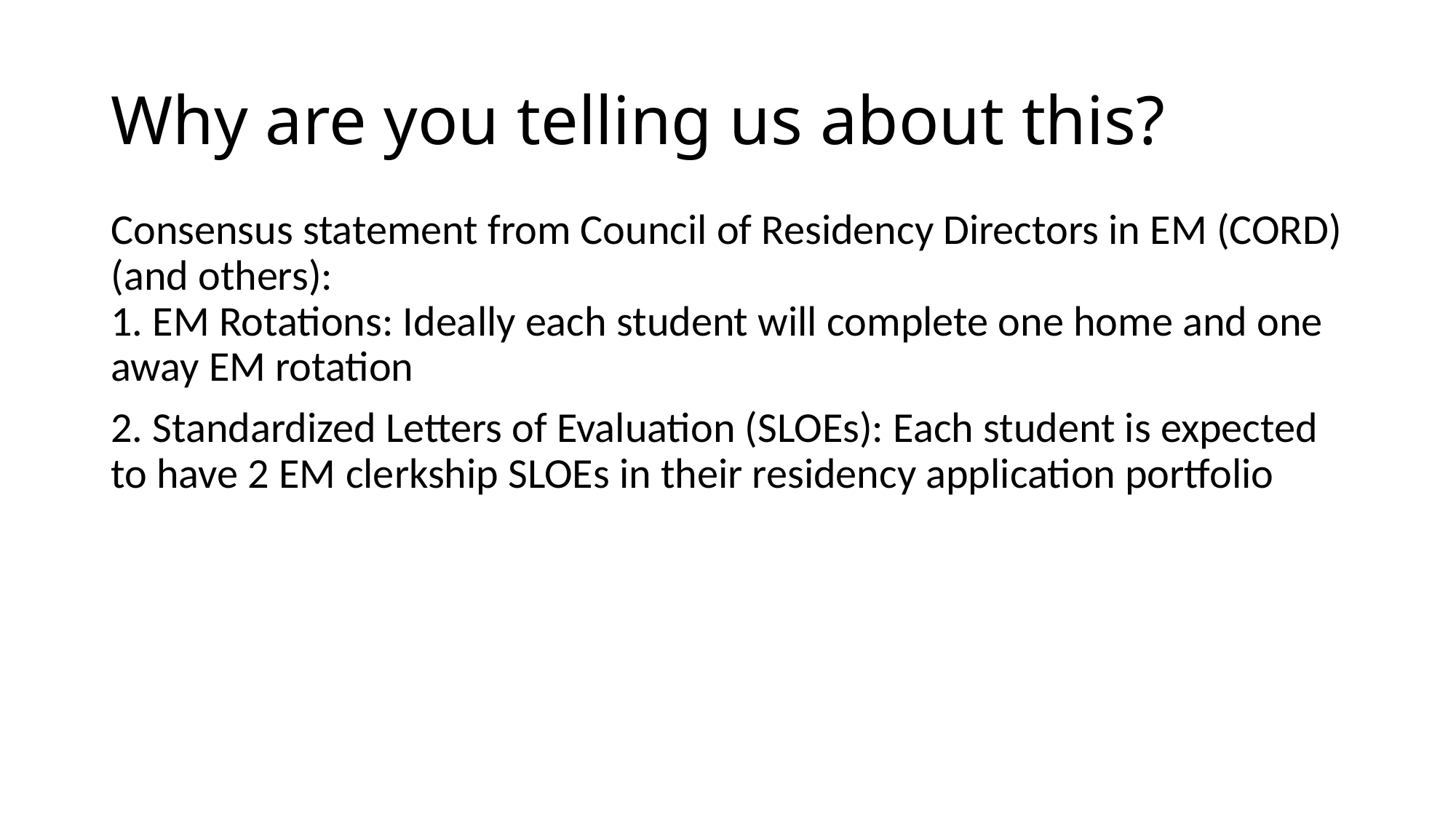

# Why are you telling us about this?
Consensus statement from Council of Residency Directors in EM (CORD) (and others):
1. EM Rotations: Ideally each student will complete one home and one away EM rotation
2. Standardized Letters of Evaluation (SLOEs): Each student is expected to have 2 EM clerkship SLOEs in their residency application portfolio

## Slide 4
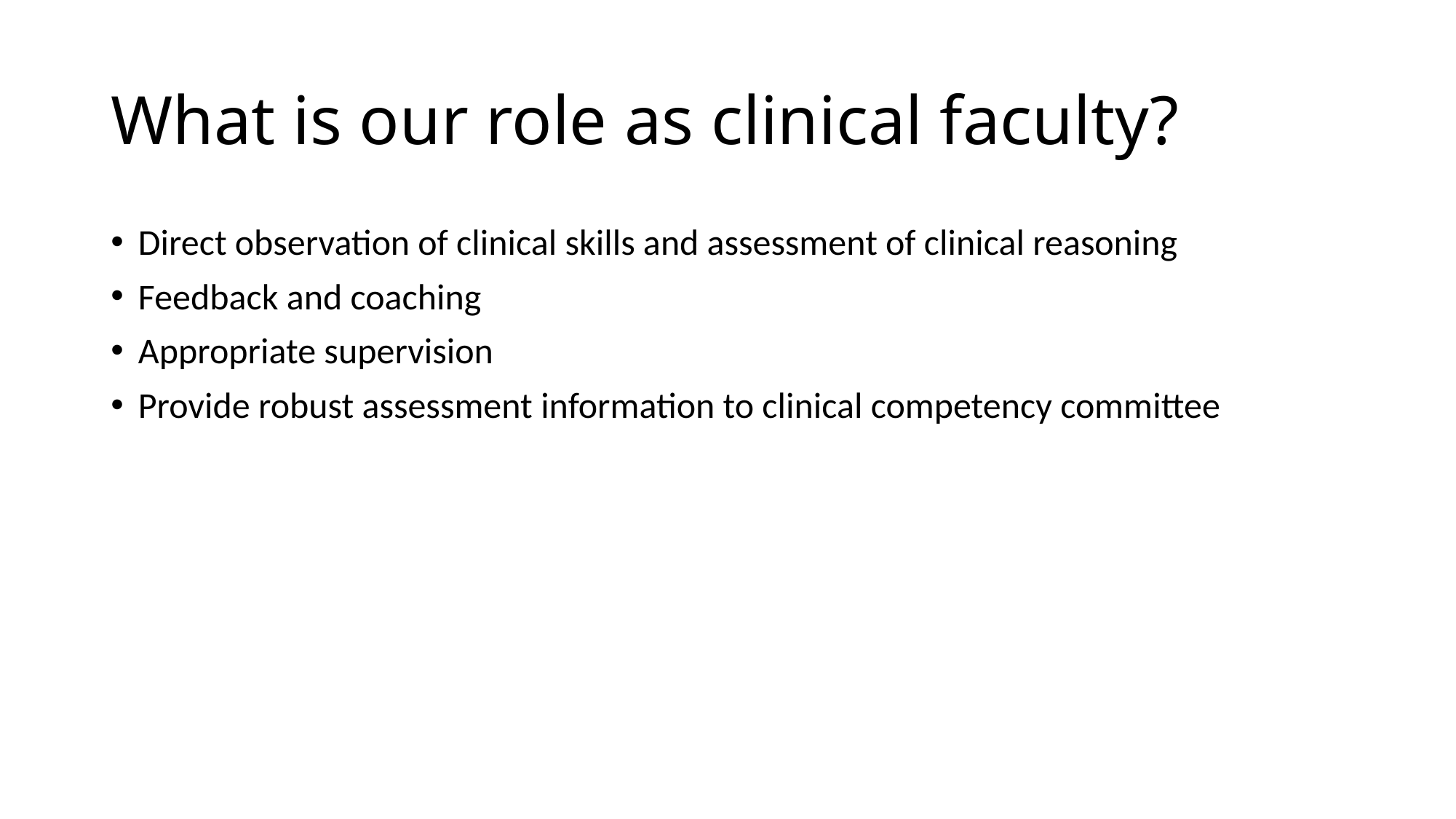

# What is our role as clinical faculty?
Direct observation of clinical skills and assessment of clinical reasoning
Feedback and coaching
Appropriate supervision
Provide robust assessment information to clinical competency committee

## Slide 5
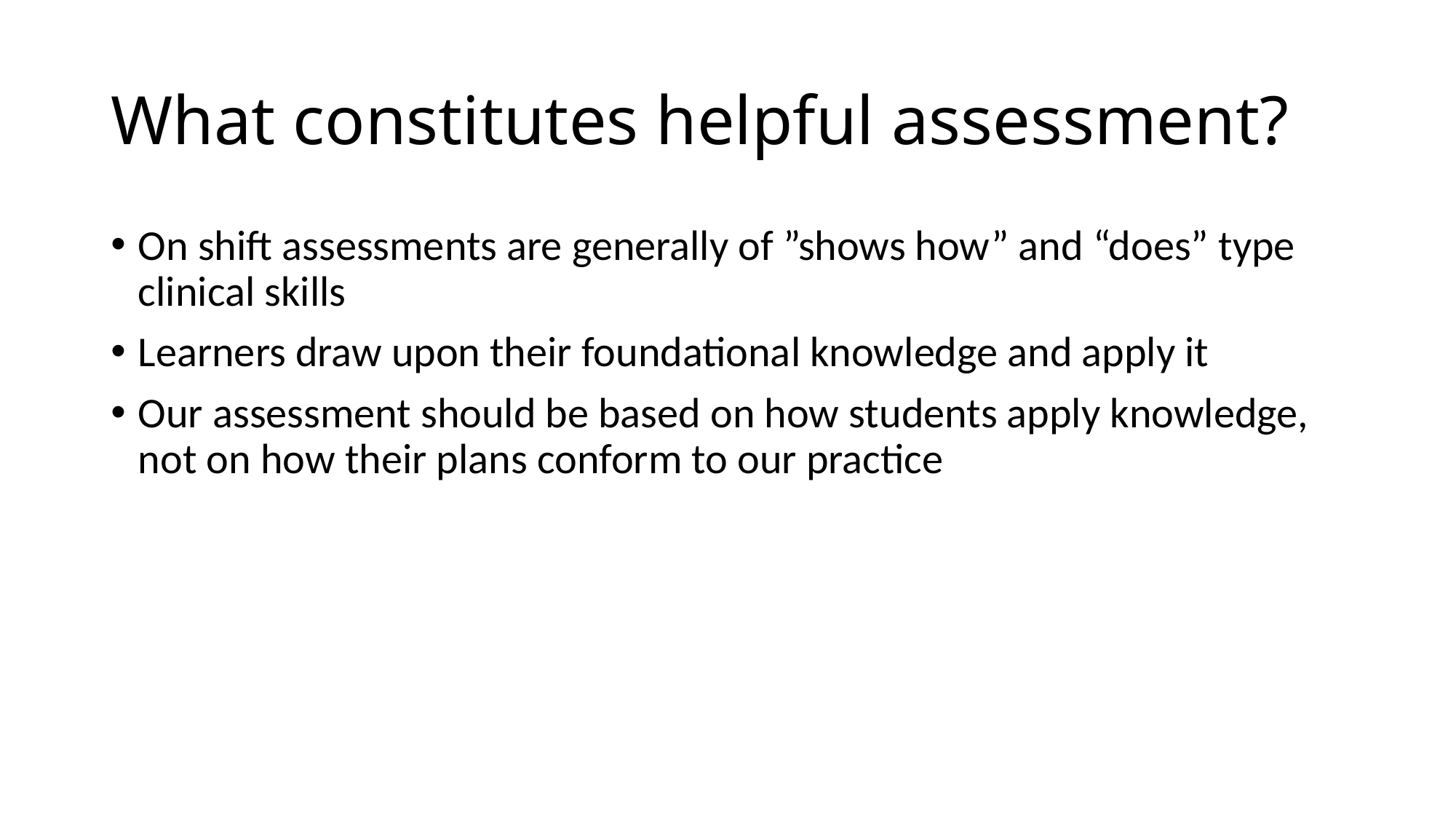

# What constitutes helpful assessment?
On shift assessments are generally of ”shows how” and “does” type clinical skills
Learners draw upon their foundational knowledge and apply it
Our assessment should be based on how students apply knowledge, not on how their plans conform to our practice

## Slide 6
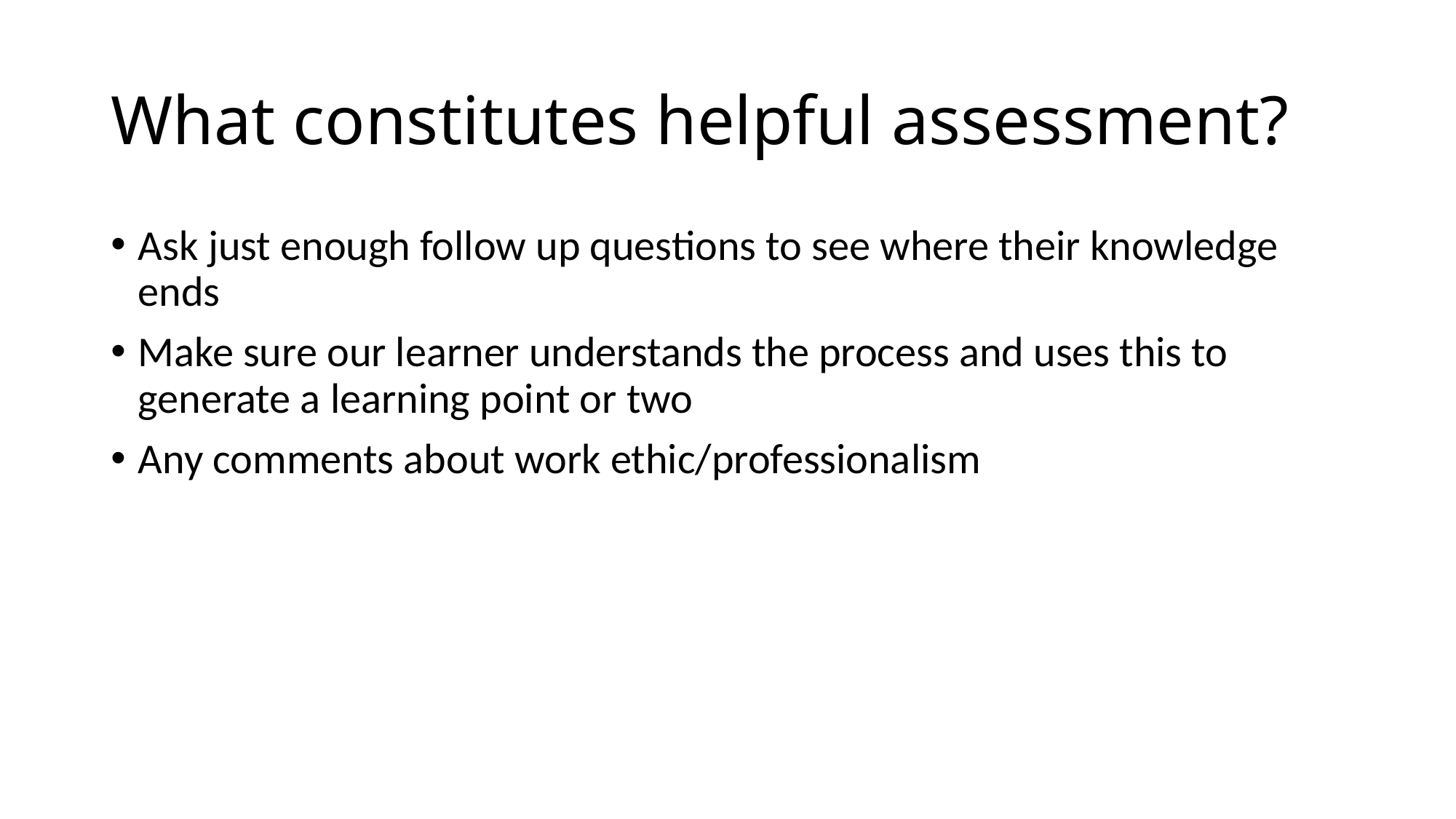

# What constitutes helpful assessment?
Ask just enough follow up questions to see where their knowledge ends
Make sure our learner understands the process and uses this to generate a learning point or two
Any comments about work ethic/professionalism

## Slide 7
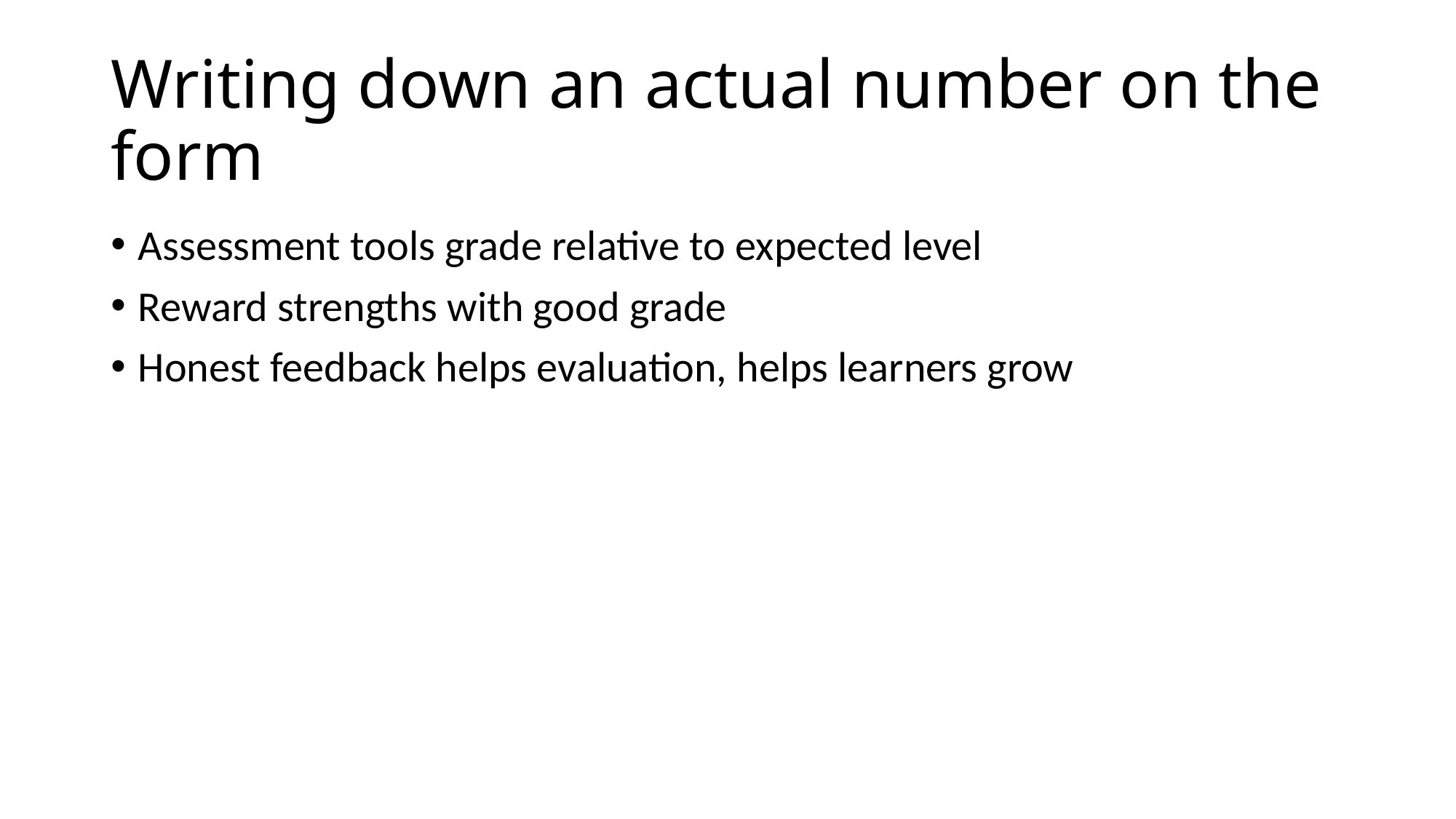

# Writing down an actual number on the form
Assessment tools grade relative to expected level
Reward strengths with good grade
Honest feedback helps evaluation, helps learners grow

## Slide 8
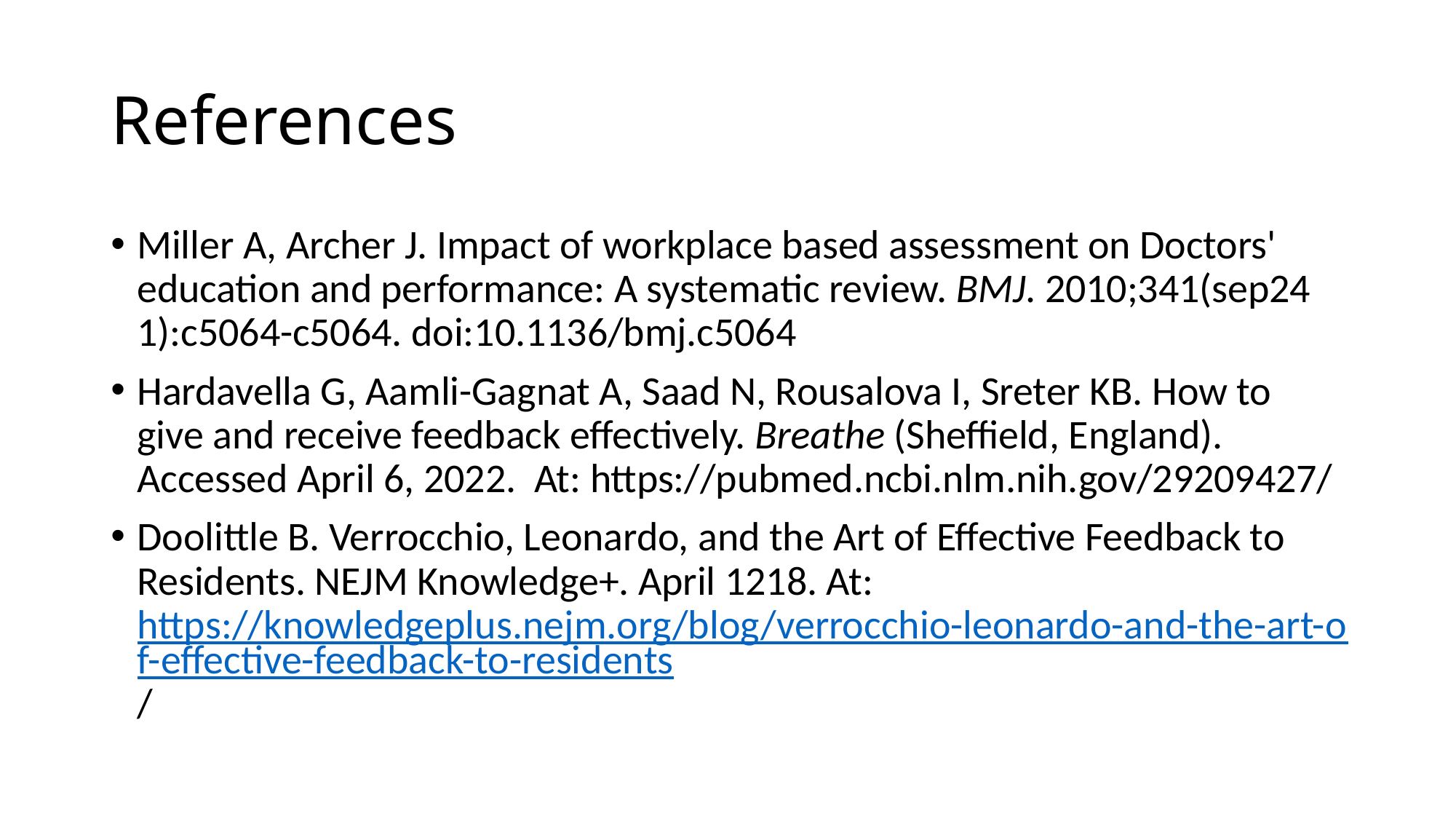

# References
Miller A, Archer J. Impact of workplace based assessment on Doctors' education and performance: A systematic review. BMJ. 2010;341(sep24 1):c5064-c5064. doi:10.1136/bmj.c5064
Hardavella G, Aamli-Gagnat A, Saad N, Rousalova I, Sreter KB. How to give and receive feedback effectively. Breathe (Sheffield, England). Accessed April 6, 2022.  At: https://pubmed.ncbi.nlm.nih.gov/29209427/
Doolittle B. Verrocchio, Leonardo, and the Art of Effective Feedback to Residents. NEJM Knowledge+. April 1218. At: https://knowledgeplus.nejm.org/blog/verrocchio-leonardo-and-the-art-of-effective-feedback-to-residents/
