# Supplementary material for: Residents Are Coming: A Faculty Development Curriculum to Prepare a Community Site For New Learners
Source: J Educ Teach Emerg Med. 2022 Jul 15;7(3):C1–C41. doi: 10.21980/J87D2N (PMC10332697; doi:10.21980/J87D2N)
Supplement: Supplementary file 2 — Please see associated PowerPoint file [file jetem-7-3-c1-appendix4.pptx]

## Slide 1
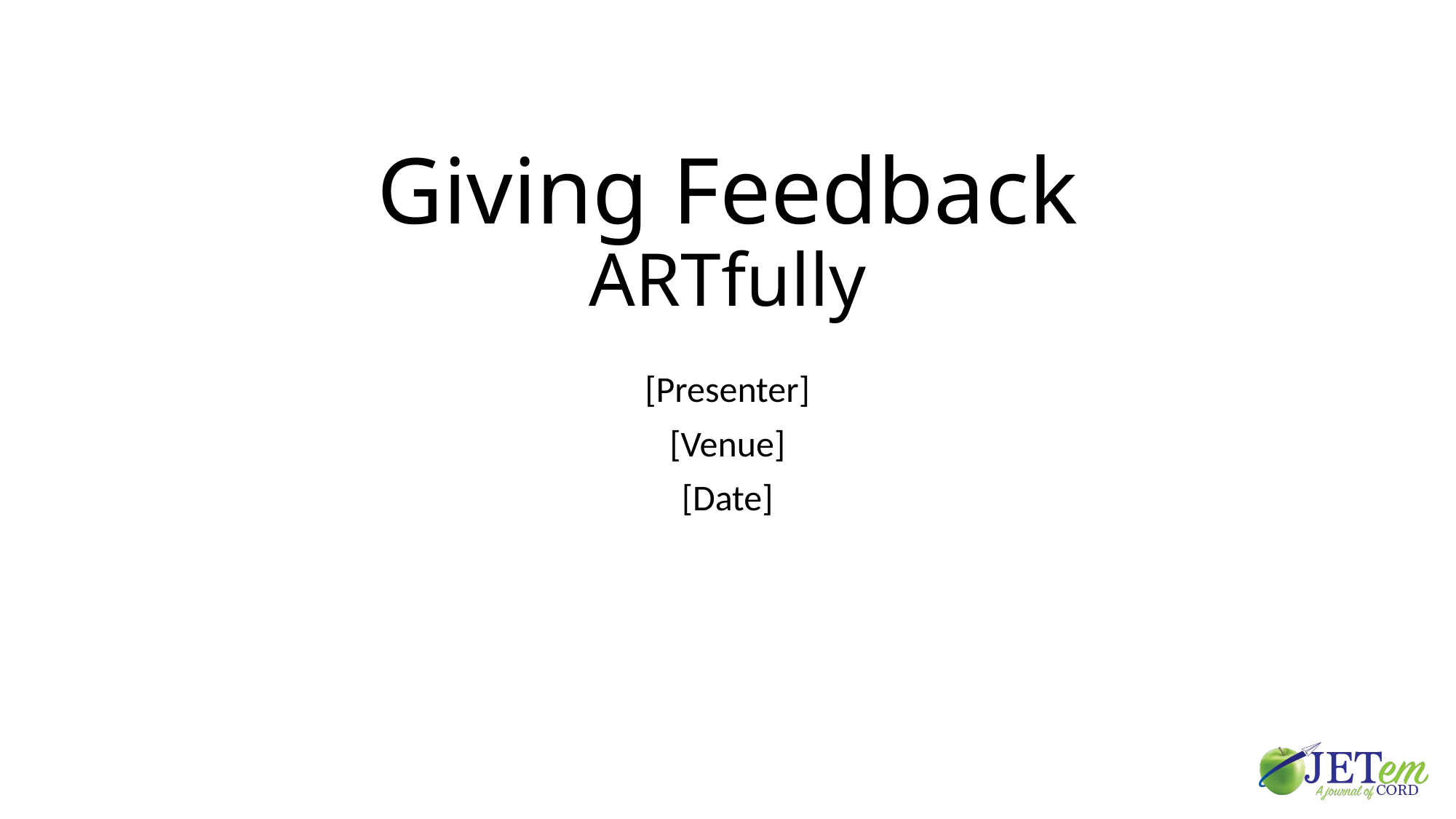

# Giving Feedback ARTfully
[Presenter]
[Venue]
[Date]

## Slide 2
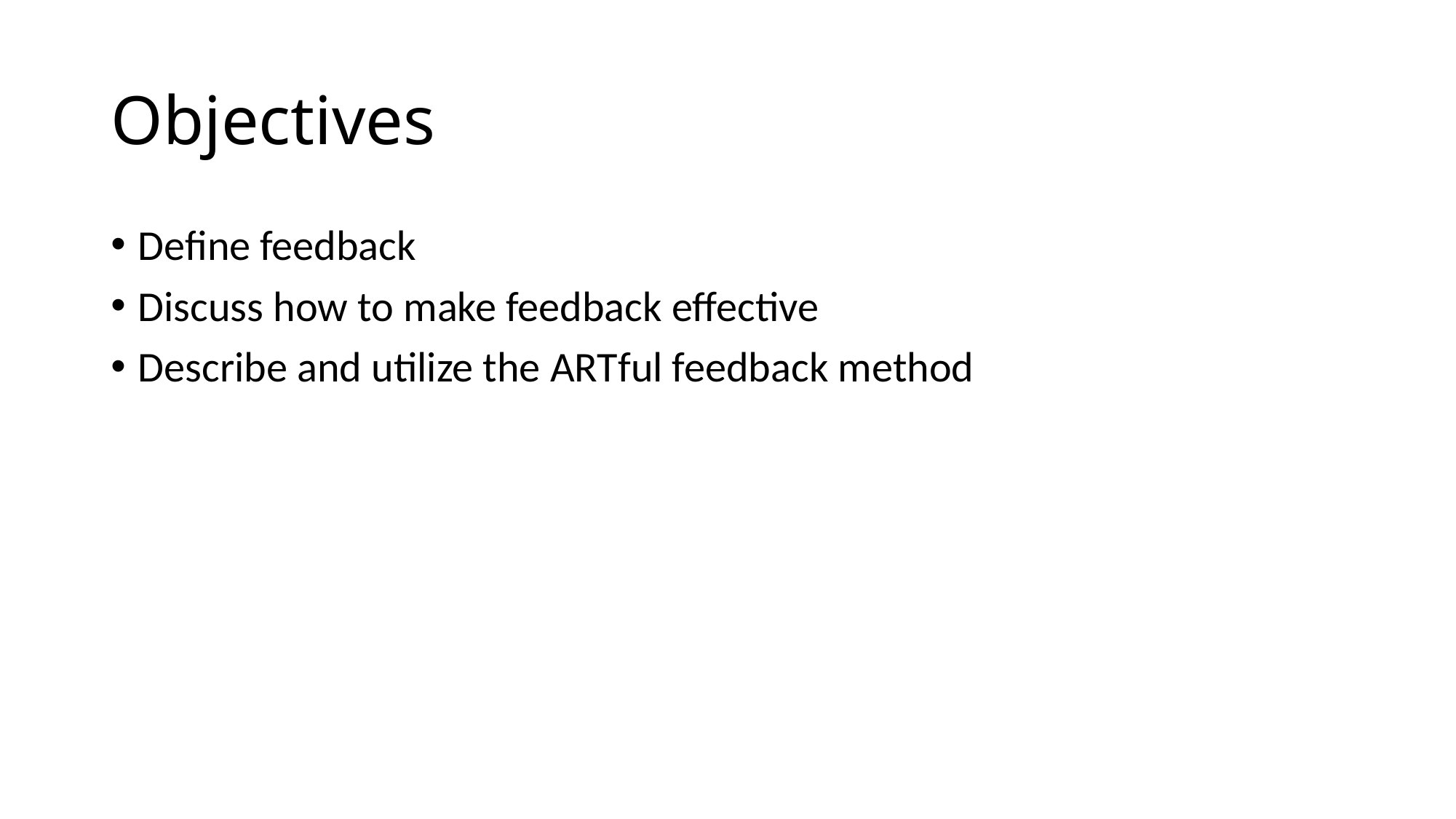

# Objectives
Define feedback
Discuss how to make feedback effective
Describe and utilize the ARTful feedback method

## Slide 3
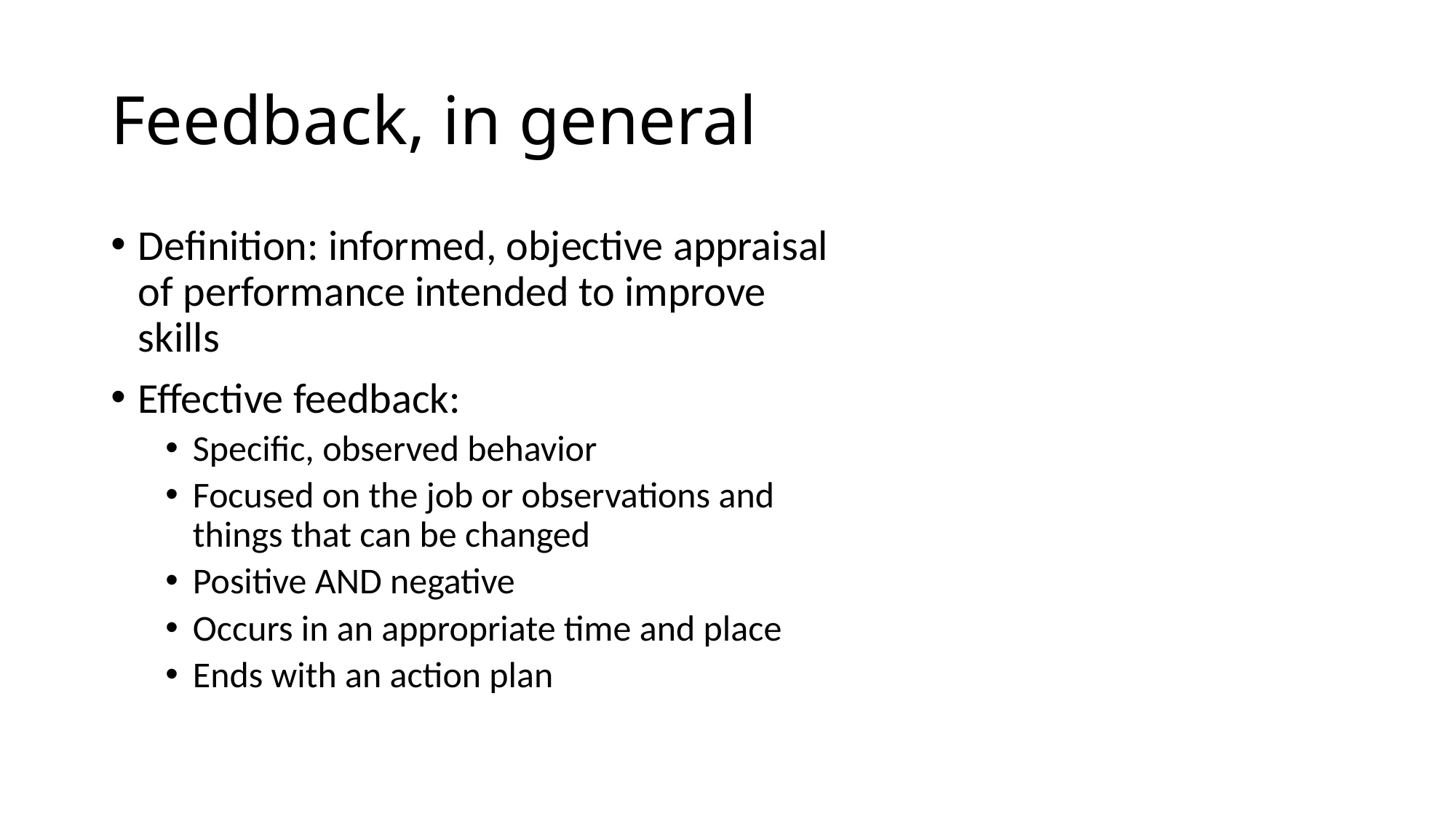

# Feedback, in general
Definition: informed, objective appraisal of performance intended to improve skills
Effective feedback:
Specific, observed behavior
Focused on the job or observations and things that can be changed
Positive AND negative
Occurs in an appropriate time and place
Ends with an action plan

## Slide 4
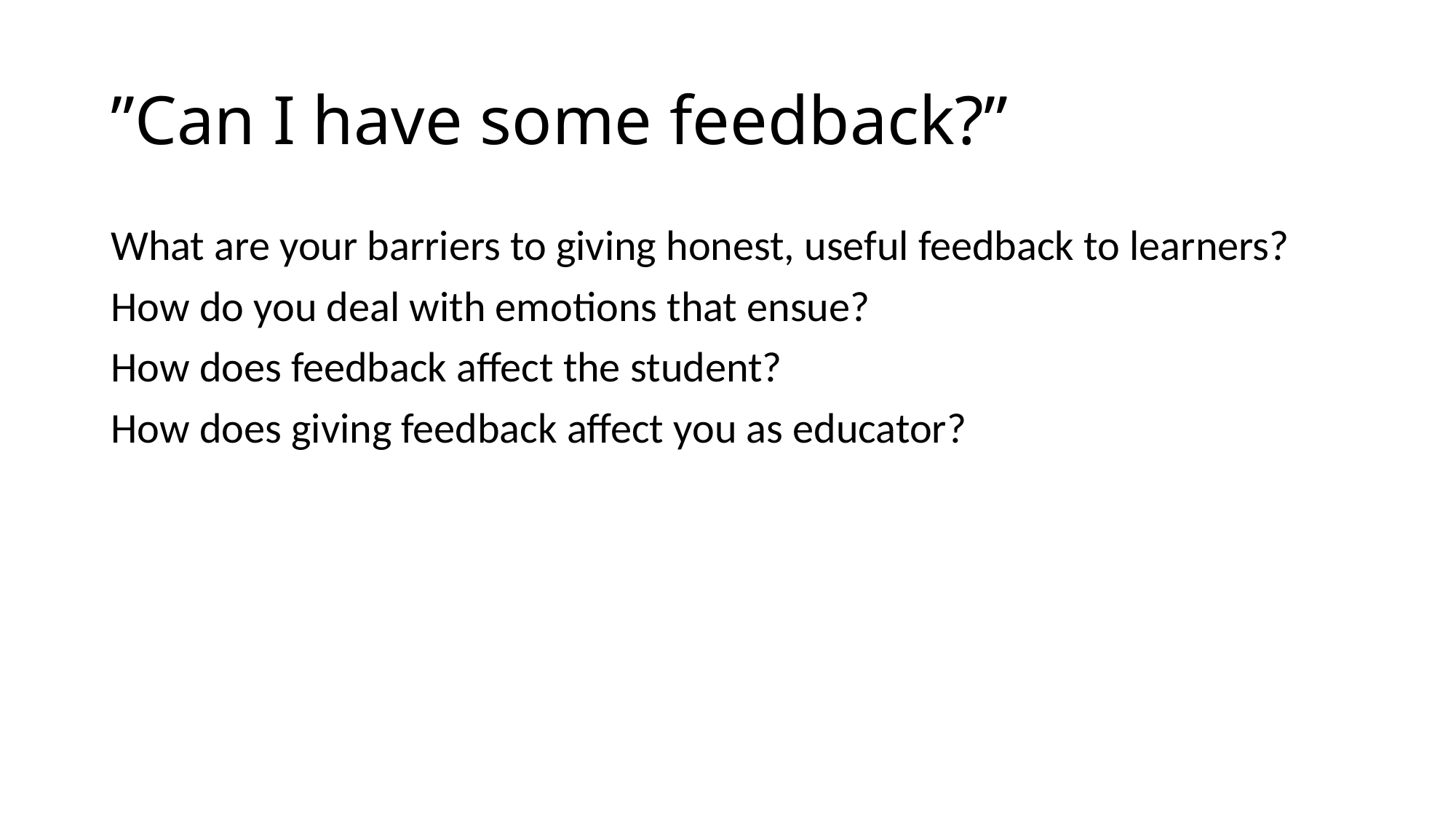

# ”Can I have some feedback?”
What are your barriers to giving honest, useful feedback to learners?
How do you deal with emotions that ensue?
How does feedback affect the student?
How does giving feedback affect you as educator?

## Slide 5
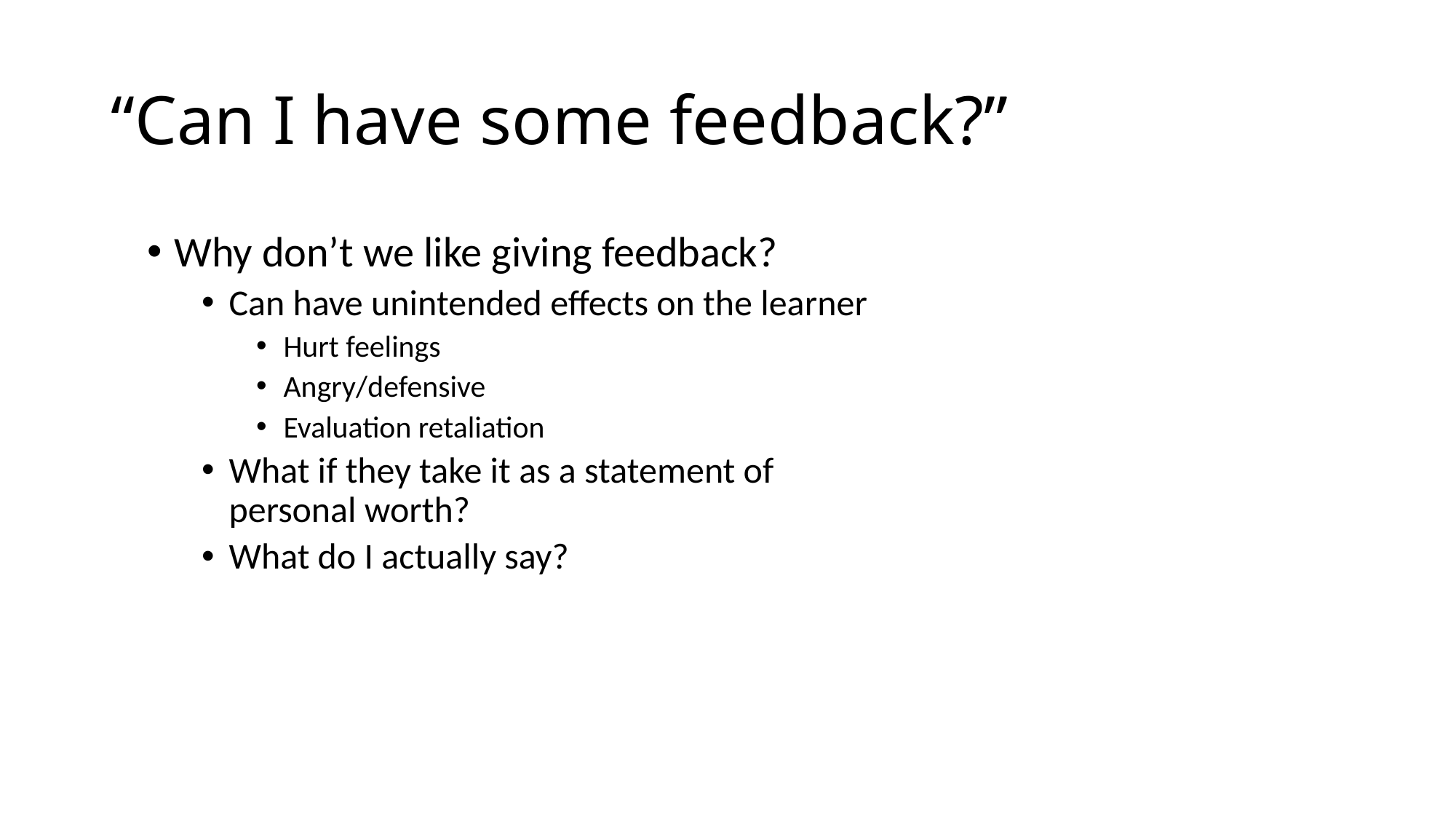

# “Can I have some feedback?”
Why don’t we like giving feedback?
Can have unintended effects on the learner
Hurt feelings
Angry/defensive
Evaluation retaliation
What if they take it as a statement of personal worth?
What do I actually say?

## Slide 6
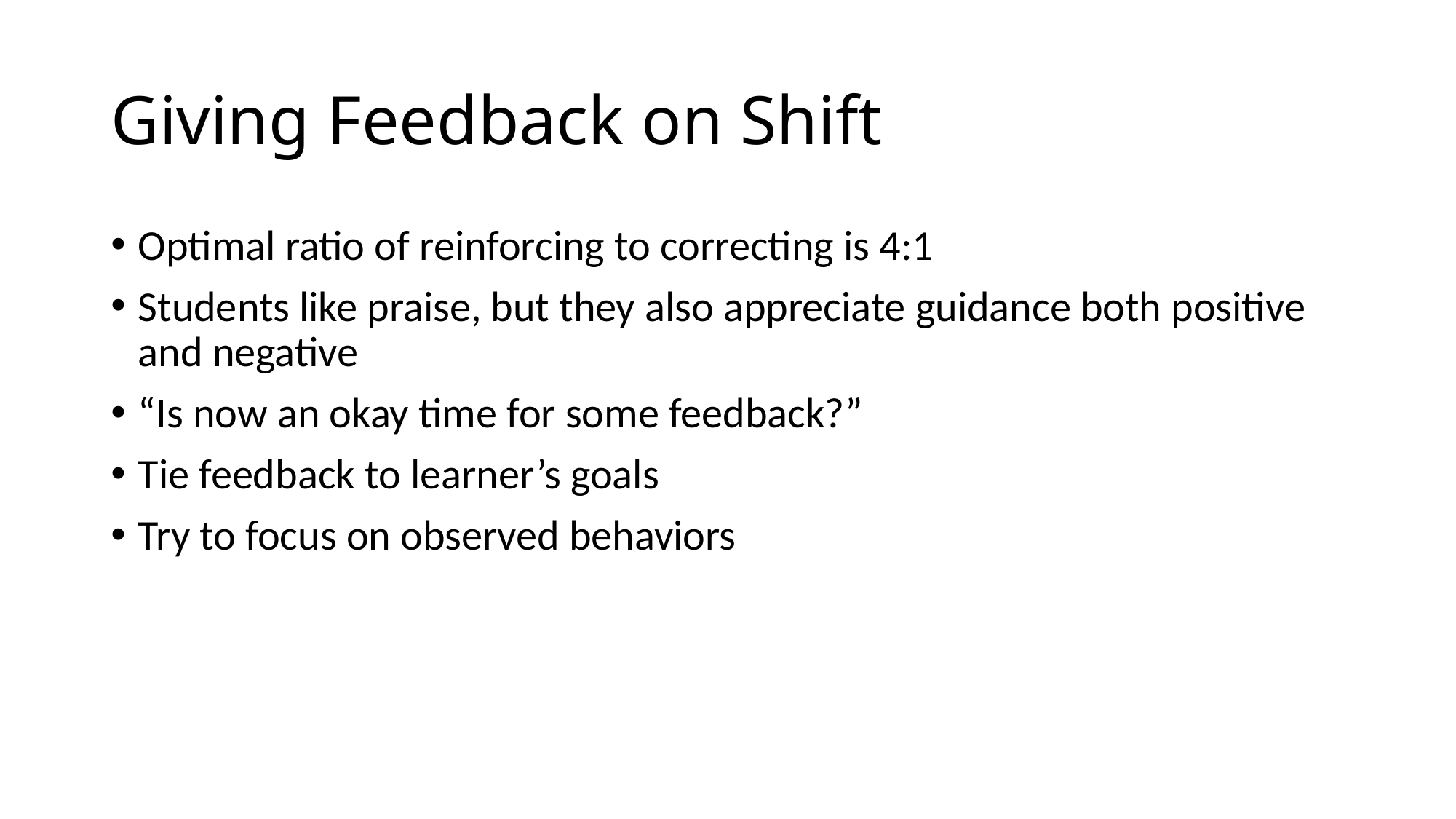

# Giving Feedback on Shift
Optimal ratio of reinforcing to correcting is 4:1
Students like praise, but they also appreciate guidance both positive and negative
“Is now an okay time for some feedback?”
Tie feedback to learner’s goals
Try to focus on observed behaviors

## Slide 7
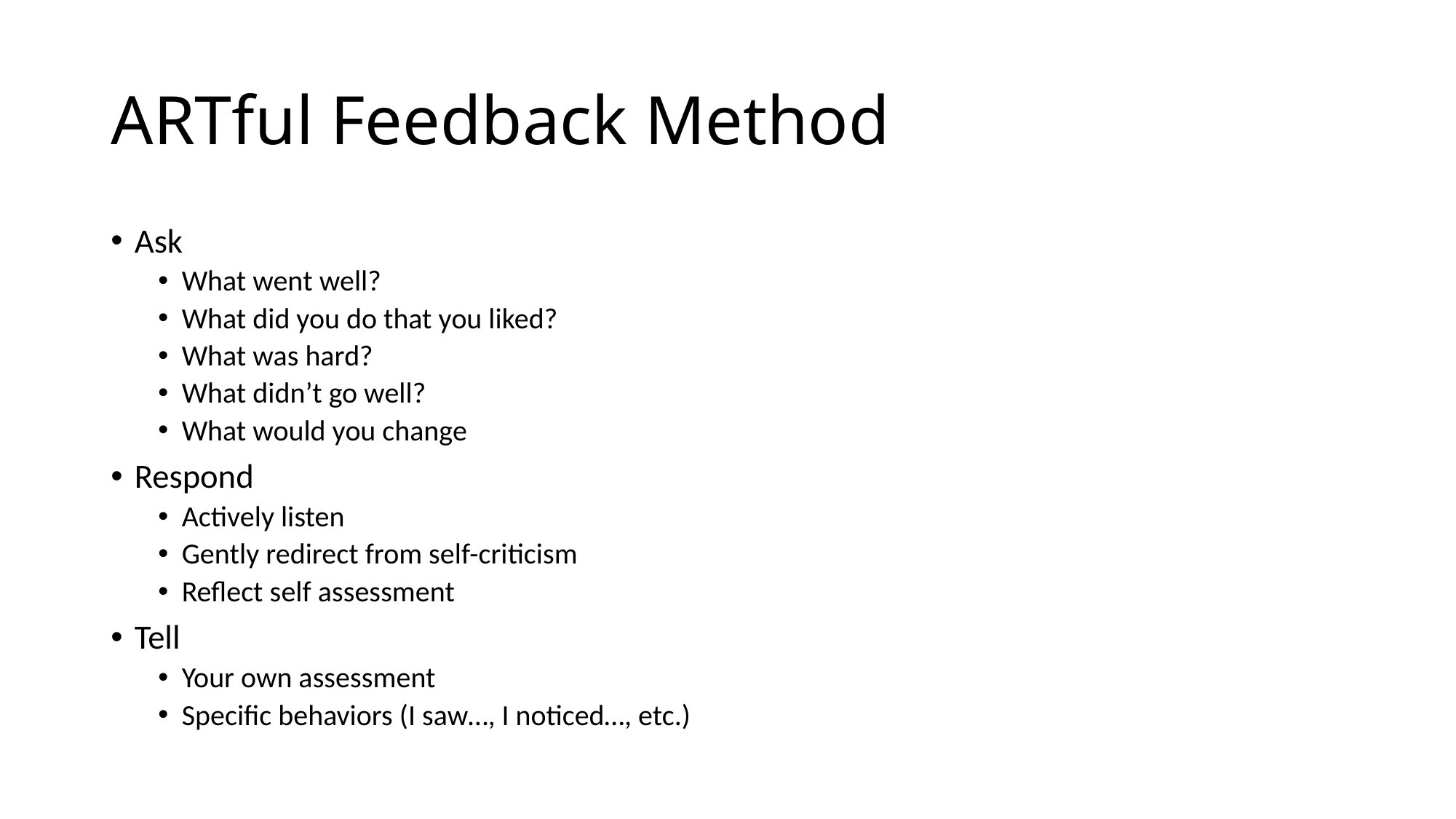

# ARTful Feedback Method
Ask
What went well?
What did you do that you liked?
What was hard?
What didn’t go well?
What would you change
Respond
Actively listen
Gently redirect from self-criticism
Reflect self assessment
Tell
Your own assessment
Specific behaviors (I saw…, I noticed…, etc.)

## Slide 8
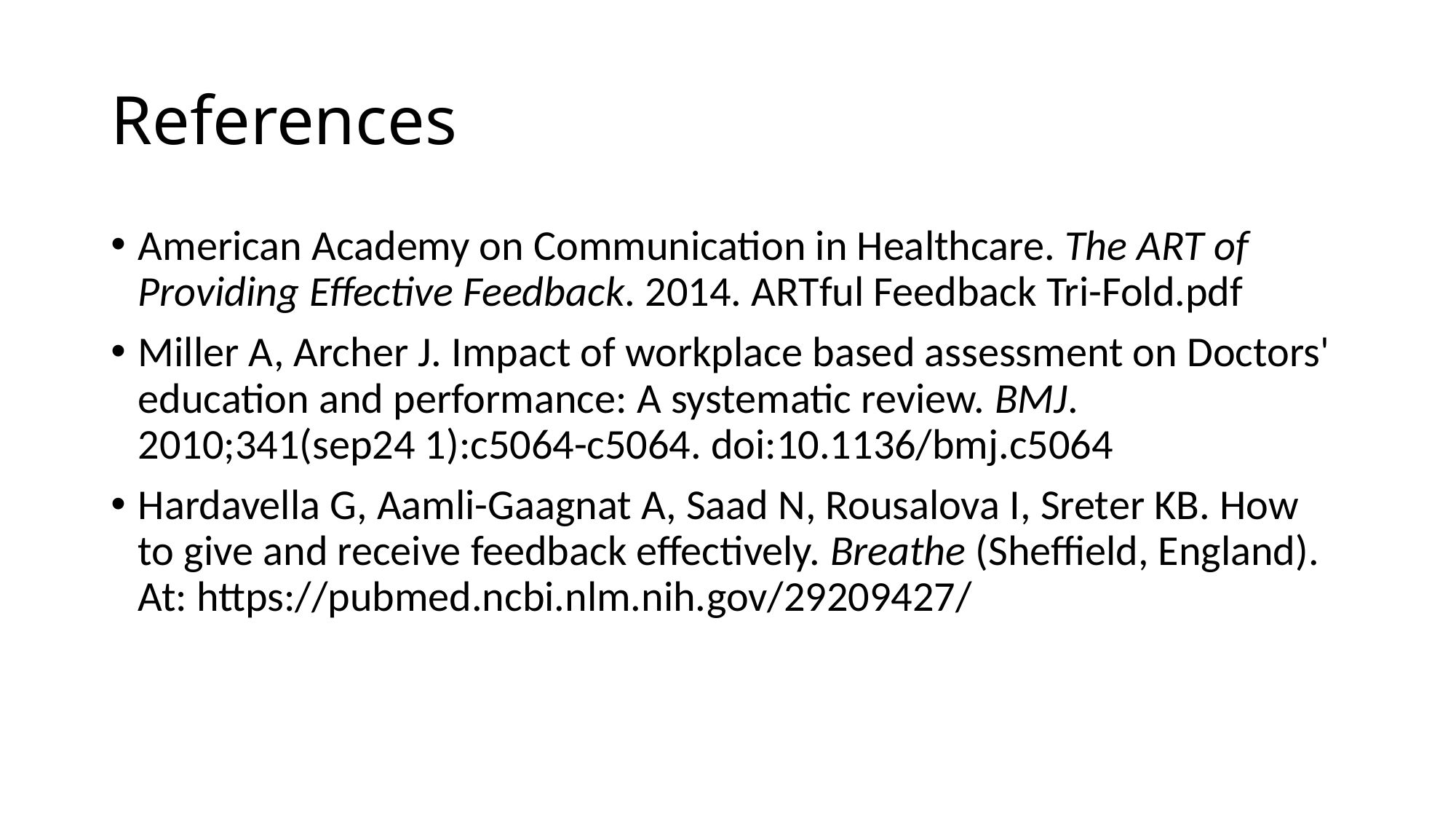

# References
American Academy on Communication in Healthcare. The ART of Providing Effective Feedback. 2014. ARTful Feedback Tri-Fold.pdf
Miller A, Archer J. Impact of workplace based assessment on Doctors' education and performance: A systematic review. BMJ. 2010;341(sep24 1):c5064-c5064. doi:10.1136/bmj.c5064
Hardavella G, Aamli-Gaagnat A, Saad N, Rousalova I, Sreter KB. How to give and receive feedback effectively. Breathe (Sheffield, England). At: https://pubmed.ncbi.nlm.nih.gov/29209427/
