# Supplementary material for: Residents Are Coming: A Faculty Development Curriculum to Prepare a Community Site For New Learners
Source: J Educ Teach Emerg Med. 2022 Jul 15;7(3):C1–C41. doi: 10.21980/J87D2N (PMC10332697; doi:10.21980/J87D2N)
Supplement: Supplementary file 3 — Please see associated PowerPoint file [file jetem-7-3-c1-appendix5.pptx]

## Slide 1
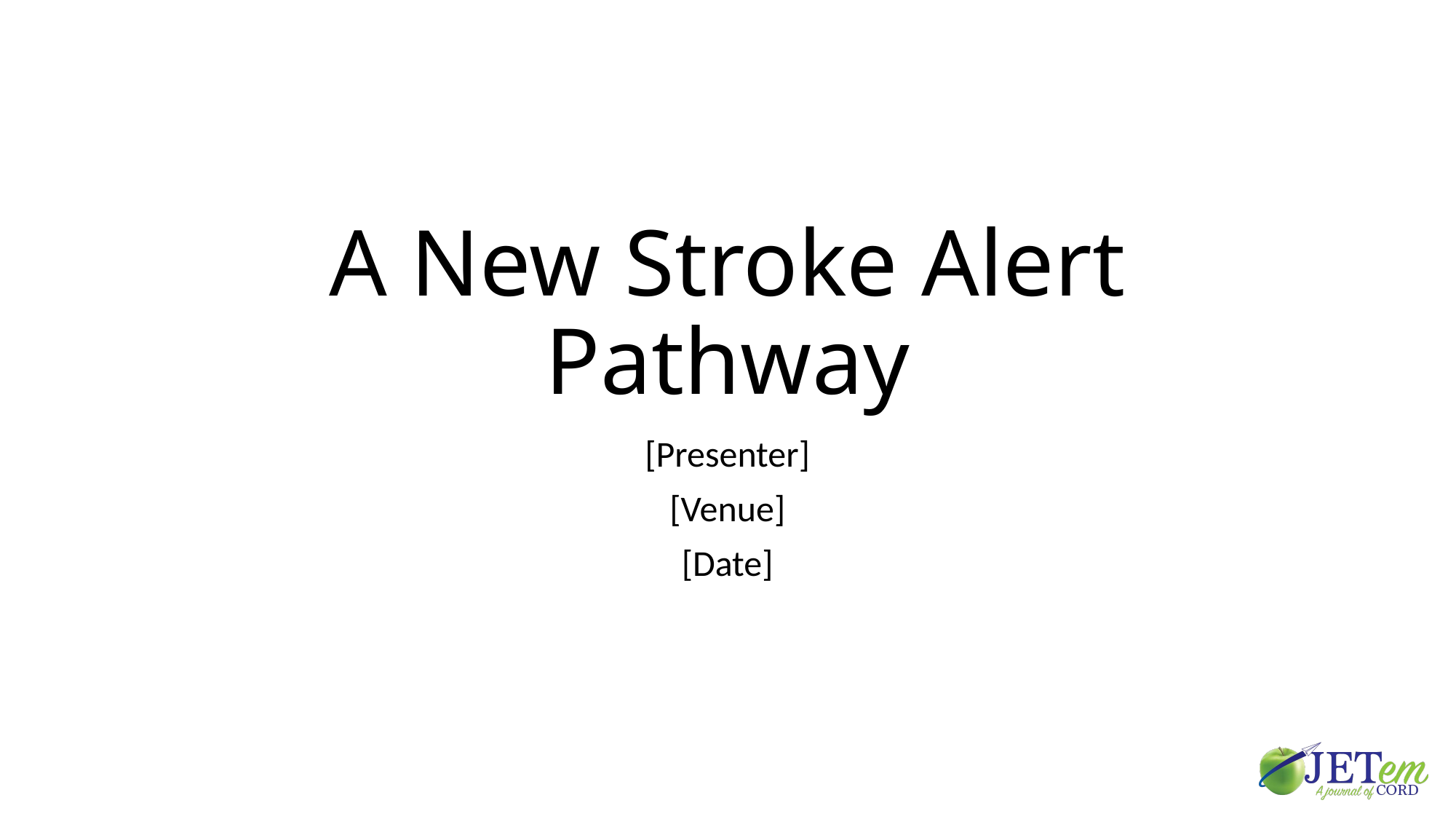

# A New Stroke Alert Pathway
[Presenter]
[Venue]
[Date]

## Slide 2
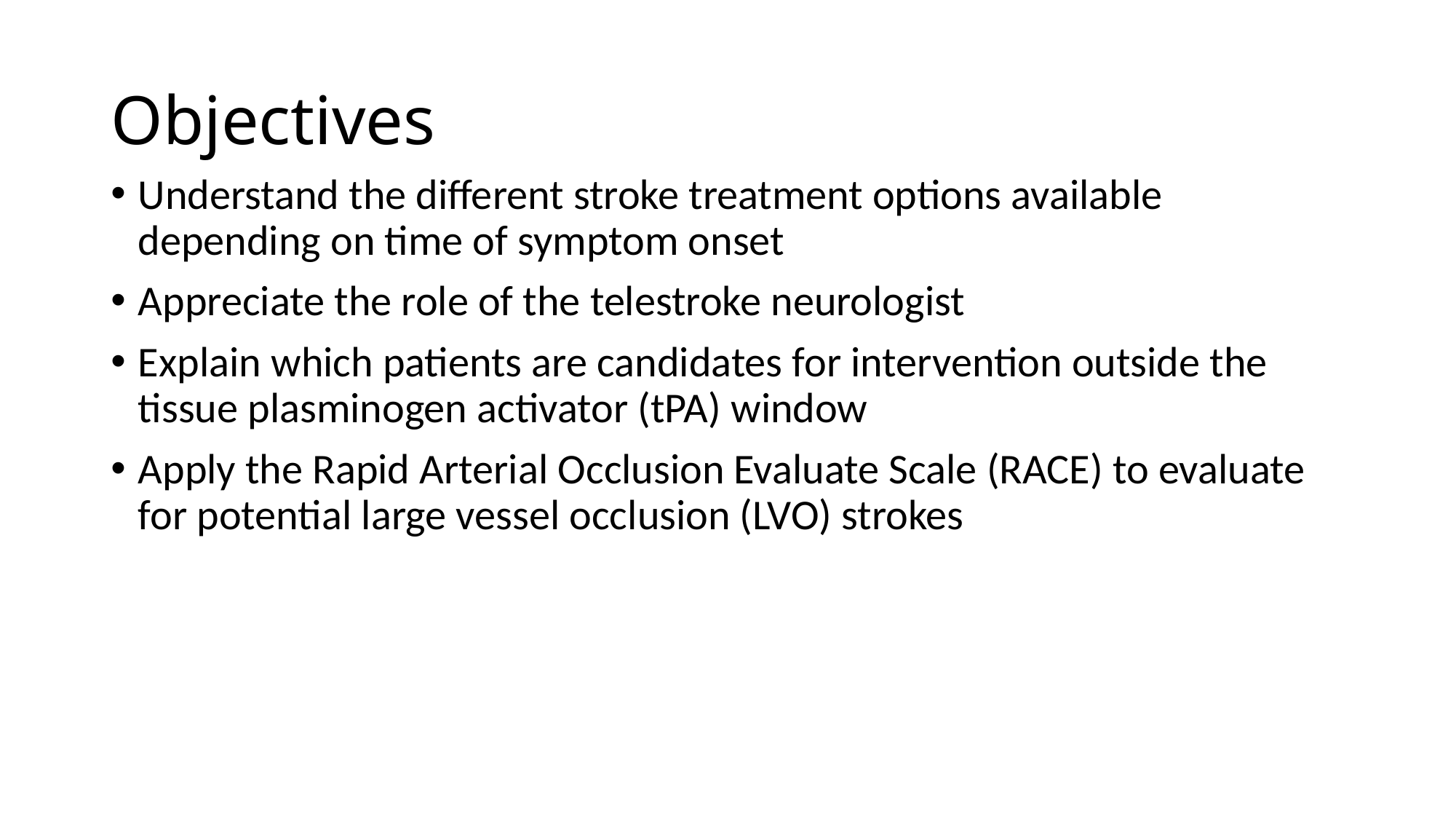

# Objectives
Understand the different stroke treatment options available depending on time of symptom onset
Appreciate the role of the telestroke neurologist
Explain which patients are candidates for intervention outside the tissue plasminogen activator (tPA) window
Apply the Rapid Arterial Occlusion Evaluate Scale (RACE) to evaluate for potential large vessel occlusion (LVO) strokes

## Slide 3
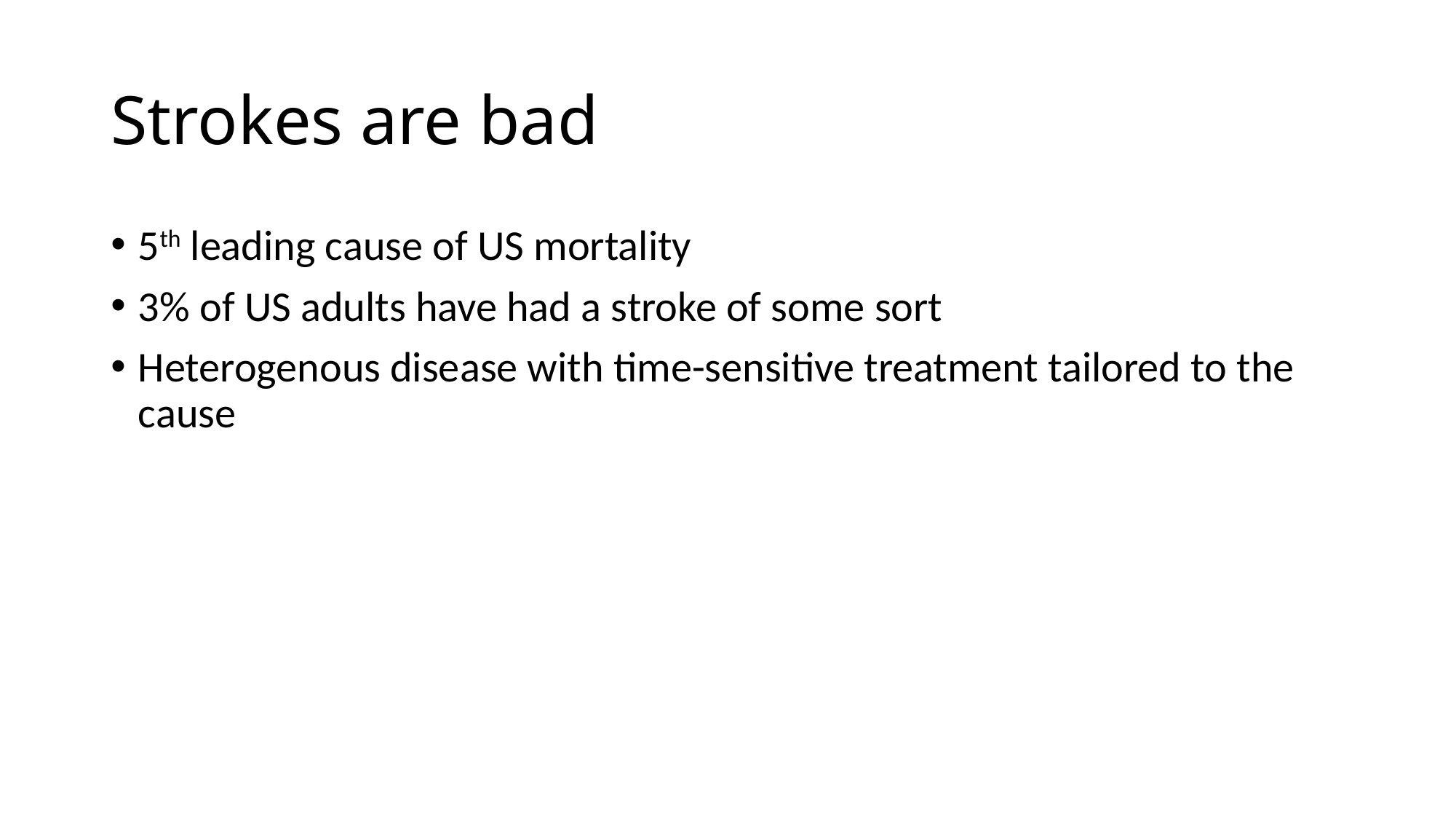

# Strokes are bad
5th leading cause of US mortality
3% of US adults have had a stroke of some sort
Heterogenous disease with time-sensitive treatment tailored to the cause

## Slide 4
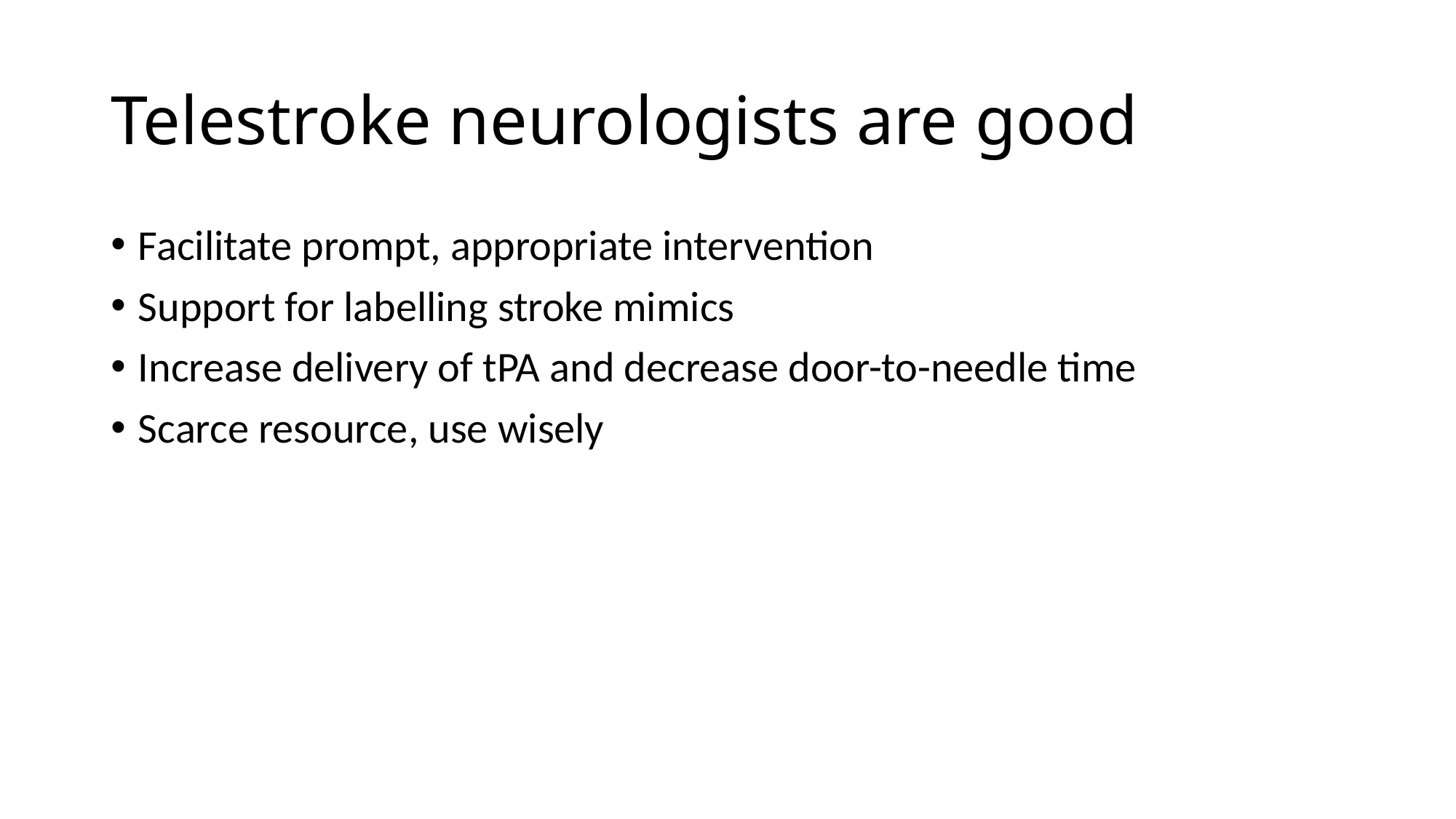

# Telestroke neurologists are good
Facilitate prompt, appropriate intervention
Support for labelling stroke mimics
Increase delivery of tPA and decrease door-to-needle time
Scarce resource, use wisely

## Slide 5
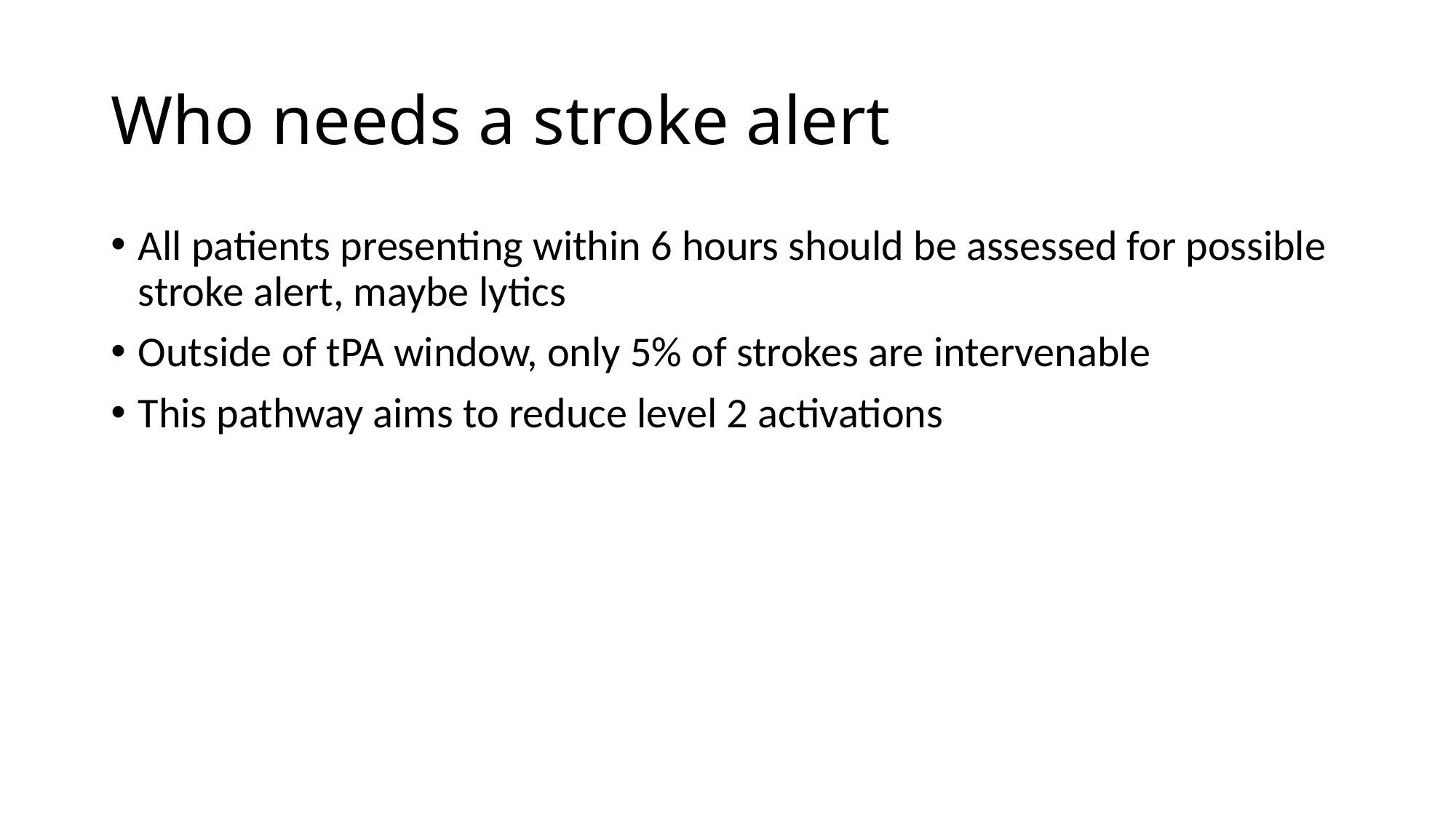

# Who needs a stroke alert
All patients presenting within 6 hours should be assessed for possible stroke alert, maybe lytics
Outside of tPA window, only 5% of strokes are intervenable
This pathway aims to reduce level 2 activations

## Slide 6
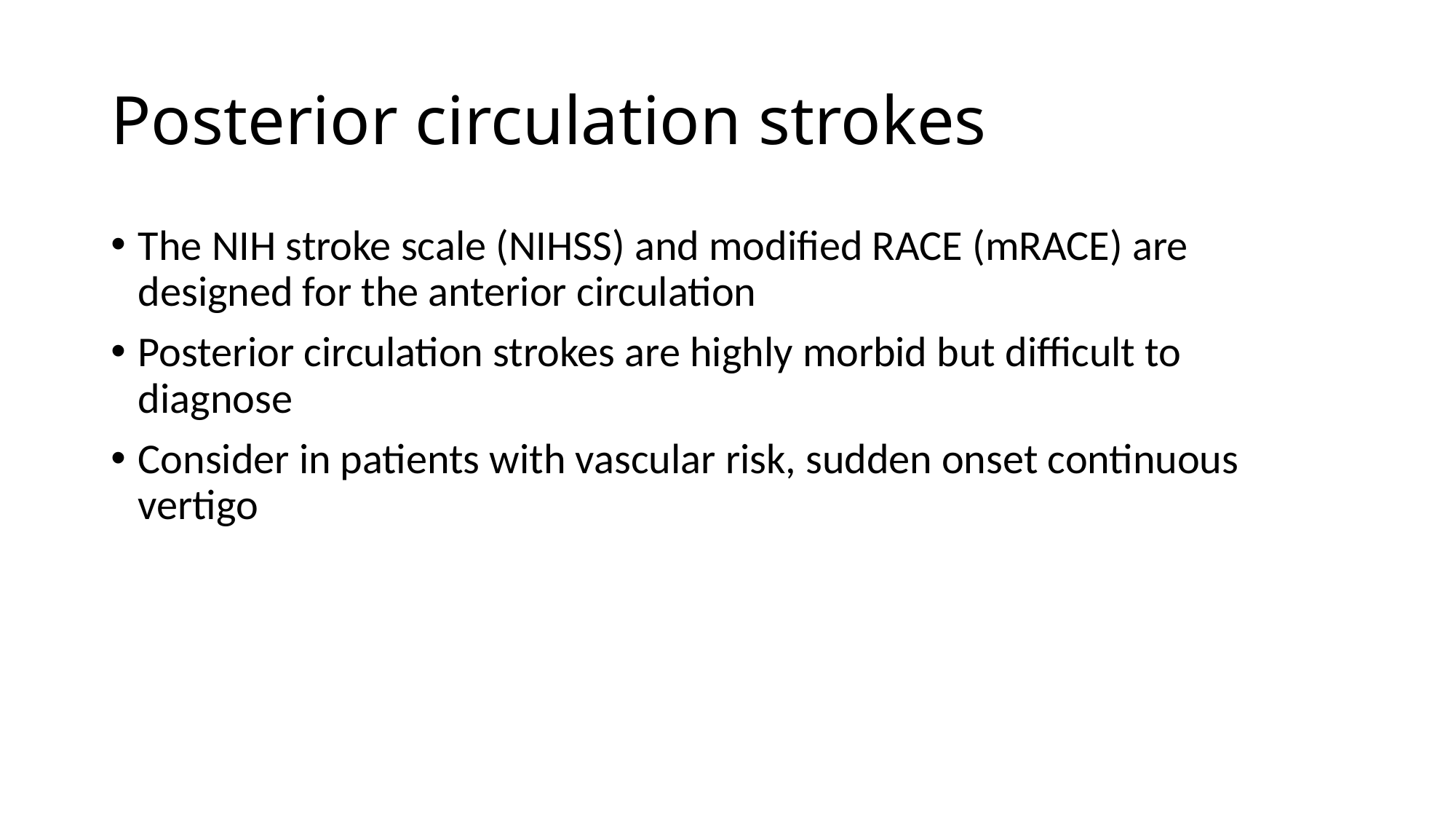

# Posterior circulation strokes
The NIH stroke scale (NIHSS) and modified RACE (mRACE) are designed for the anterior circulation
Posterior circulation strokes are highly morbid but difficult to diagnose
Consider in patients with vascular risk, sudden onset continuous vertigo

## Slide 7
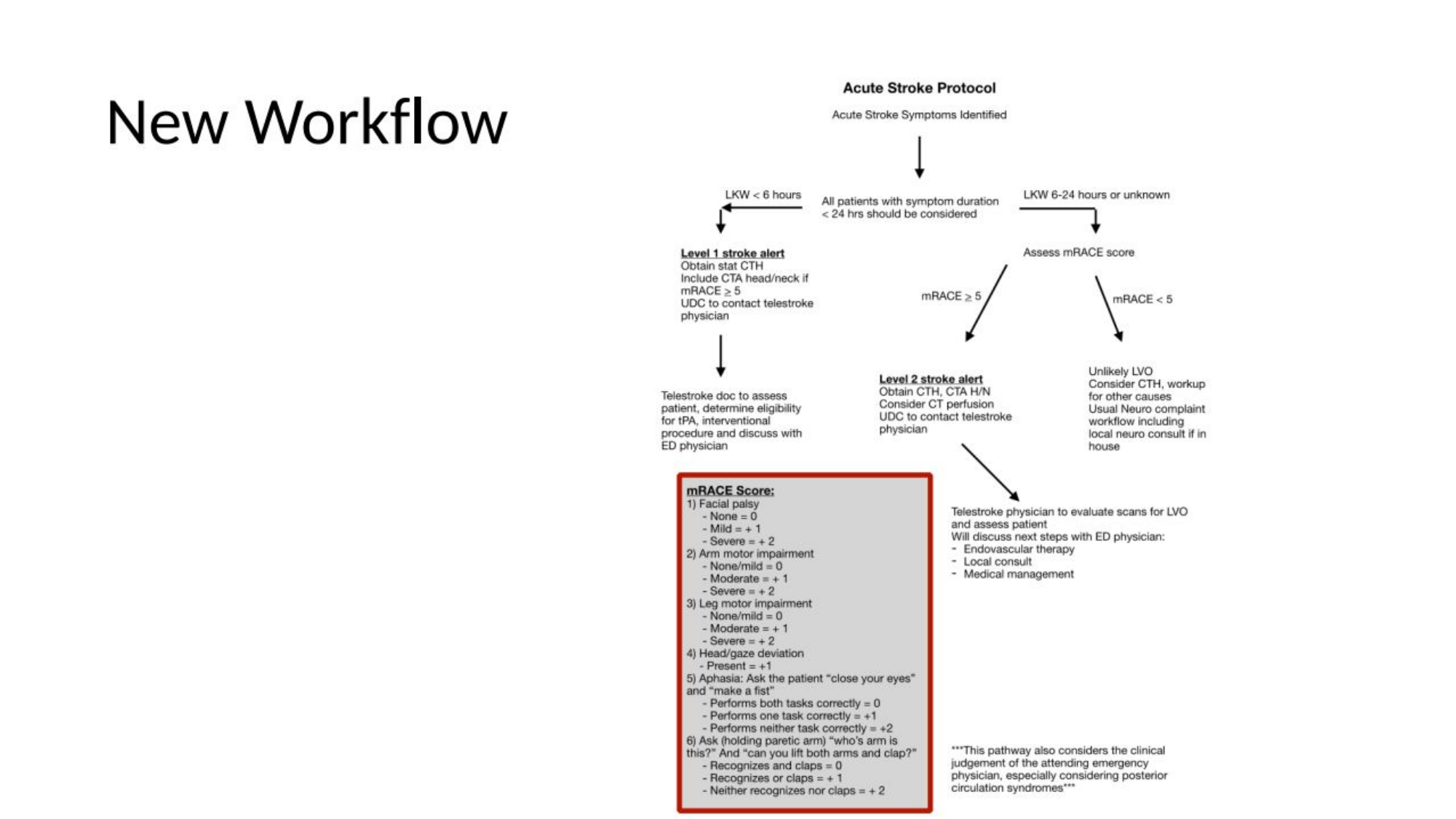

#

## Slide 8
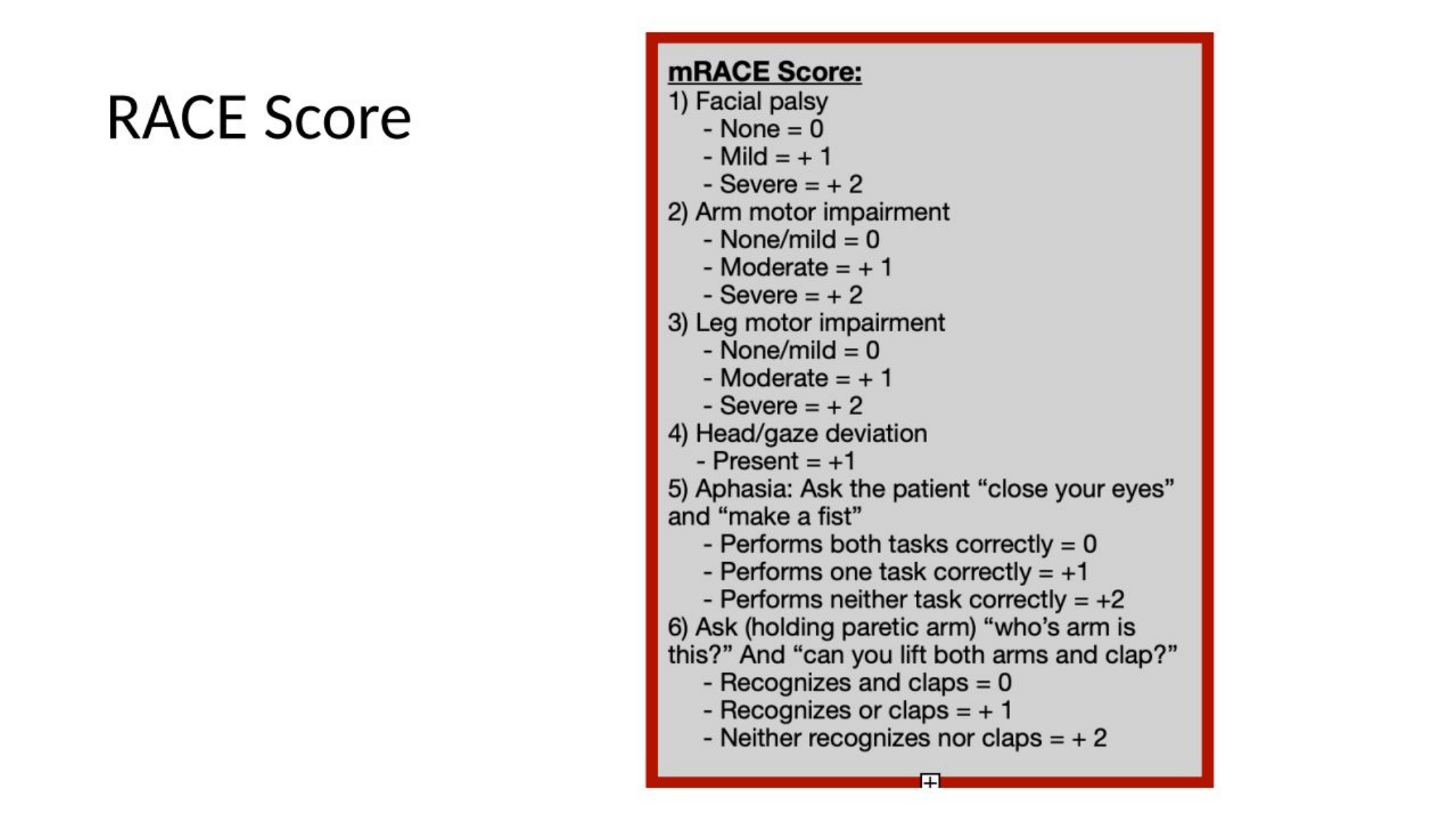

#
