# Supplementary material for: Residents Are Coming: A Faculty Development Curriculum to Prepare a Community Site For New Learners
Source: J Educ Teach Emerg Med. 2022 Jul 15;7(3):C1–C41. doi: 10.21980/J87D2N (PMC10332697; doi:10.21980/J87D2N)
Supplement: Supplementary file 4 — Please see associated PowerPoint file [file jetem-7-3-c1-appendix6.pptx]

## Slide 1
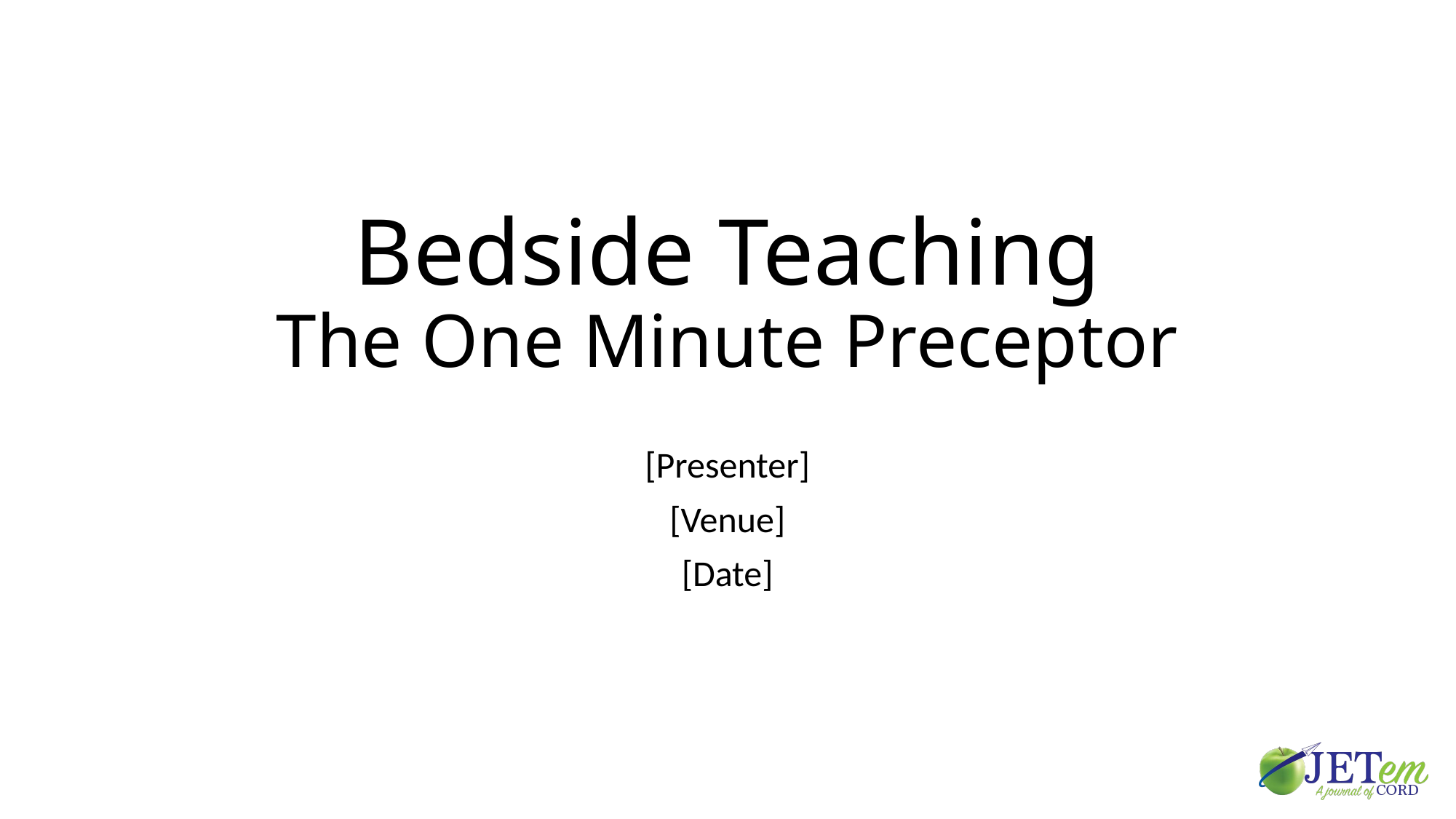

# Bedside Teaching The One Minute Preceptor
[Presenter]
[Venue]
[Date]

## Slide 2
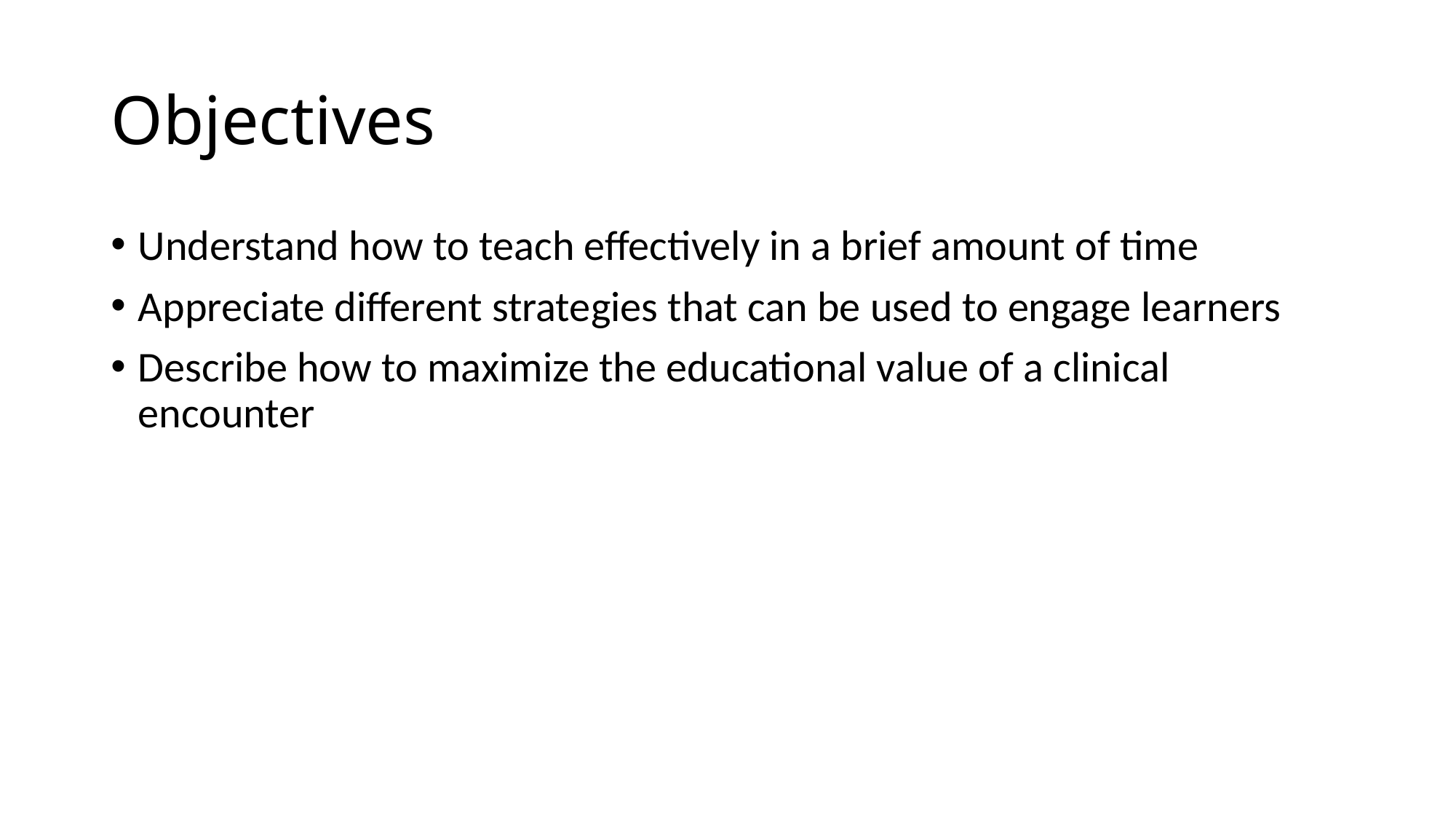

# Objectives
Understand how to teach effectively in a brief amount of time
Appreciate different strategies that can be used to engage learners
Describe how to maximize the educational value of a clinical encounter

## Slide 3
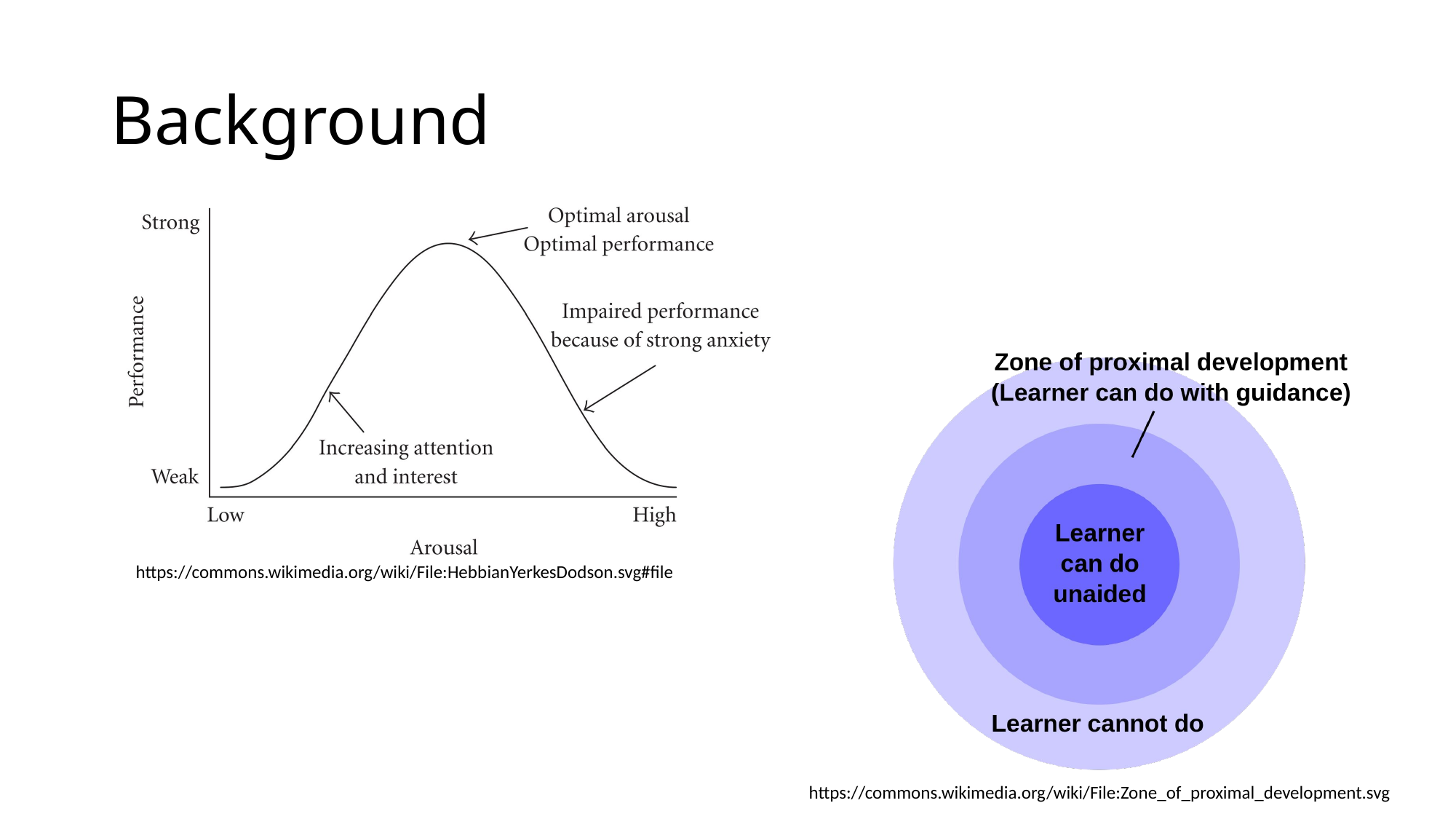

# Background
https://commons.wikimedia.org/wiki/File:HebbianYerkesDodson.svg#file
https://commons.wikimedia.org/wiki/File:Zone_of_proximal_development.svg

## Slide 4
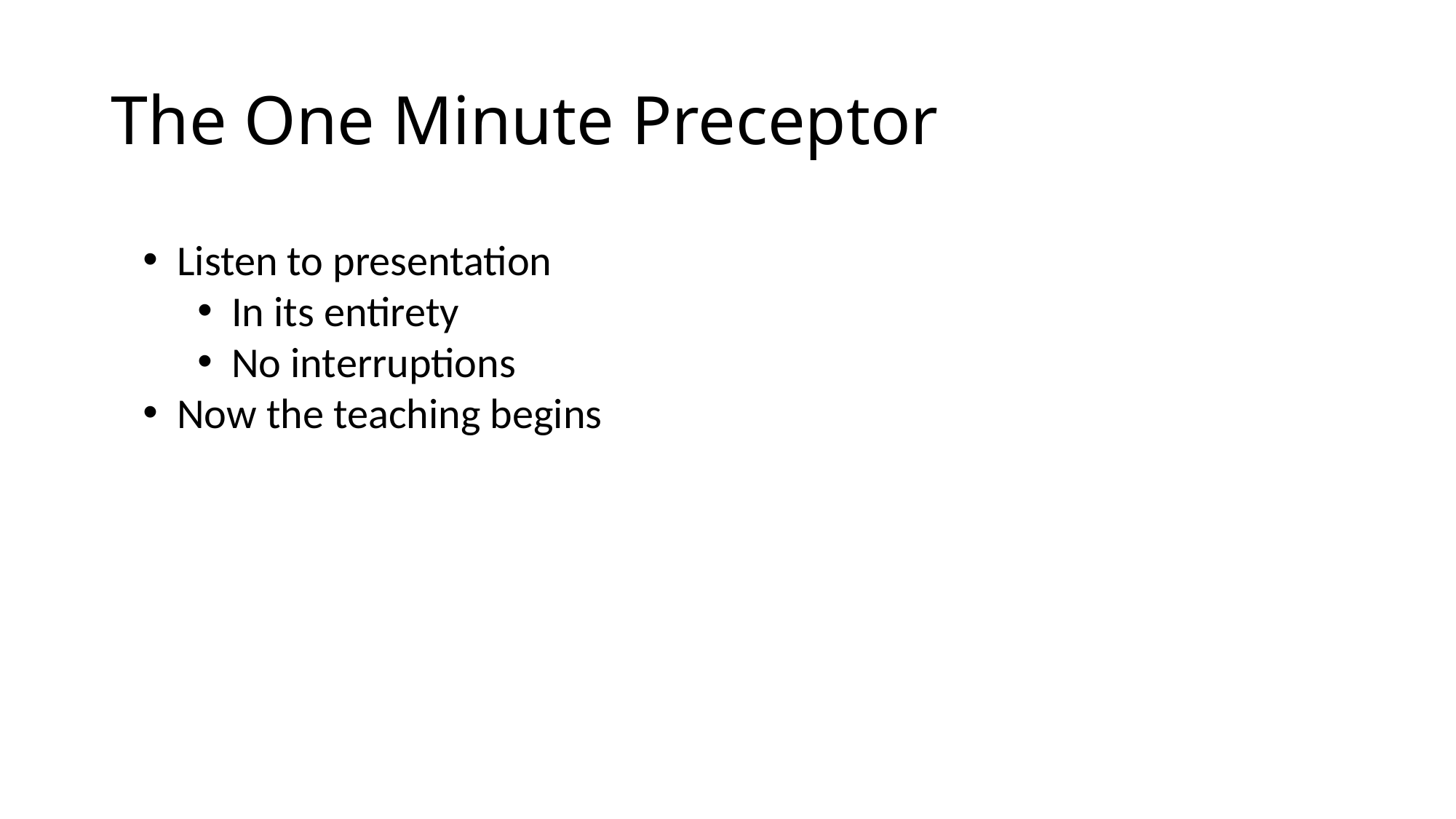

# The One Minute Preceptor
Listen to presentation
In its entirety
No interruptions
Now the teaching begins

## Slide 5
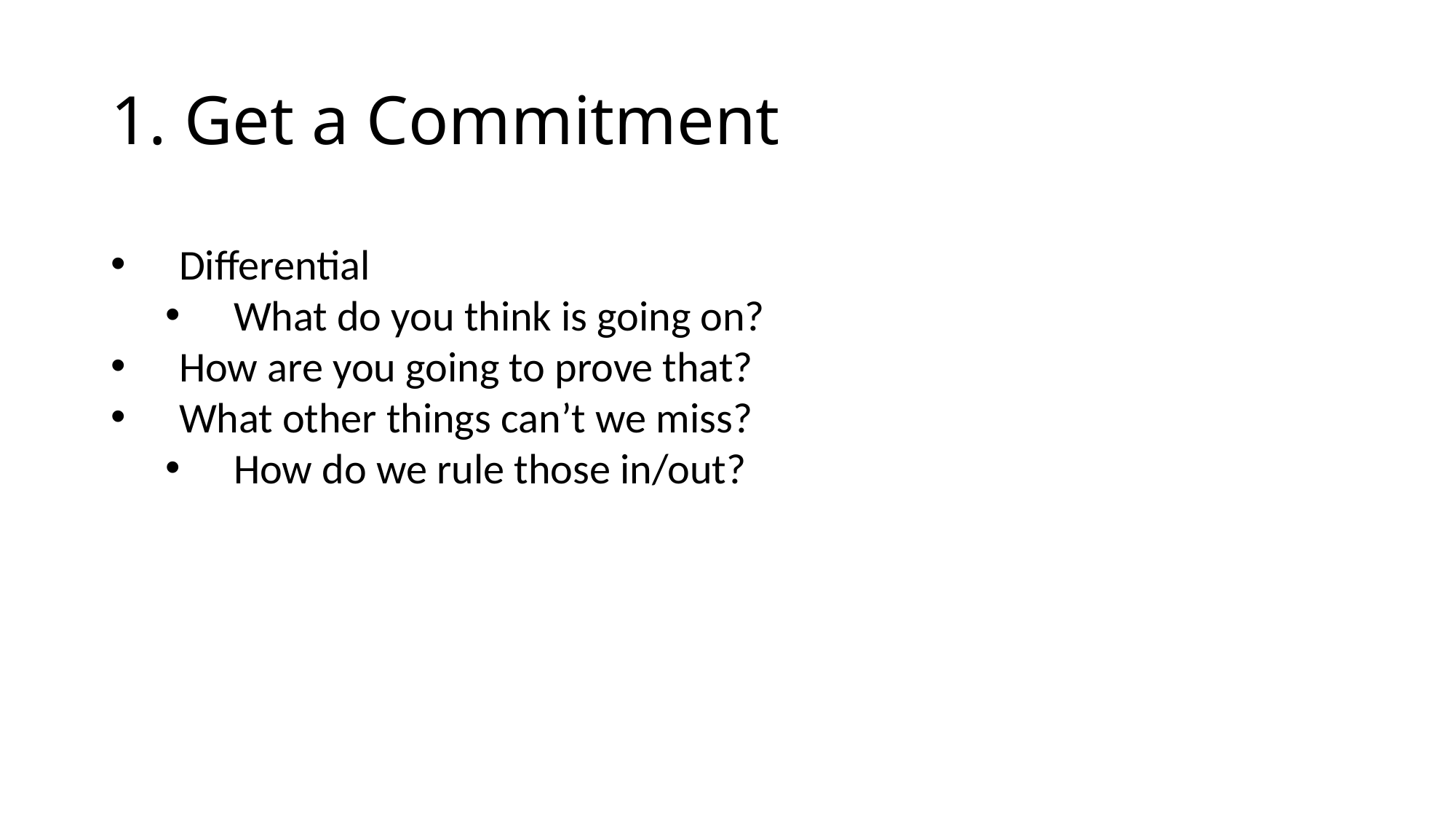

# 1. Get a Commitment
Differential
What do you think is going on?
How are you going to prove that?
What other things can’t we miss?
How do we rule those in/out?

## Slide 6
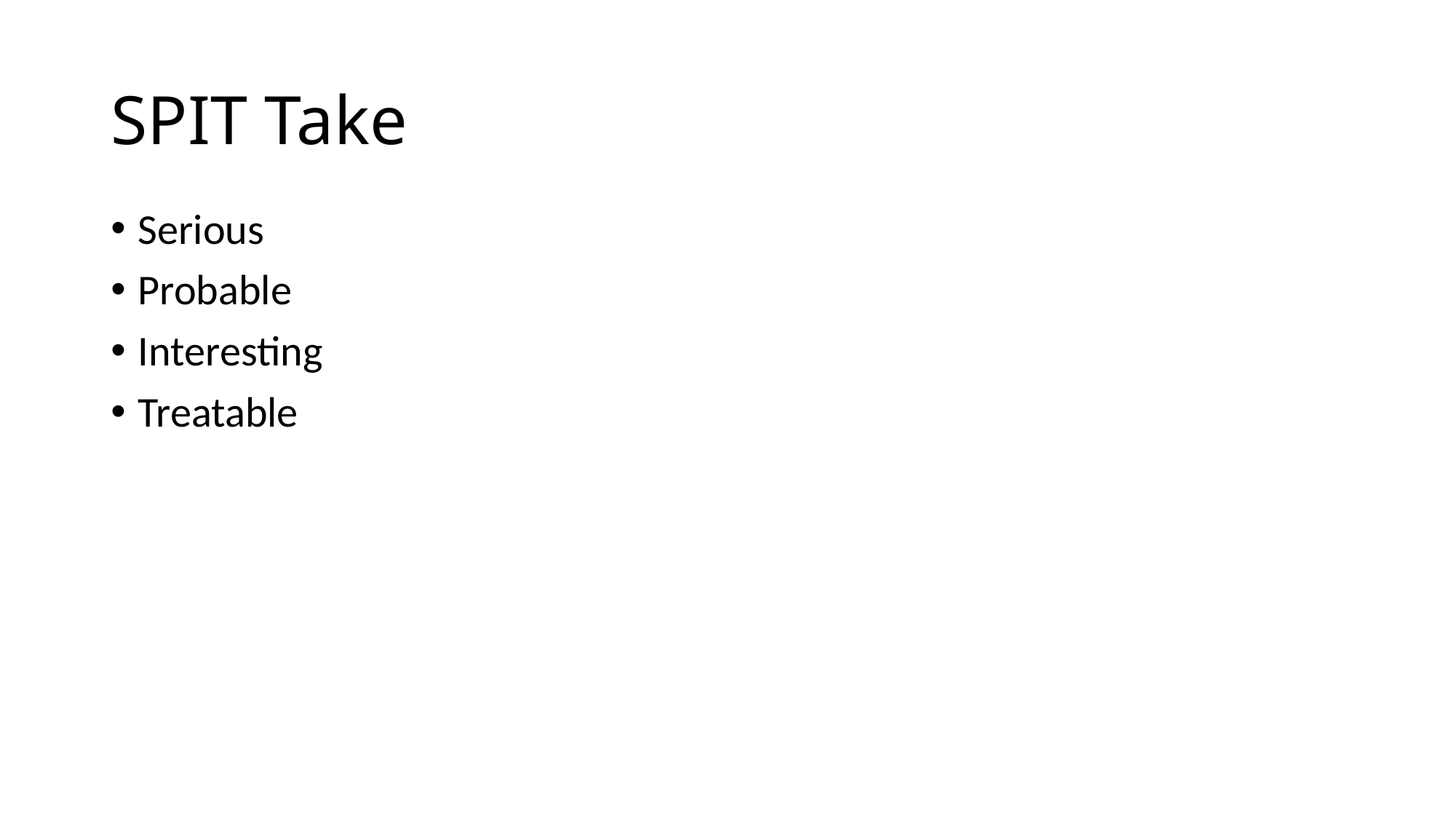

# SPIT Take
Serious
Probable
Interesting
Treatable

## Slide 7
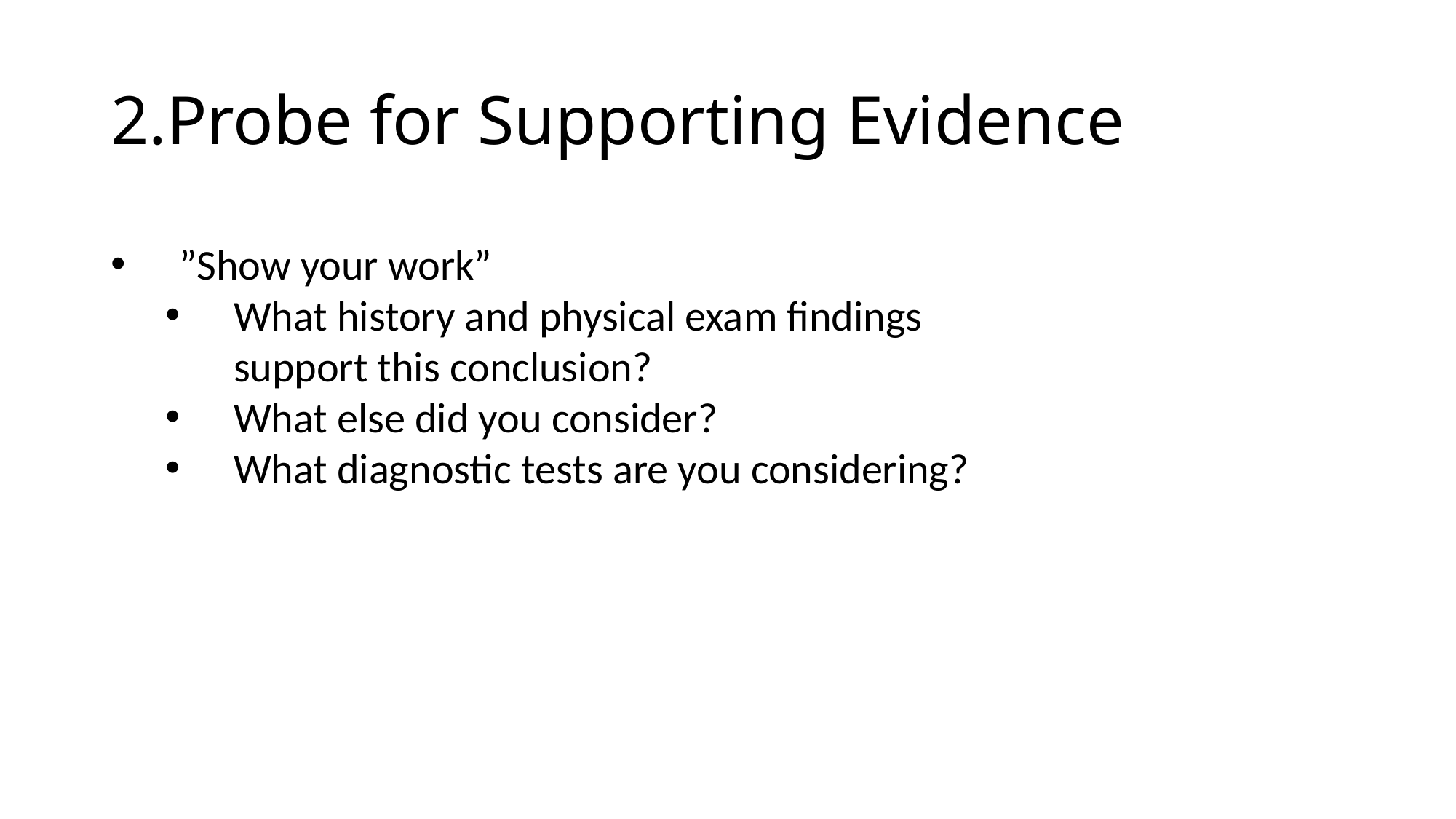

# 2.Probe for Supporting Evidence
”Show your work”
What history and physical exam findings support this conclusion?
What else did you consider?
What diagnostic tests are you considering?

## Slide 8
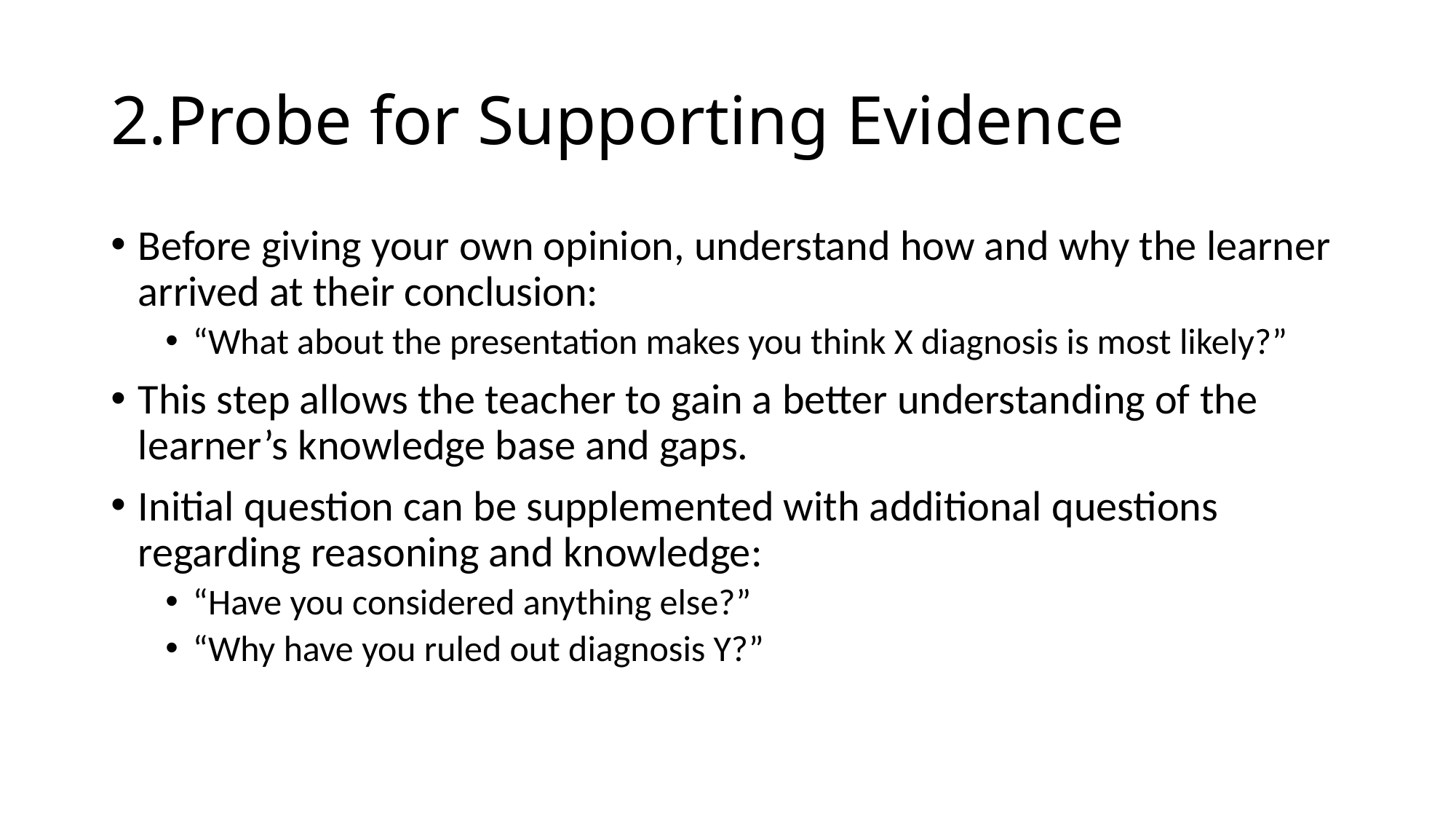

# 2.Probe for Supporting Evidence
Before giving your own opinion, understand how and why the learner arrived at their conclusion:
“What about the presentation makes you think X diagnosis is most likely?”
This step allows the teacher to gain a better understanding of the learner’s knowledge base and gaps.
Initial question can be supplemented with additional questions regarding reasoning and knowledge:
“Have you considered anything else?”
“Why have you ruled out diagnosis Y?”

## Slide 9
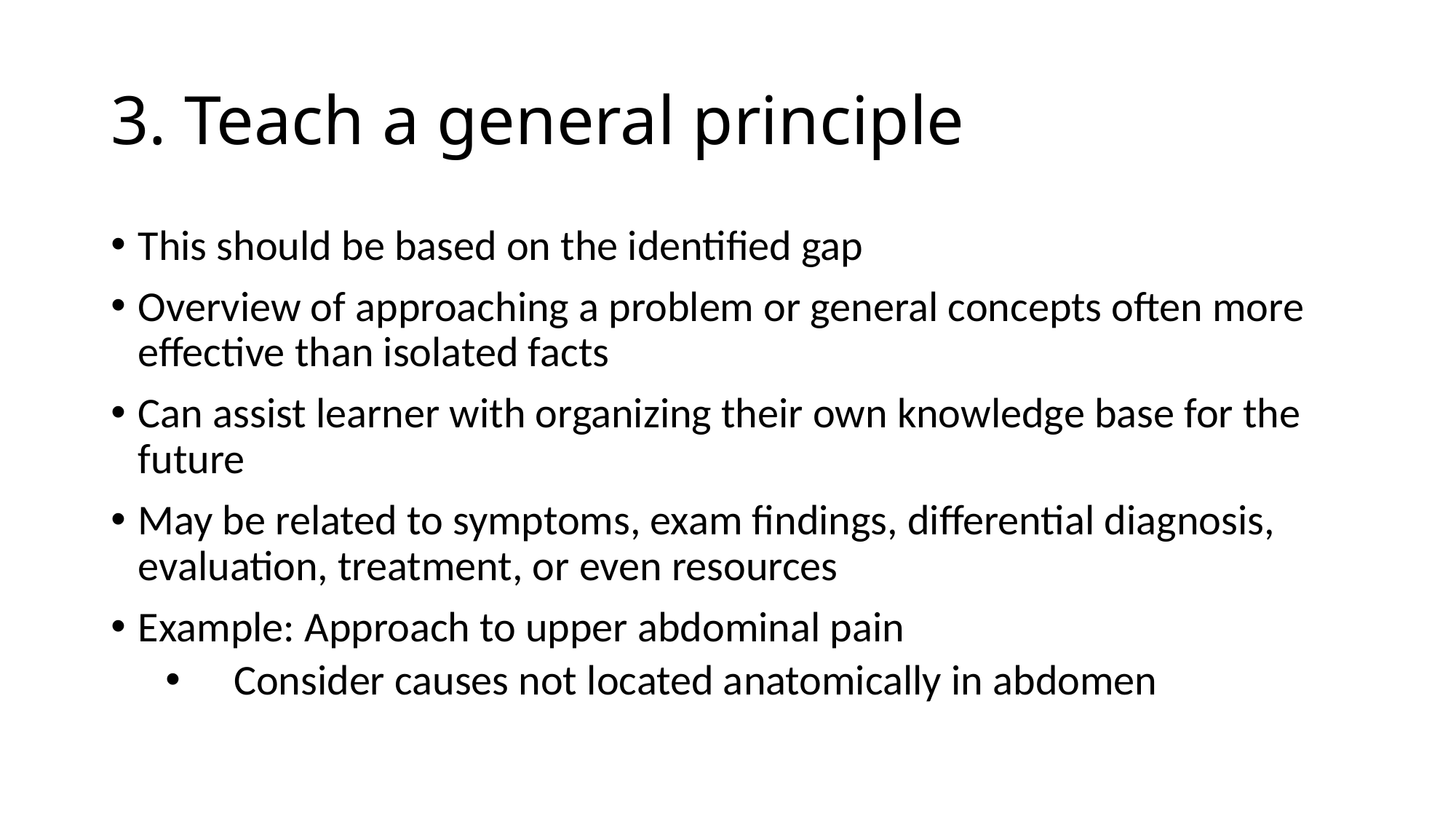

# 3. Teach a general principle
This should be based on the identified gap
Overview of approaching a problem or general concepts often more effective than isolated facts
Can assist learner with organizing their own knowledge base for the future
May be related to symptoms, exam findings, differential diagnosis, evaluation, treatment, or even resources
Example: Approach to upper abdominal pain
Consider causes not located anatomically in abdomen

## Slide 10
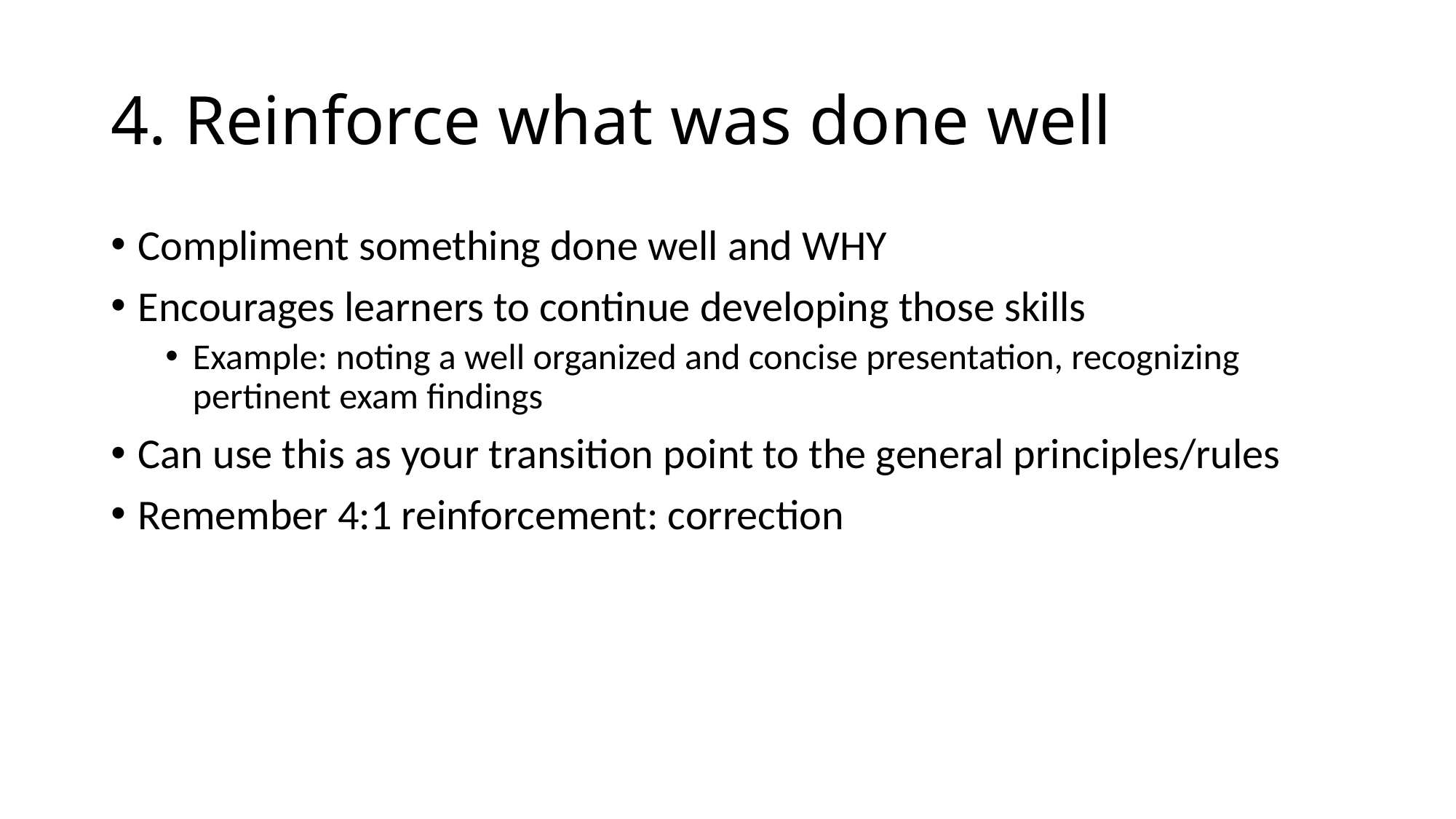

# 4. Reinforce what was done well
Compliment something done well and WHY
Encourages learners to continue developing those skills
Example: noting a well organized and concise presentation, recognizing pertinent exam findings
Can use this as your transition point to the general principles/rules
Remember 4:1 reinforcement: correction

## Slide 11
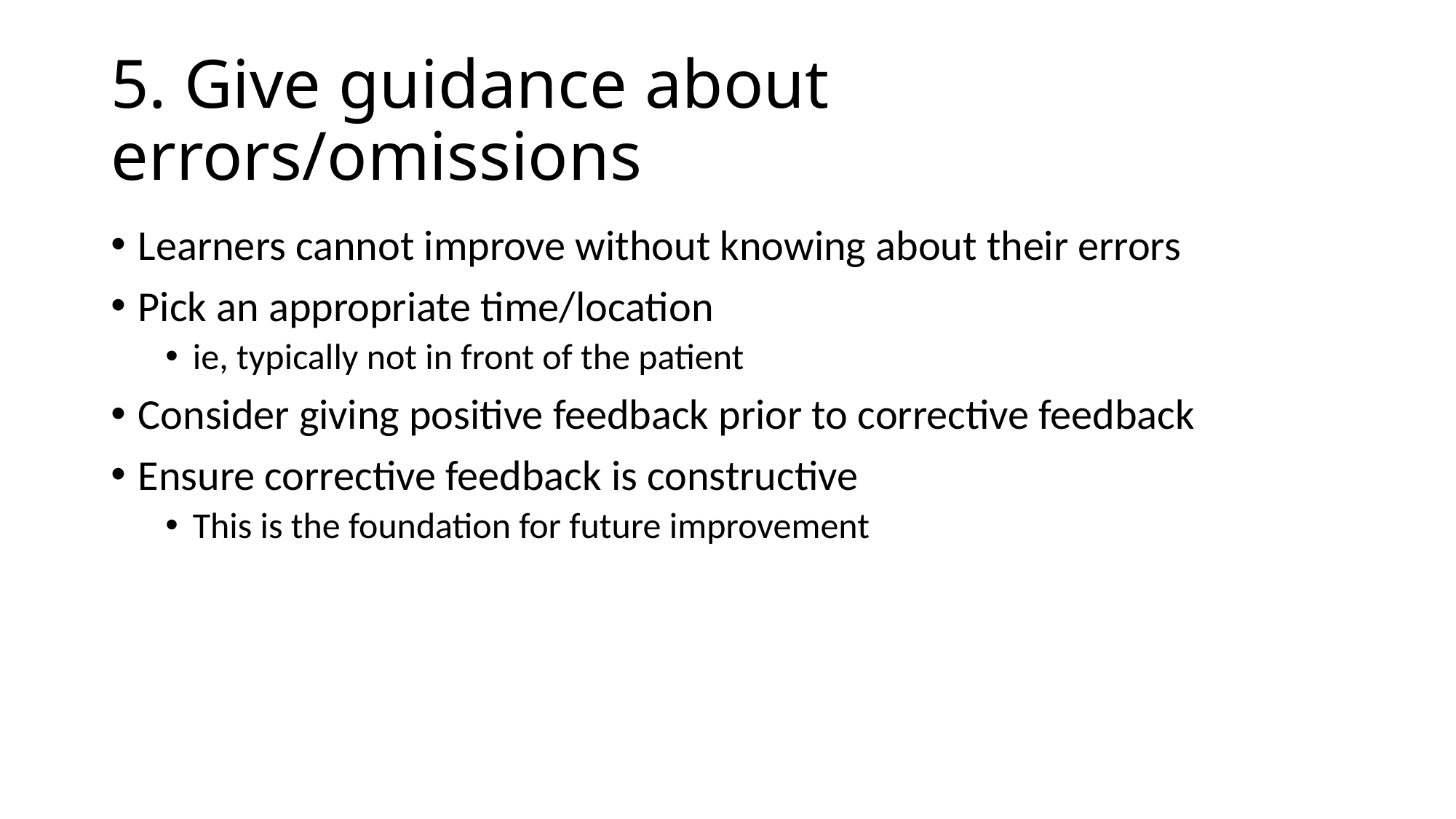

# 5. Give guidance about errors/omissions
Learners cannot improve without knowing about their errors
Pick an appropriate time/location
ie, typically not in front of the patient
Consider giving positive feedback prior to corrective feedback
Ensure corrective feedback is constructive
This is the foundation for future improvement

## Slide 12
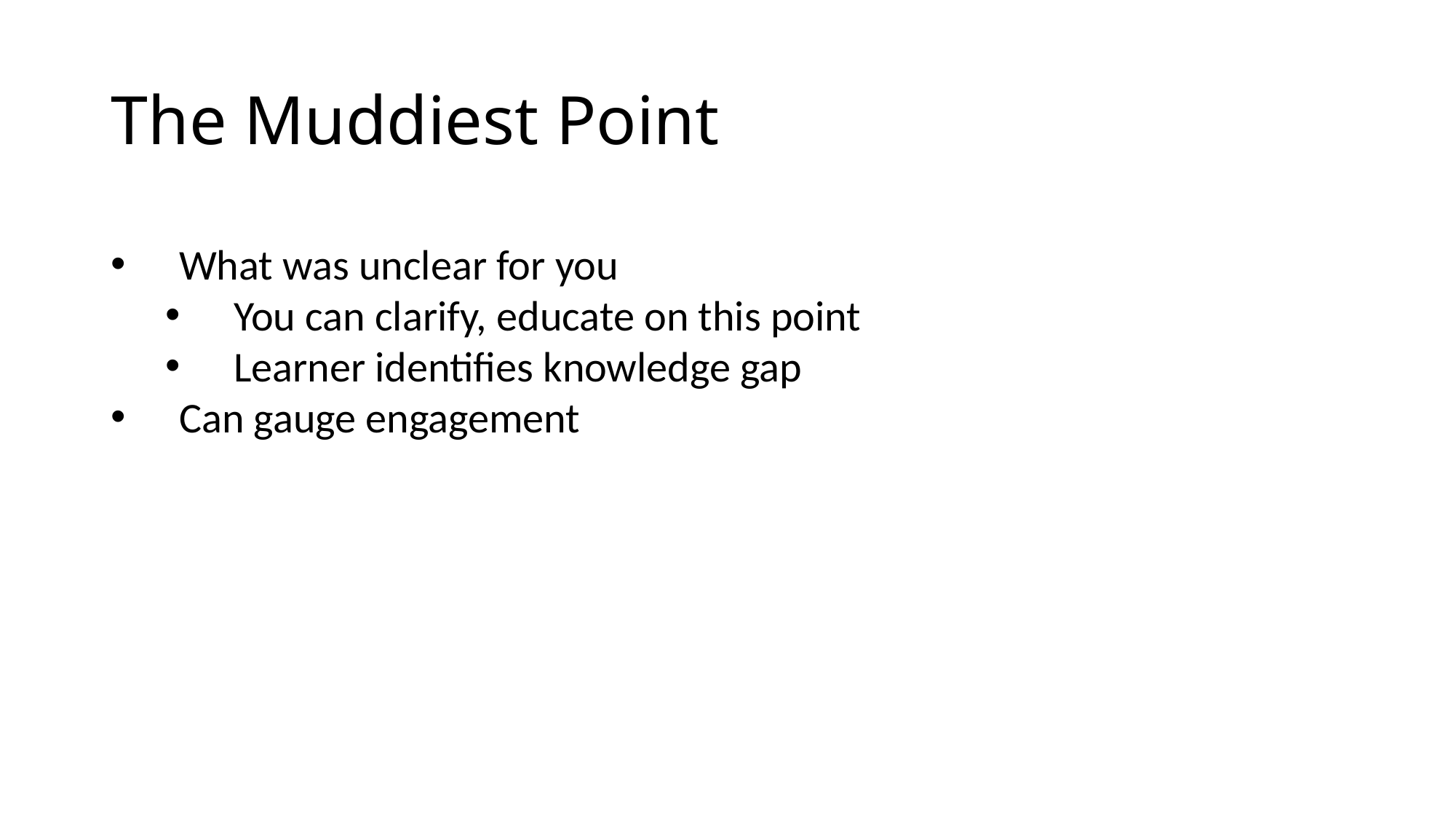

# The Muddiest Point
What was unclear for you
You can clarify, educate on this point
Learner identifies knowledge gap
Can gauge engagement

## Slide 13
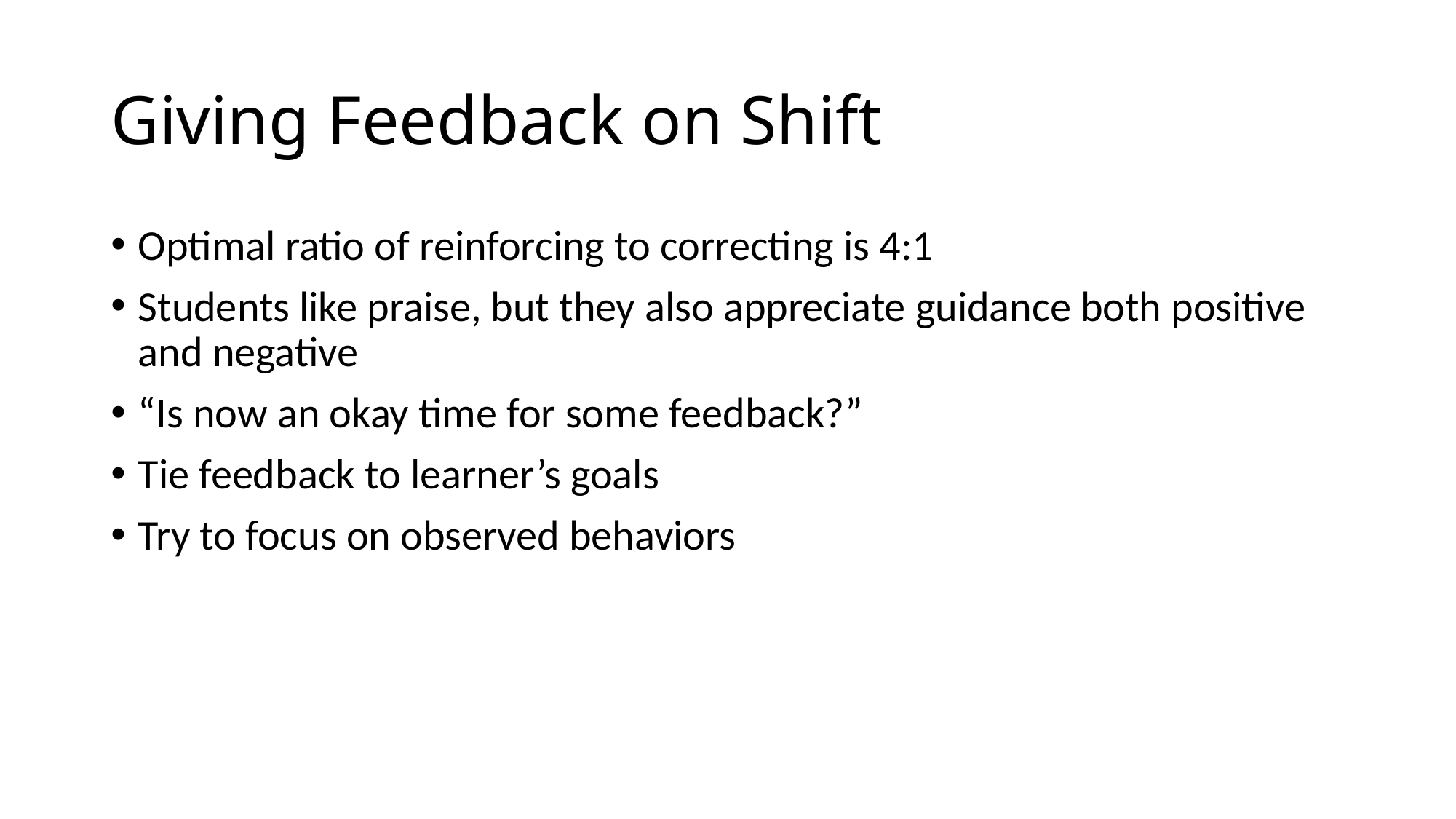

# Giving Feedback on Shift
Optimal ratio of reinforcing to correcting is 4:1
Students like praise, but they also appreciate guidance both positive and negative
“Is now an okay time for some feedback?”
Tie feedback to learner’s goals
Try to focus on observed behaviors

## Slide 14
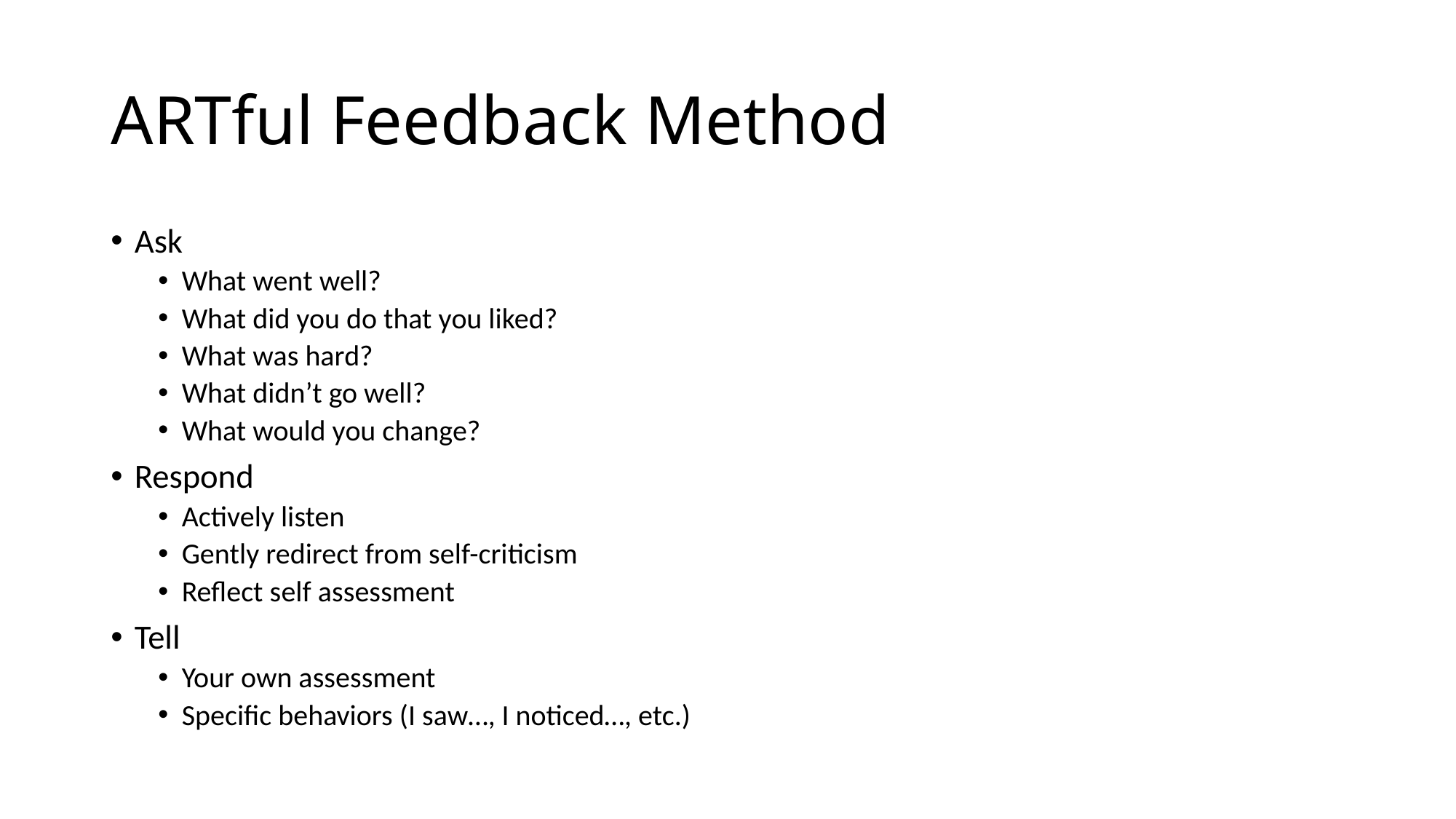

# ARTful Feedback Method
Ask
What went well?
What did you do that you liked?
What was hard?
What didn’t go well?
What would you change?
Respond
Actively listen
Gently redirect from self-criticism
Reflect self assessment
Tell
Your own assessment
Specific behaviors (I saw…, I noticed…, etc.)

## Slide 15
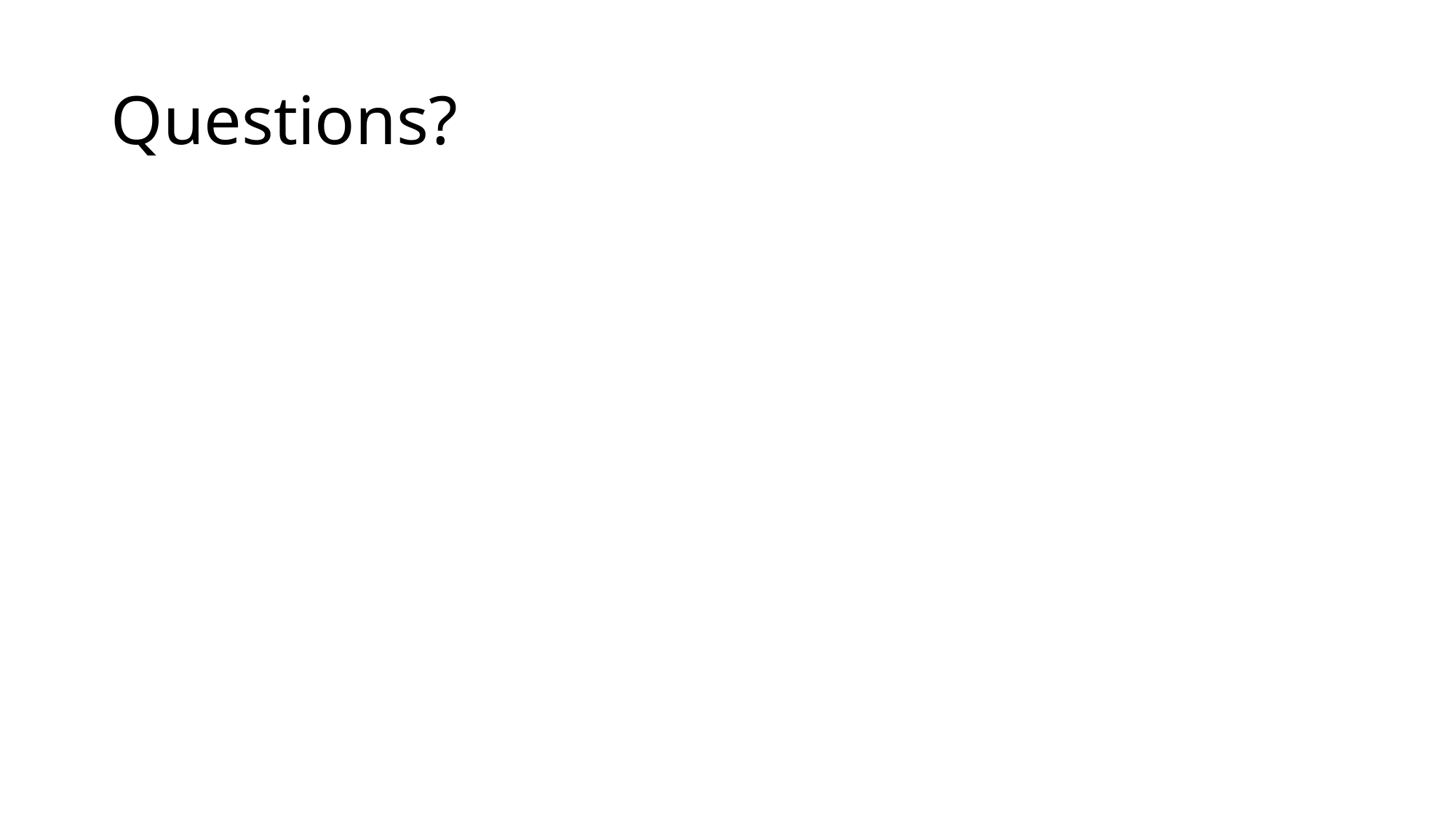

# Questions?

## Slide 16
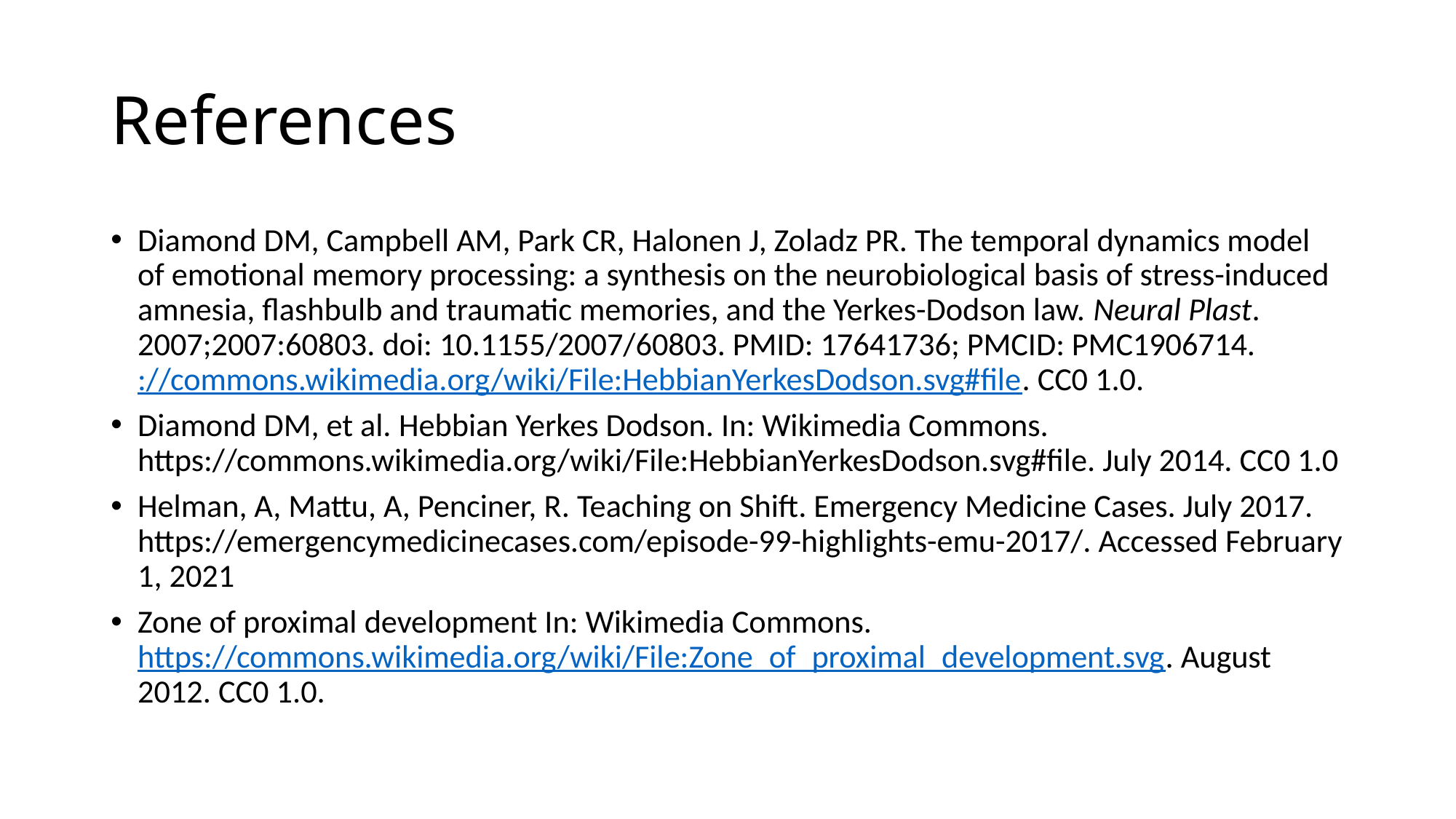

# References
Diamond DM, Campbell AM, Park CR, Halonen J, Zoladz PR. The temporal dynamics model of emotional memory processing: a synthesis on the neurobiological basis of stress-induced amnesia, flashbulb and traumatic memories, and the Yerkes-Dodson law. Neural Plast. 2007;2007:60803. doi: 10.1155/2007/60803. PMID: 17641736; PMCID: PMC1906714.://commons.wikimedia.org/wiki/File:HebbianYerkesDodson.svg#file. CC0 1.0.
Diamond DM, et al. Hebbian Yerkes Dodson. In: Wikimedia Commons. https://commons.wikimedia.org/wiki/File:HebbianYerkesDodson.svg#file. July 2014. CC0 1.0
Helman, A, Mattu, A, Penciner, R. Teaching on Shift. Emergency Medicine Cases. July 2017. https://emergencymedicinecases.com/episode-99-highlights-emu-2017/. Accessed February 1, 2021
Zone of proximal development In: Wikimedia Commons. https://commons.wikimedia.org/wiki/File:Zone_of_proximal_development.svg. August 2012. CC0 1.0.
