# Supplementary material for: Residents Are Coming: A Faculty Development Curriculum to Prepare a Community Site For New Learners
Source: J Educ Teach Emerg Med. 2022 Jul 15;7(3):C1–C41. doi: 10.21980/J87D2N (PMC10332697; doi:10.21980/J87D2N)
Supplement: Supplementary file 5 — Please see associated PowerPoint file [file jetem-7-3-c1-appendix7.pptx]

## Slide 1
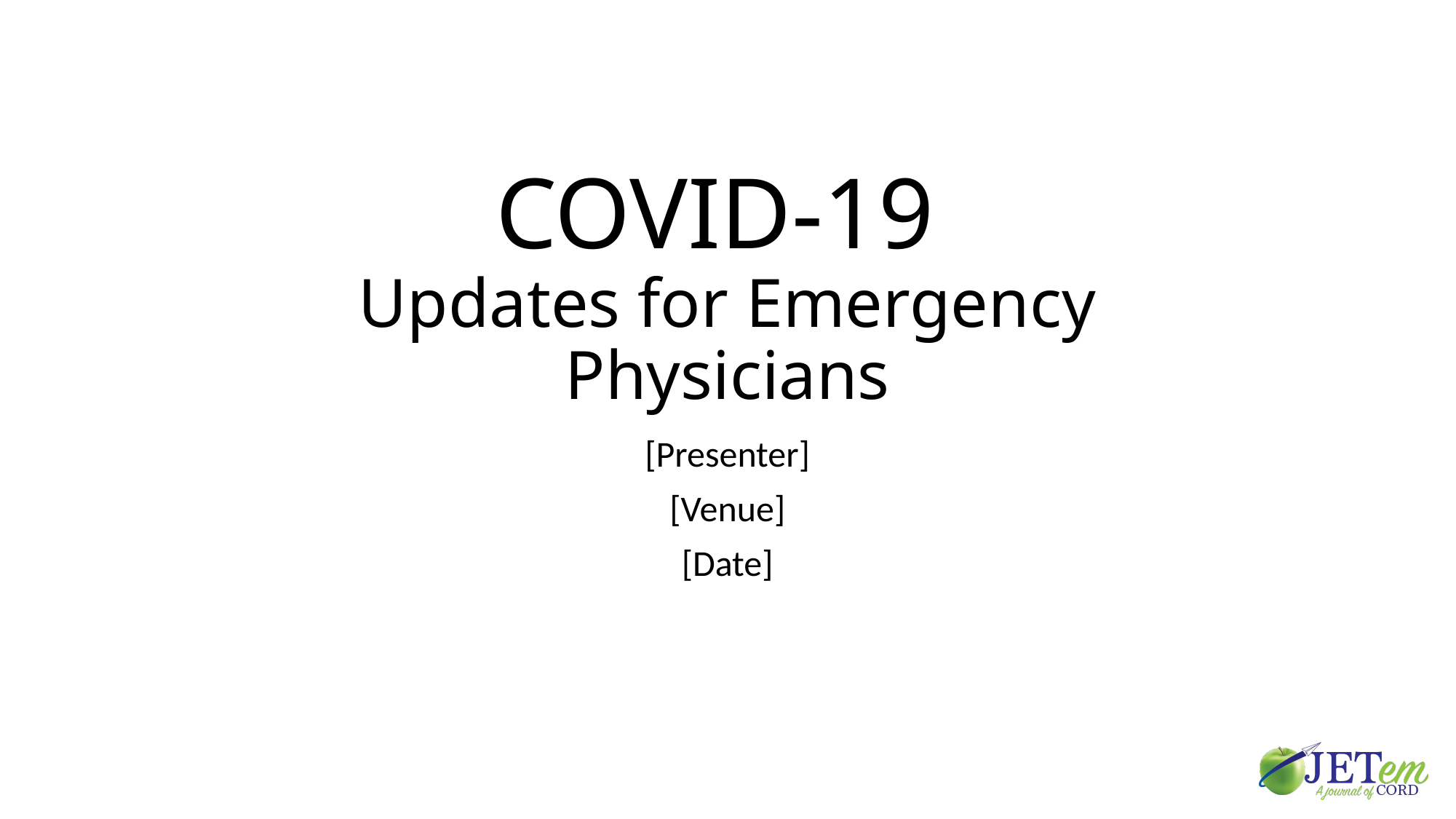

# COVID-19 Updates for Emergency Physicians
[Presenter]
[Venue]
[Date]

## Slide 2
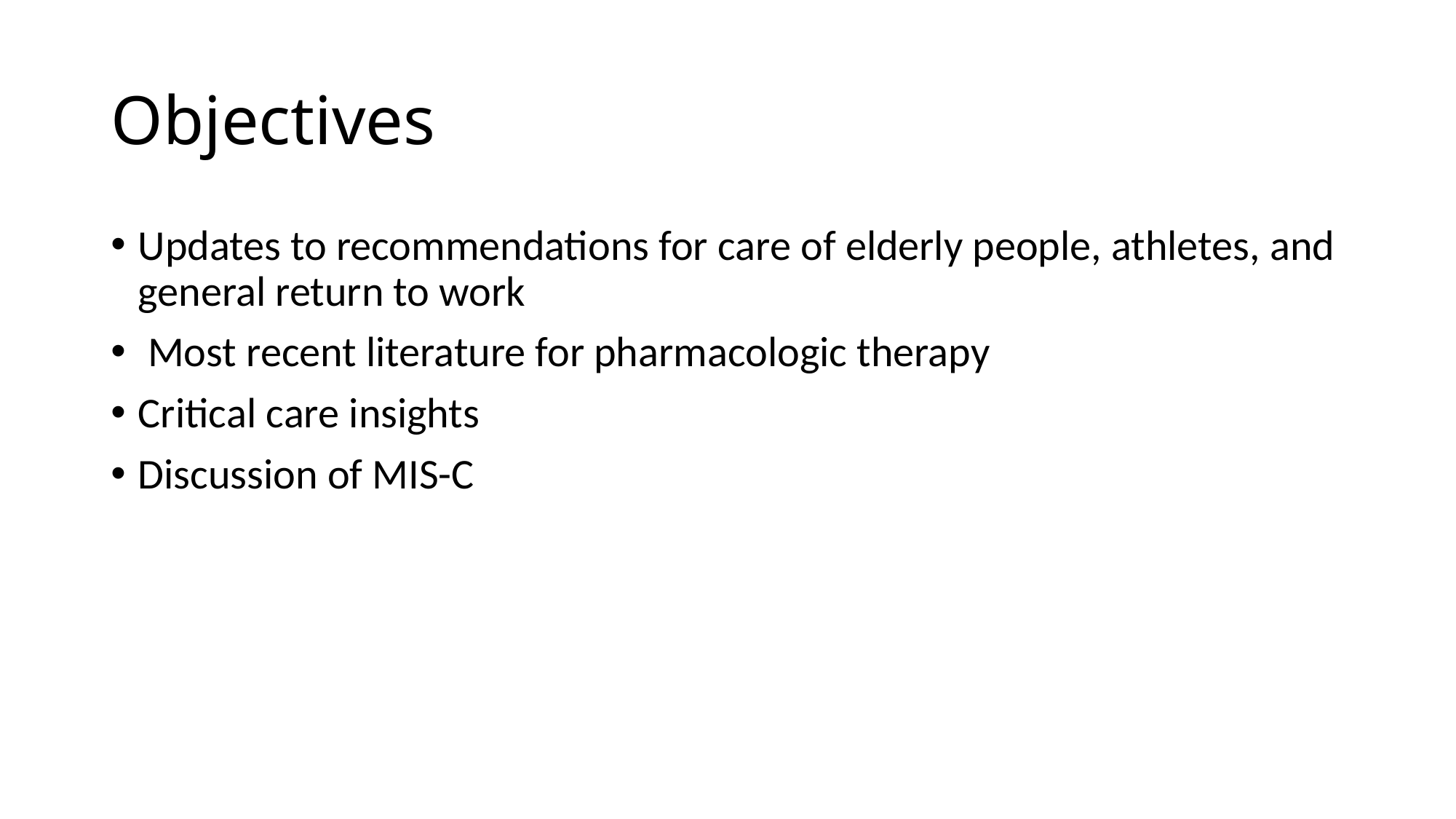

# Objectives
Updates to recommendations for care of elderly people, athletes, and general return to work
 Most recent literature for pharmacologic therapy
Critical care insights
Discussion of MIS-C

## Slide 3
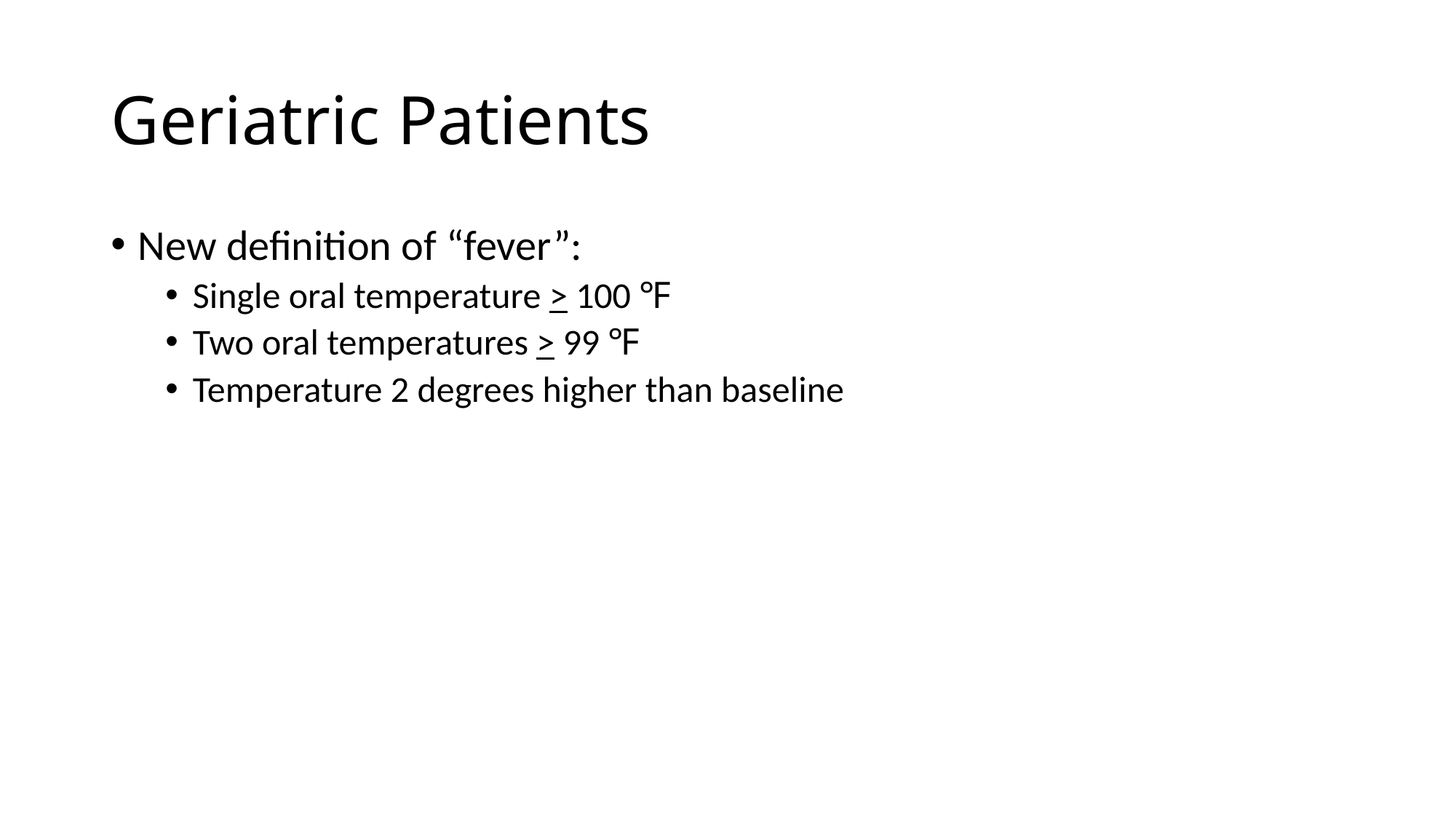

# Geriatric Patients
New definition of “fever”:
Single oral temperature > 100 ℉
Two oral temperatures > 99 ℉
Temperature 2 degrees higher than baseline

## Slide 4
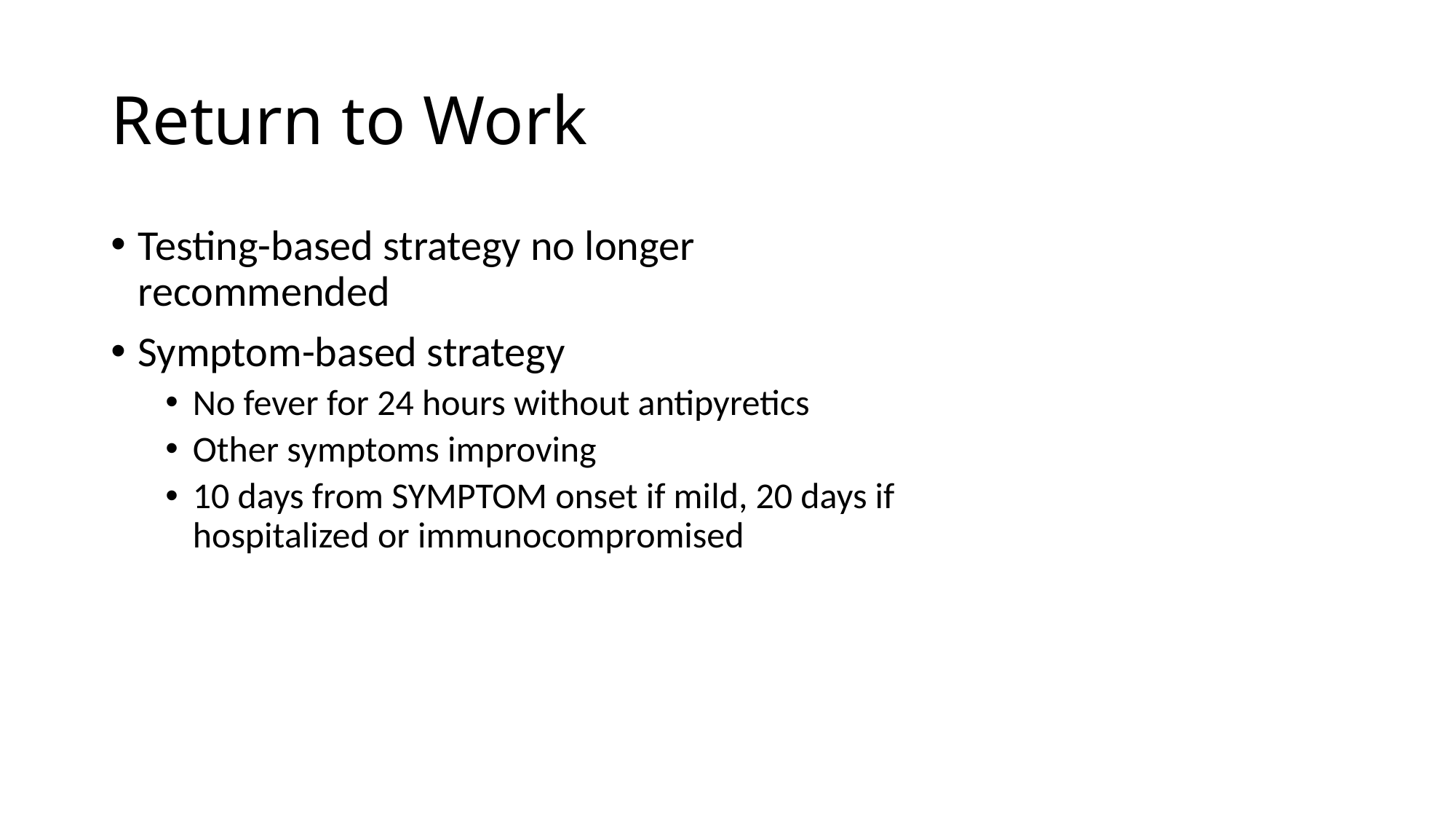

# Return to Work
Testing-based strategy no longer recommended
Symptom-based strategy
No fever for 24 hours without antipyretics
Other symptoms improving
10 days from SYMPTOM onset if mild, 20 days if hospitalized or immunocompromised

## Slide 5
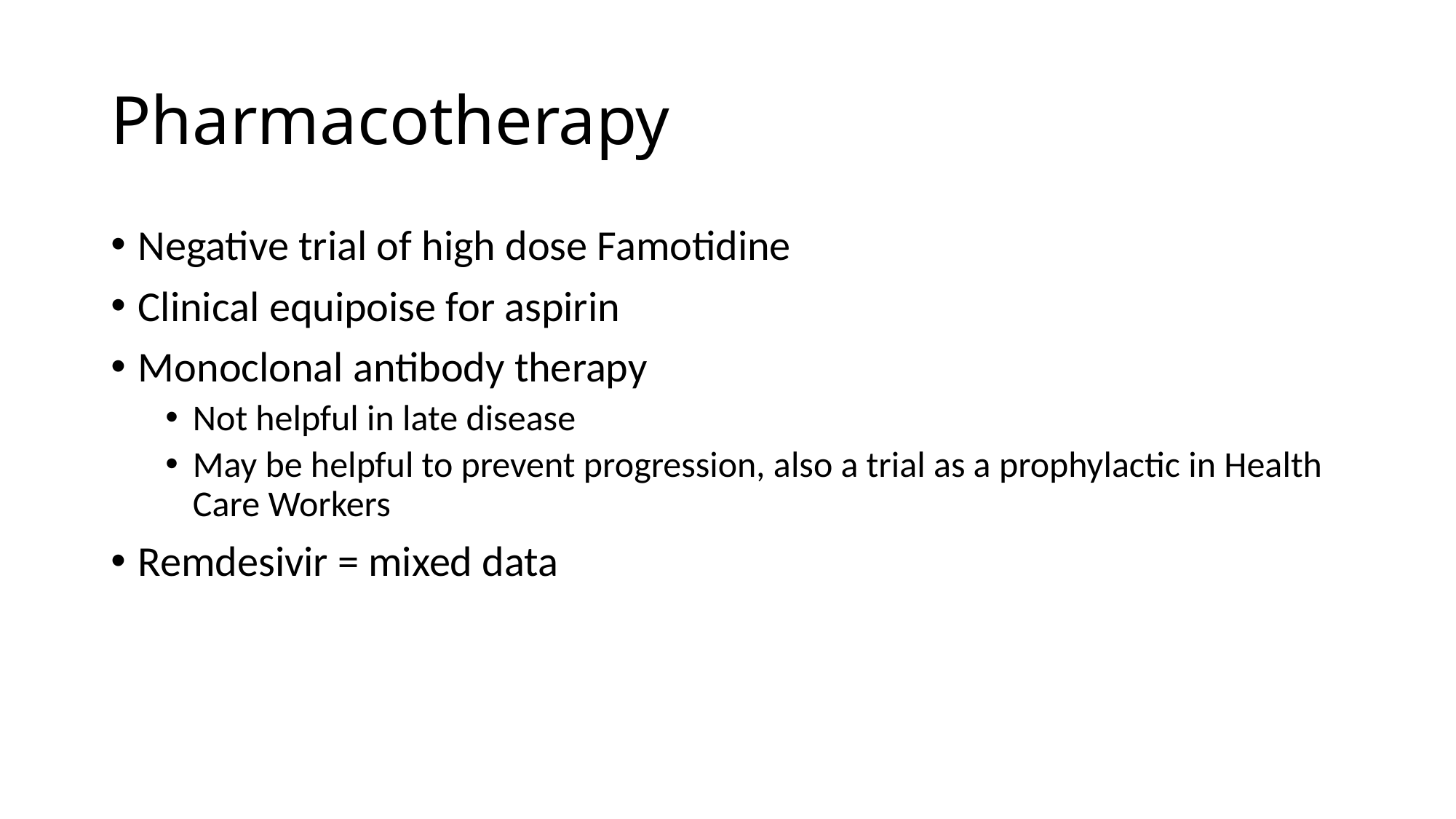

# Pharmacotherapy
Negative trial of high dose Famotidine
Clinical equipoise for aspirin
Monoclonal antibody therapy
Not helpful in late disease
May be helpful to prevent progression, also a trial as a prophylactic in Health Care Workers
Remdesivir = mixed data

## Slide 6
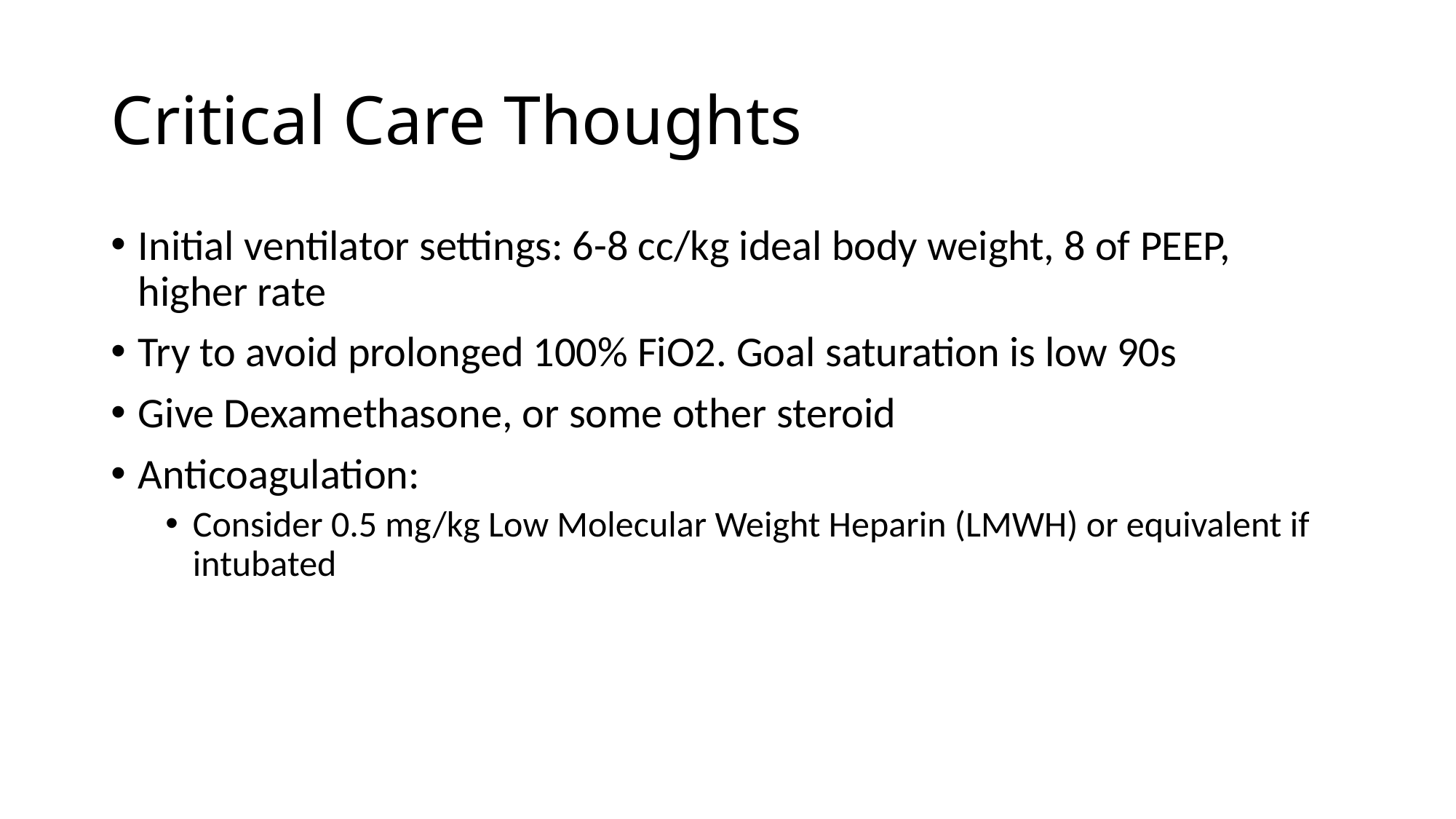

# Critical Care Thoughts
Initial ventilator settings: 6-8 cc/kg ideal body weight, 8 of PEEP, higher rate
Try to avoid prolonged 100% FiO2. Goal saturation is low 90s
Give Dexamethasone, or some other steroid
Anticoagulation:
Consider 0.5 mg/kg Low Molecular Weight Heparin (LMWH) or equivalent if intubated

## Slide 7
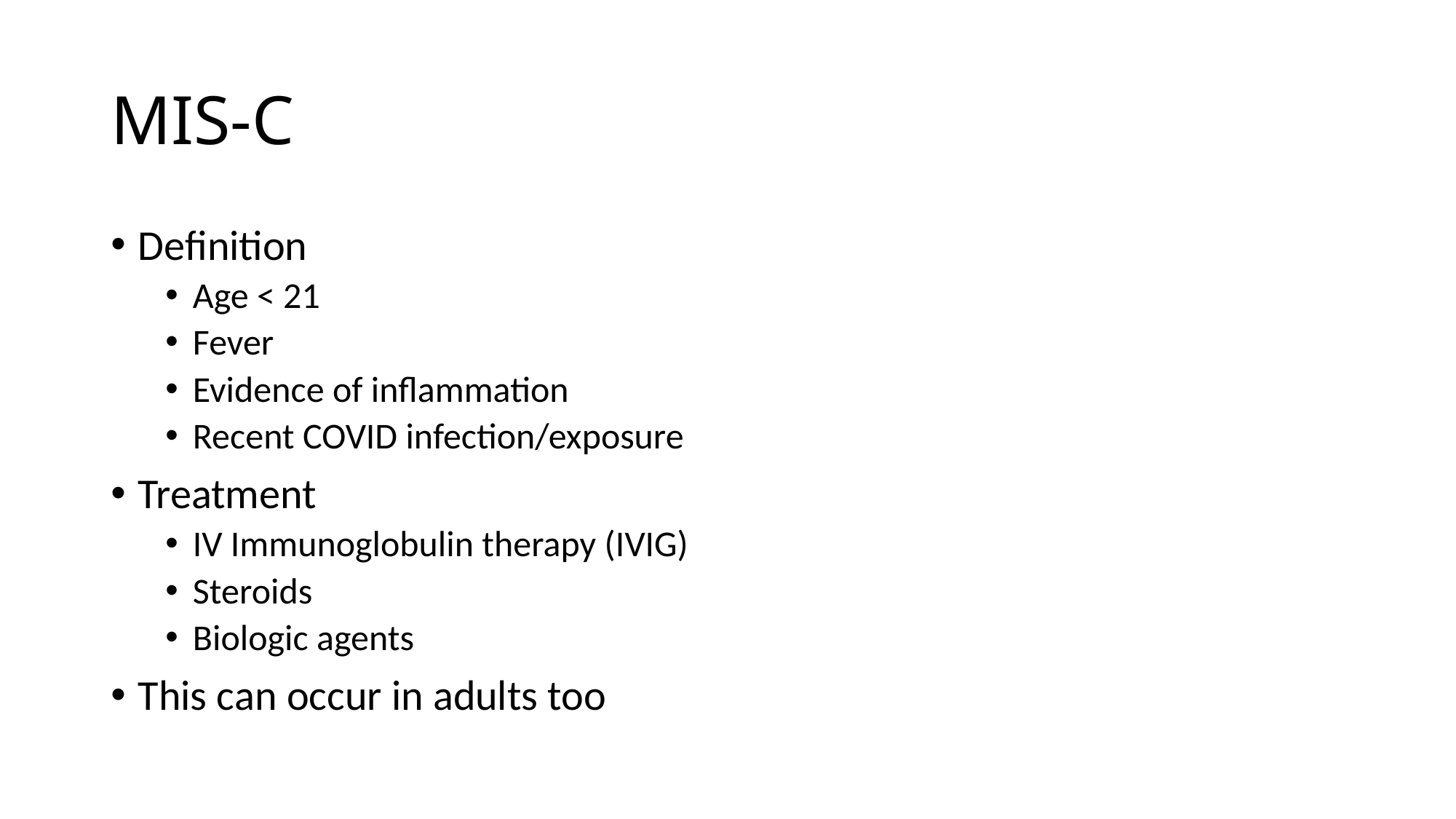

# MIS-C
Definition
Age < 21
Fever
Evidence of inflammation
Recent COVID infection/exposure
Treatment
IV Immunoglobulin therapy (IVIG)
Steroids
Biologic agents
This can occur in adults too

## Slide 8
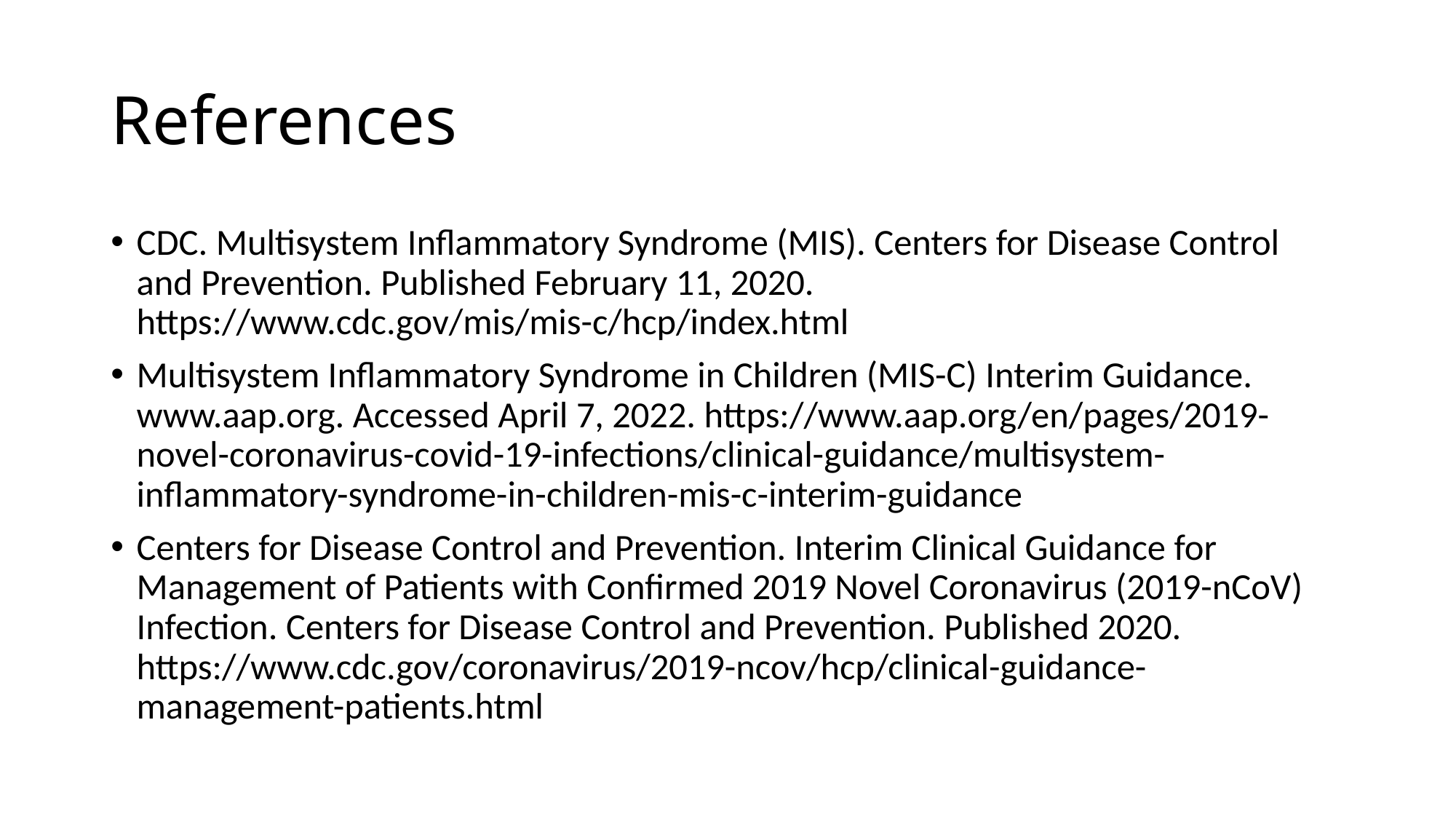

# References
CDC. Multisystem Inflammatory Syndrome (MIS). Centers for Disease Control and Prevention. Published February 11, 2020. https://www.cdc.gov/mis/mis-c/hcp/index.html
Multisystem Inflammatory Syndrome in Children (MIS-C) Interim Guidance. www.aap.org. Accessed April 7, 2022. https://www.aap.org/en/pages/2019-novel-coronavirus-covid-19-infections/clinical-guidance/multisystem-inflammatory-syndrome-in-children-mis-c-interim-guidance
Centers for Disease Control and Prevention. Interim Clinical Guidance for Management of Patients with Confirmed 2019 Novel Coronavirus (2019-nCoV) Infection. Centers for Disease Control and Prevention. Published 2020. https://www.cdc.gov/coronavirus/2019-ncov/hcp/clinical-guidance-management-patients.html
