# Supplementary material for: Residents Are Coming: A Faculty Development Curriculum to Prepare a Community Site For New Learners
Source: J Educ Teach Emerg Med. 2022 Jul 15;7(3):C1–C41. doi: 10.21980/J87D2N (PMC10332697; doi:10.21980/J87D2N)
Supplement: Supplementary file 6 — Please see associated PowerPoint file [file jetem-7-3-c1-appendix8.pptx]

## Slide 1
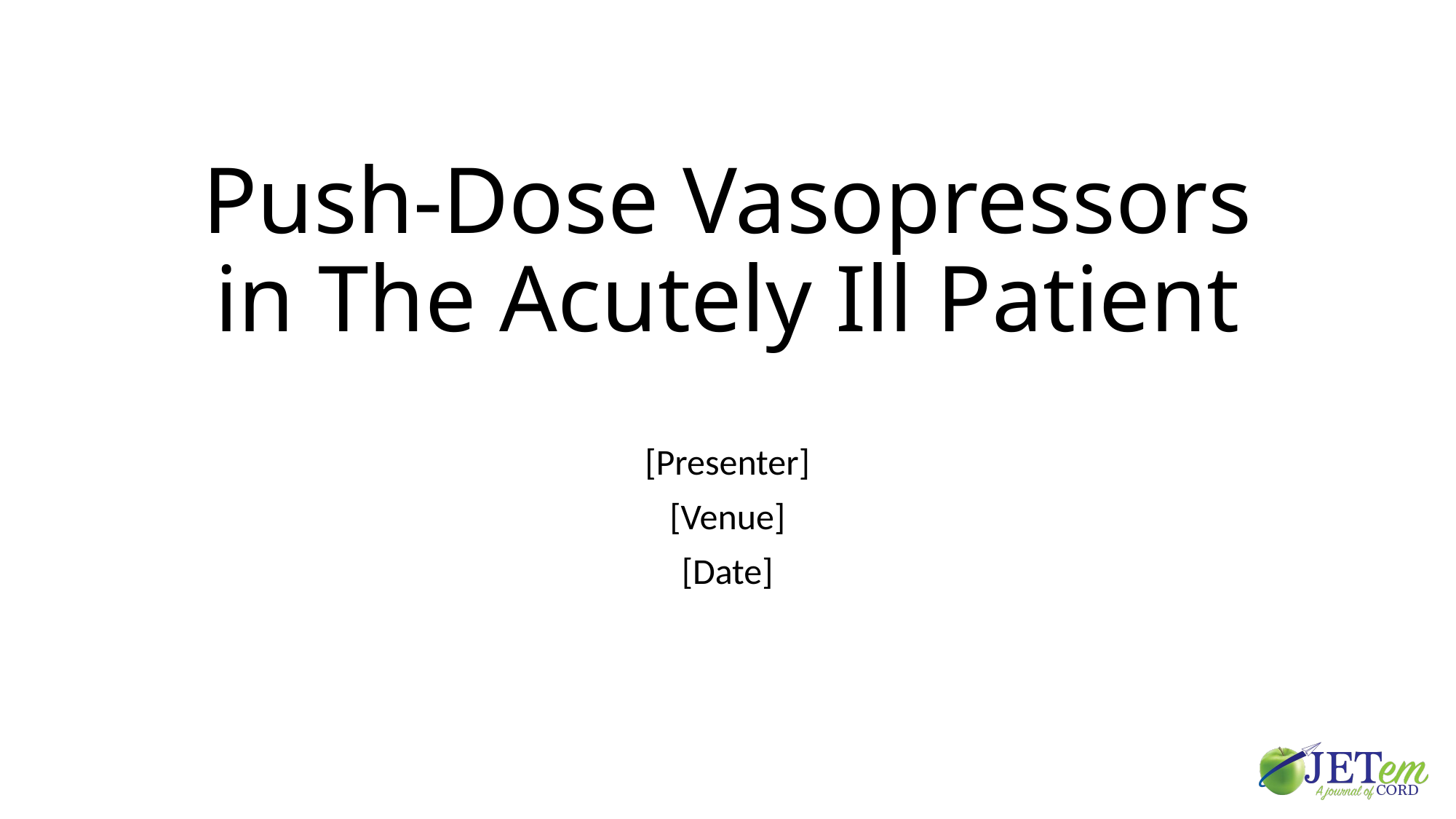

# Push-Dose Vasopressors in The Acutely Ill Patient
[Presenter]
[Venue]
[Date]

## Slide 2
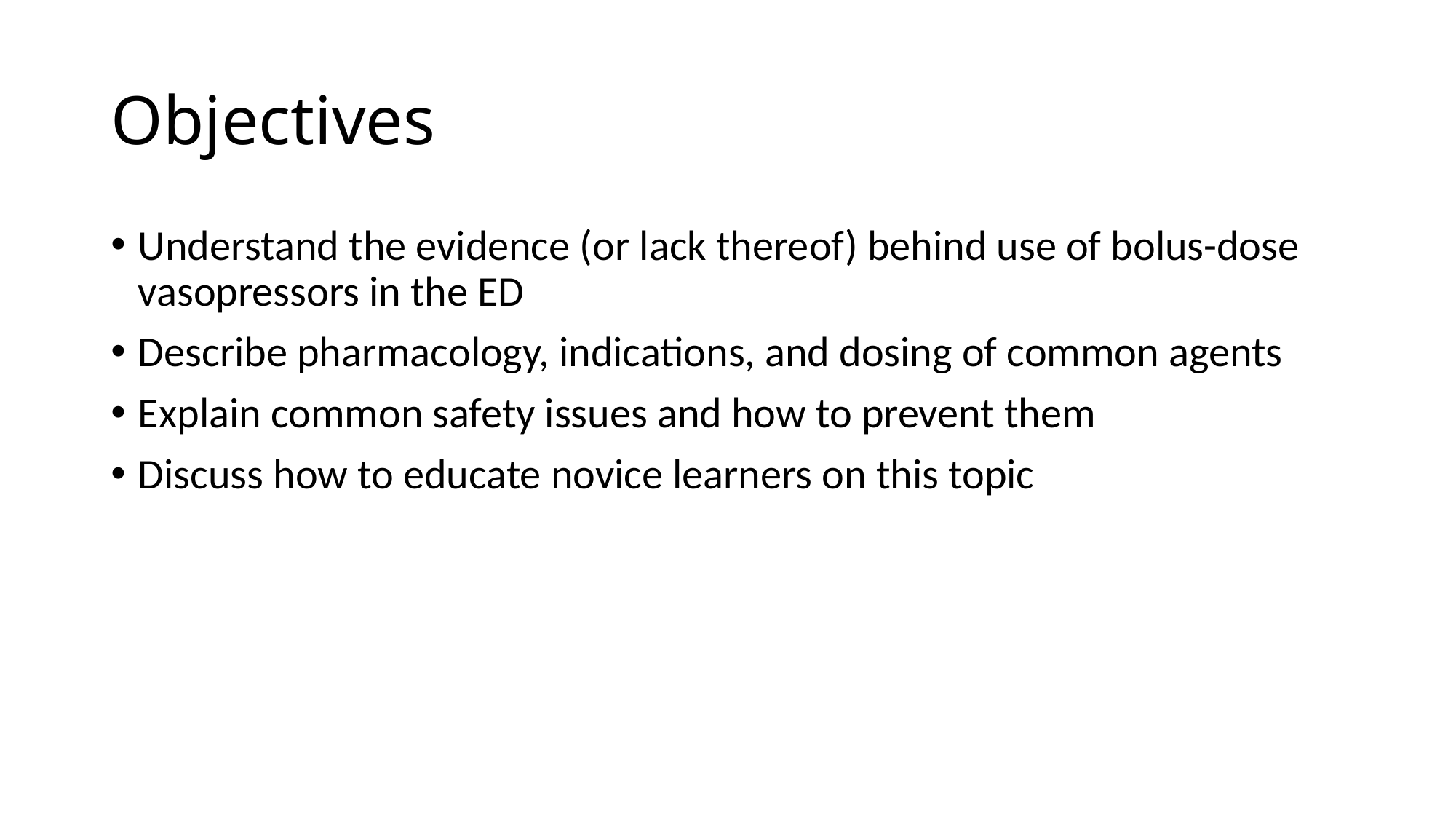

# Objectives
Understand the evidence (or lack thereof) behind use of bolus-dose vasopressors in the ED
Describe pharmacology, indications, and dosing of common agents
Explain common safety issues and how to prevent them
Discuss how to educate novice learners on this topic

## Slide 3
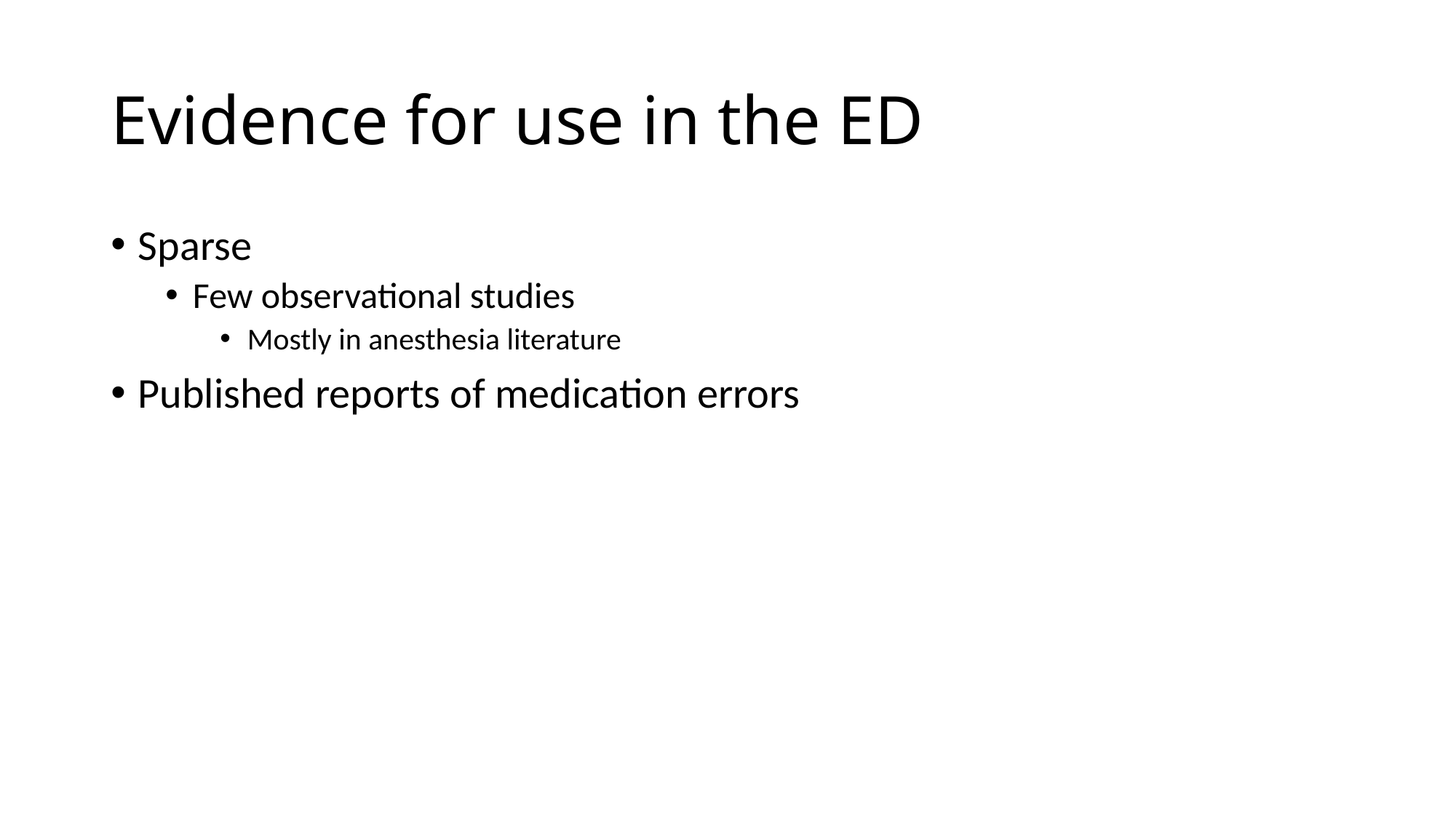

# Evidence for use in the ED
Sparse
Few observational studies
Mostly in anesthesia literature
Published reports of medication errors

## Slide 4
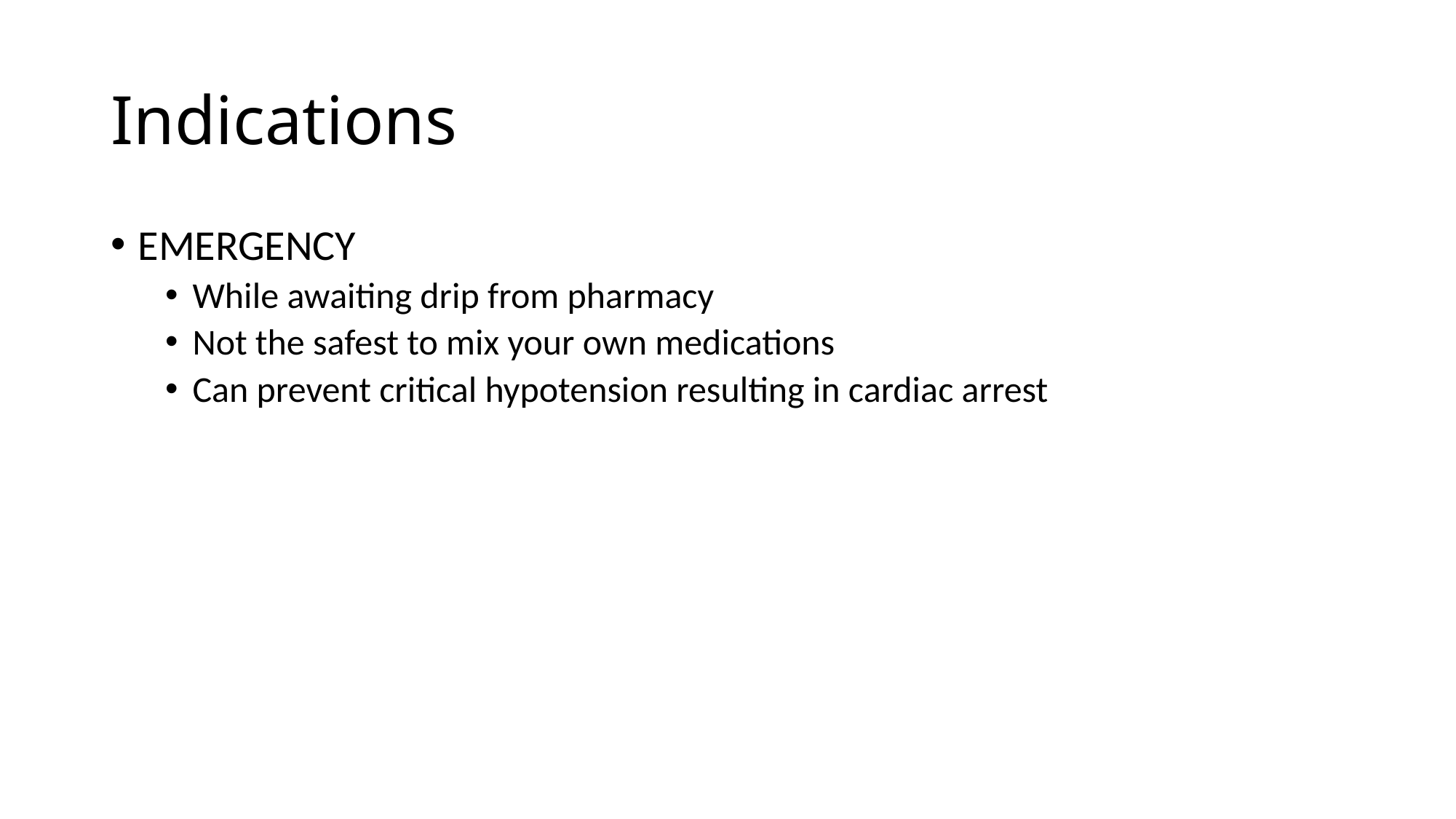

# Indications
EMERGENCY
While awaiting drip from pharmacy
Not the safest to mix your own medications
Can prevent critical hypotension resulting in cardiac arrest

## Slide 5
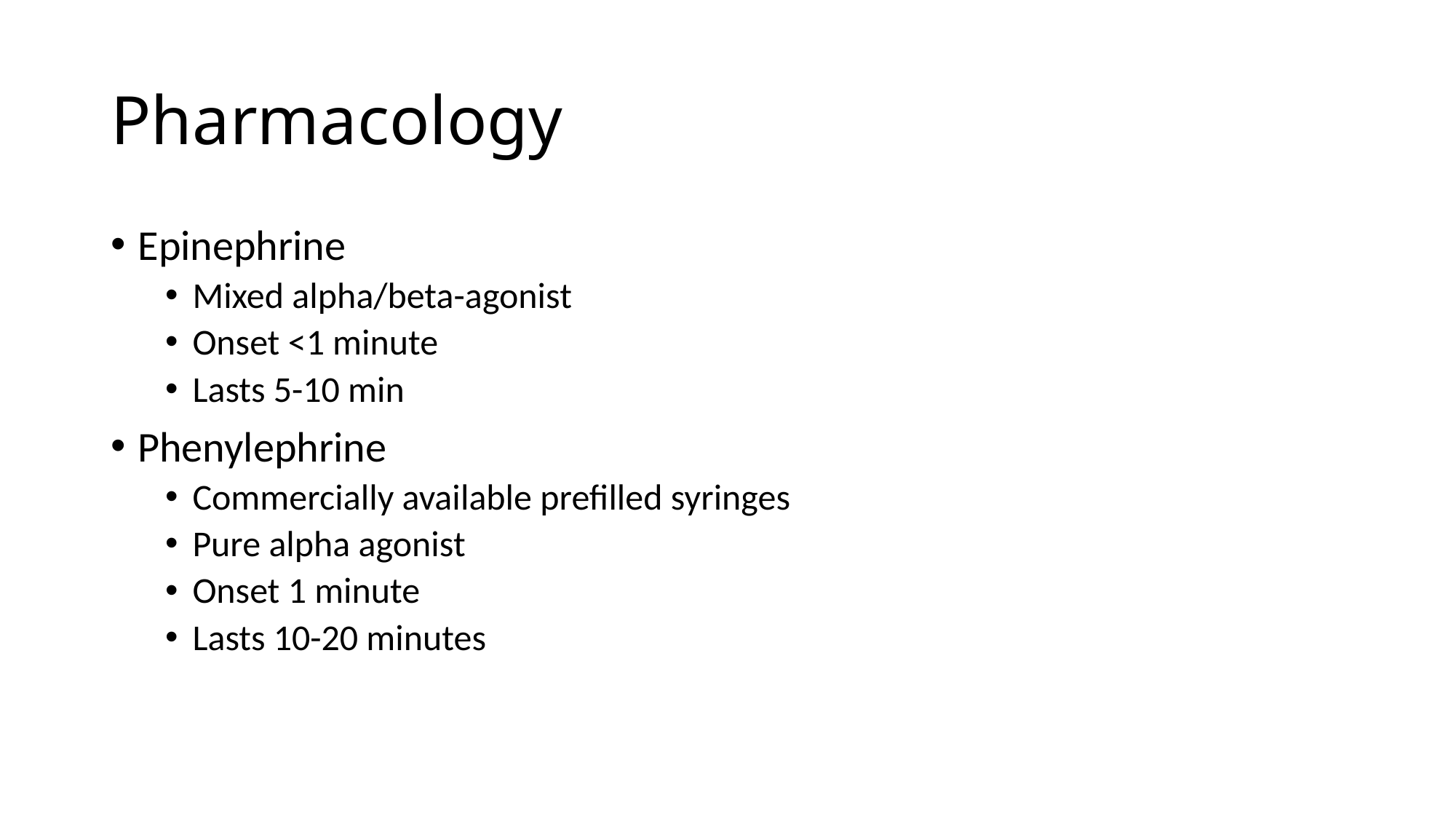

# Pharmacology
Epinephrine
Mixed alpha/beta-agonist
Onset <1 minute
Lasts 5-10 min
Phenylephrine
Commercially available prefilled syringes
Pure alpha agonist
Onset 1 minute
Lasts 10-20 minutes

## Slide 6
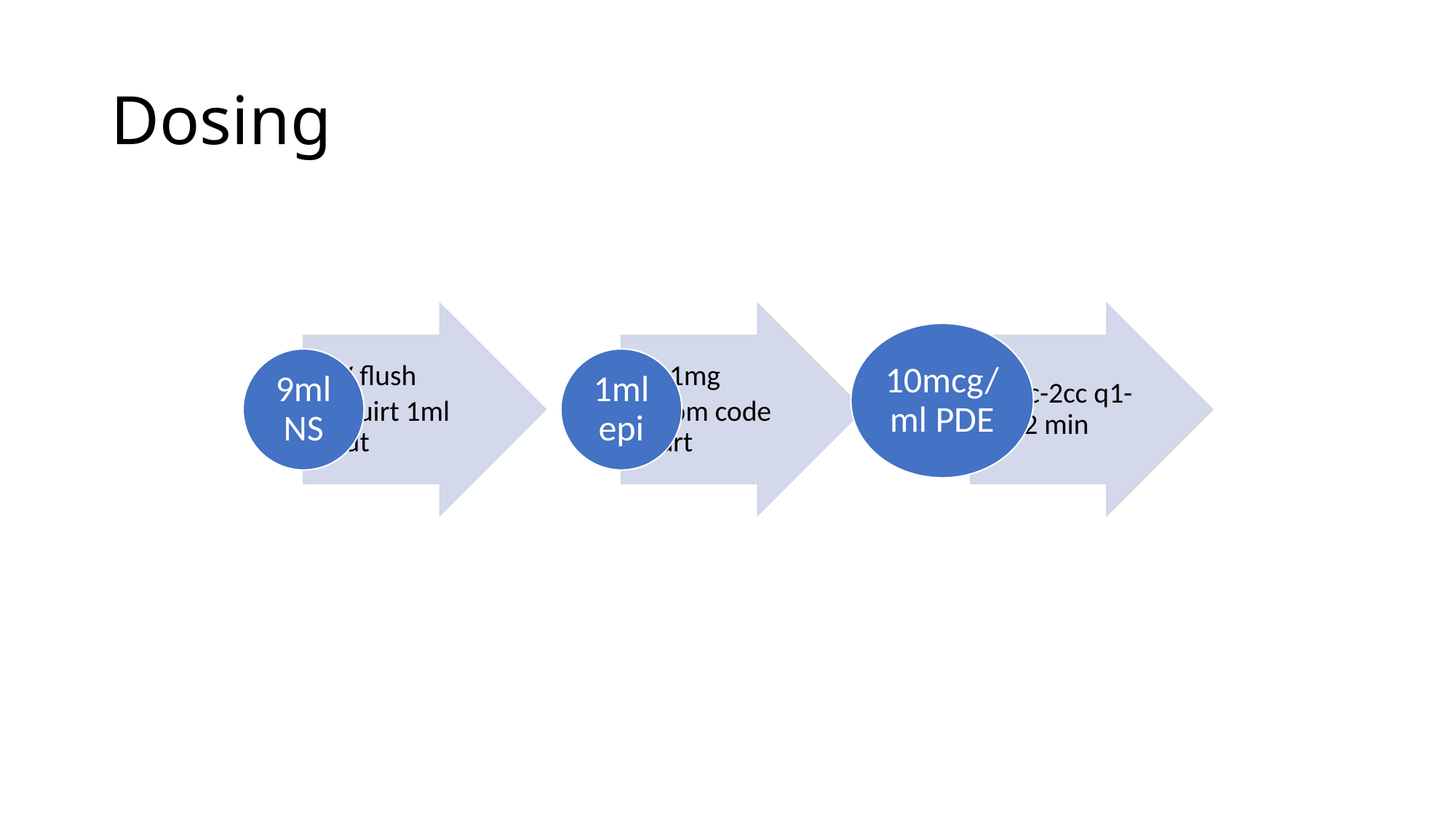

# Dosing

## Slide 7
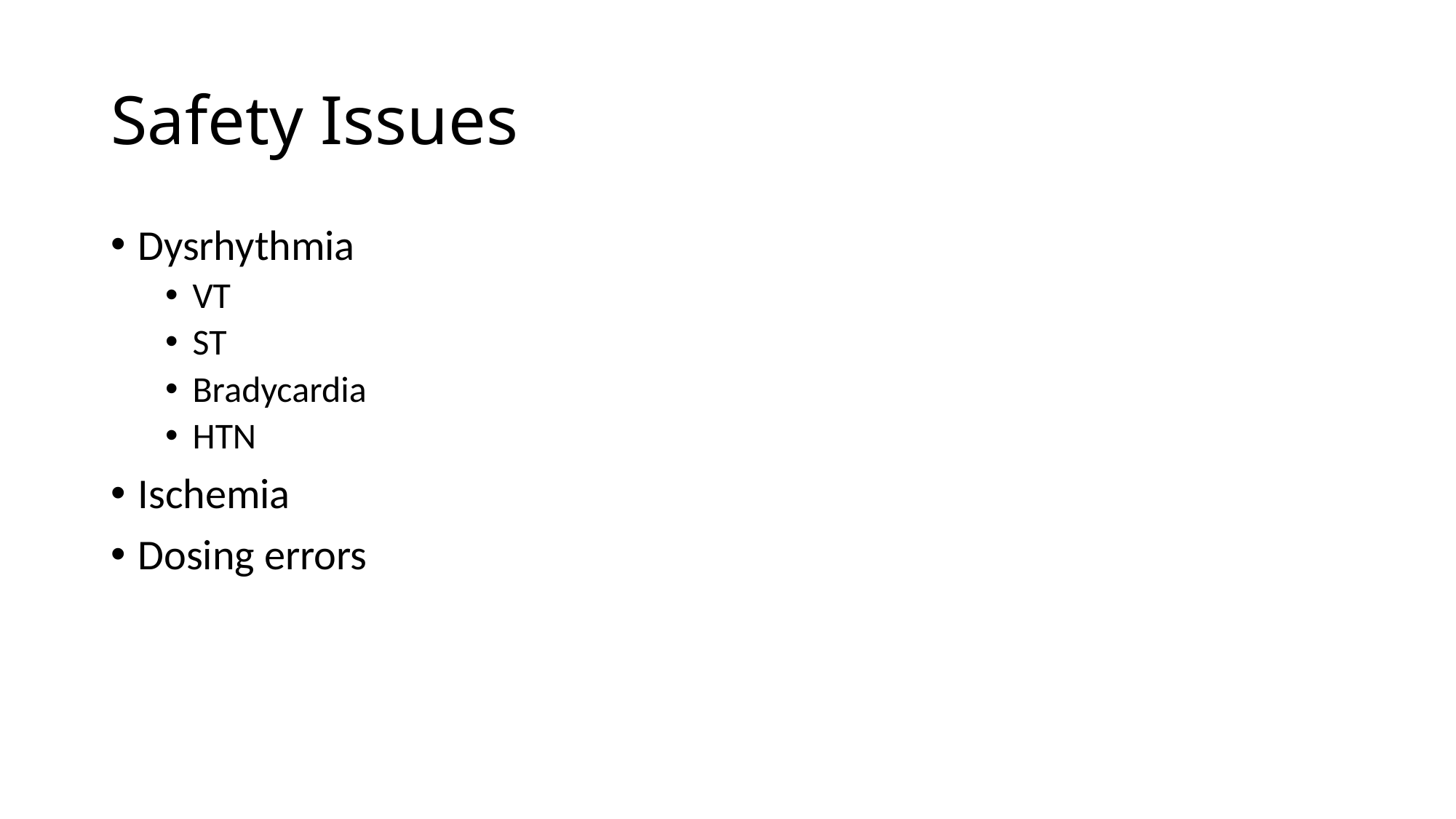

# Safety Issues
Dysrhythmia
VT
ST
Bradycardia
HTN
Ischemia
Dosing errors

## Slide 8
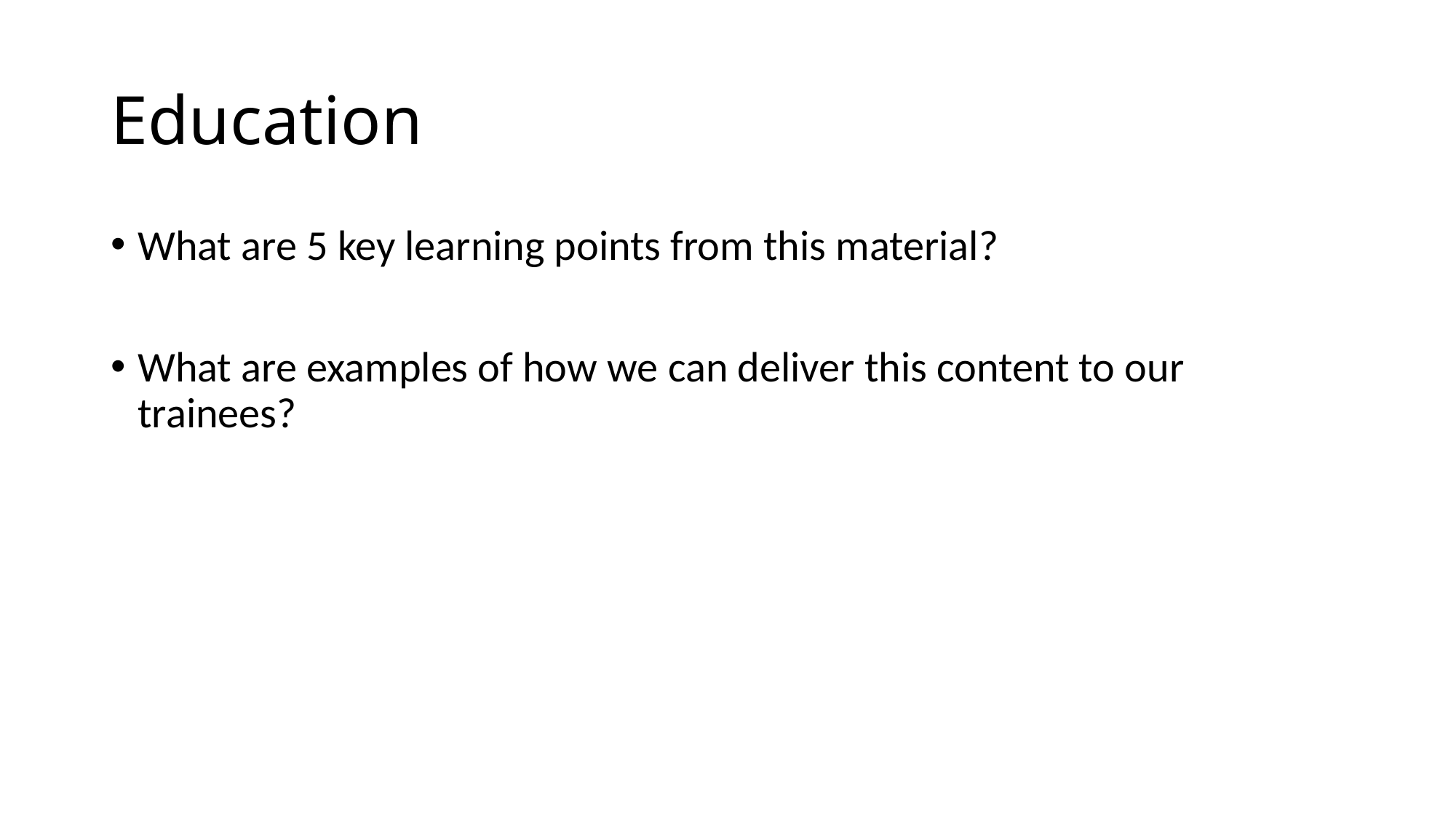

# Education
What are 5 key learning points from this material?
What are examples of how we can deliver this content to our trainees?

## Slide 9
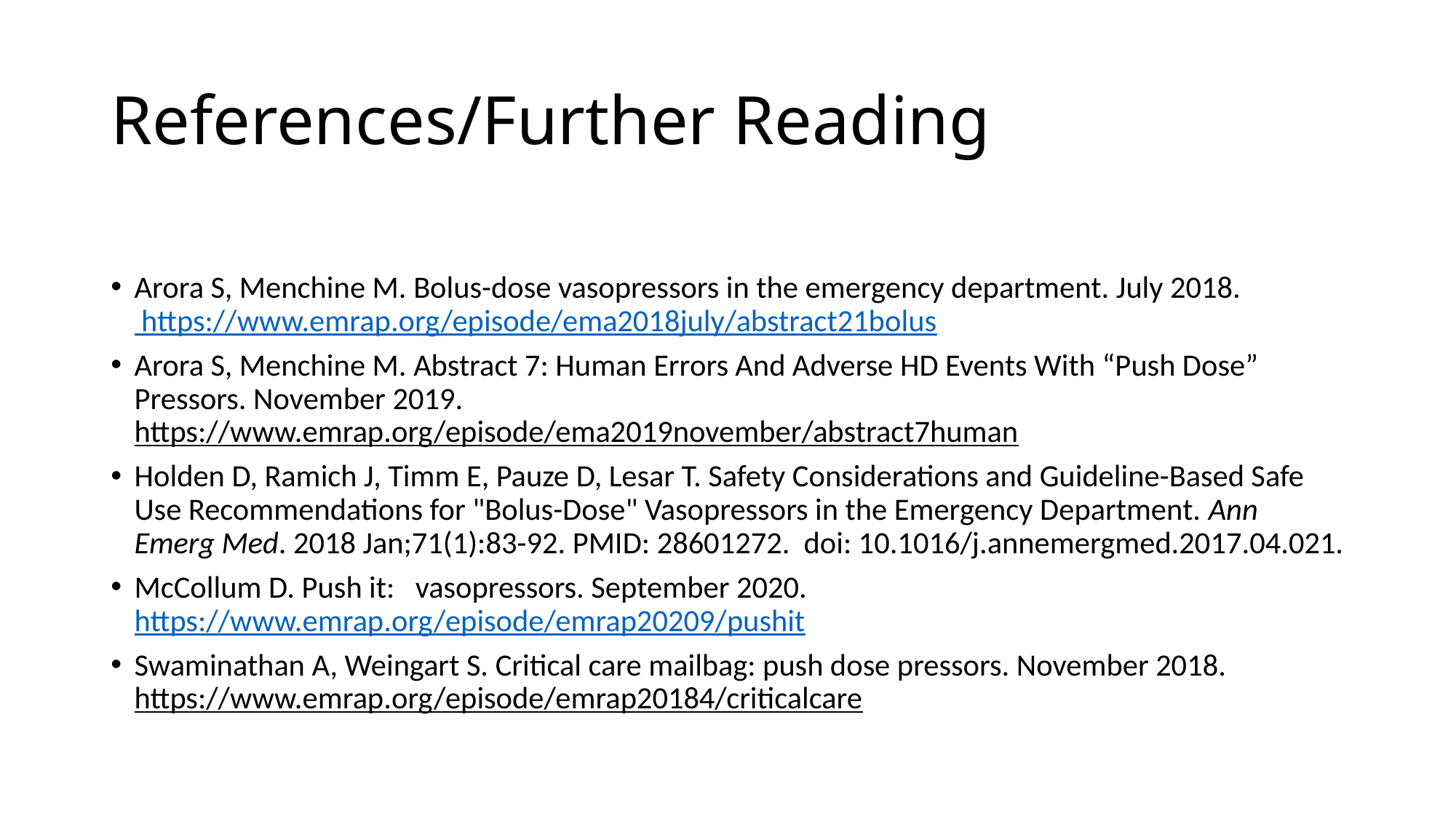

# References/Further Reading
Arora S, Menchine M. Bolus-dose vasopressors in the emergency department. July 2018. https://www.emrap.org/episode/ema2018july/abstract21bolus
Arora S, Menchine M. Abstract 7: Human Errors And Adverse HD Events With “Push Dose” Pressors. November 2019. https://www.emrap.org/episode/ema2019november/abstract7human
Holden D, Ramich J, Timm E, Pauze D, Lesar T. Safety Considerations and Guideline-Based Safe Use Recommendations for "Bolus-Dose" Vasopressors in the Emergency Department. Ann Emerg Med. 2018 Jan;71(1):83-92. PMID: 28601272. doi: 10.1016/j.annemergmed.2017.04.021.
McCollum D. Push it: vasopressors. September 2020. https://www.emrap.org/episode/emrap20209/pushit
Swaminathan A, Weingart S. Critical care mailbag: push dose pressors. November 2018. https://www.emrap.org/episode/emrap20184/criticalcare
