# Supplementary material for: Residents Are Coming: A Faculty Development Curriculum to Prepare a Community Site For New Learners
Source: J Educ Teach Emerg Med. 2022 Jul 15;7(3):C1–C41. doi: 10.21980/J87D2N (PMC10332697; doi:10.21980/J87D2N)
Supplement: Supplementary file 7 — Please see associated PowerPoint file [file jetem-7-3-c1-appendix9.pptx]

## Slide 1
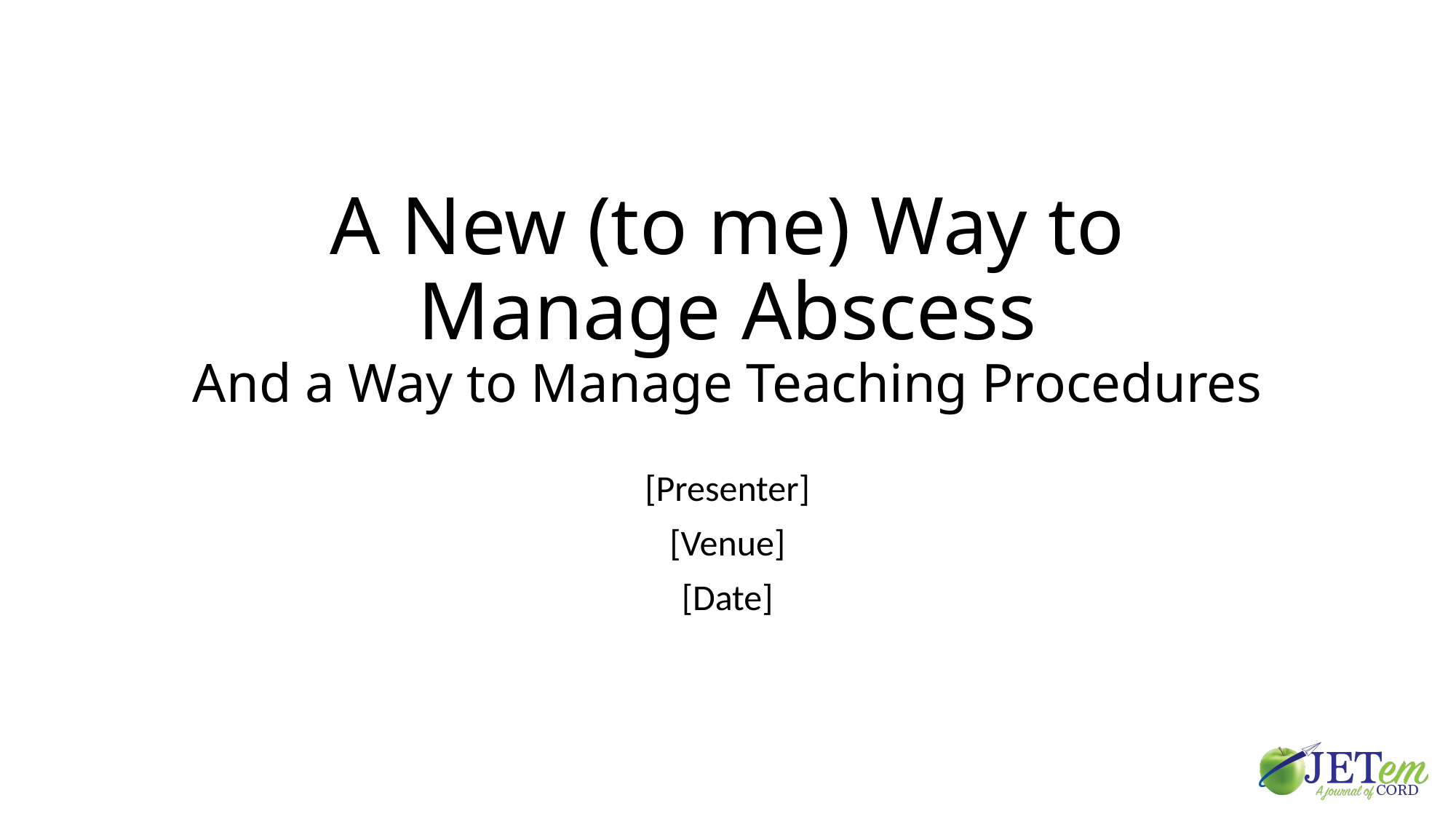

# A New (to me) Way to Manage Abscess And a Way to Manage Teaching Procedures
[Presenter]
[Venue]
[Date]

## Slide 2
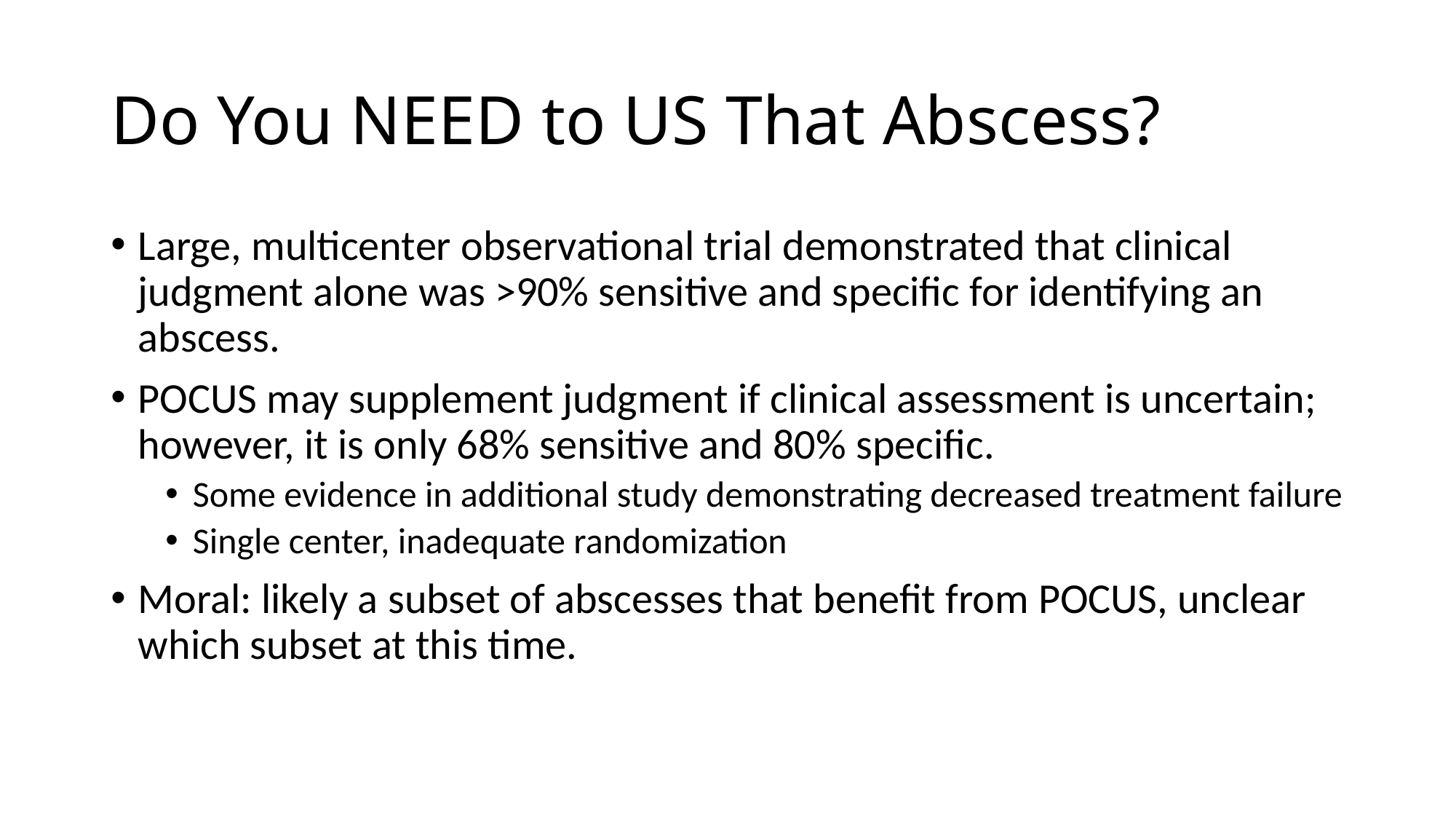

# Do You NEED to US That Abscess?
Large, multicenter observational trial demonstrated that clinical judgment alone was >90% sensitive and specific for identifying an abscess.
POCUS may supplement judgment if clinical assessment is uncertain; however, it is only 68% sensitive and 80% specific.
Some evidence in additional study demonstrating decreased treatment failure
Single center, inadequate randomization
Moral: likely a subset of abscesses that benefit from POCUS, unclear which subset at this time.

## Slide 3
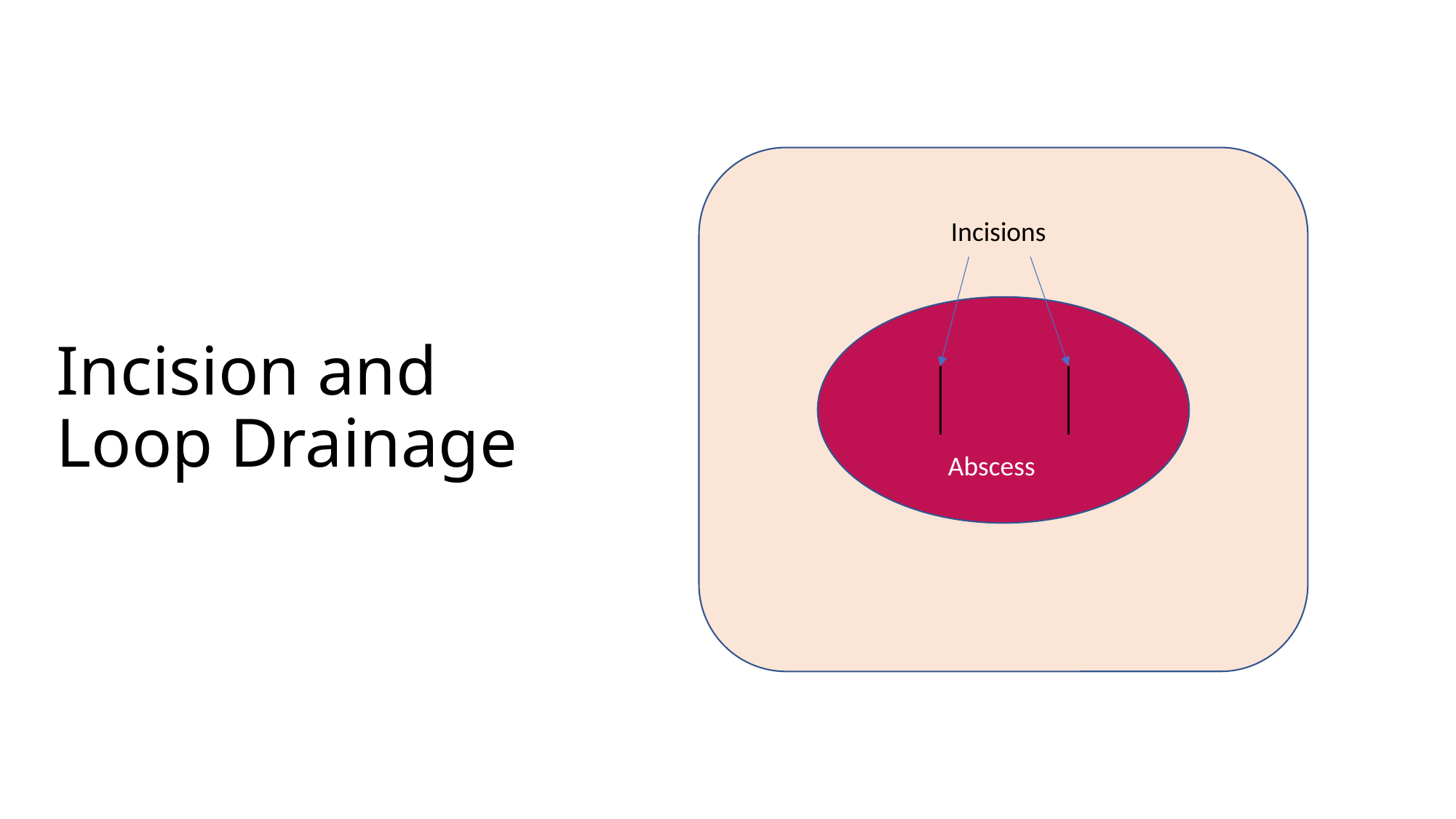

Incisions
# Incision and Loop Drainage
Abscess

## Slide 4
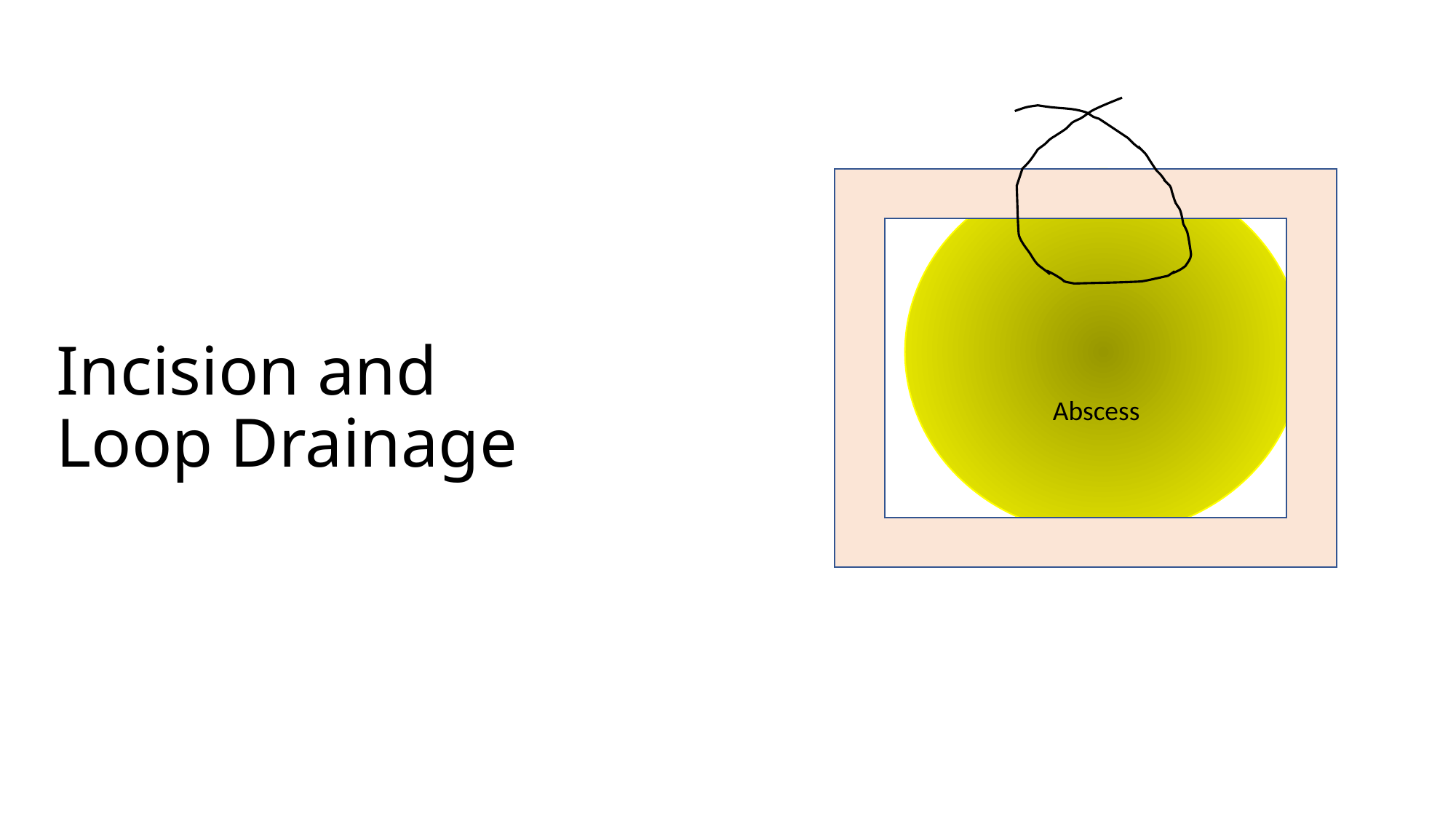

# Incision and Loop Drainage
Abscess

## Slide 5
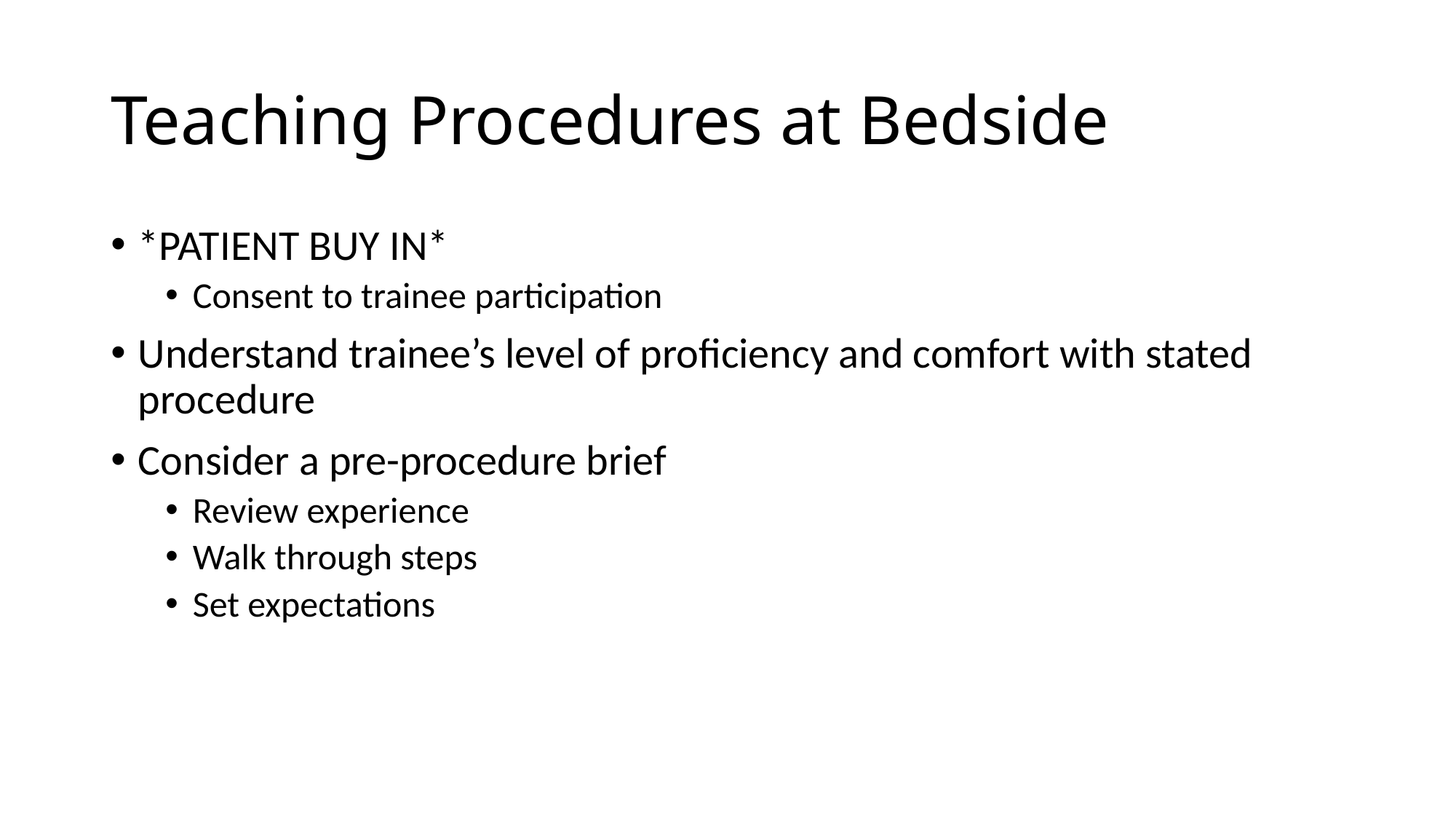

# Teaching Procedures at Bedside
*PATIENT BUY IN*
Consent to trainee participation
Understand trainee’s level of proficiency and comfort with stated procedure
Consider a pre-procedure brief
Review experience
Walk through steps
Set expectations

## Slide 6
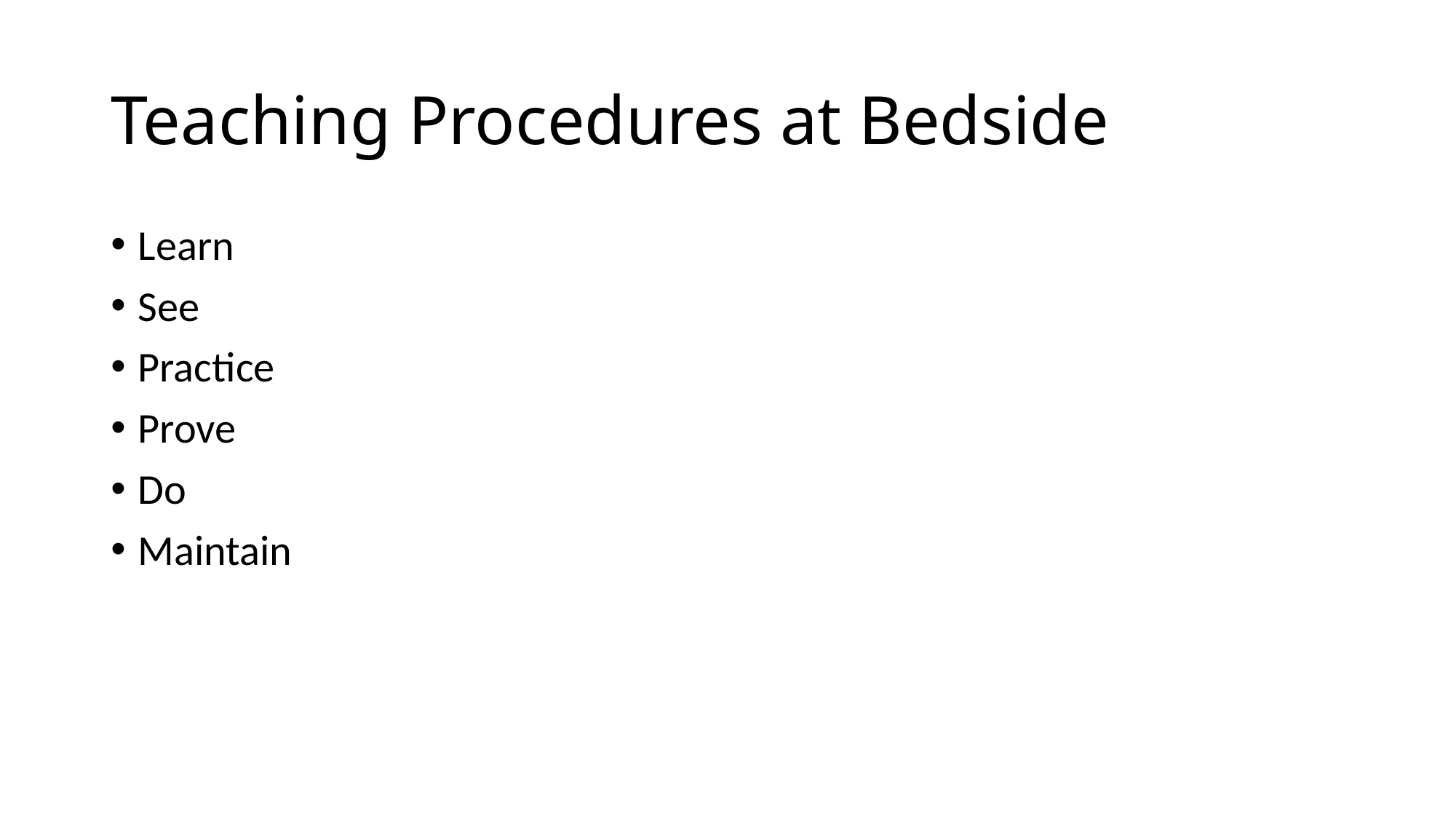

# Teaching Procedures at Bedside
Learn
See
Practice
Prove
Do
Maintain

## Slide 7
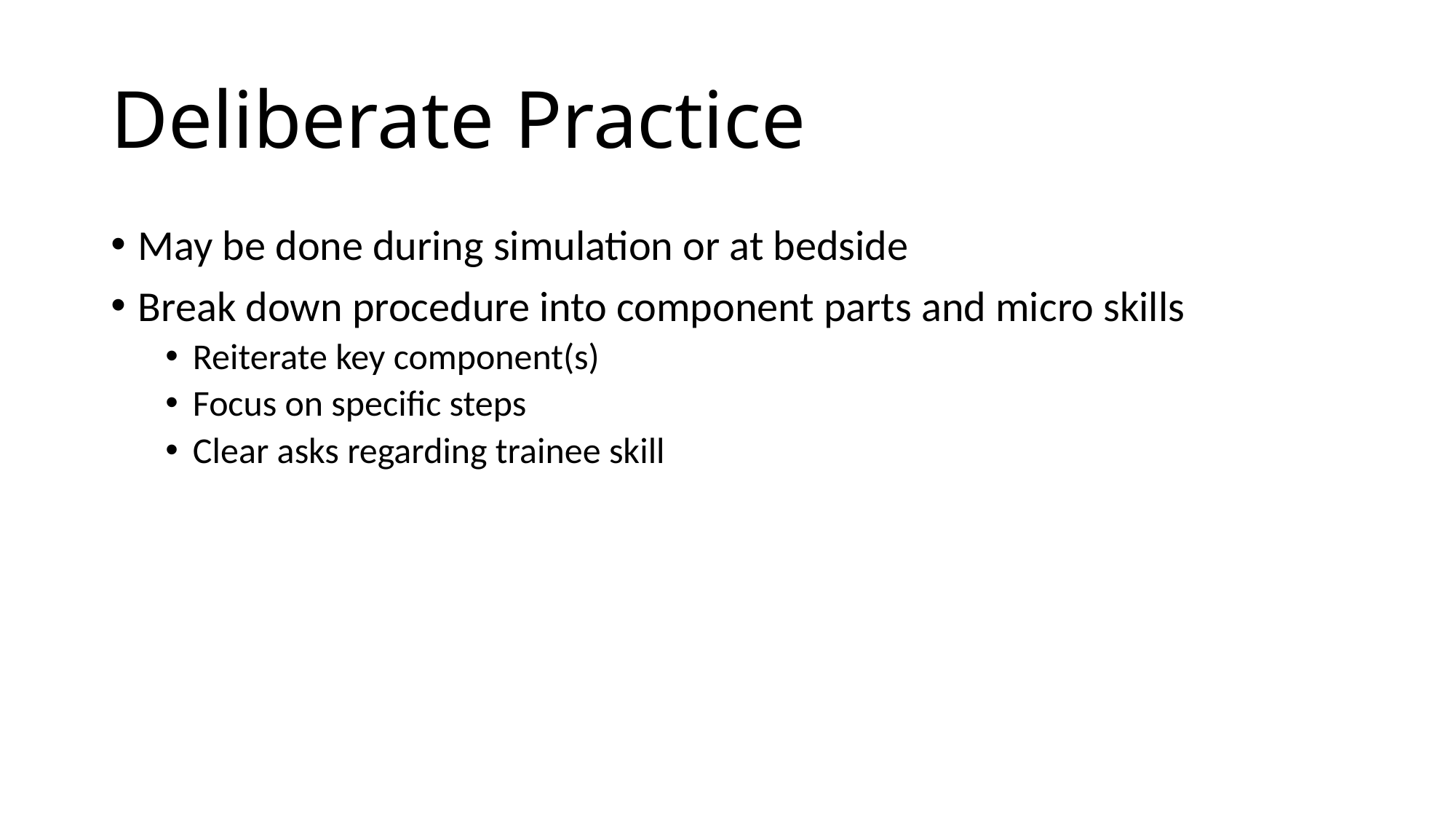

# Deliberate Practice
May be done during simulation or at bedside
Break down procedure into component parts and micro skills
Reiterate key component(s)
Focus on specific steps
Clear asks regarding trainee skill

## Slide 8
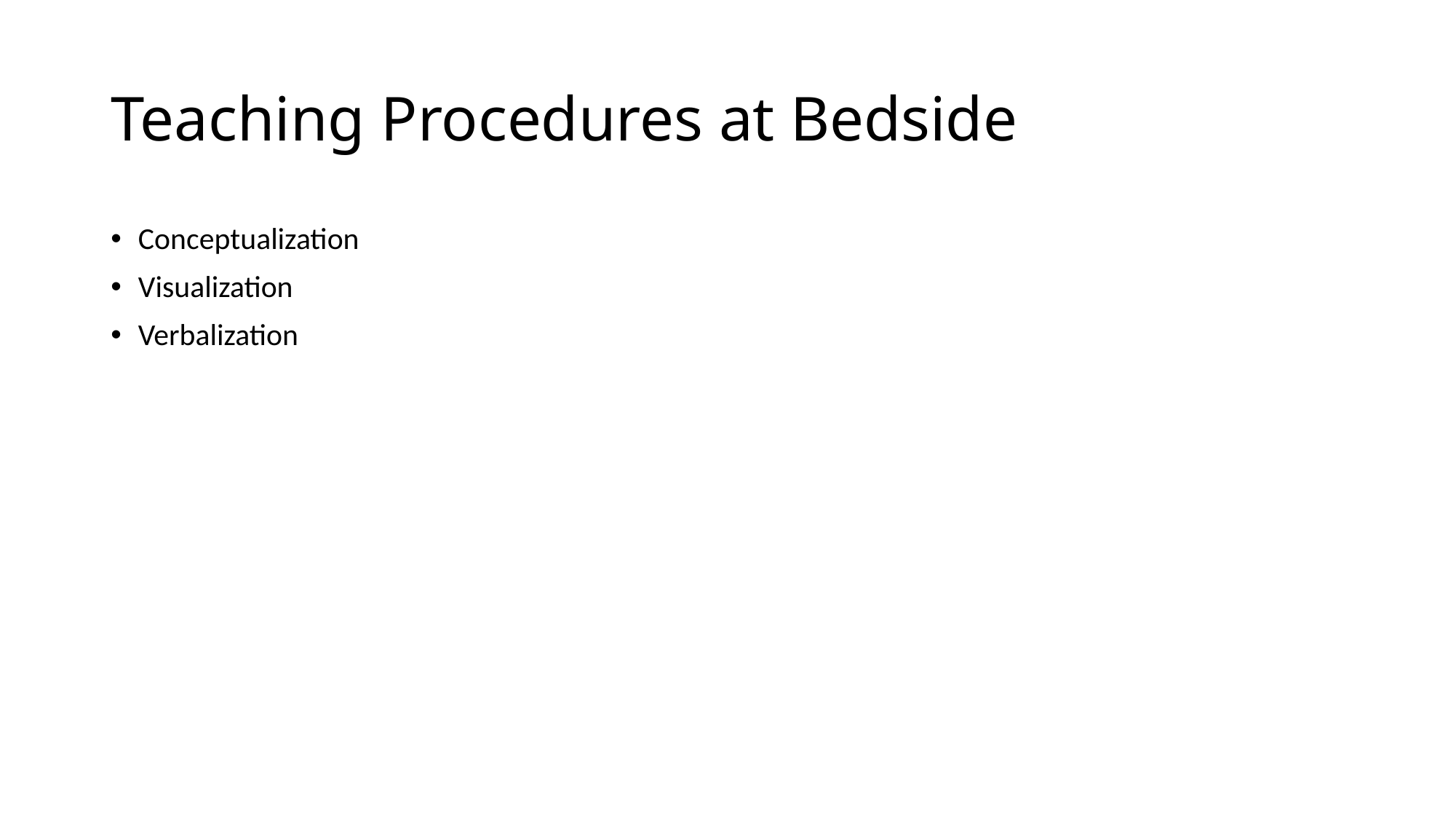

# Teaching Procedures at Bedside
Conceptualization
Visualization
Verbalization

## Slide 9
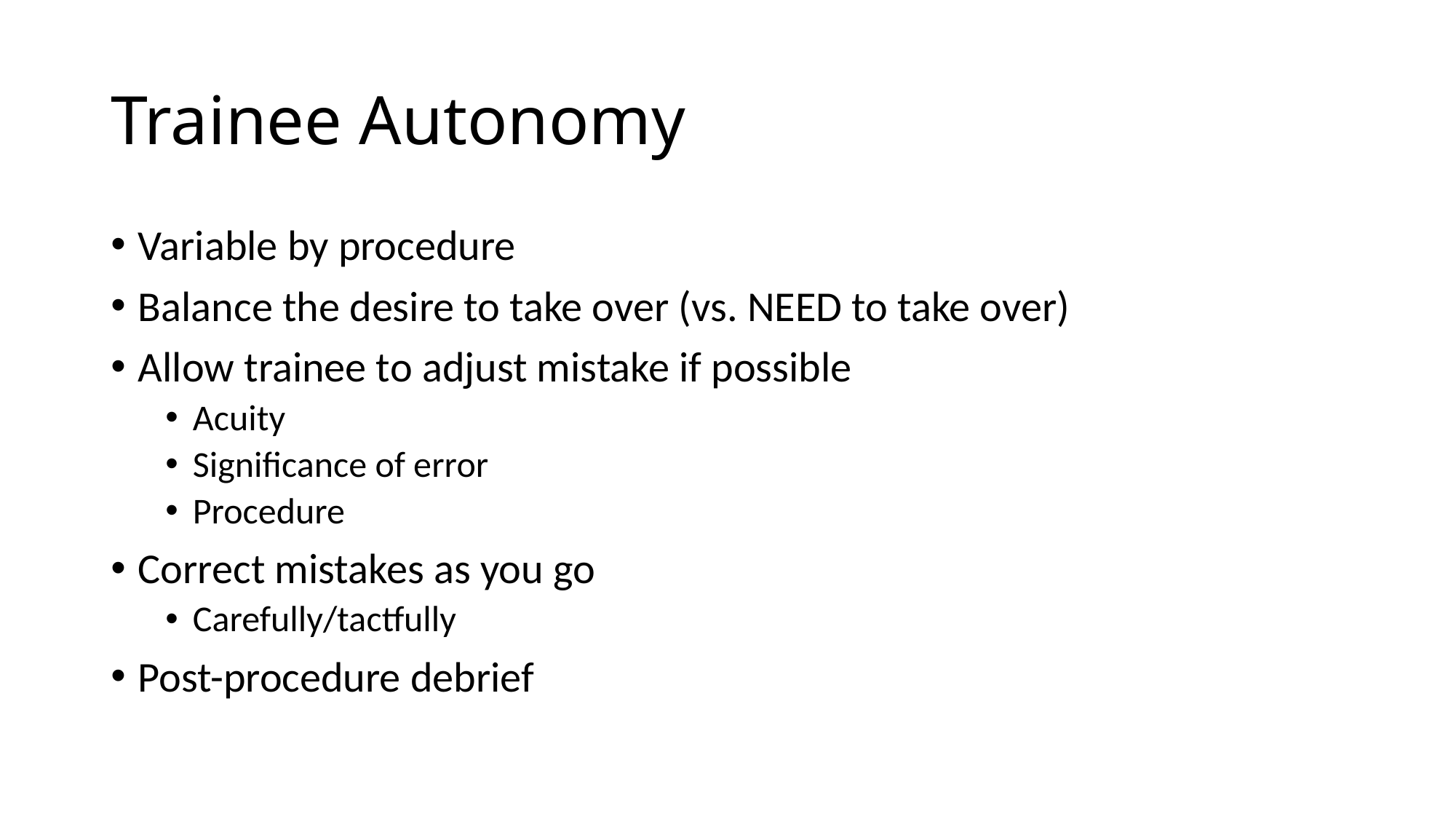

# Trainee Autonomy
Variable by procedure
Balance the desire to take over (vs. NEED to take over)
Allow trainee to adjust mistake if possible
Acuity
Significance of error
Procedure
Correct mistakes as you go
Carefully/tactfully
Post-procedure debrief

## Slide 10
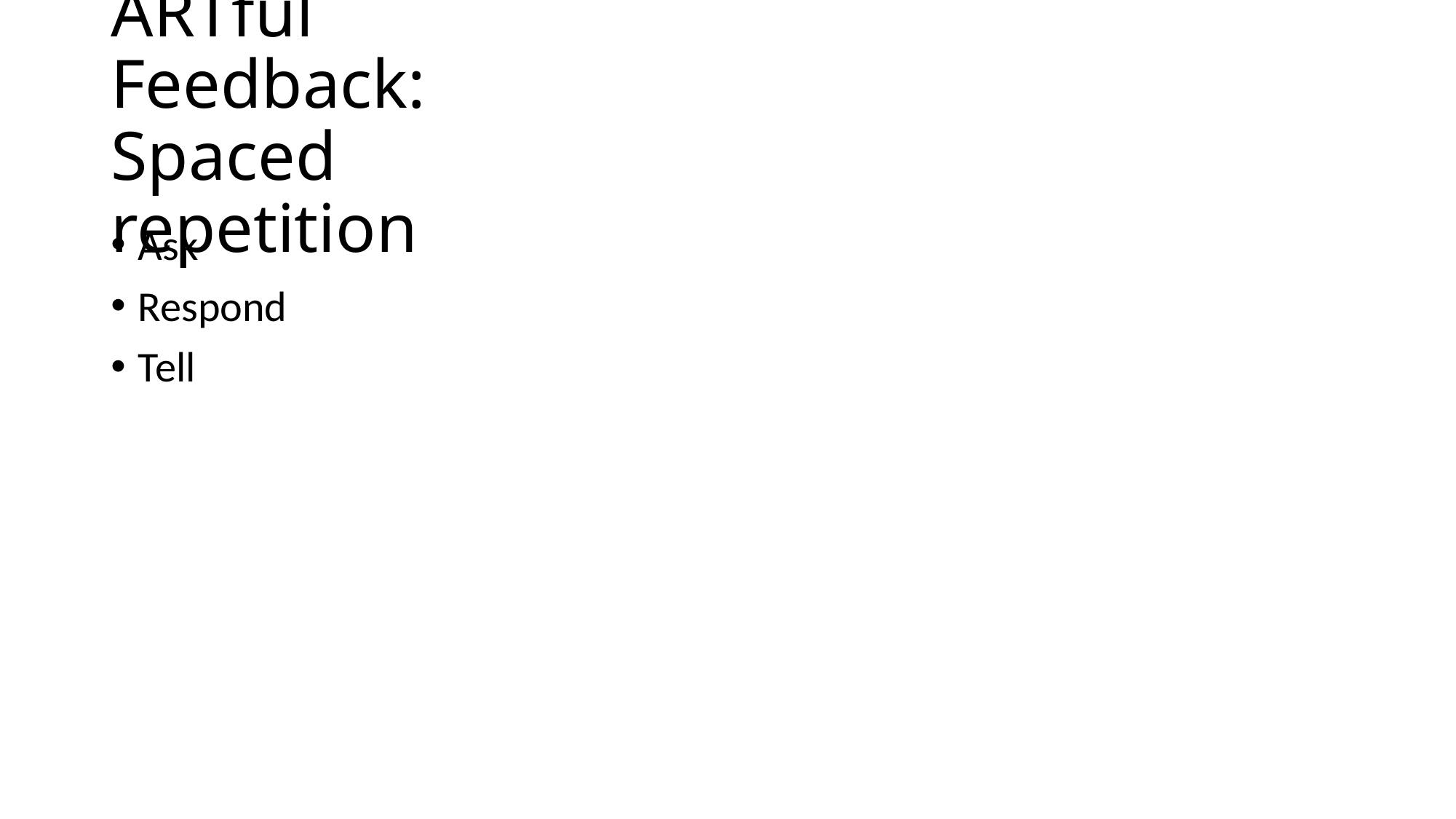

# ARTful Feedback: Spaced repetition
Ask
Respond
Tell

## Slide 11
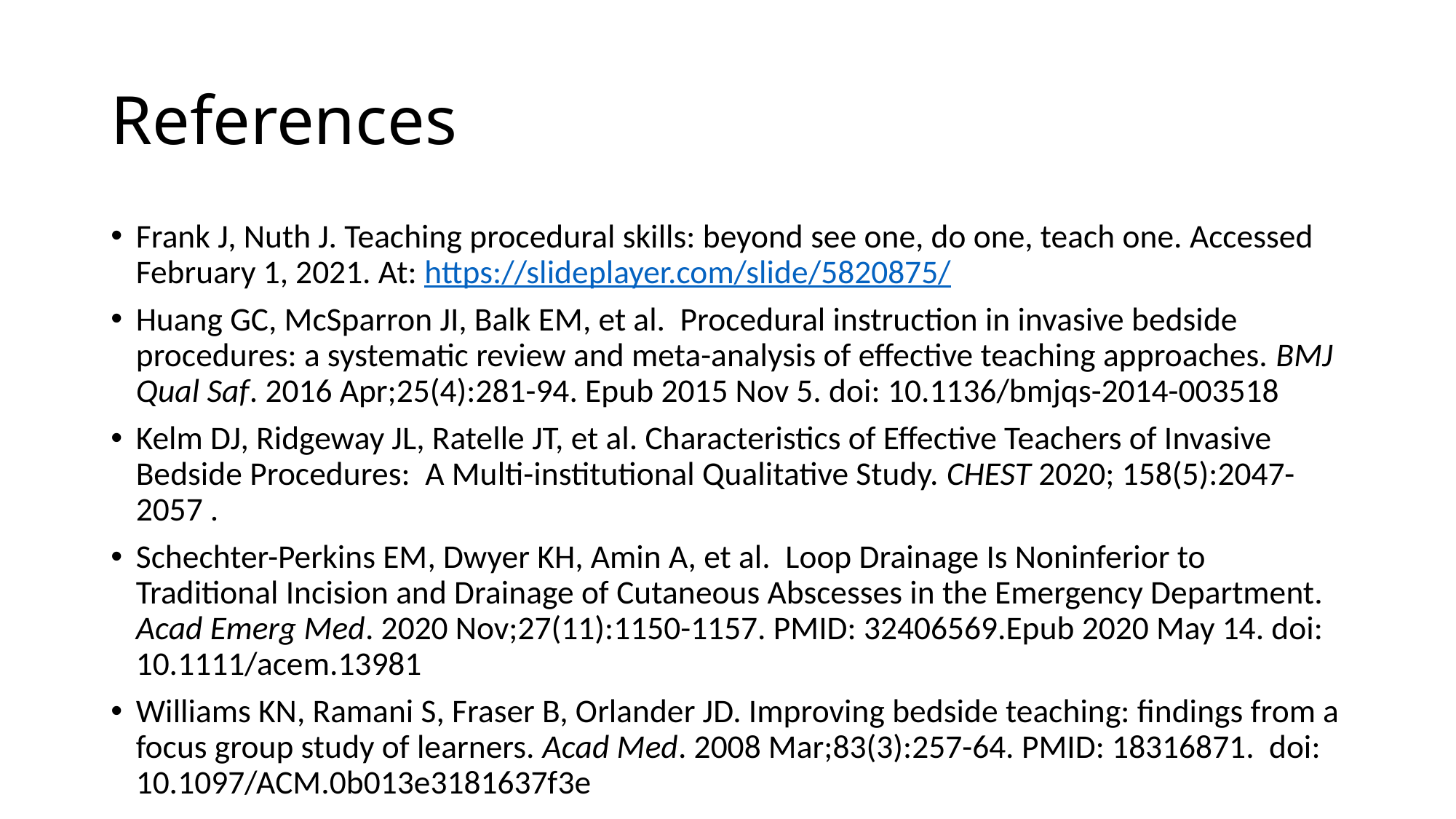

# References
Frank J, Nuth J. Teaching procedural skills: beyond see one, do one, teach one. Accessed February 1, 2021. At: https://slideplayer.com/slide/5820875/
Huang GC, McSparron JI, Balk EM, et al. Procedural instruction in invasive bedside procedures: a systematic review and meta-analysis of effective teaching approaches. BMJ Qual Saf. 2016 Apr;25(4):281-94. Epub 2015 Nov 5. doi: 10.1136/bmjqs-2014-003518
Kelm DJ, Ridgeway JL, Ratelle JT, et al. Characteristics of Effective Teachers of Invasive Bedside Procedures: A Multi-institutional Qualitative Study. CHEST 2020; 158(5):2047-2057 .
Schechter-Perkins EM, Dwyer KH, Amin A, et al. Loop Drainage Is Noninferior to Traditional Incision and Drainage of Cutaneous Abscesses in the Emergency Department. Acad Emerg Med. 2020 Nov;27(11):1150-1157. PMID: 32406569.Epub 2020 May 14. doi: 10.1111/acem.13981
Williams KN, Ramani S, Fraser B, Orlander JD. Improving bedside teaching: findings from a focus group study of learners. Acad Med. 2008 Mar;83(3):257-64. PMID: 18316871. doi: 10.1097/ACM.0b013e3181637f3e

## Slide 12
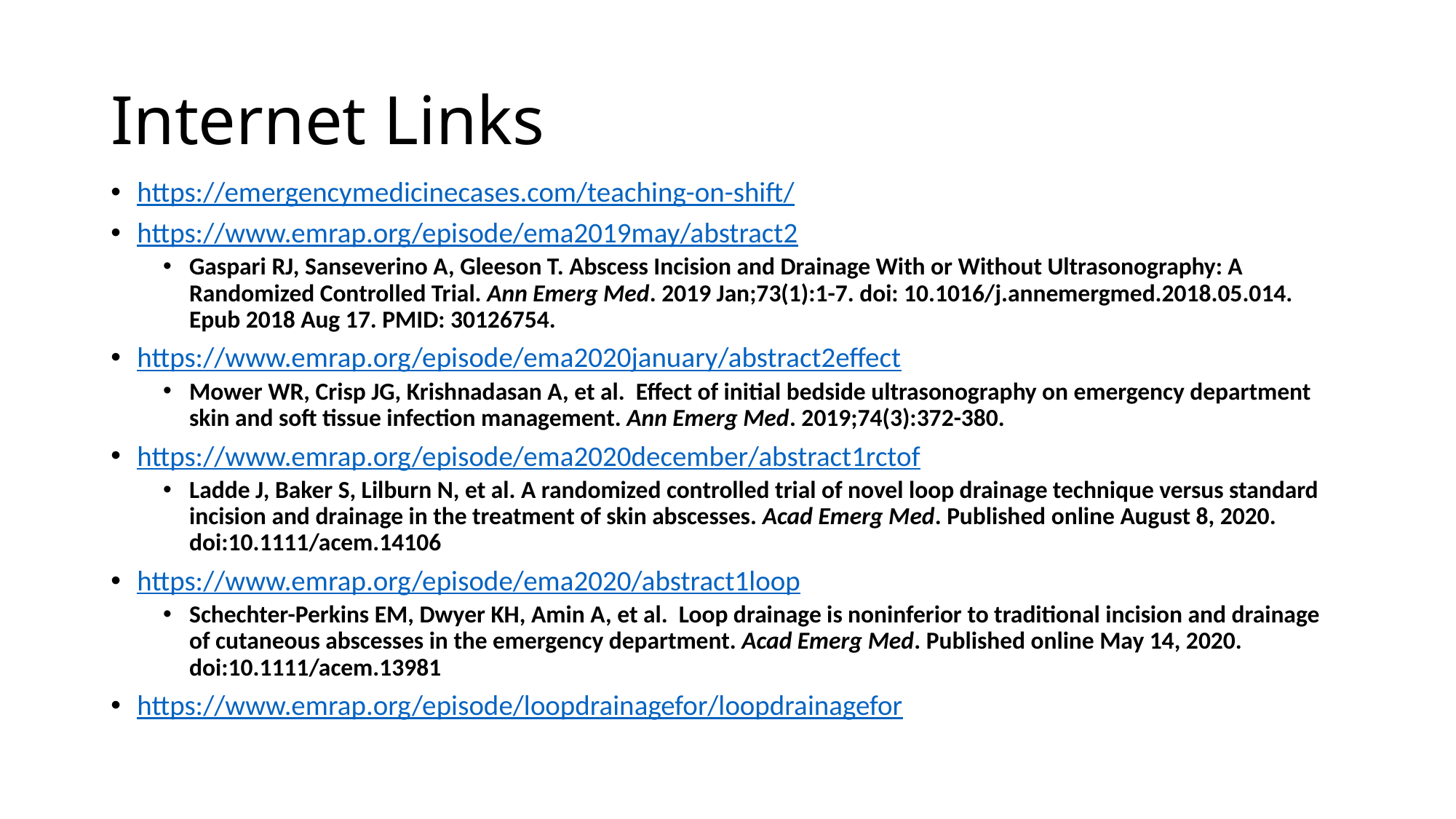

# Internet Links
https://emergencymedicinecases.com/teaching-on-shift/
https://www.emrap.org/episode/ema2019may/abstract2
Gaspari RJ, Sanseverino A, Gleeson T. Abscess Incision and Drainage With or Without Ultrasonography: A Randomized Controlled Trial. Ann Emerg Med. 2019 Jan;73(1):1-7. doi: 10.1016/j.annemergmed.2018.05.014. Epub 2018 Aug 17. PMID: 30126754.
https://www.emrap.org/episode/ema2020january/abstract2effect
Mower WR, Crisp JG, Krishnadasan A, et al. Effect of initial bedside ultrasonography on emergency department skin and soft tissue infection management. Ann Emerg Med. 2019;74(3):372-380.
https://www.emrap.org/episode/ema2020december/abstract1rctof
Ladde J, Baker S, Lilburn N, et al. A randomized controlled trial of novel loop drainage technique versus standard incision and drainage in the treatment of skin abscesses. Acad Emerg Med. Published online August 8, 2020. doi:10.1111/acem.14106
https://www.emrap.org/episode/ema2020/abstract1loop
Schechter-Perkins EM, Dwyer KH, Amin A, et al. Loop drainage is noninferior to traditional incision and drainage of cutaneous abscesses in the emergency department. Acad Emerg Med. Published online May 14, 2020. doi:10.1111/acem.13981
https://www.emrap.org/episode/loopdrainagefor/loopdrainagefor
