# Supplementary material for: Residents Are Coming: A Faculty Development Curriculum to Prepare a Community Site For New Learners
Source: J Educ Teach Emerg Med. 2022 Jul 15;7(3):C1–C41. doi: 10.21980/J87D2N (PMC10332697; doi:10.21980/J87D2N)
Supplement: Supplementary file 8 — Please see associated PowerPoint file [file jetem-7-3-c1-appendix10.pptx]

## Slide 1
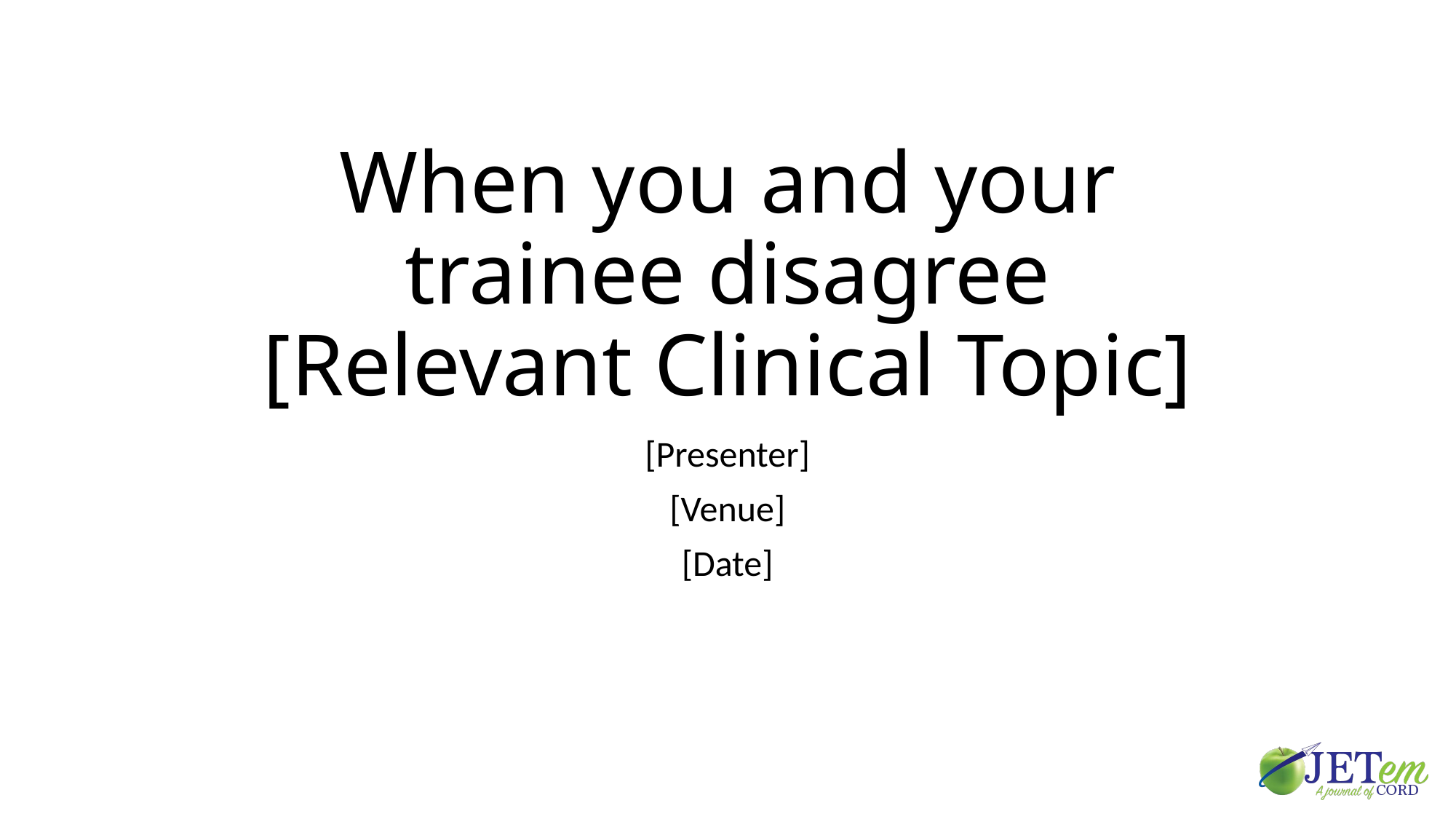

# When you and your trainee disagree [Relevant Clinical Topic]
[Presenter]
[Venue]
[Date]

## Slide 2
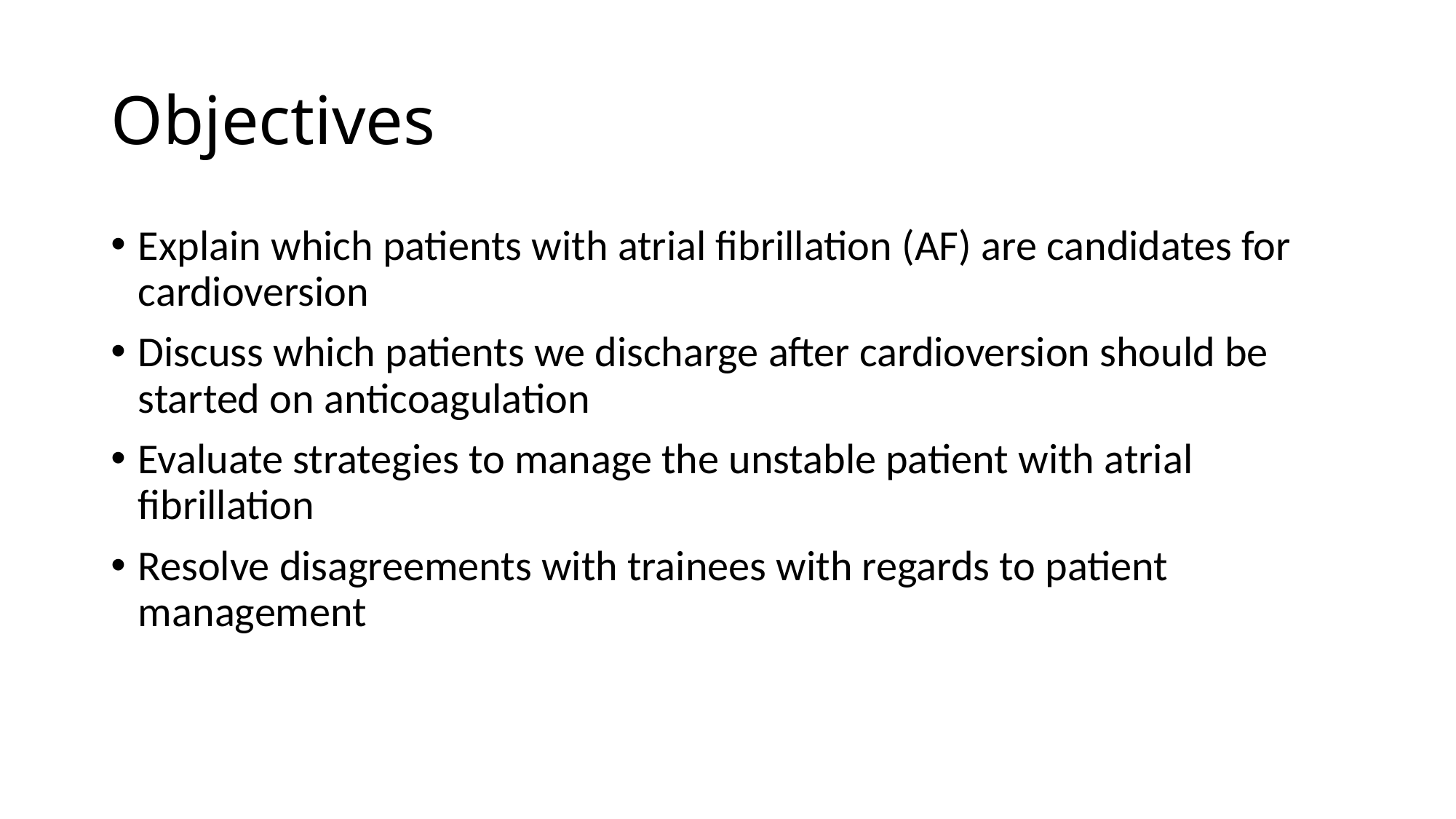

# Objectives
Explain which patients with atrial fibrillation (AF) are candidates for cardioversion
Discuss which patients we discharge after cardioversion should be started on anticoagulation
Evaluate strategies to manage the unstable patient with atrial fibrillation
Resolve disagreements with trainees with regards to patient management

## Slide 3
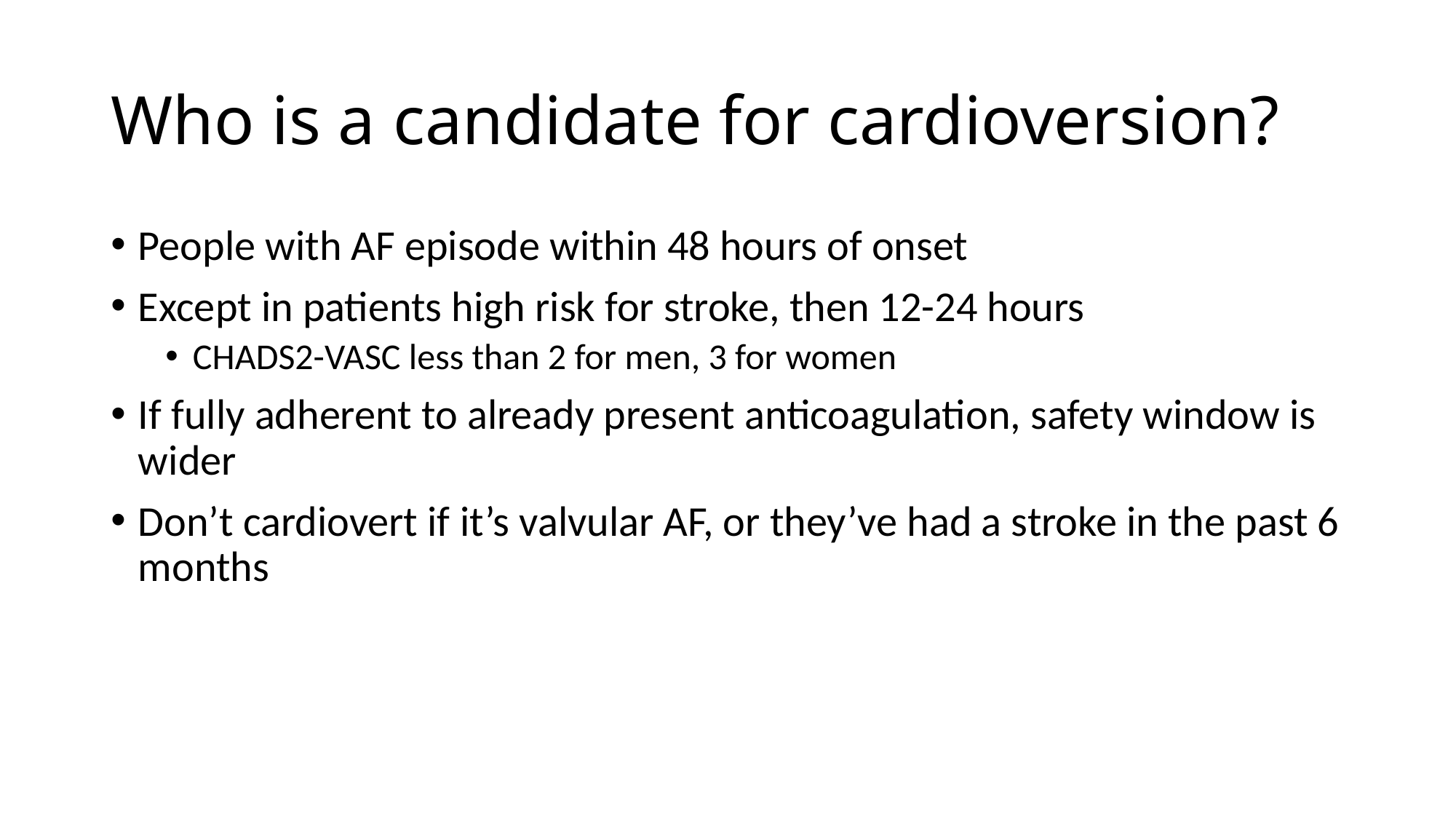

# Who is a candidate for cardioversion?
People with AF episode within 48 hours of onset
Except in patients high risk for stroke, then 12-24 hours
CHADS2-VASC less than 2 for men, 3 for women
If fully adherent to already present anticoagulation, safety window is wider
Don’t cardiovert if it’s valvular AF, or they’ve had a stroke in the past 6 months

## Slide 4
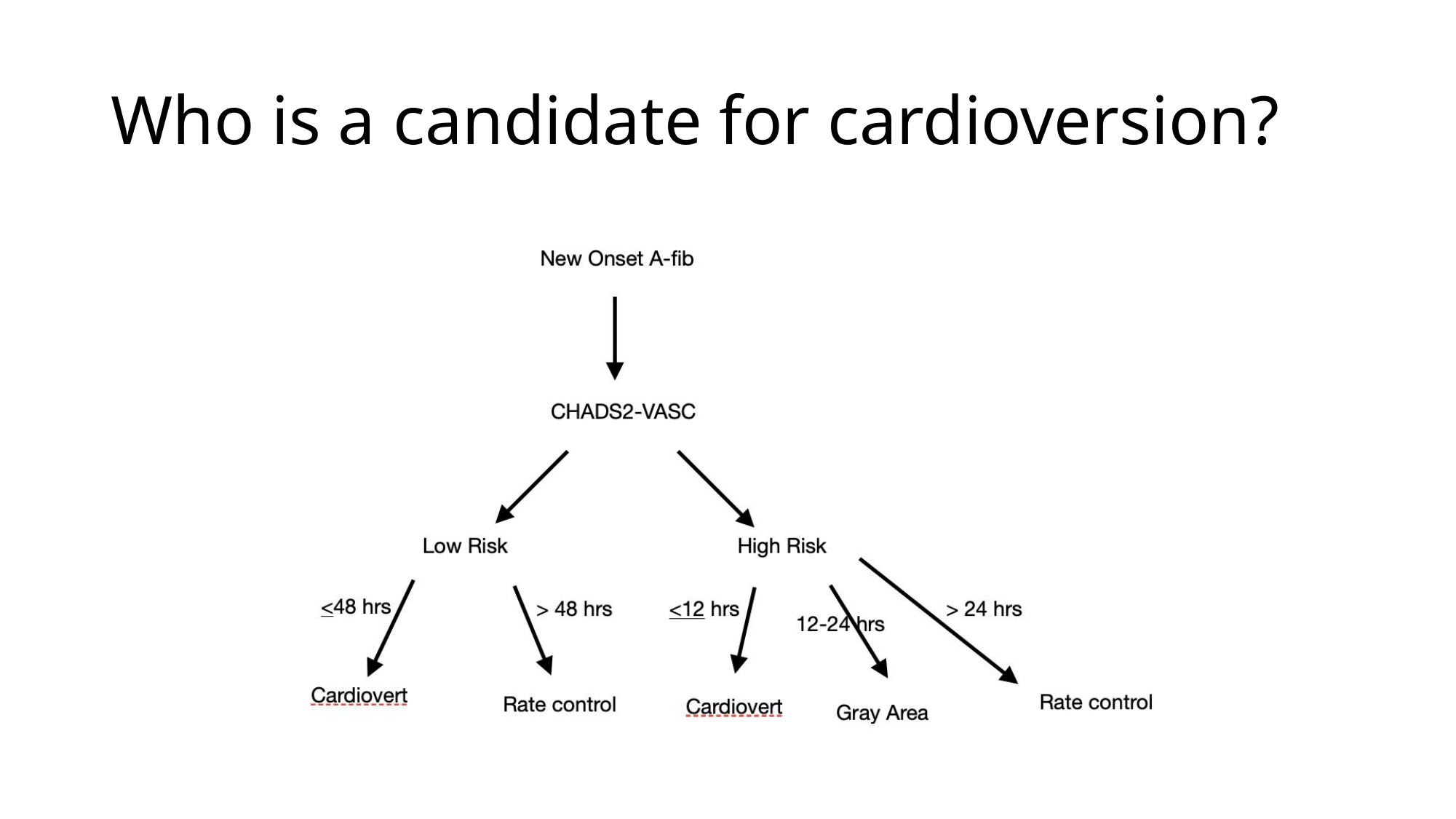

# Who is a candidate for cardioversion?

## Slide 5
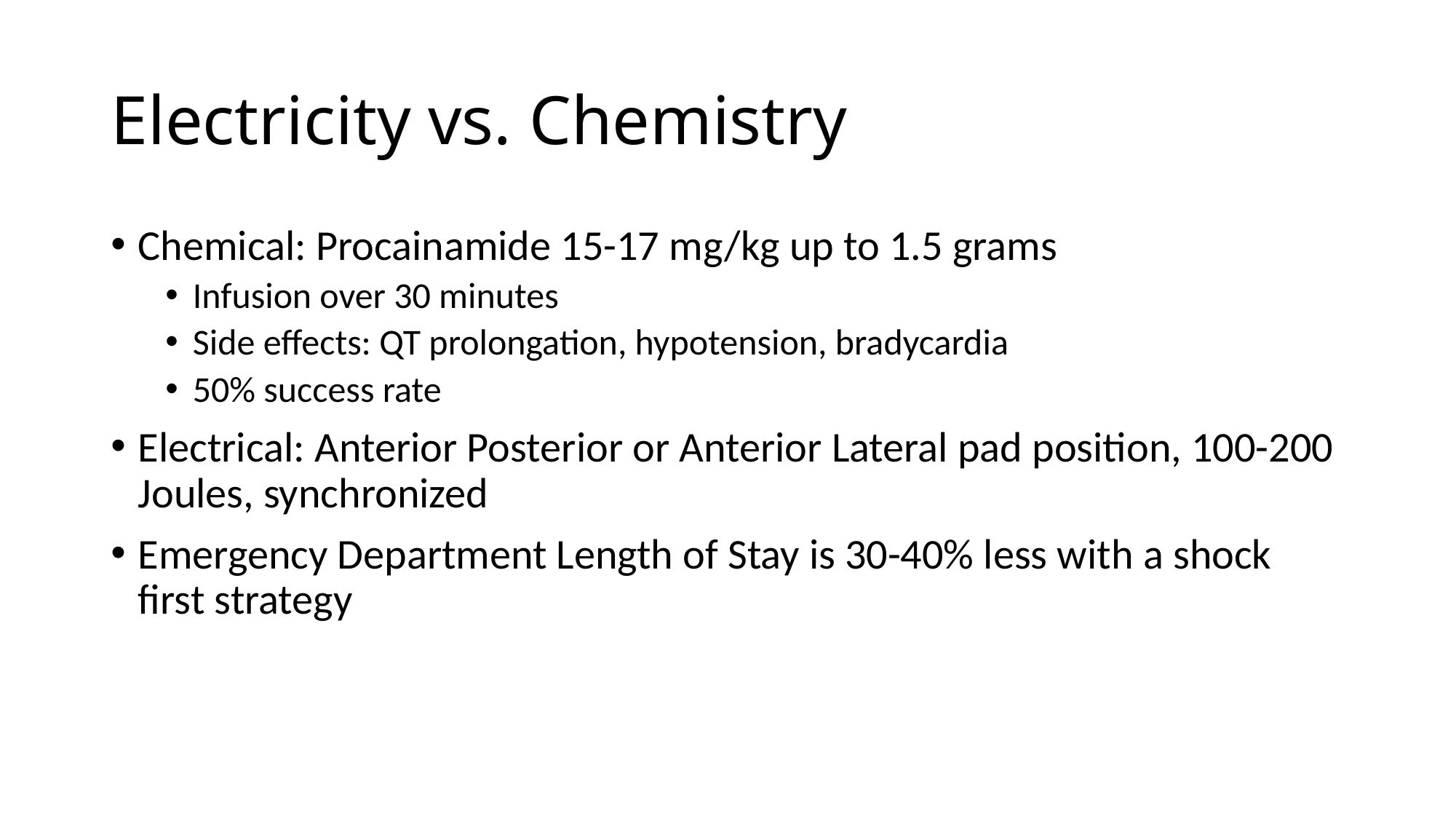

# Electricity vs. Chemistry
Chemical: Procainamide 15-17 mg/kg up to 1.5 grams
Infusion over 30 minutes
Side effects: QT prolongation, hypotension, bradycardia
50% success rate
Electrical: Anterior Posterior or Anterior Lateral pad position, 100-200 Joules, synchronized
Emergency Department Length of Stay is 30-40% less with a shock first strategy

## Slide 6
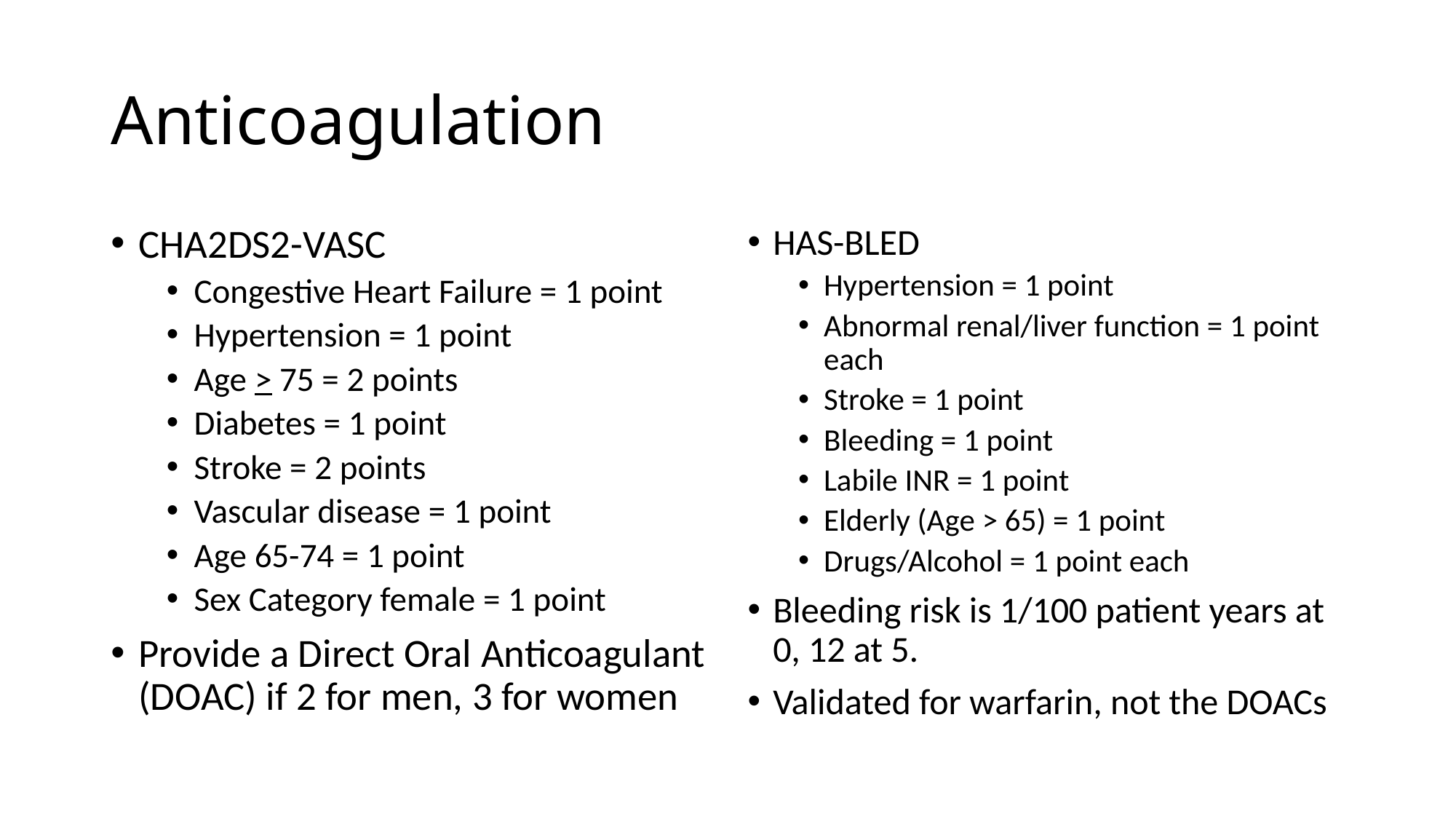

# Anticoagulation
CHA2DS2-VASC
Congestive Heart Failure = 1 point
Hypertension = 1 point
Age > 75 = 2 points
Diabetes = 1 point
Stroke = 2 points
Vascular disease = 1 point
Age 65-74 = 1 point
Sex Category female = 1 point
Provide a Direct Oral Anticoagulant (DOAC) if 2 for men, 3 for women
HAS-BLED
Hypertension = 1 point
Abnormal renal/liver function = 1 point each
Stroke = 1 point
Bleeding = 1 point
Labile INR = 1 point
Elderly (Age > 65) = 1 point
Drugs/Alcohol = 1 point each
Bleeding risk is 1/100 patient years at 0, 12 at 5.
Validated for warfarin, not the DOACs

## Slide 7
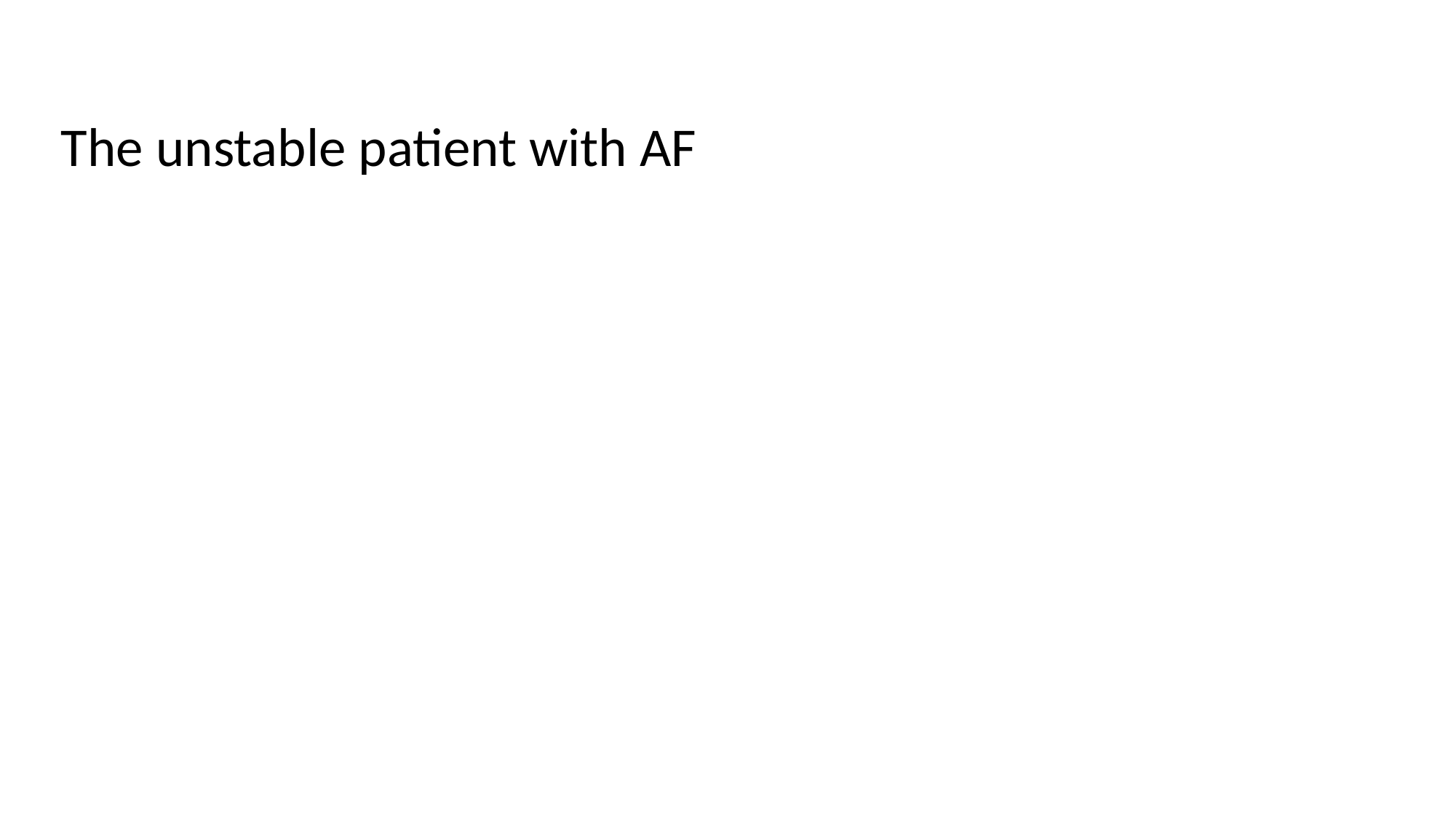

The unstable patient with AF
NOT SURE IF UNSTABLE BECAUSE OF AFIB
OR IN AFIB BECAUSE UNSTABLE

## Slide 8
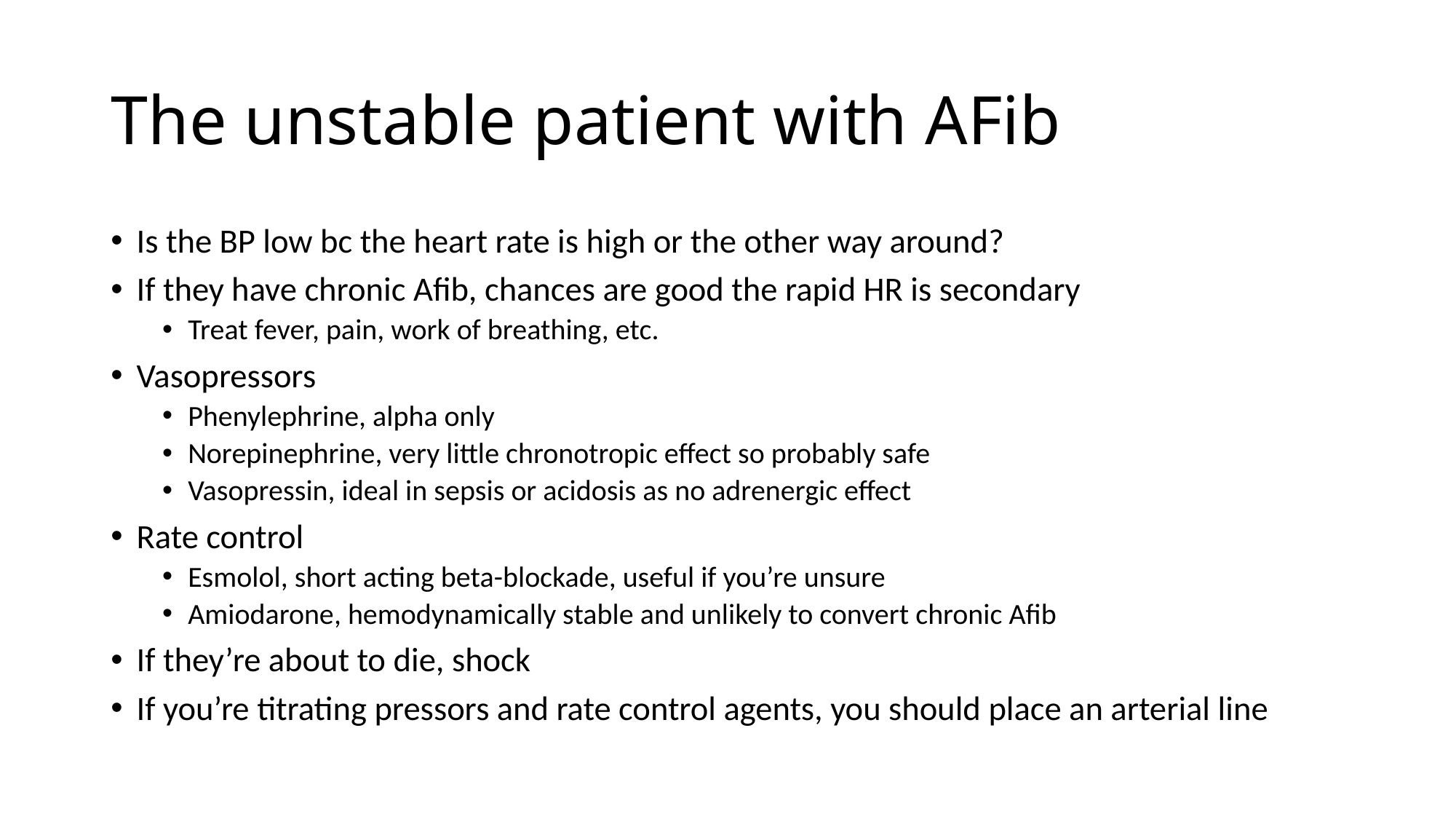

# The unstable patient with AFib
Is the BP low bc the heart rate is high or the other way around?
If they have chronic Afib, chances are good the rapid HR is secondary
Treat fever, pain, work of breathing, etc.
Vasopressors
Phenylephrine, alpha only
Norepinephrine, very little chronotropic effect so probably safe
Vasopressin, ideal in sepsis or acidosis as no adrenergic effect
Rate control
Esmolol, short acting beta-blockade, useful if you’re unsure
Amiodarone, hemodynamically stable and unlikely to convert chronic Afib
If they’re about to die, shock
If you’re titrating pressors and rate control agents, you should place an arterial line

## Slide 9
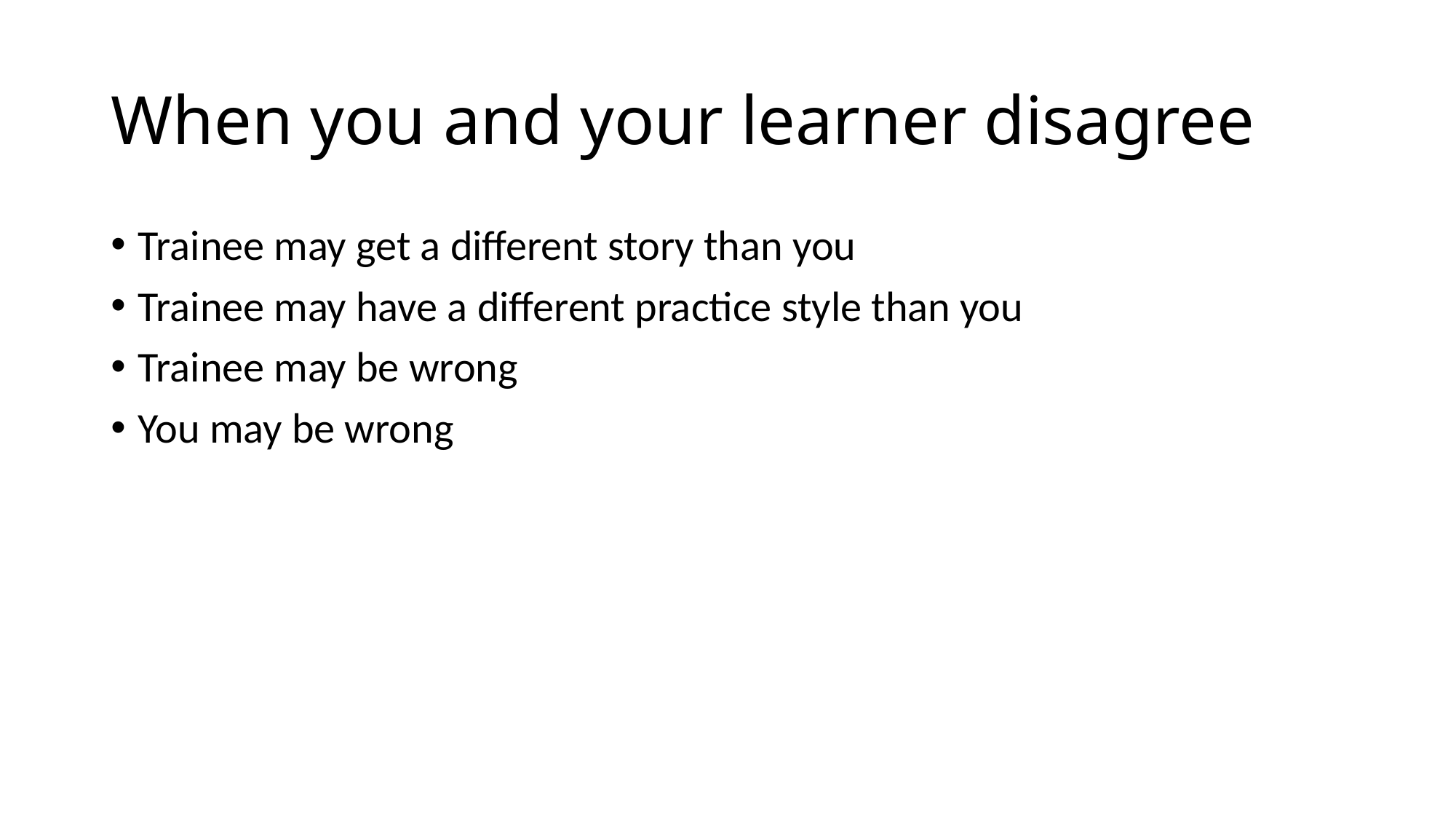

# When you and your learner disagree
Trainee may get a different story than you
Trainee may have a different practice style than you
Trainee may be wrong
You may be wrong

## Slide 10
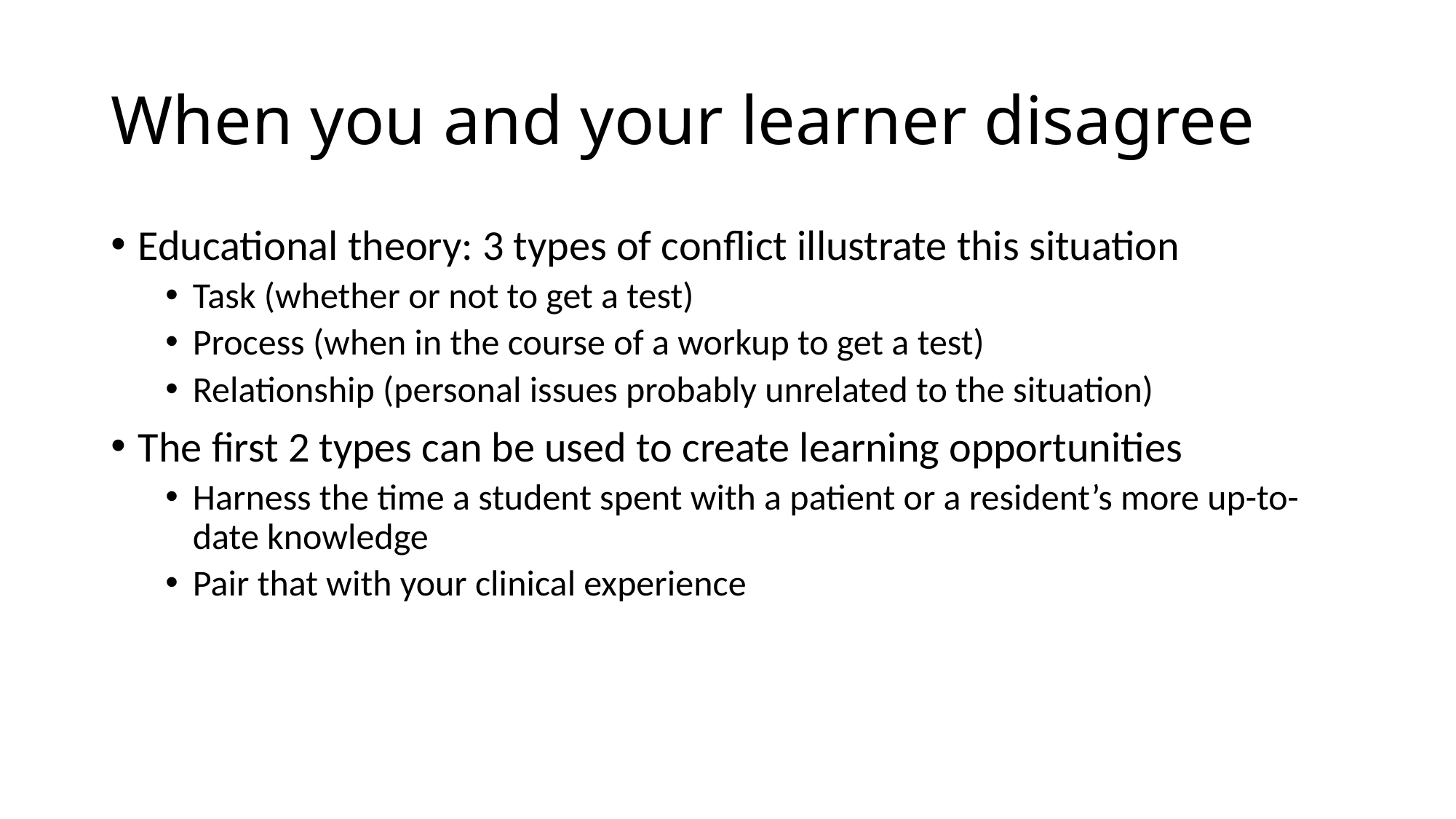

# When you and your learner disagree
Educational theory: 3 types of conflict illustrate this situation
Task (whether or not to get a test)
Process (when in the course of a workup to get a test)
Relationship (personal issues probably unrelated to the situation)
The first 2 types can be used to create learning opportunities
Harness the time a student spent with a patient or a resident’s more up-to-date knowledge
Pair that with your clinical experience

## Slide 11
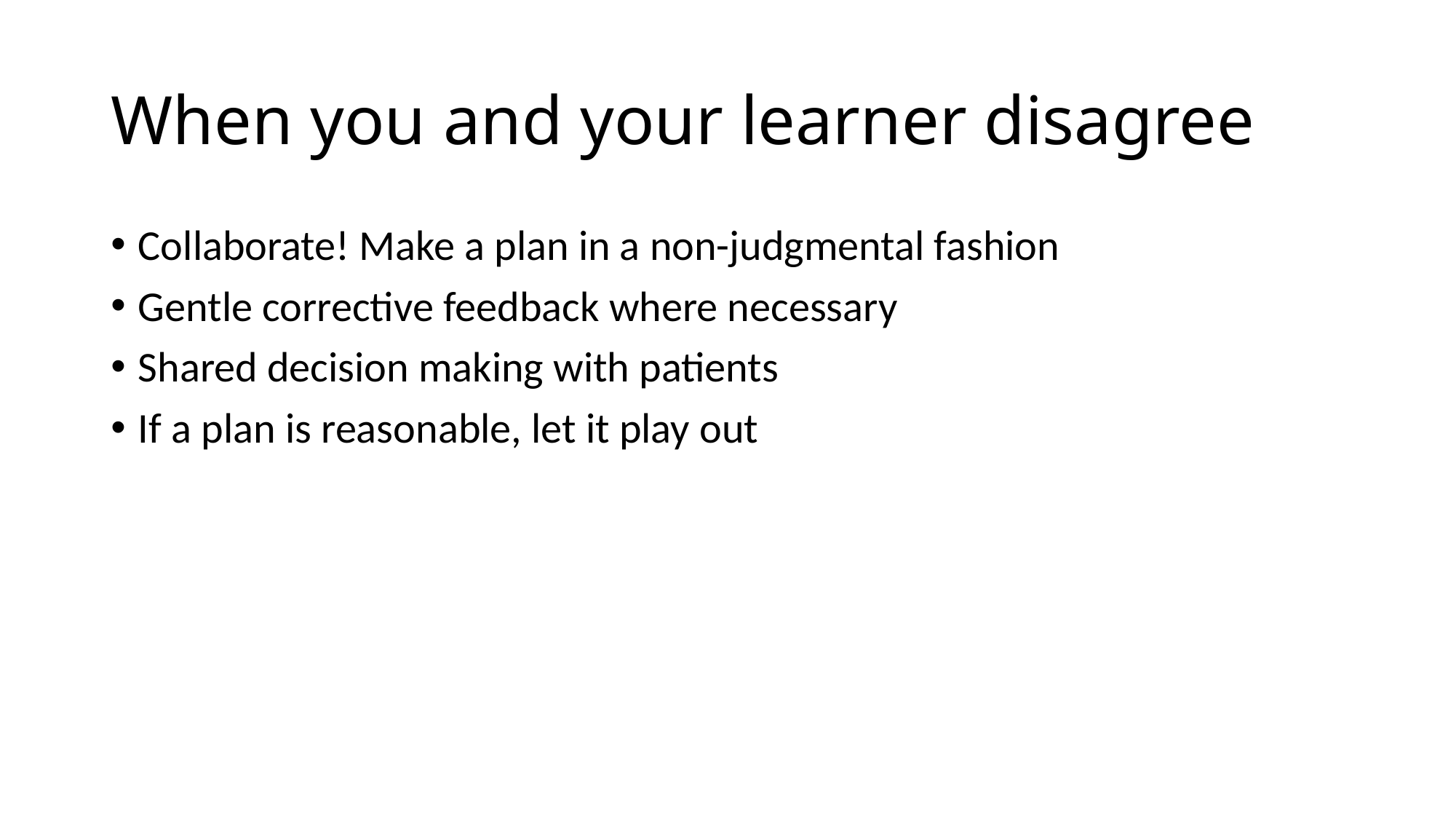

# When you and your learner disagree
Collaborate! Make a plan in a non-judgmental fashion
Gentle corrective feedback where necessary
Shared decision making with patients
If a plan is reasonable, let it play out

## Slide 12
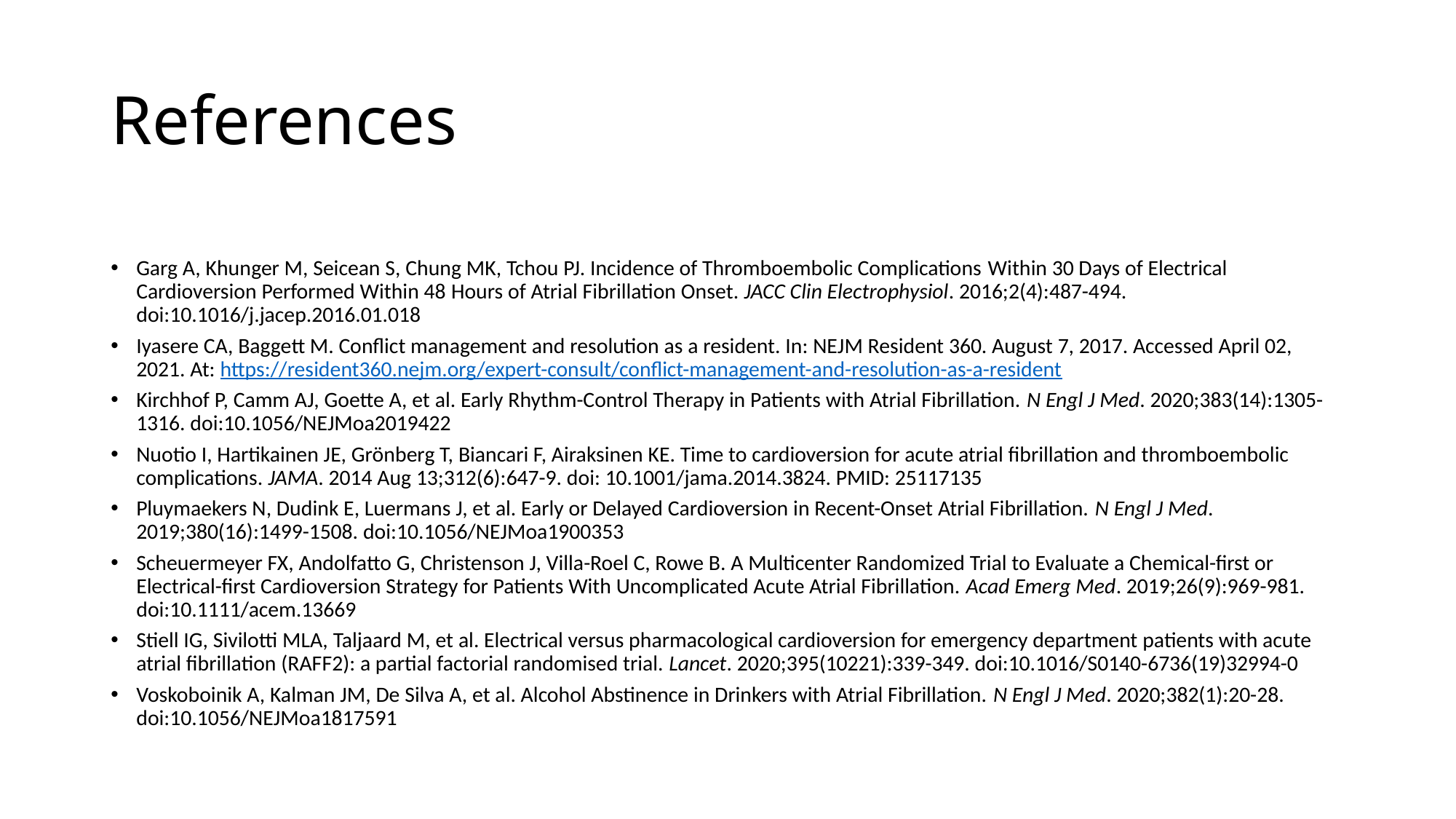

# References
Garg A, Khunger M, Seicean S, Chung MK, Tchou PJ. Incidence of Thromboembolic Complications Within 30 Days of Electrical Cardioversion Performed Within 48 Hours of Atrial Fibrillation Onset. JACC Clin Electrophysiol. 2016;2(4):487-494. doi:10.1016/j.jacep.2016.01.018
Iyasere CA, Baggett M. Conflict management and resolution as a resident. In: NEJM Resident 360. August 7, 2017. Accessed April 02, 2021. At: https://resident360.nejm.org/expert-consult/conflict-management-and-resolution-as-a-resident
Kirchhof P, Camm AJ, Goette A, et al. Early Rhythm-Control Therapy in Patients with Atrial Fibrillation. N Engl J Med. 2020;383(14):1305-1316. doi:10.1056/NEJMoa2019422
Nuotio I, Hartikainen JE, Grönberg T, Biancari F, Airaksinen KE. Time to cardioversion for acute atrial fibrillation and thromboembolic complications. JAMA. 2014 Aug 13;312(6):647-9. doi: 10.1001/jama.2014.3824. PMID: 25117135
Pluymaekers N, Dudink E, Luermans J, et al. Early or Delayed Cardioversion in Recent-Onset Atrial Fibrillation. N Engl J Med. 2019;380(16):1499-1508. doi:10.1056/NEJMoa1900353
Scheuermeyer FX, Andolfatto G, Christenson J, Villa-Roel C, Rowe B. A Multicenter Randomized Trial to Evaluate a Chemical-first or Electrical-first Cardioversion Strategy for Patients With Uncomplicated Acute Atrial Fibrillation. Acad Emerg Med. 2019;26(9):969-981. doi:10.1111/acem.13669
Stiell IG, Sivilotti MLA, Taljaard M, et al. Electrical versus pharmacological cardioversion for emergency department patients with acute atrial fibrillation (RAFF2): a partial factorial randomised trial. Lancet. 2020;395(10221):339-349. doi:10.1016/S0140-6736(19)32994-0
Voskoboinik A, Kalman JM, De Silva A, et al. Alcohol Abstinence in Drinkers with Atrial Fibrillation. N Engl J Med. 2020;382(1):20-28. doi:10.1056/NEJMoa1817591

## Slide 13
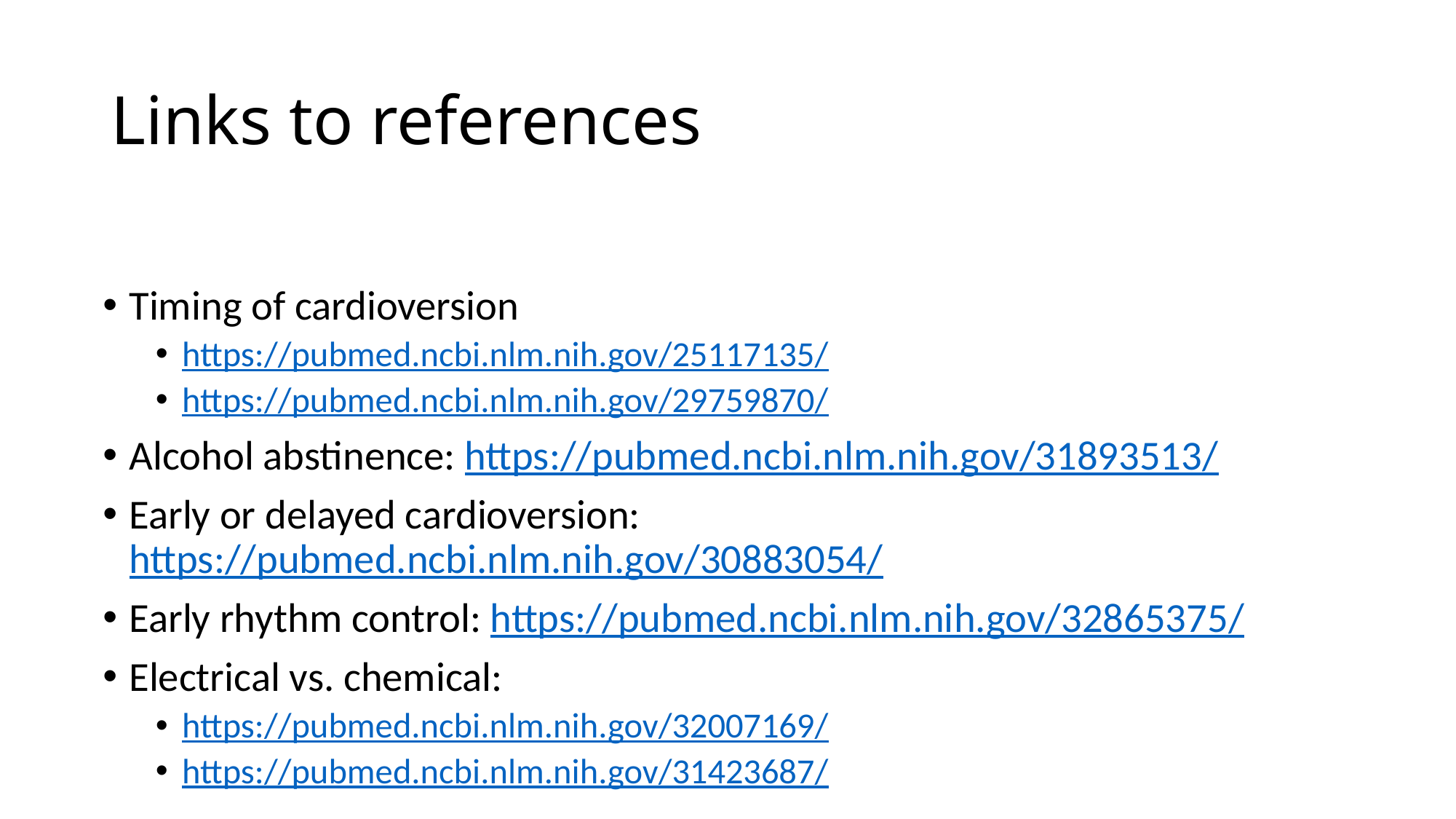

# Links to references
Timing of cardioversion
https://pubmed.ncbi.nlm.nih.gov/25117135/
https://pubmed.ncbi.nlm.nih.gov/29759870/
Alcohol abstinence: https://pubmed.ncbi.nlm.nih.gov/31893513/
Early or delayed cardioversion: https://pubmed.ncbi.nlm.nih.gov/30883054/
Early rhythm control: https://pubmed.ncbi.nlm.nih.gov/32865375/
Electrical vs. chemical:
https://pubmed.ncbi.nlm.nih.gov/32007169/
https://pubmed.ncbi.nlm.nih.gov/31423687/

## Slide 14
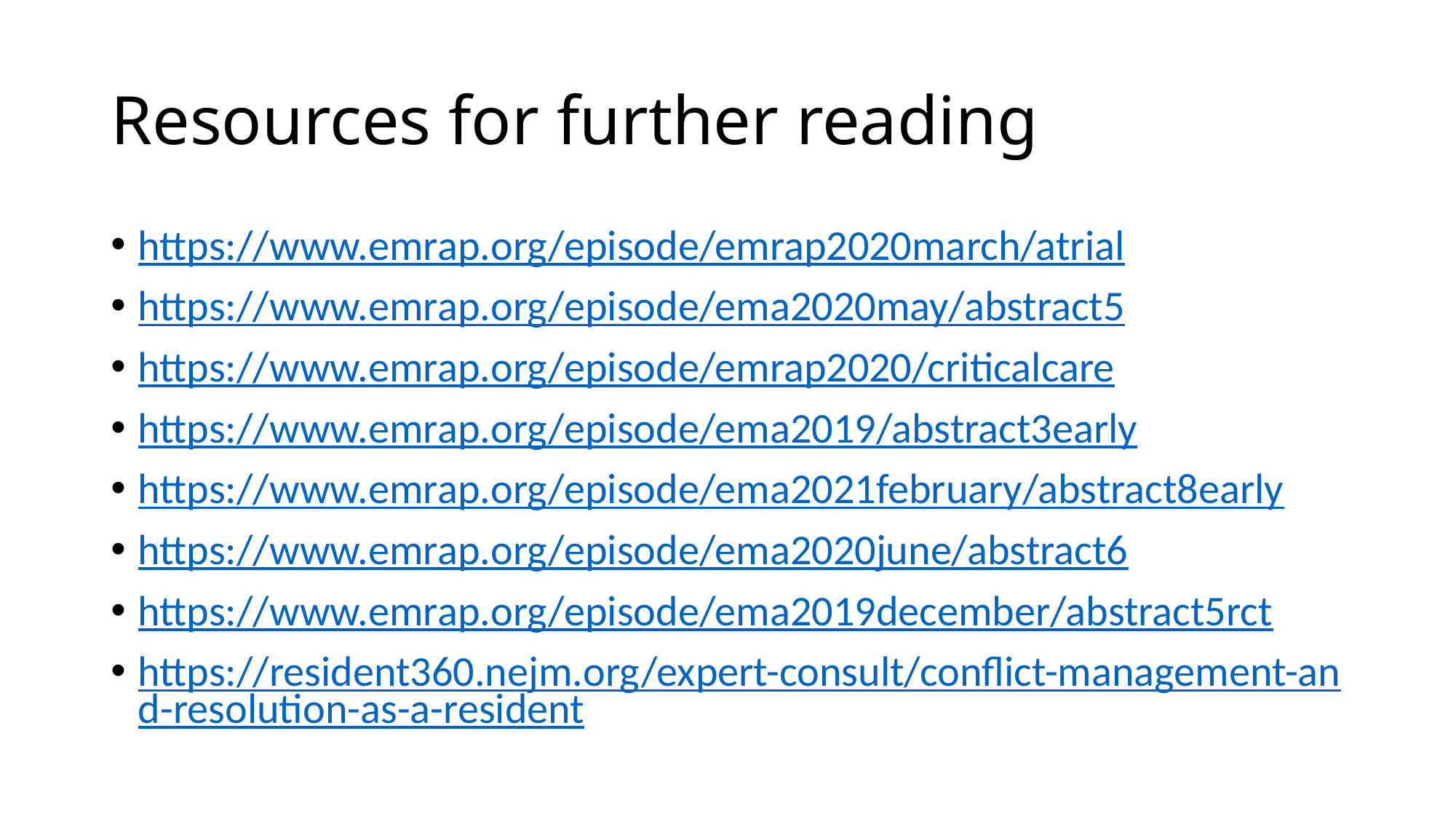

# Resources for further reading
https://www.emrap.org/episode/emrap2020march/atrial
https://www.emrap.org/episode/ema2020may/abstract5
https://www.emrap.org/episode/emrap2020/criticalcare
https://www.emrap.org/episode/ema2019/abstract3early
https://www.emrap.org/episode/ema2021february/abstract8early
https://www.emrap.org/episode/ema2020june/abstract6
https://www.emrap.org/episode/ema2019december/abstract5rct
https://resident360.nejm.org/expert-consult/conflict-management-and-resolution-as-a-resident
