# Supplementary material for: Residents Are Coming: A Faculty Development Curriculum to Prepare a Community Site For New Learners
Source: J Educ Teach Emerg Med. 2022 Jul 15;7(3):C1–C41. doi: 10.21980/J87D2N (PMC10332697; doi:10.21980/J87D2N)
Supplement: Supplementary file 9 — Please see associated PowerPoint file [file jetem-7-3-c1-appendix11.pptx]

## Slide 1
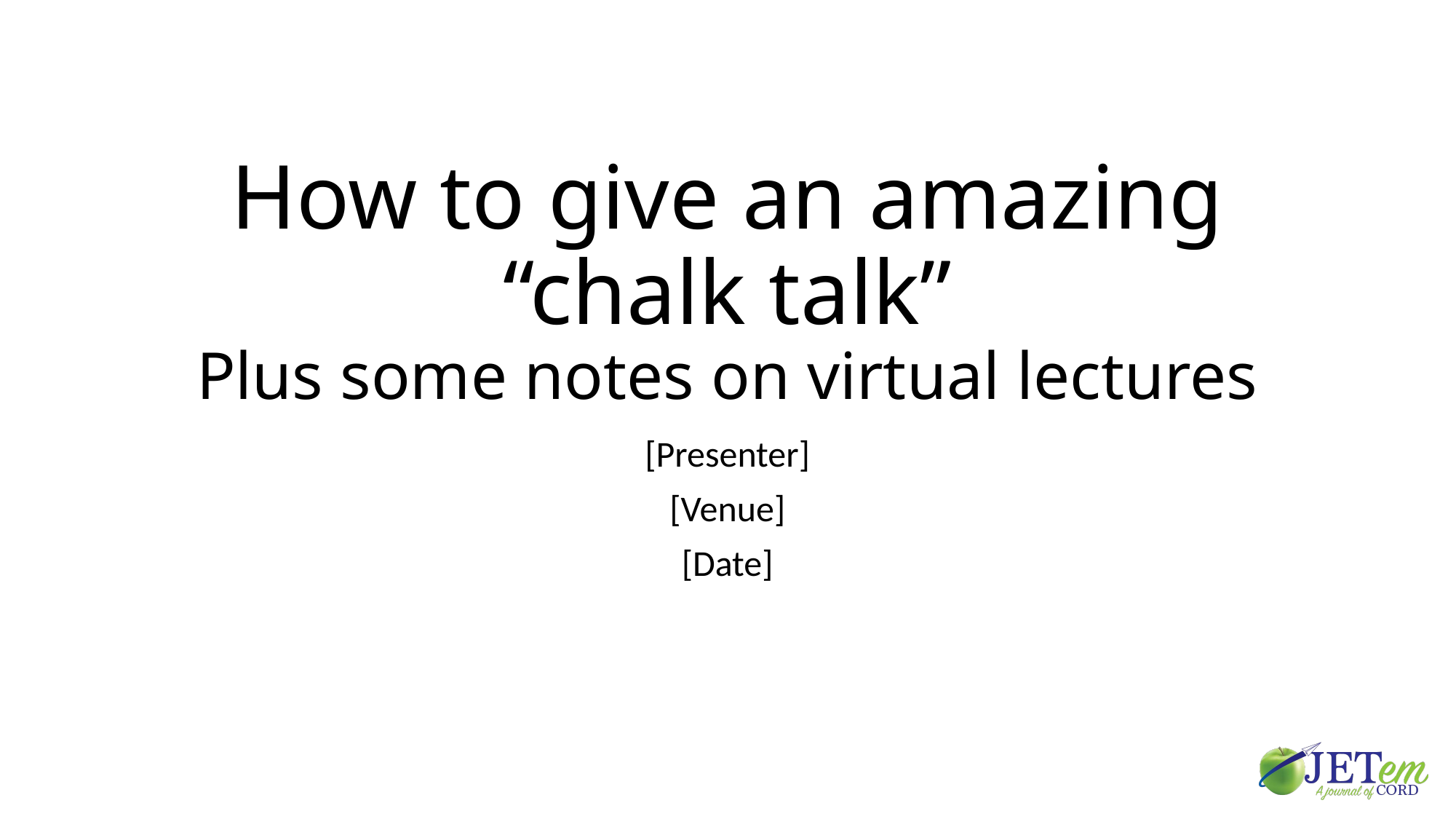

# How to give an amazing “chalk talk” Plus some notes on virtual lectures
[Presenter]
[Venue]
[Date]

## Slide 2
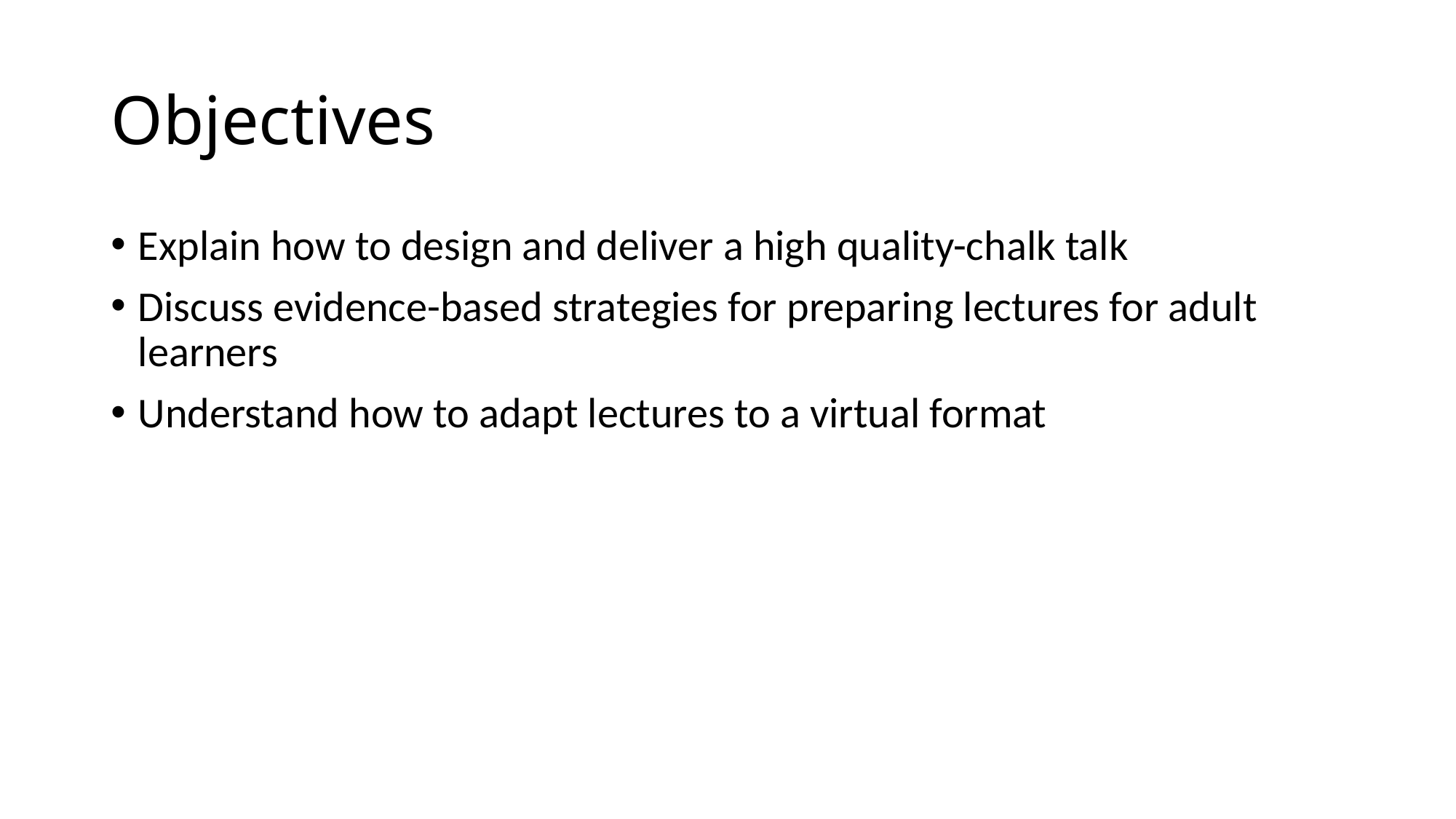

# Objectives
Explain how to design and deliver a high quality-chalk talk
Discuss evidence-based strategies for preparing lectures for adult learners
Understand how to adapt lectures to a virtual format

## Slide 3
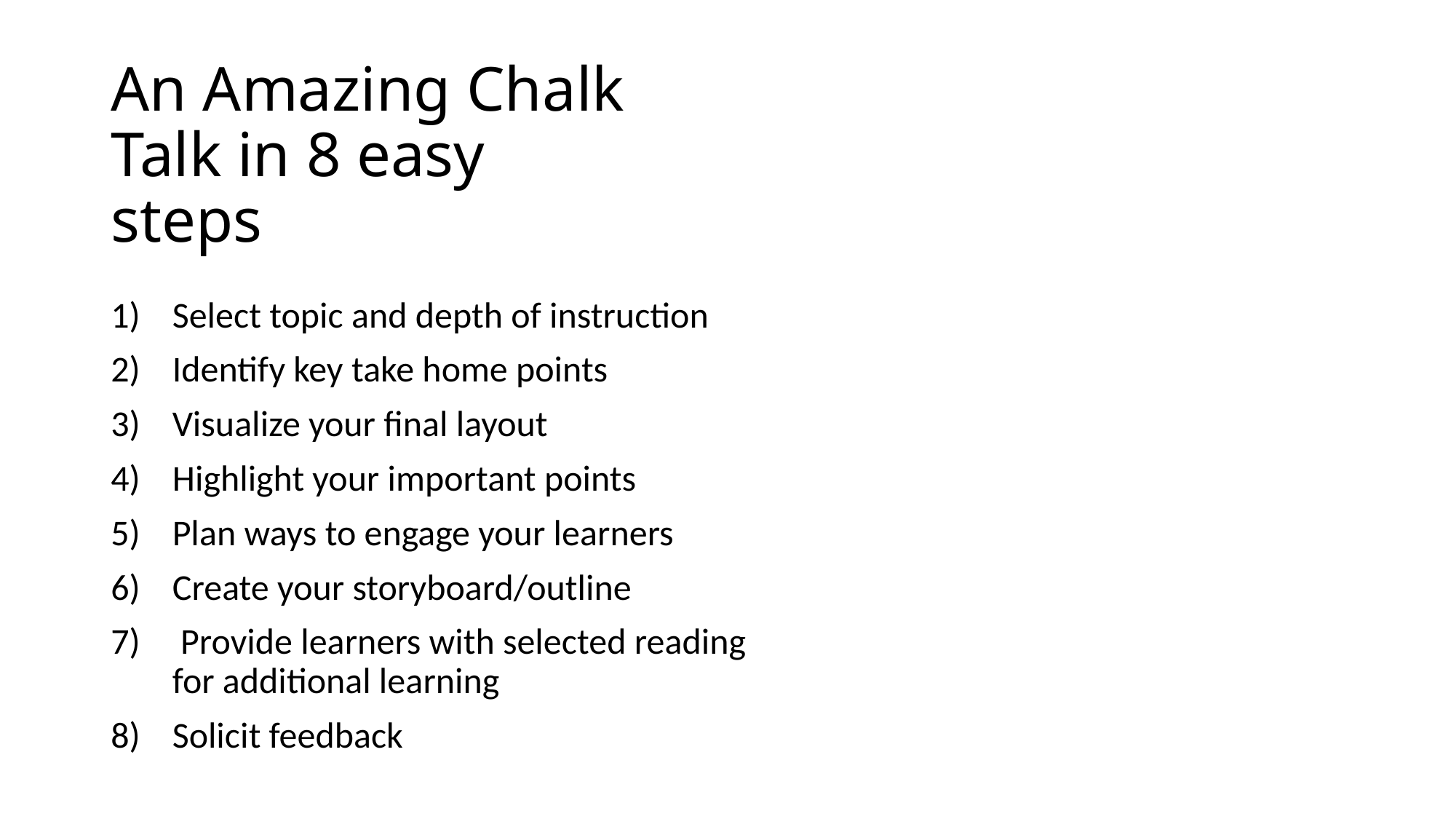

# An Amazing Chalk Talk in 8 easy steps
Select topic and depth of instruction
Identify key take home points
Visualize your final layout
Highlight your important points
Plan ways to engage your learners
Create your storyboard/outline
 Provide learners with selected reading for additional learning
Solicit feedback

## Slide 4
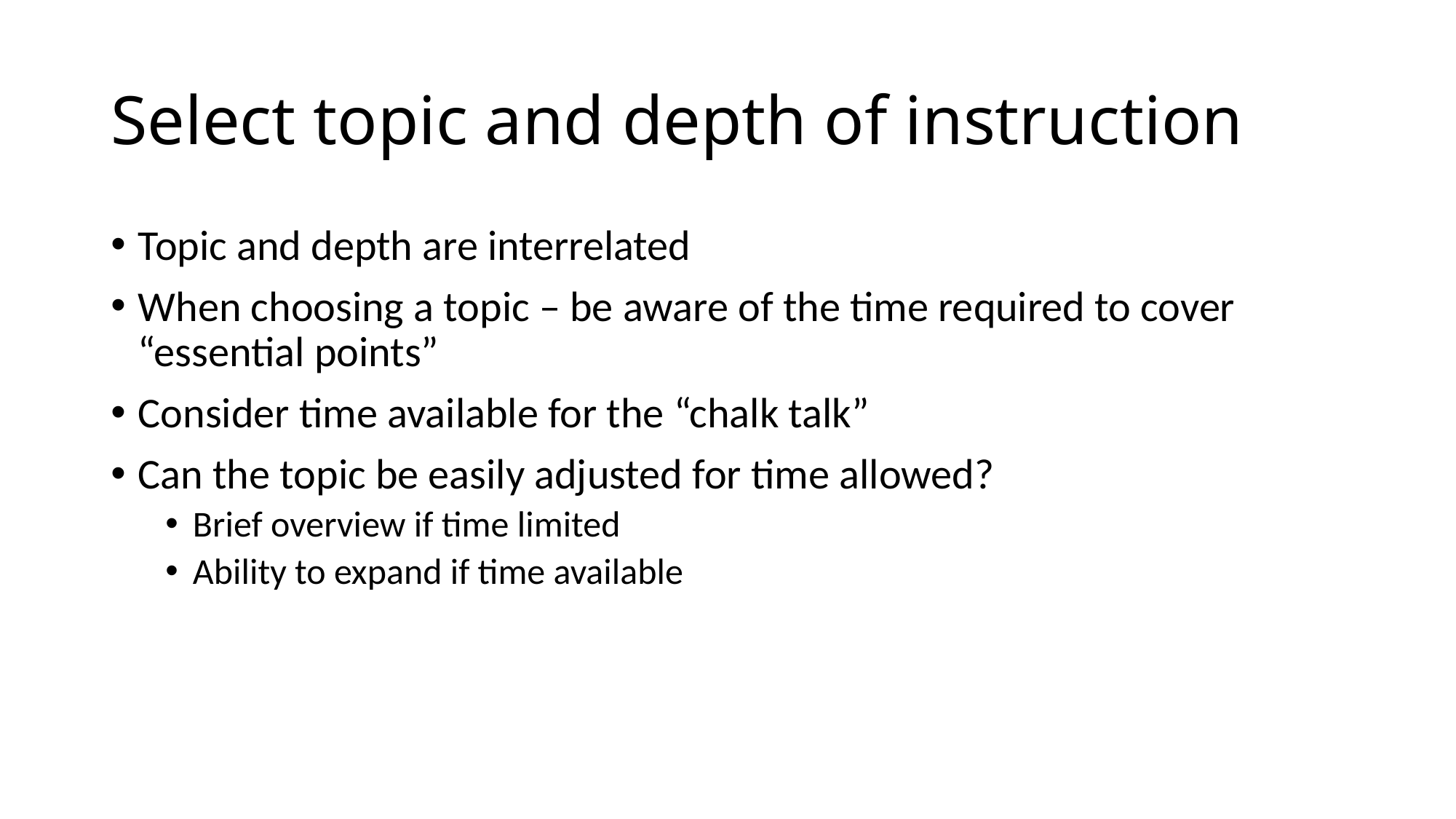

# Select topic and depth of instruction
Topic and depth are interrelated
When choosing a topic – be aware of the time required to cover “essential points”
Consider time available for the “chalk talk”
Can the topic be easily adjusted for time allowed?
Brief overview if time limited
Ability to expand if time available

## Slide 5
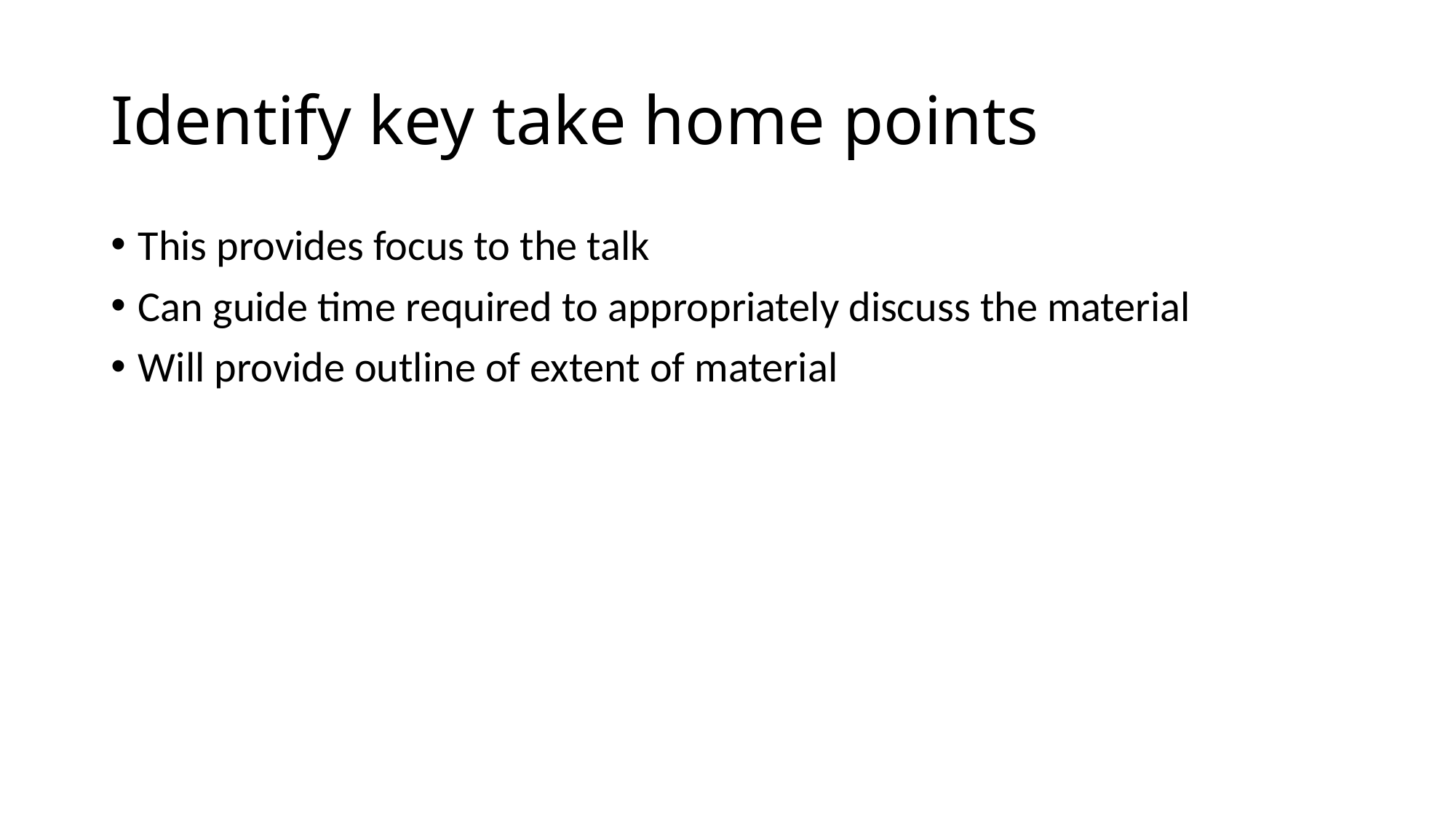

# Identify key take home points
This provides focus to the talk
Can guide time required to appropriately discuss the material
Will provide outline of extent of material

## Slide 6
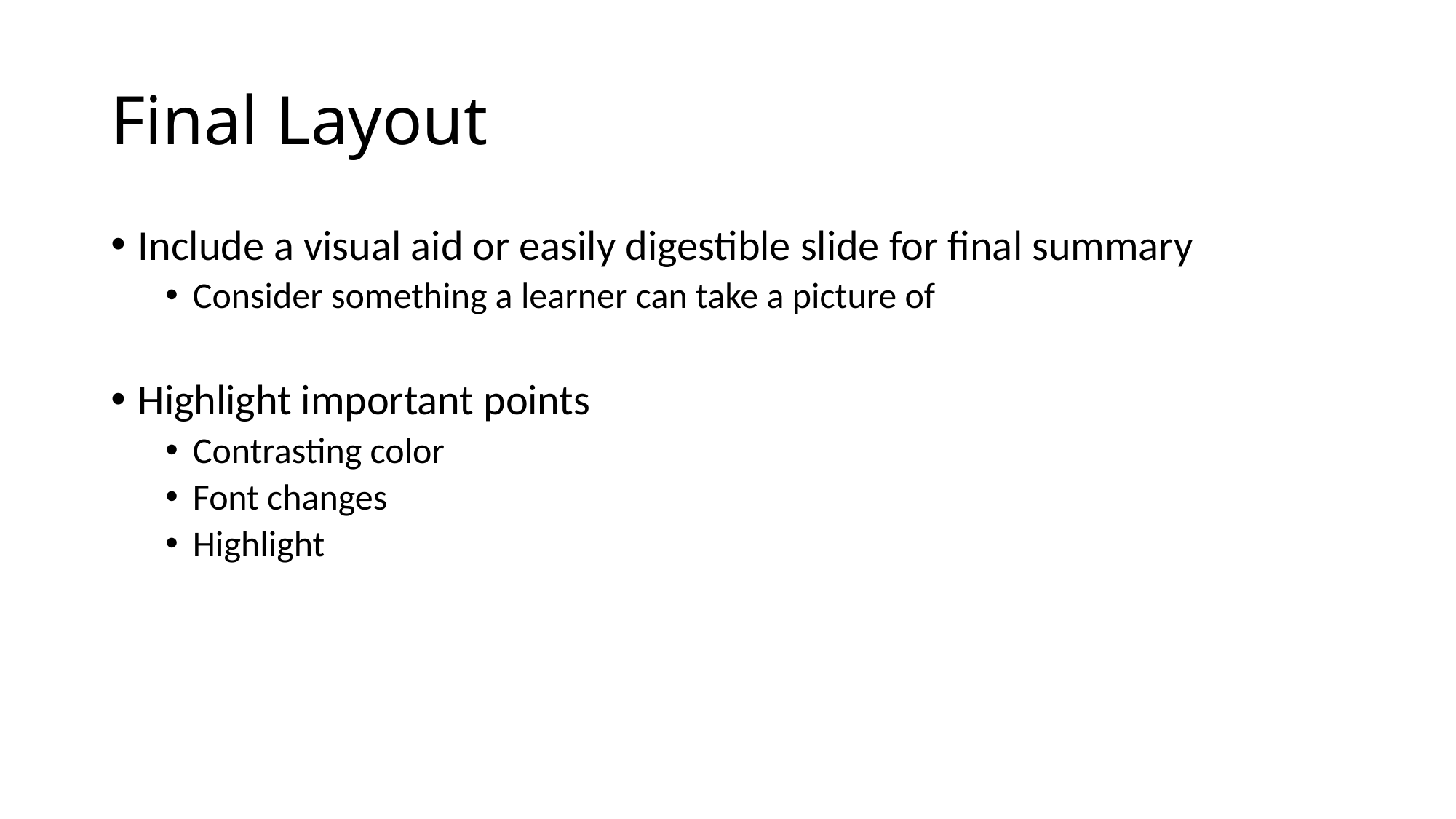

# Final Layout
Include a visual aid or easily digestible slide for final summary
Consider something a learner can take a picture of
Highlight important points
Contrasting color
Font changes
Highlight

## Slide 7
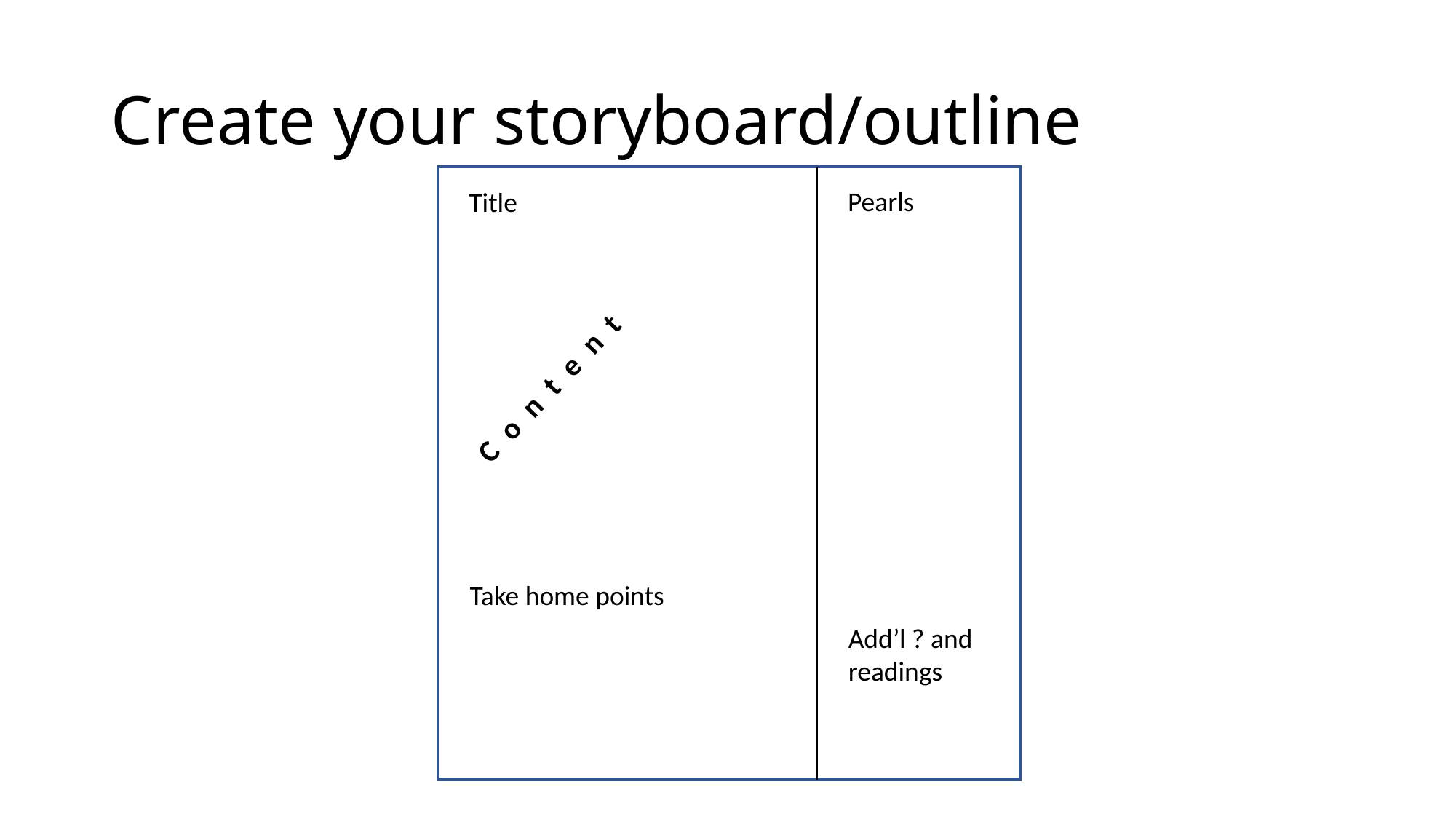

# Create your storyboard/outline
Pearls
Title
Content
Take home points
Add’l ? and
readings

## Slide 8
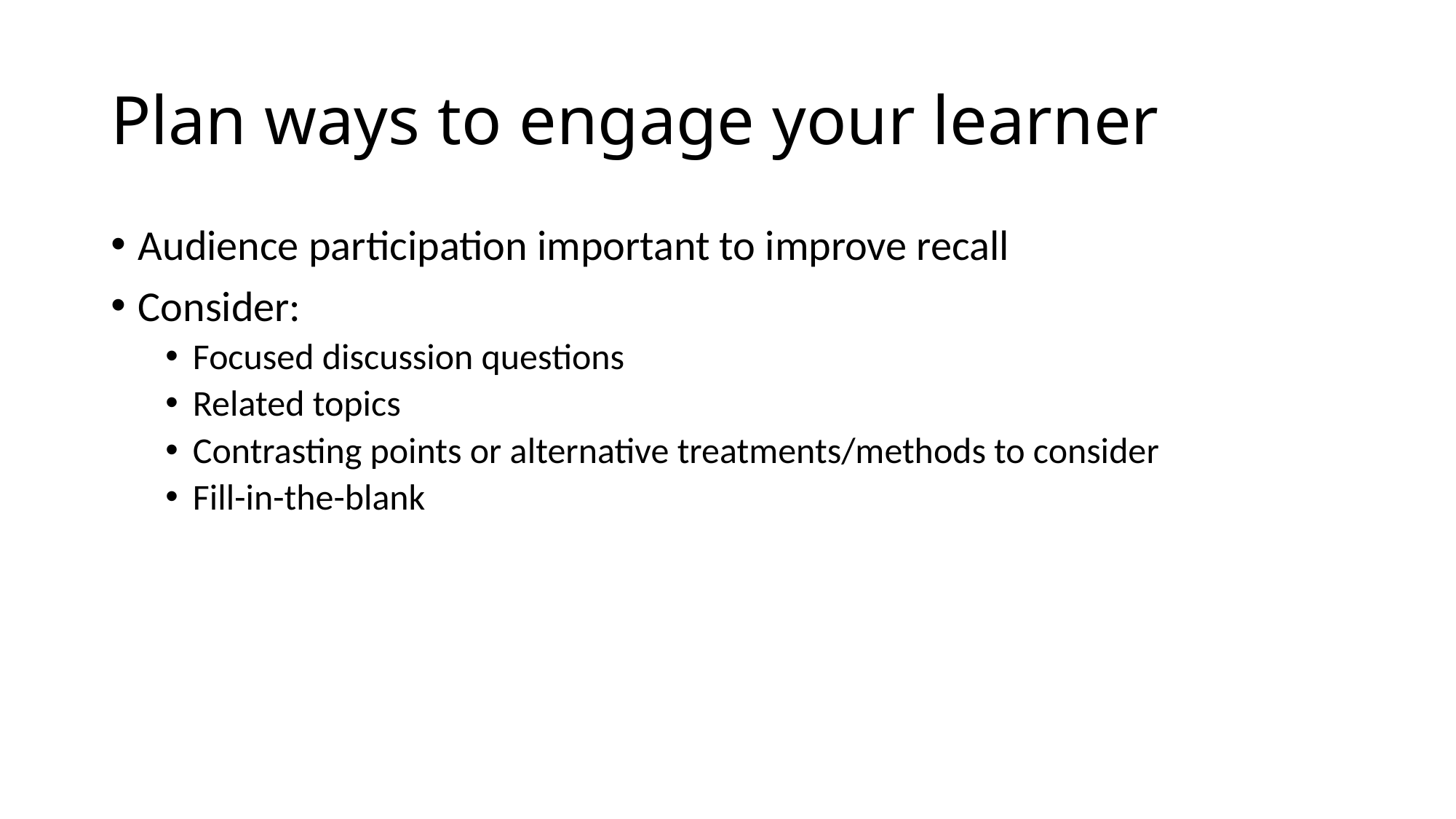

# Plan ways to engage your learner
Audience participation important to improve recall
Consider:
Focused discussion questions
Related topics
Contrasting points or alternative treatments/methods to consider
Fill-in-the-blank

## Slide 9
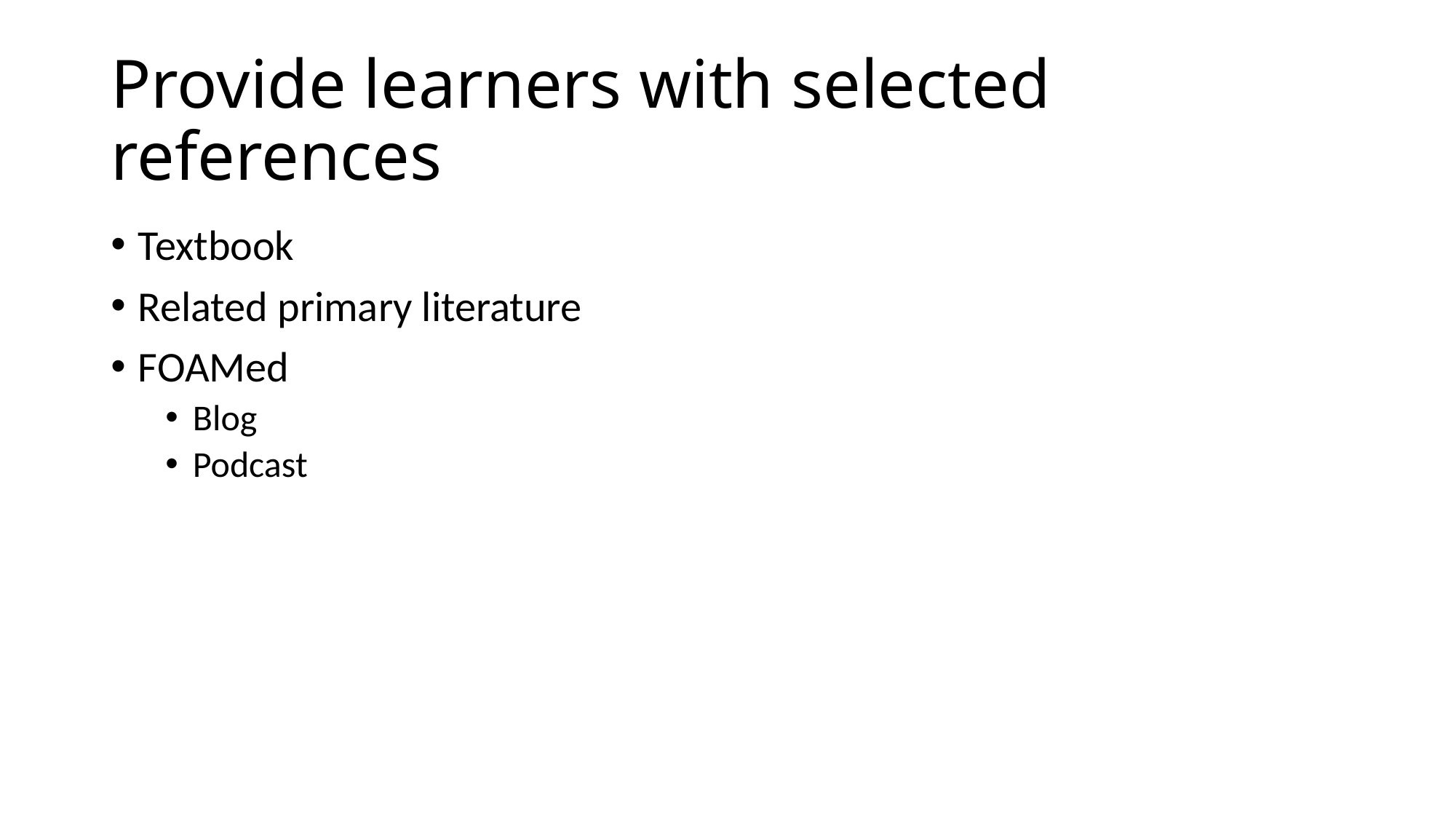

# Provide learners with selected references
Textbook
Related primary literature
FOAMed
Blog
Podcast

## Slide 10
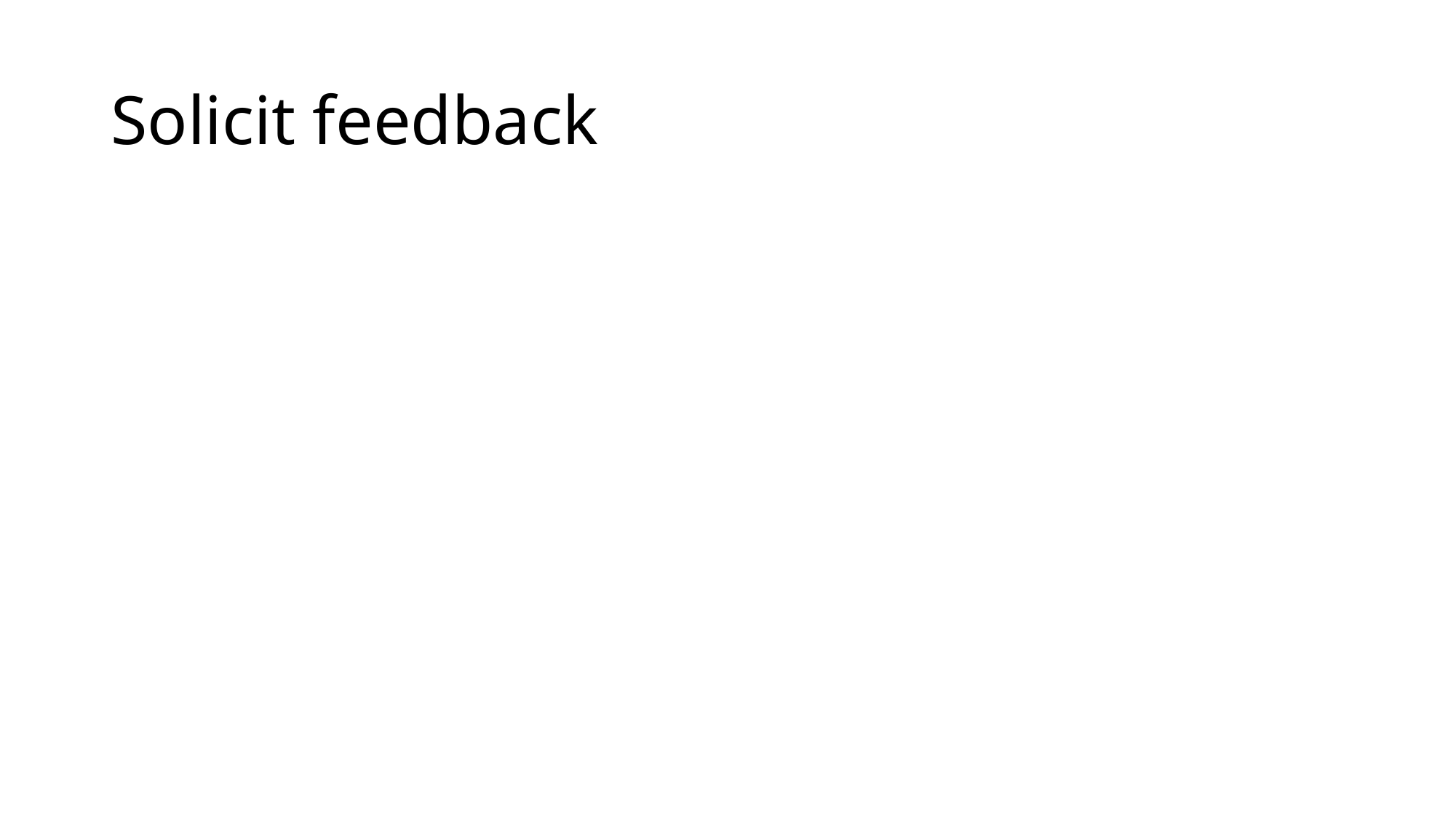

# Solicit feedback

## Slide 11
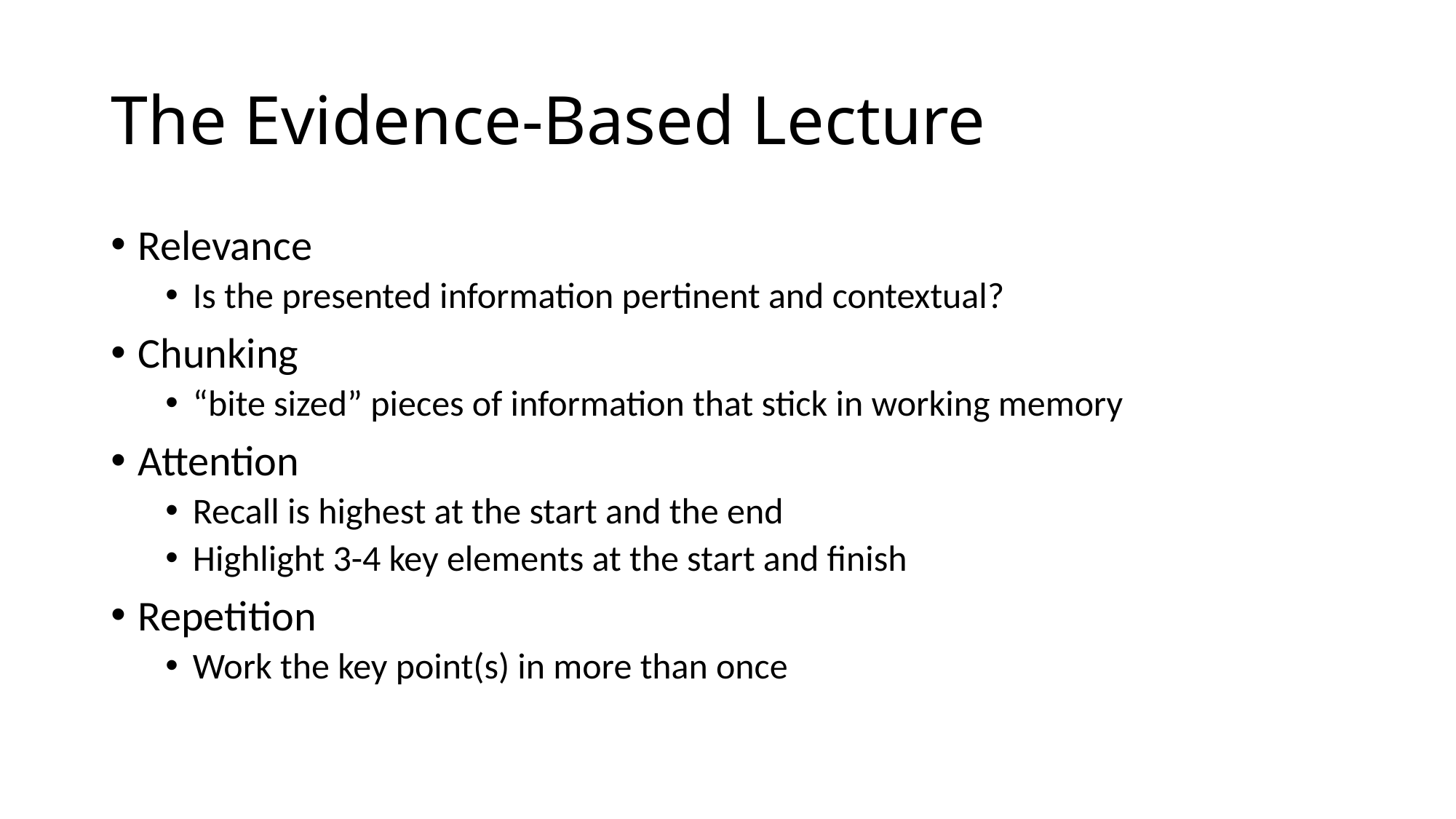

# The Evidence-Based Lecture
Relevance
Is the presented information pertinent and contextual?
Chunking
“bite sized” pieces of information that stick in working memory
Attention
Recall is highest at the start and the end
Highlight 3-4 key elements at the start and finish
Repetition
Work the key point(s) in more than once

## Slide 12
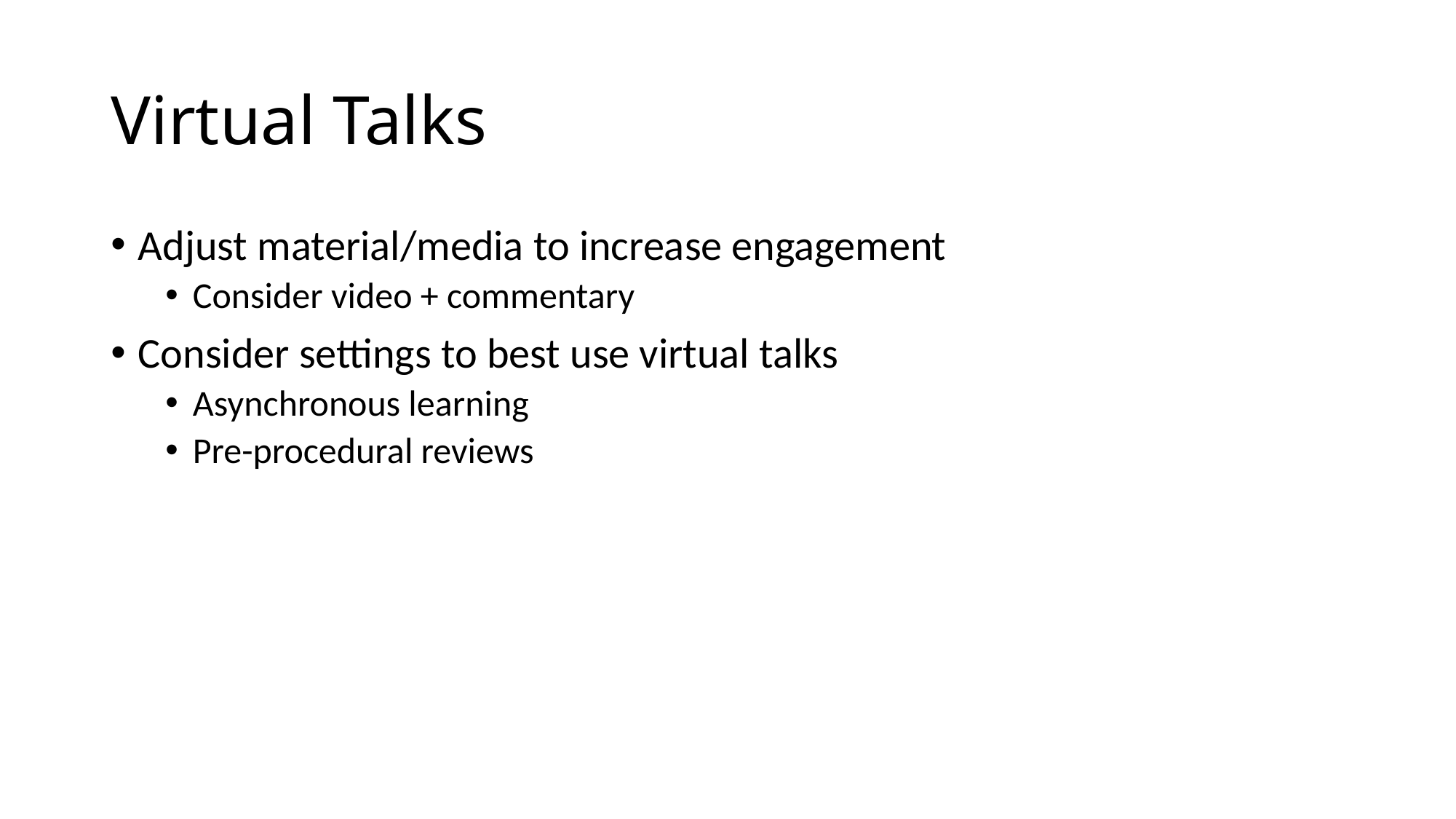

# Virtual Talks
Adjust material/media to increase engagement
Consider video + commentary
Consider settings to best use virtual talks
Asynchronous learning
Pre-procedural reviews

## Slide 13
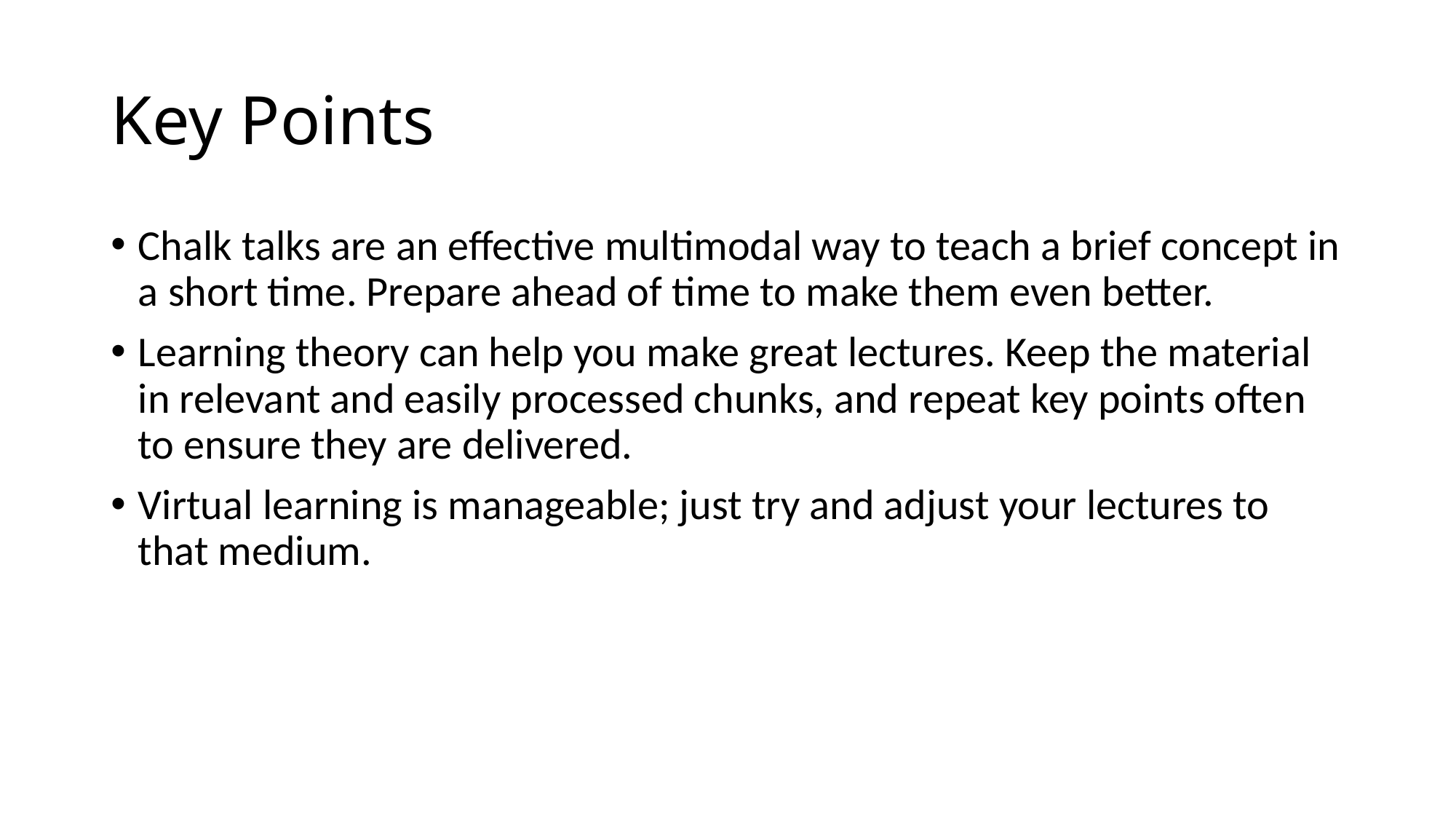

# Key Points
Chalk talks are an effective multimodal way to teach a brief concept in a short time. Prepare ahead of time to make them even better.
Learning theory can help you make great lectures. Keep the material in relevant and easily processed chunks, and repeat key points often to ensure they are delivered.
Virtual learning is manageable; just try and adjust your lectures to that medium.

## Slide 14
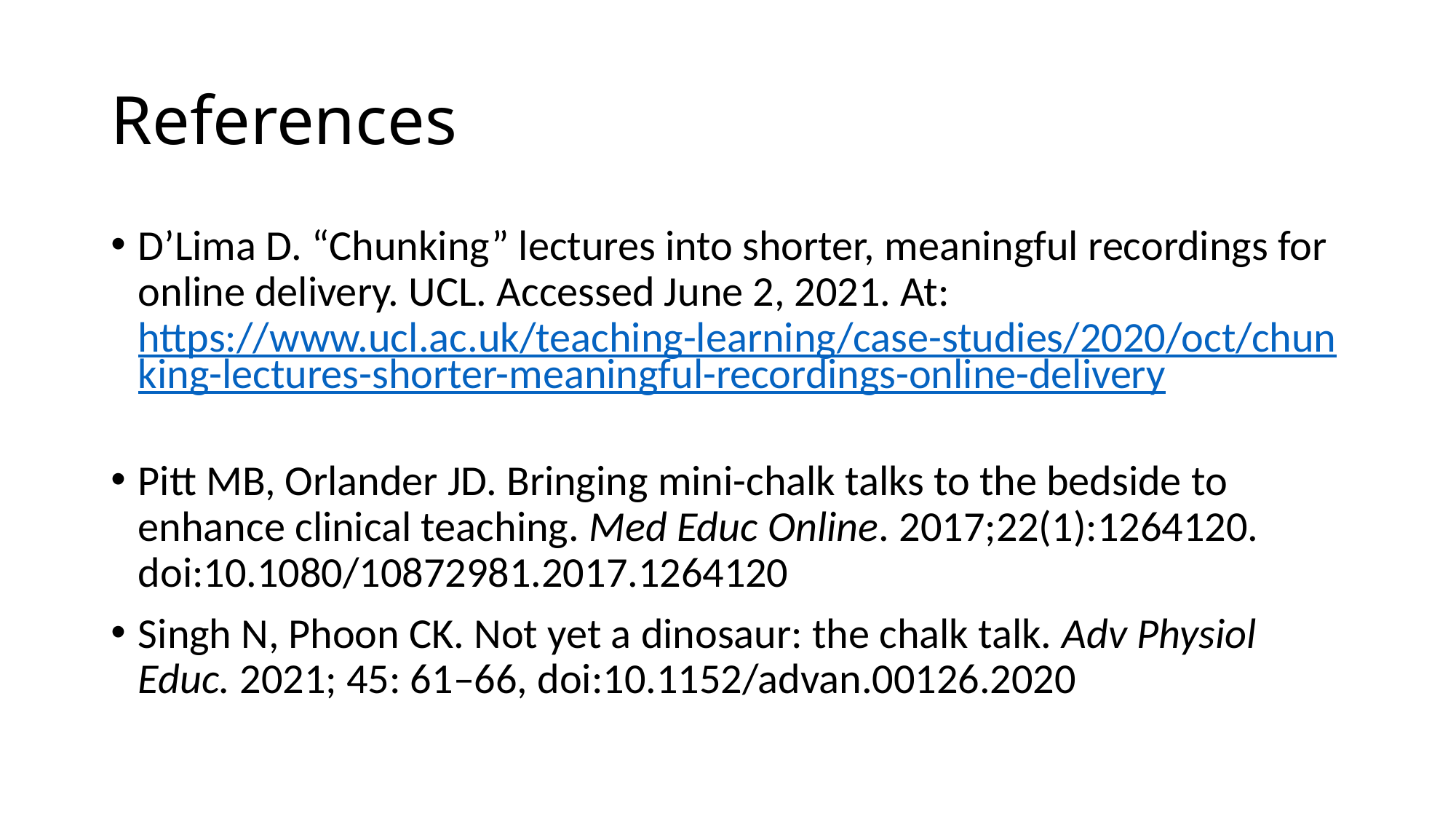

# References
D’Lima D. “Chunking” lectures into shorter, meaningful recordings for online delivery. UCL. Accessed June 2, 2021. At: https://www.ucl.ac.uk/teaching-learning/case-studies/2020/oct/chunking-lectures-shorter-meaningful-recordings-online-delivery
Pitt MB, Orlander JD. Bringing mini-chalk talks to the bedside to enhance clinical teaching. Med Educ Online. 2017;22(1):1264120. doi:10.1080/10872981.2017.1264120
Singh N, Phoon CK. Not yet a dinosaur: the chalk talk. Adv Physiol Educ. 2021; 45: 61–66, doi:10.1152/advan.00126.2020
