# Supplementary material for: Residents Are Coming: A Faculty Development Curriculum to Prepare a Community Site For New Learners
Source: J Educ Teach Emerg Med. 2022 Jul 15;7(3):C1–C41. doi: 10.21980/J87D2N (PMC10332697; doi:10.21980/J87D2N)
Supplement: Supplementary file 10 — Please see associated PowerPoint file [file jetem-7-3-c1-appendix12.pptx]

## Slide 1
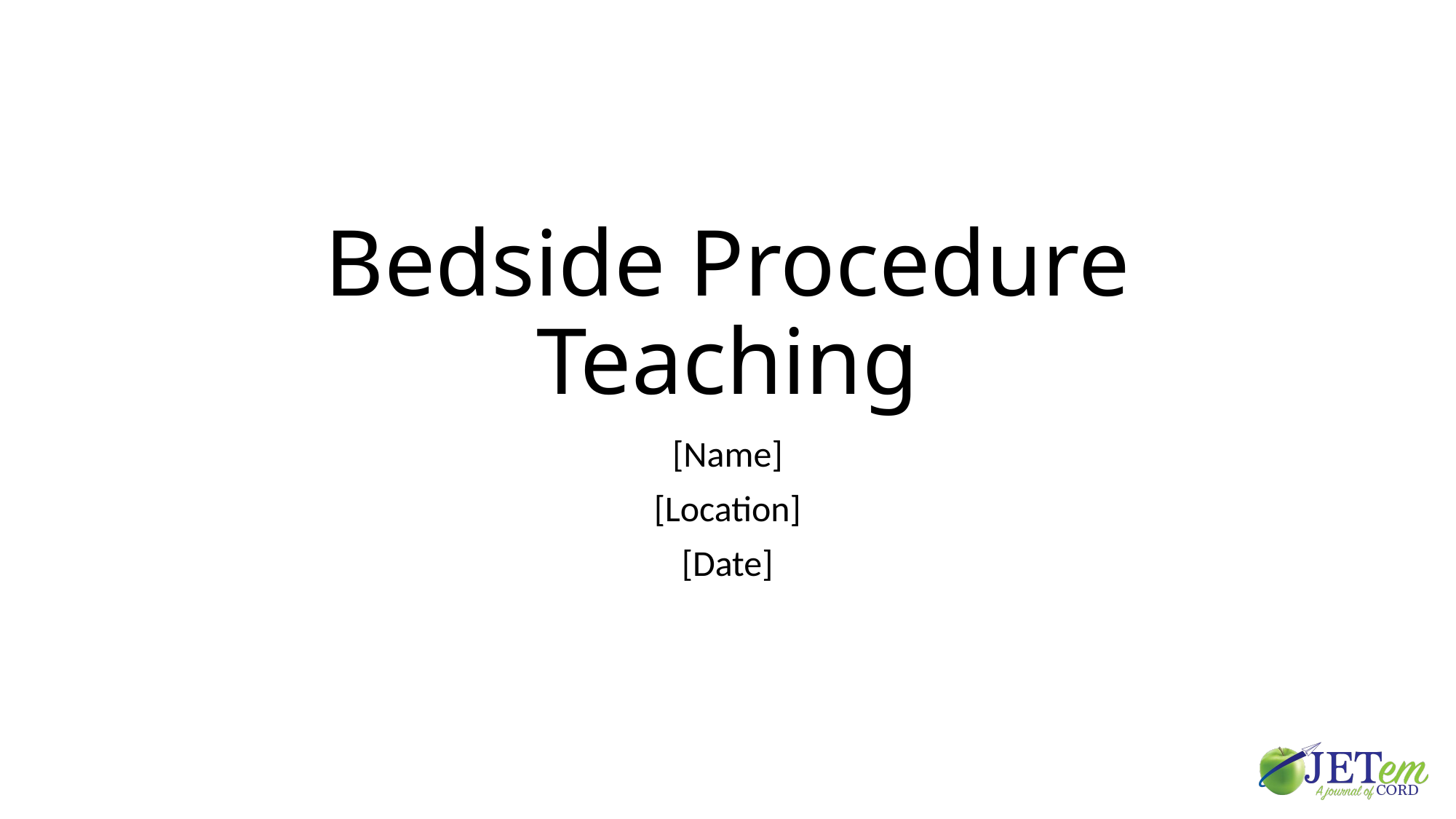

# Bedside Procedure Teaching
[Name]
[Location]
[Date]

## Slide 2
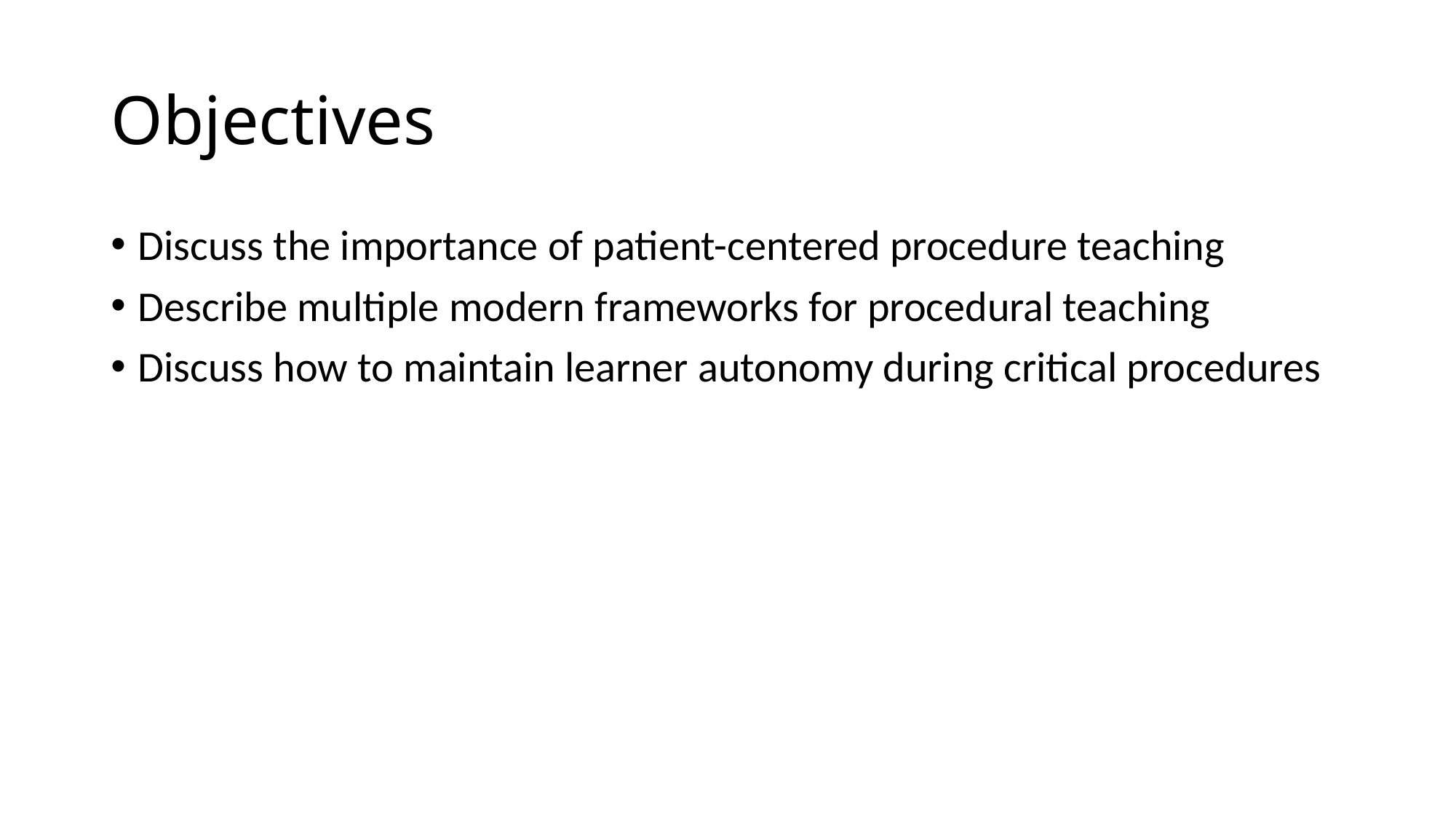

# Objectives
Discuss the importance of patient-centered procedure teaching
Describe multiple modern frameworks for procedural teaching
Discuss how to maintain learner autonomy during critical procedures

## Slide 3
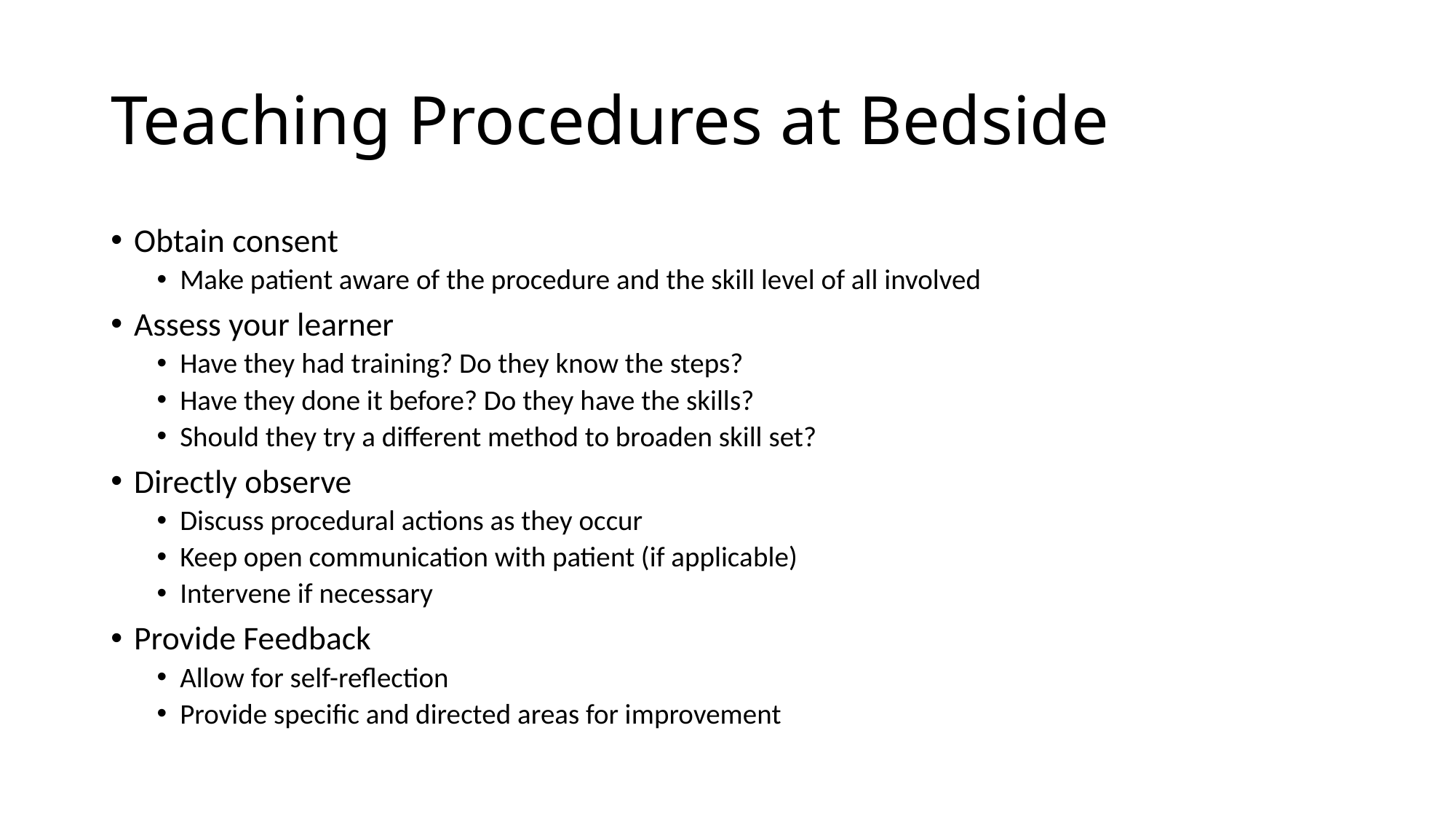

# Teaching Procedures at Bedside
Obtain consent
Make patient aware of the procedure and the skill level of all involved
Assess your learner
Have they had training? Do they know the steps?
Have they done it before? Do they have the skills?
Should they try a different method to broaden skill set?
Directly observe
Discuss procedural actions as they occur
Keep open communication with patient (if applicable)
Intervene if necessary
Provide Feedback
Allow for self-reflection
Provide specific and directed areas for improvement

## Slide 4
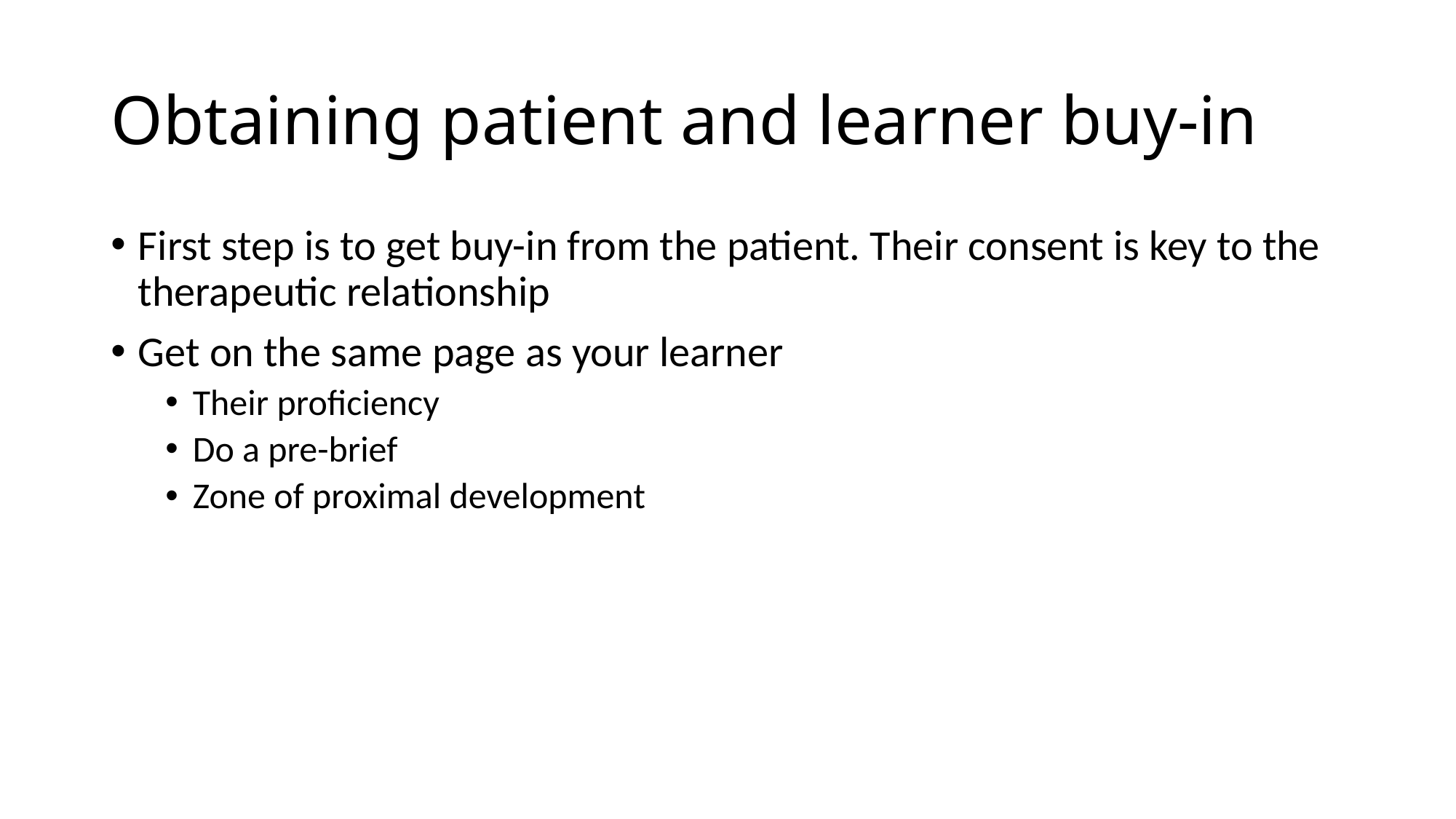

# Obtaining patient and learner buy-in
First step is to get buy-in from the patient. Their consent is key to the therapeutic relationship
Get on the same page as your learner
Their proficiency
Do a pre-brief
Zone of proximal development

## Slide 5
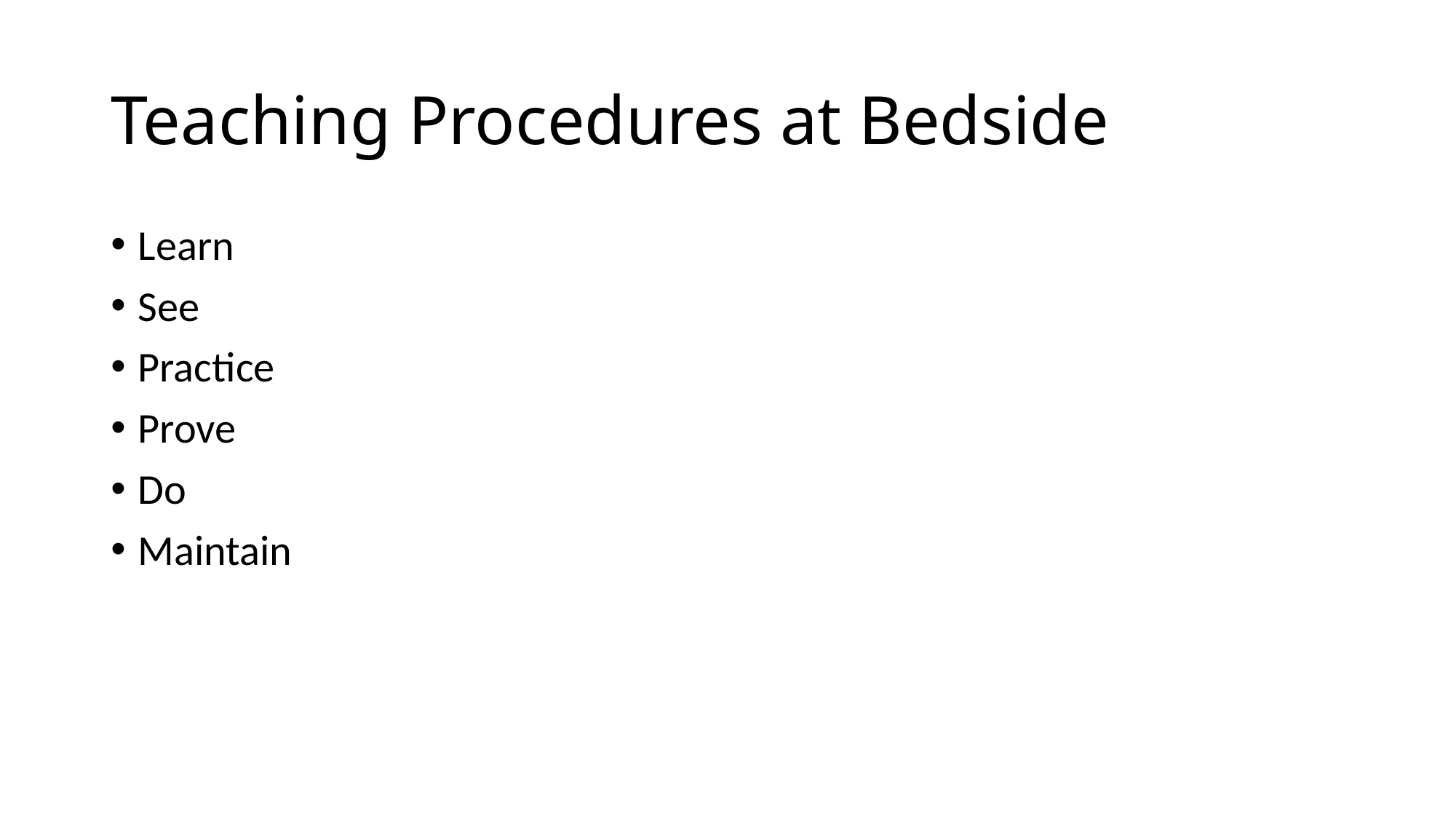

# Teaching Procedures at Bedside
Learn
See
Practice
Prove
Do
Maintain

## Slide 6
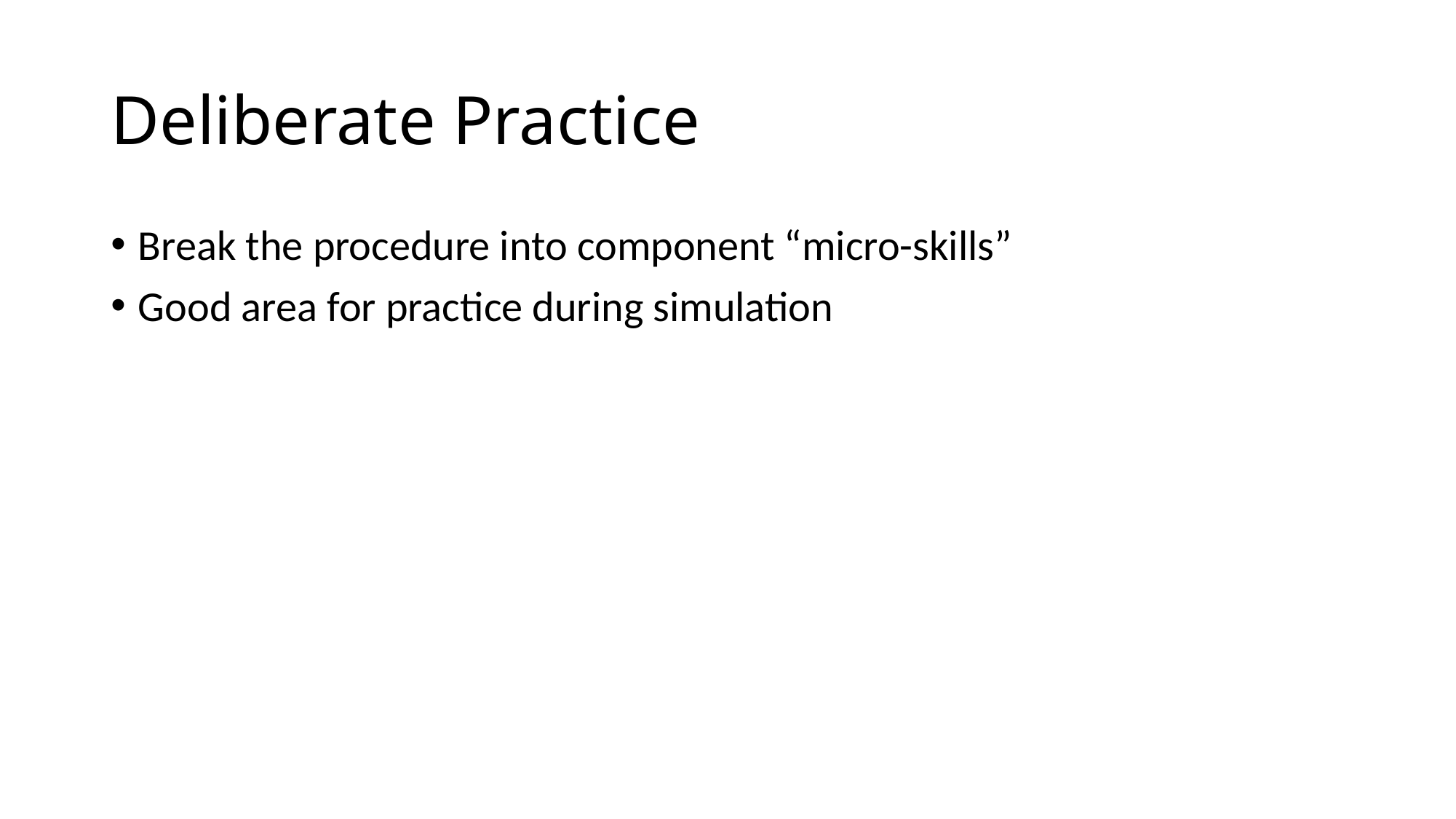

# Deliberate Practice
Break the procedure into component “micro-skills”
Good area for practice during simulation

## Slide 7
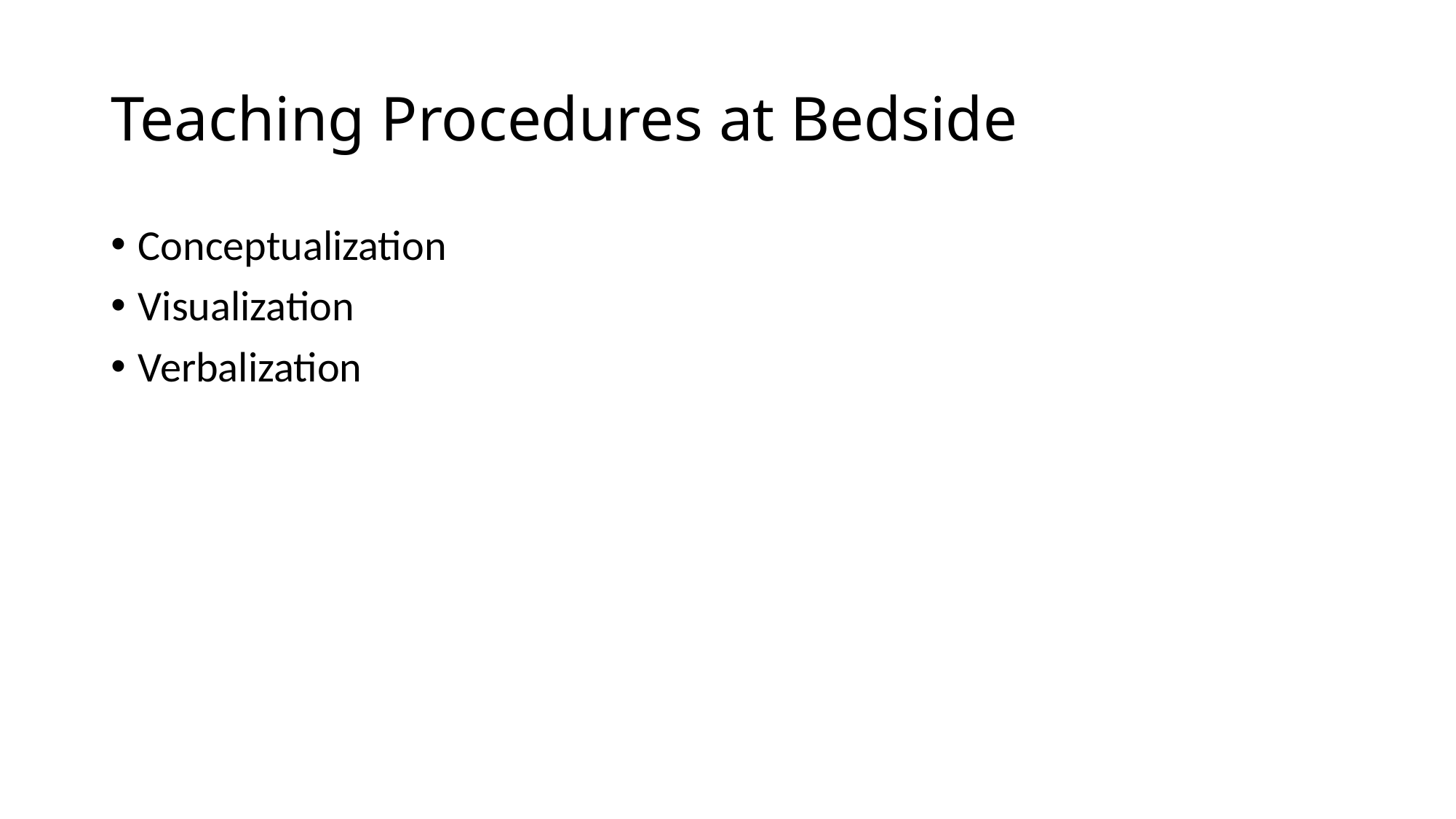

# Teaching Procedures at Bedside
Conceptualization
Visualization
Verbalization

## Slide 8
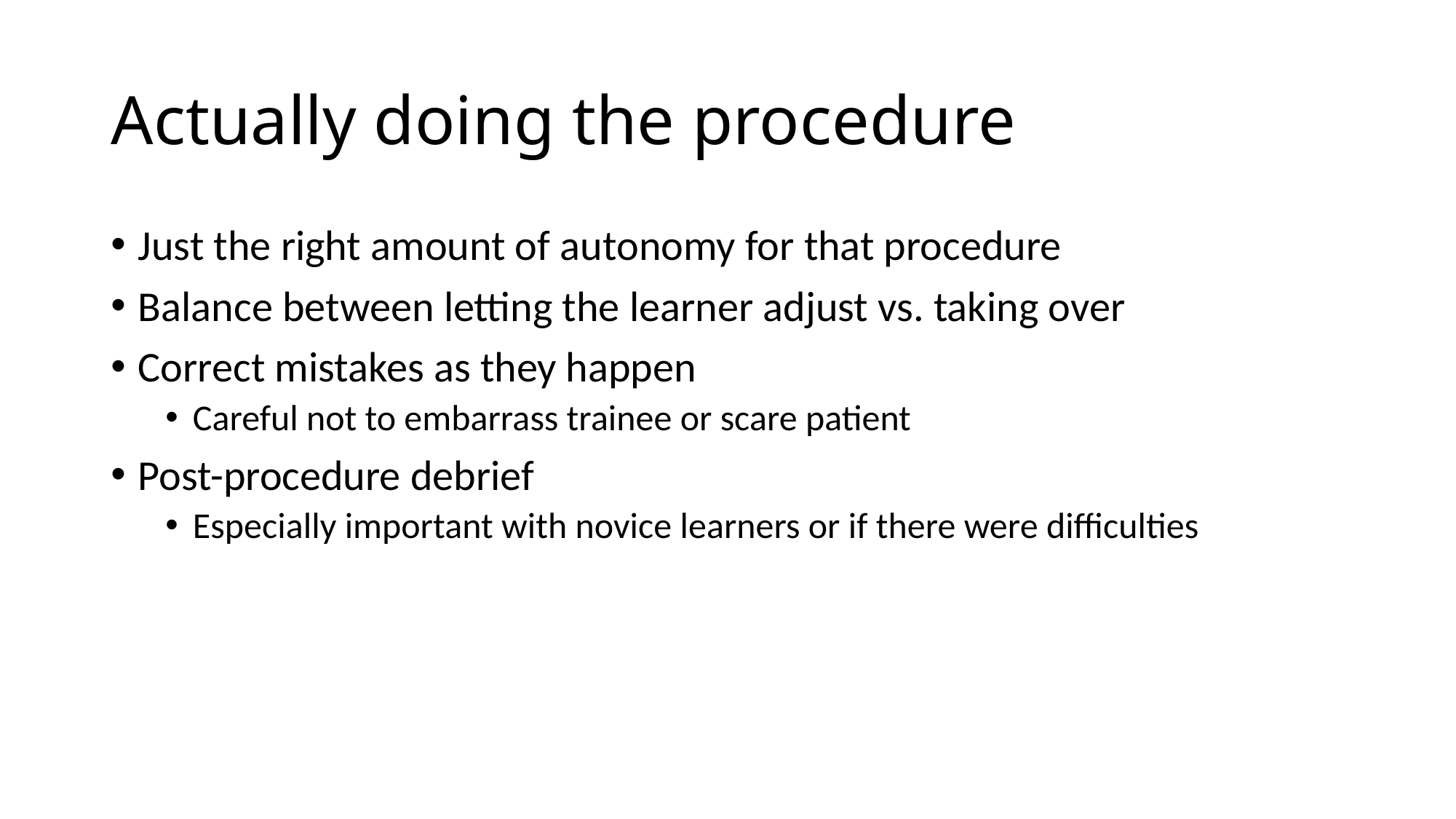

# Actually doing the procedure
Just the right amount of autonomy for that procedure
Balance between letting the learner adjust vs. taking over
Correct mistakes as they happen
Careful not to embarrass trainee or scare patient
Post-procedure debrief
Especially important with novice learners or if there were difficulties

## Slide 9
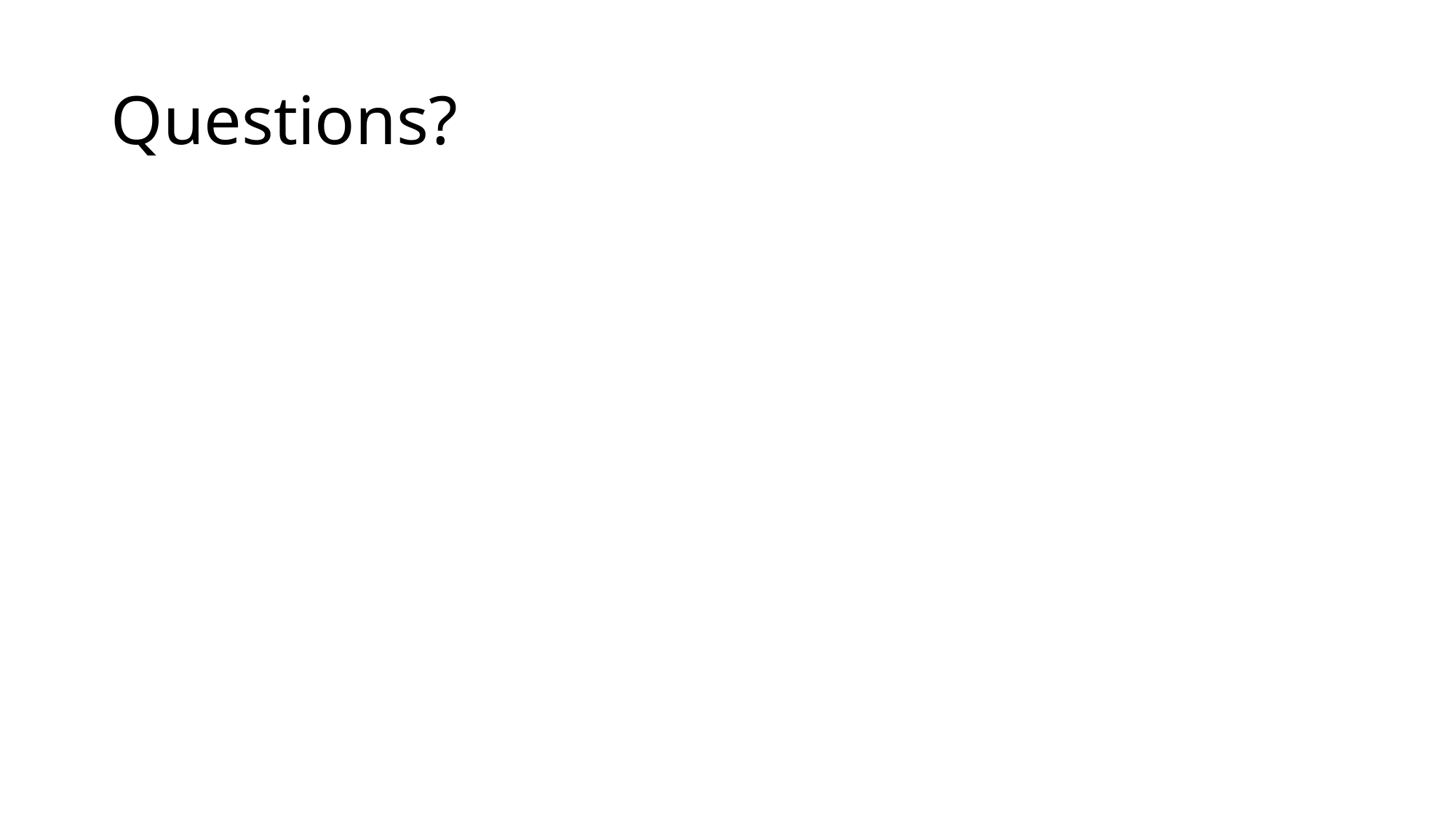

# Questions?

## Slide 10
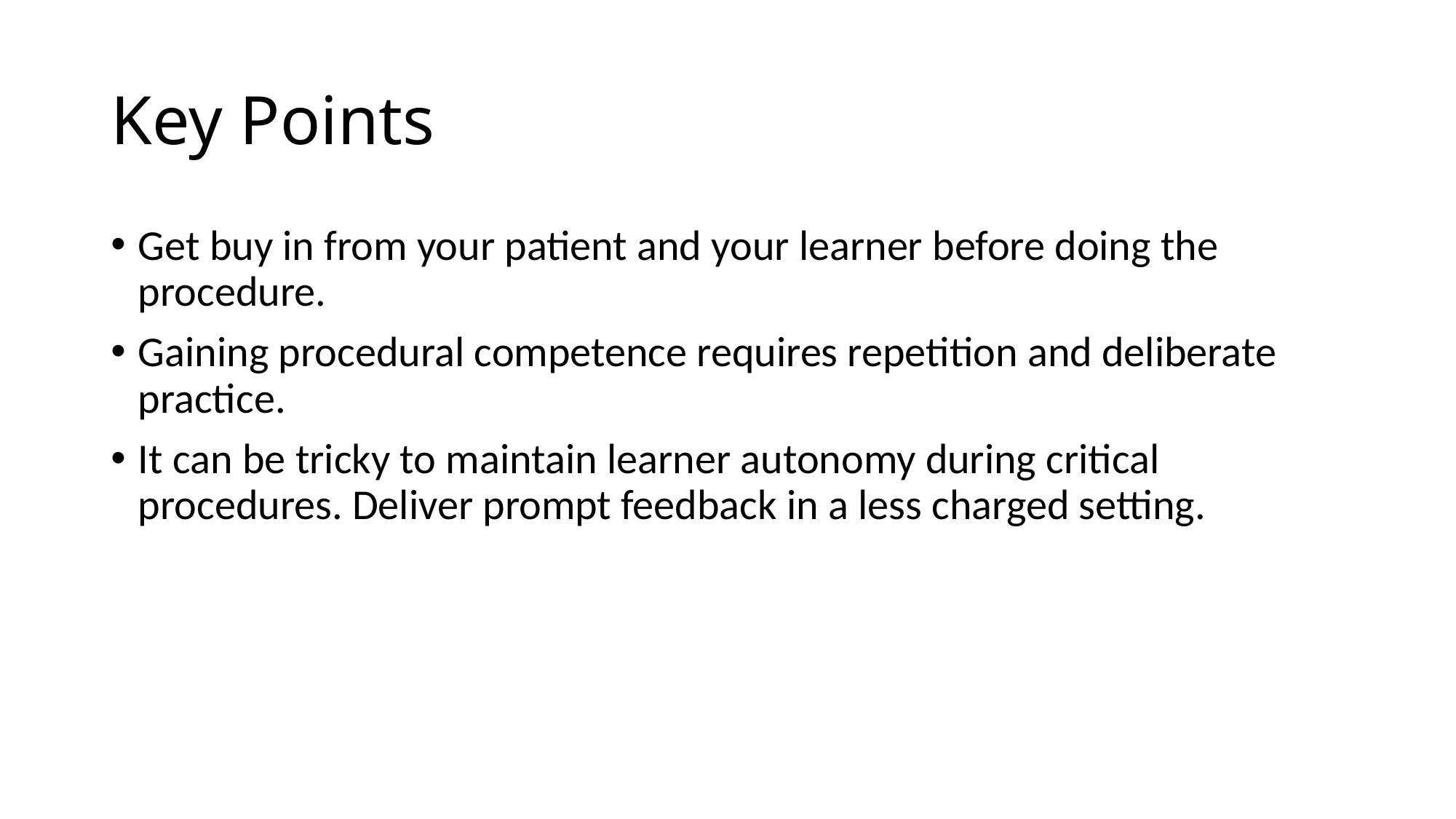

# Key Points
Get buy in from your patient and your learner before doing the procedure.
Gaining procedural competence requires repetition and deliberate practice.
It can be tricky to maintain learner autonomy during critical procedures. Deliver prompt feedback in a less charged setting.

## Slide 11
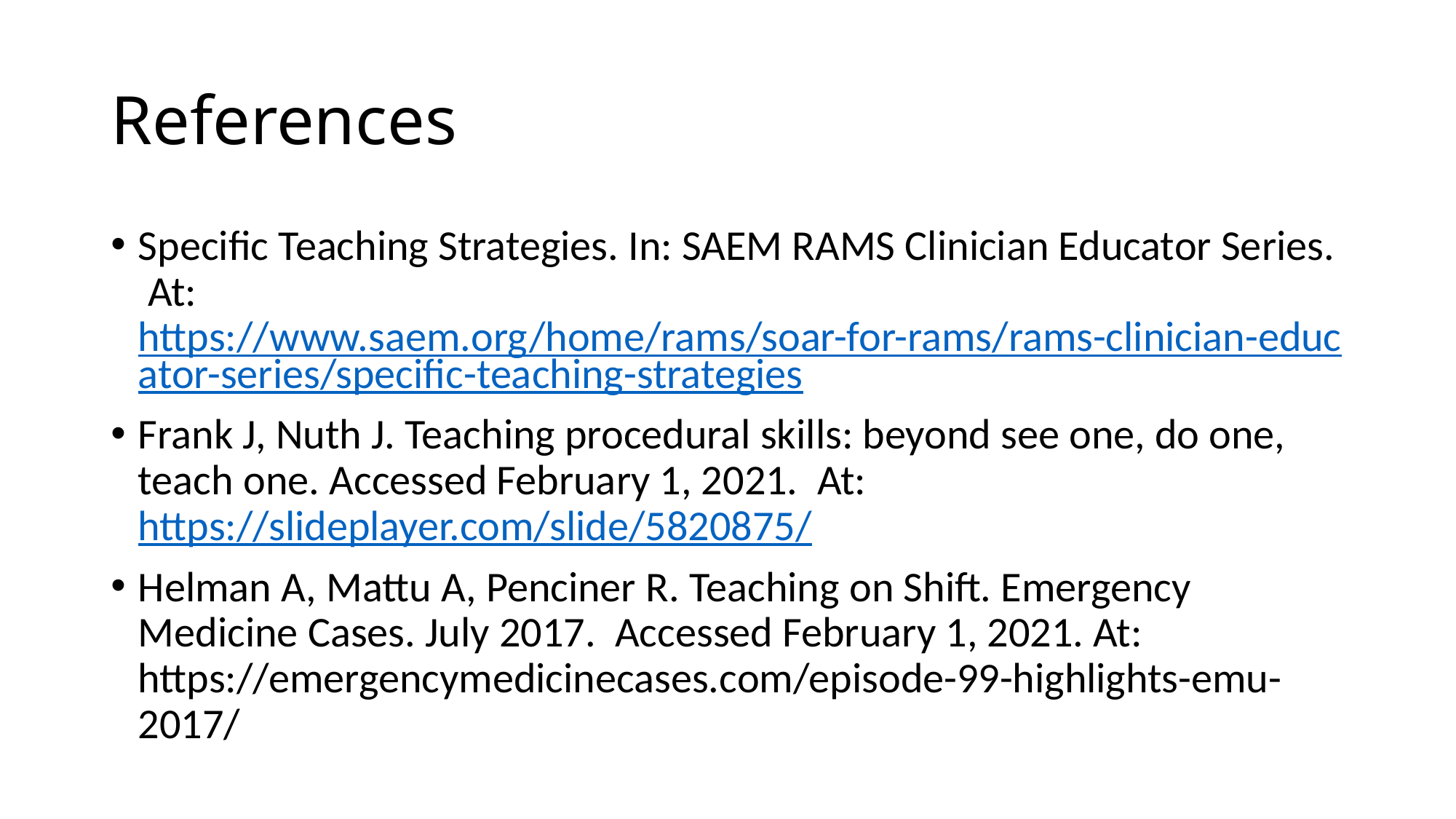

# References
Specific Teaching Strategies. In: SAEM RAMS Clinician Educator Series. At: https://www.saem.org/home/rams/soar-for-rams/rams-clinician-educator-series/specific-teaching-strategies
Frank J, Nuth J. Teaching procedural skills: beyond see one, do one, teach one. Accessed February 1, 2021. At: https://slideplayer.com/slide/5820875/
Helman A, Mattu A, Penciner R. Teaching on Shift. Emergency Medicine Cases. July 2017. Accessed February 1, 2021. At: https://emergencymedicinecases.com/episode-99-highlights-emu-2017/
