# Supplementary material for: Residents Are Coming: A Faculty Development Curriculum to Prepare a Community Site For New Learners
Source: J Educ Teach Emerg Med. 2022 Jul 15;7(3):C1–C41. doi: 10.21980/J87D2N (PMC10332697; doi:10.21980/J87D2N)
Supplement: Supplementary file 11 — Please see associated PowerPoint file [file jetem-7-3-c1-appendix13.pptx]

## Slide 1
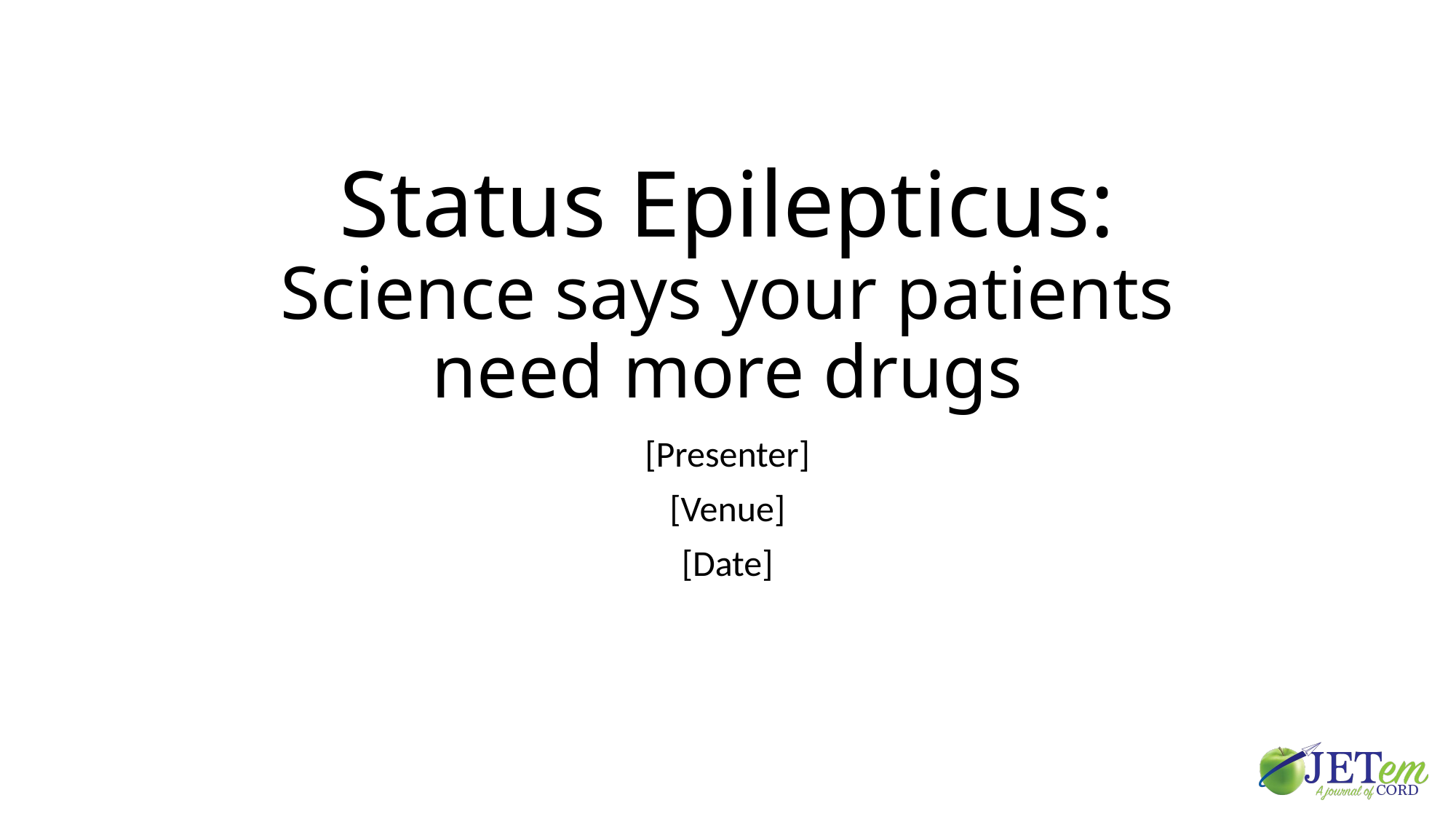

# Status Epilepticus: Science says your patients need more drugs
[Presenter]
[Venue]
[Date]

## Slide 2
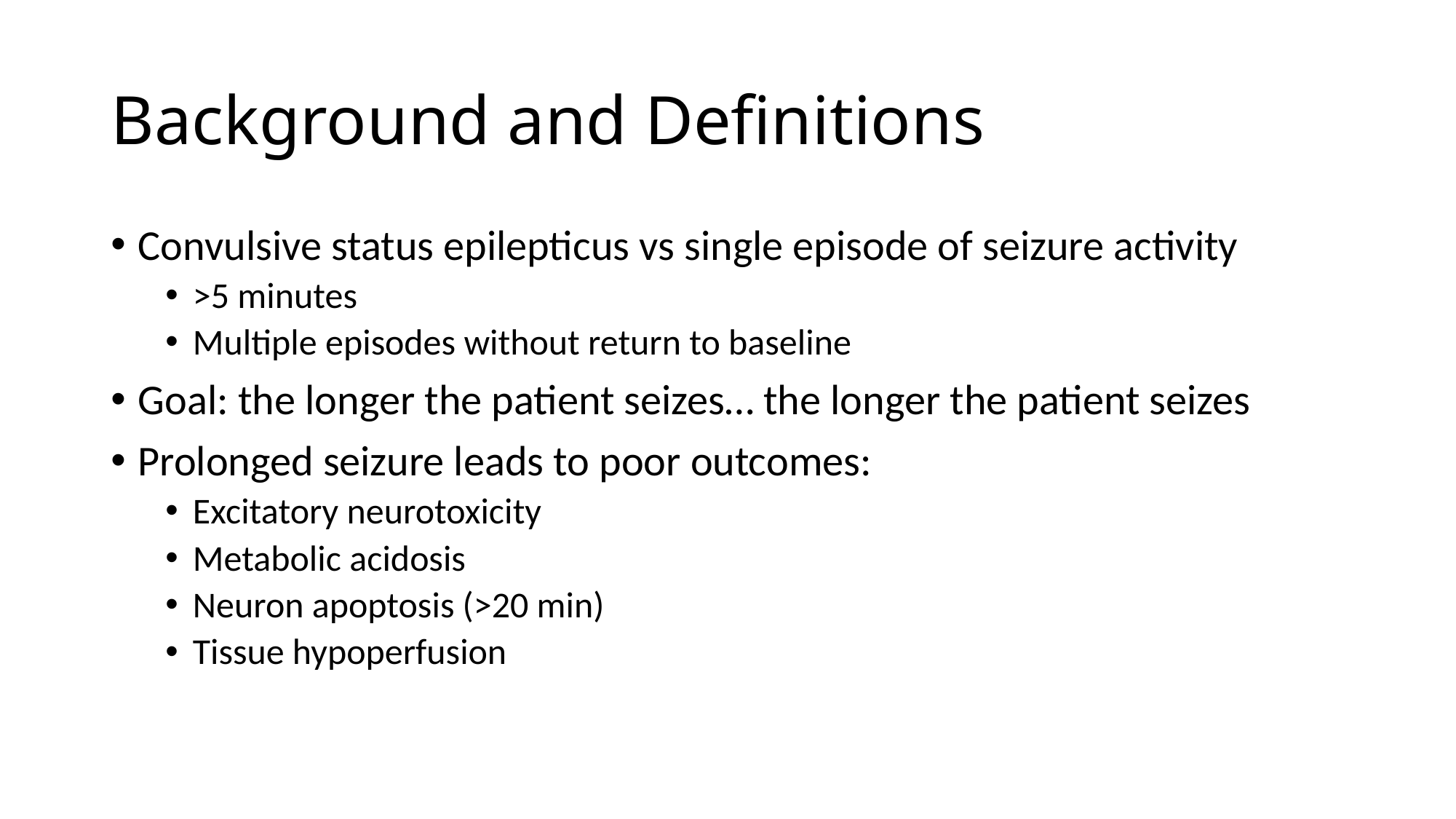

# Background and Definitions
Convulsive status epilepticus vs single episode of seizure activity
>5 minutes
Multiple episodes without return to baseline
Goal: the longer the patient seizes… the longer the patient seizes
Prolonged seizure leads to poor outcomes:
Excitatory neurotoxicity
Metabolic acidosis
Neuron apoptosis (>20 min)
Tissue hypoperfusion

## Slide 3
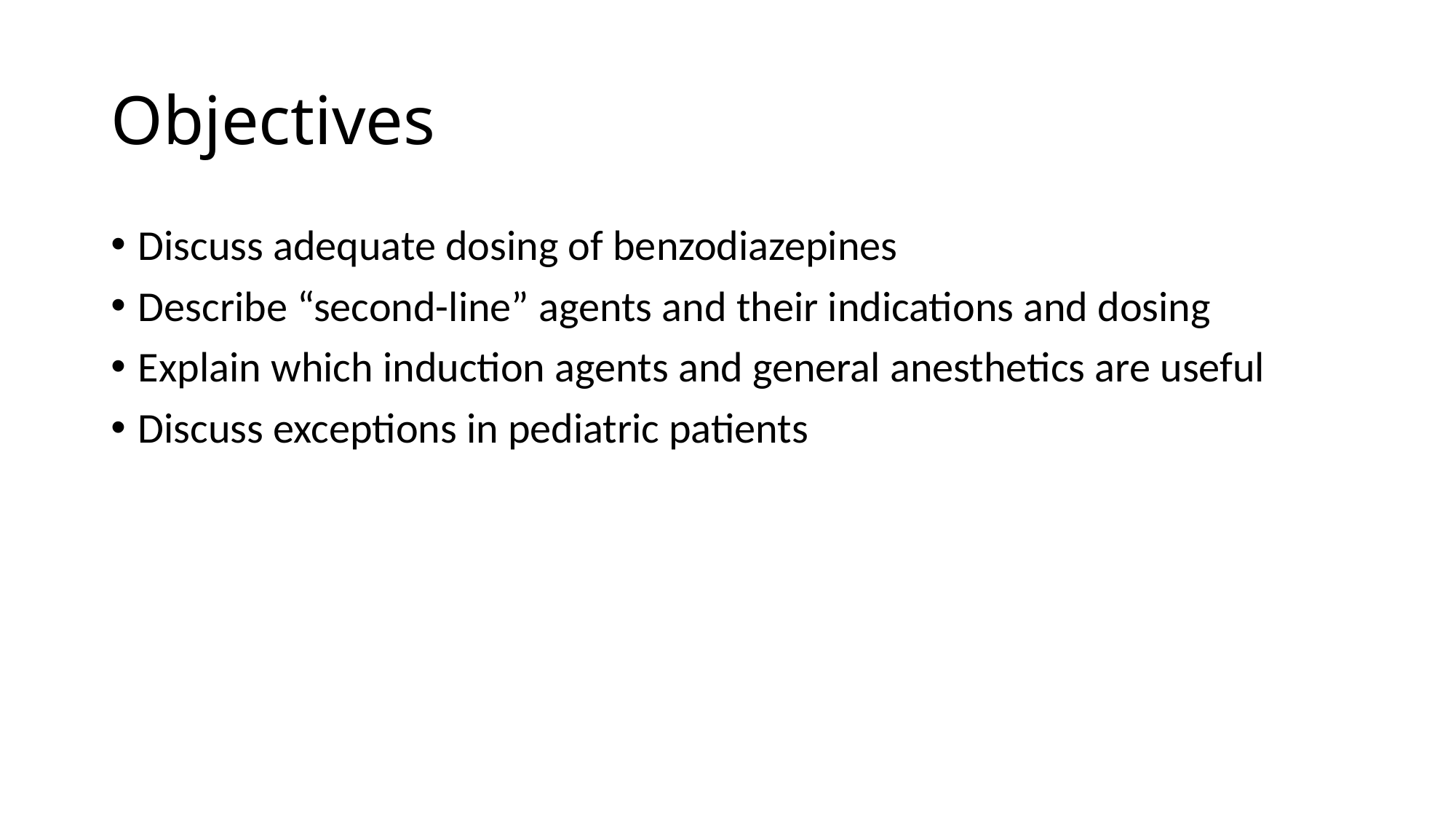

# Objectives
Discuss adequate dosing of benzodiazepines
Describe “second-line” agents and their indications and dosing
Explain which induction agents and general anesthetics are useful
Discuss exceptions in pediatric patients

## Slide 4
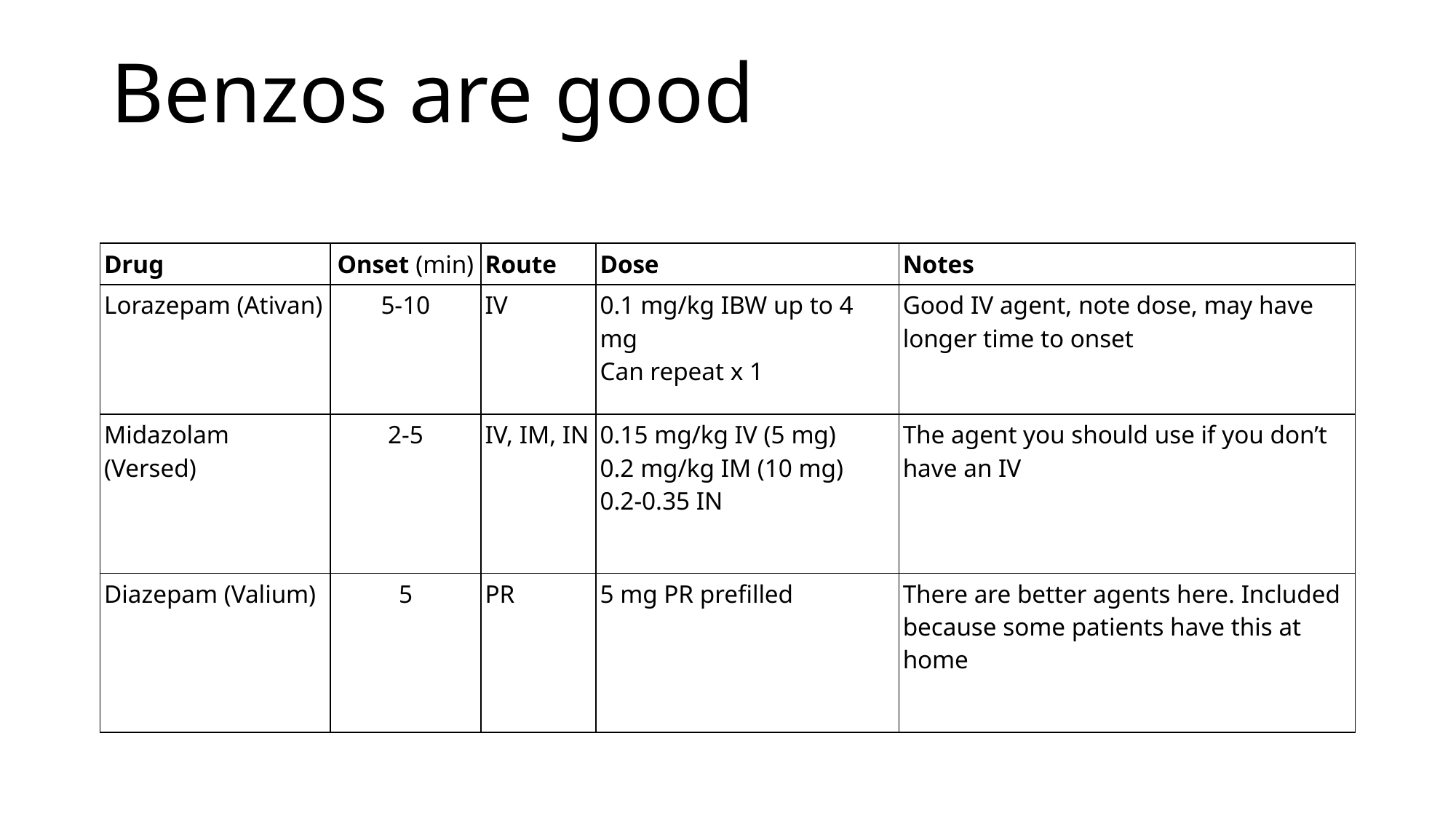

# Benzos are good
| Drug | Onset (min) | Route | Dose | Notes |
| --- | --- | --- | --- | --- |
| Lorazepam (Ativan) | 5-10 | IV | 0.1 mg/kg IBW up to 4 mg Can repeat x 1 | Good IV agent, note dose, may have longer time to onset |
| Midazolam (Versed) | 2-5 | IV, IM, IN | 0.15 mg/kg IV (5 mg) 0.2 mg/kg IM (10 mg) 0.2-0.35 IN | The agent you should use if you don’t have an IV |
| Diazepam (Valium) | 5 | PR | 5 mg PR prefilled | There are better agents here. Included because some patients have this at home |

## Slide 5
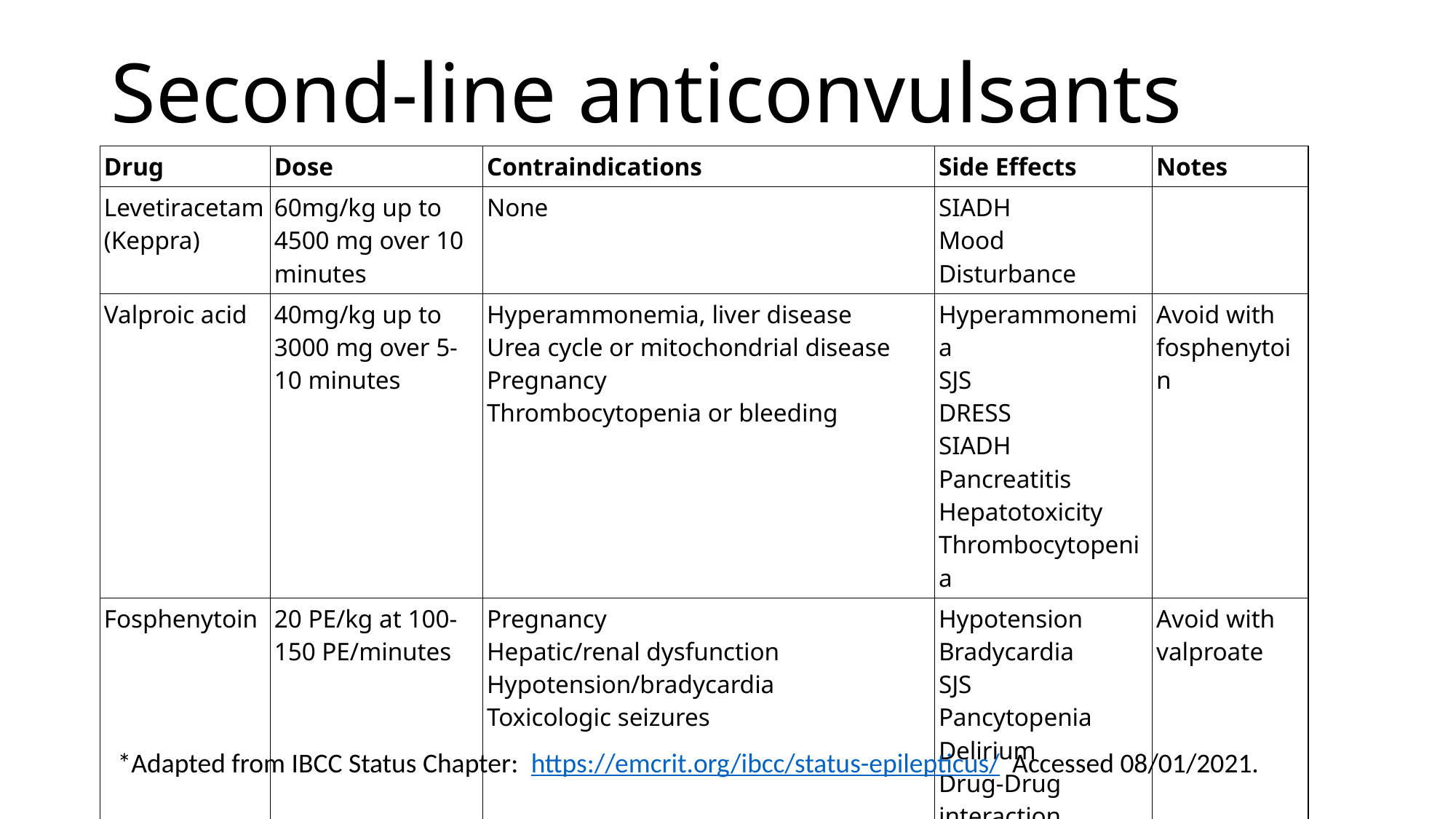

# Second-line anticonvulsants
| Drug | Dose | Contraindications | Side Effects | Notes |
| --- | --- | --- | --- | --- |
| Levetiracetam (Keppra) | 60mg/kg up to 4500 mg over 10 minutes | None | SIADH Mood Disturbance | |
| Valproic acid | 40mg/kg up to 3000 mg over 5-10 minutes | Hyperammonemia, liver disease Urea cycle or mitochondrial disease Pregnancy Thrombocytopenia or bleeding | Hyperammonemia SJS DRESS SIADH Pancreatitis Hepatotoxicity Thrombocytopenia | Avoid with fosphenytoin |
| Fosphenytoin | 20 PE/kg at 100-150 PE/minutes | Pregnancy Hepatic/renal dysfunction Hypotension/bradycardia Toxicologic seizures | Hypotension Bradycardia SJS Pancytopenia Delirium Drug-Drug interaction | Avoid with valproate |
*Adapted from IBCC Status Chapter: https://emcrit.org/ibcc/status-epilepticus/ Accessed 08/01/2021.

## Slide 6
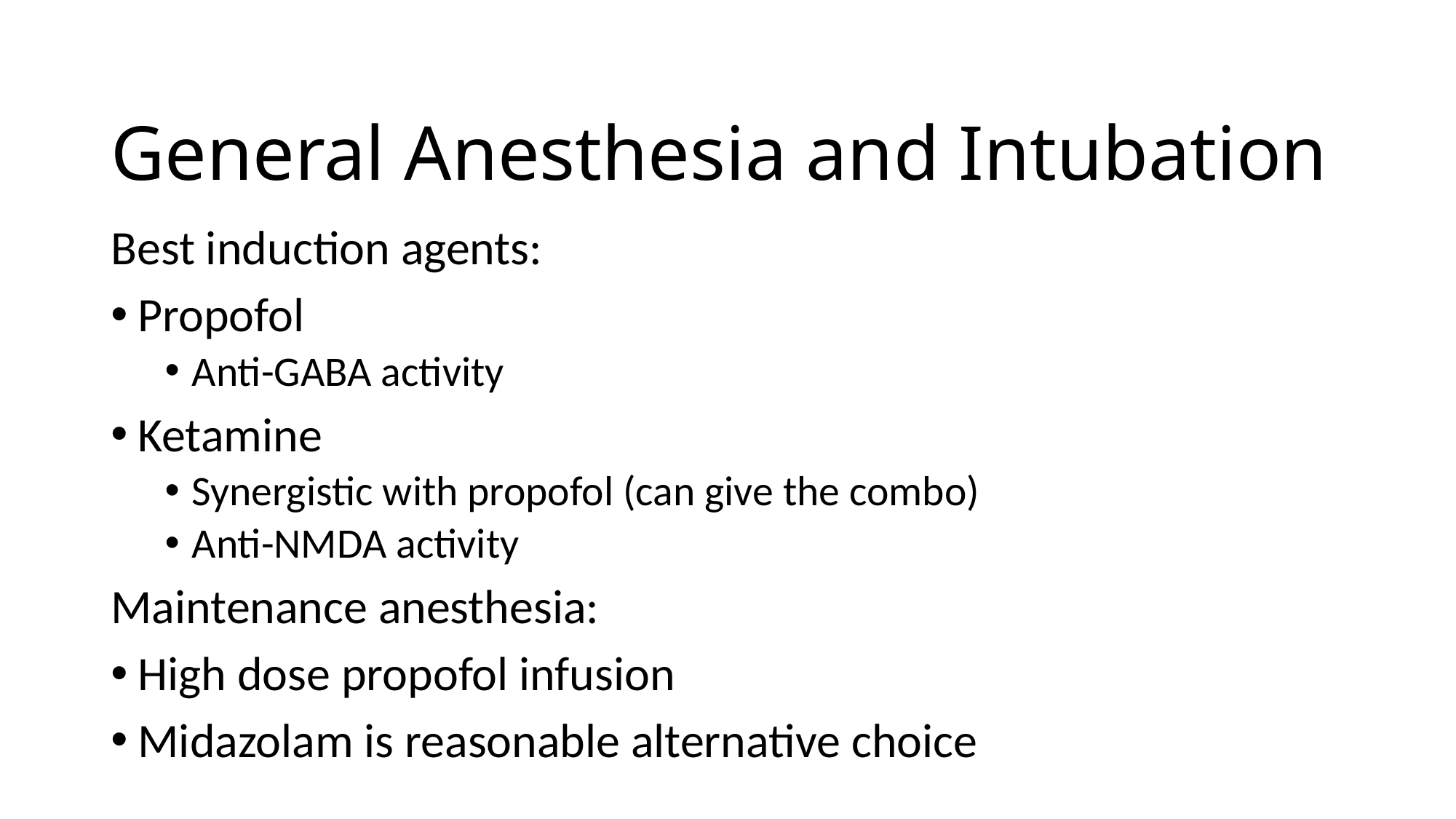

# General Anesthesia and Intubation
Best induction agents:
Propofol
Anti-GABA activity
Ketamine
Synergistic with propofol (can give the combo)
Anti-NMDA activity
Maintenance anesthesia:
High dose propofol infusion
Midazolam is reasonable alternative choice

## Slide 7
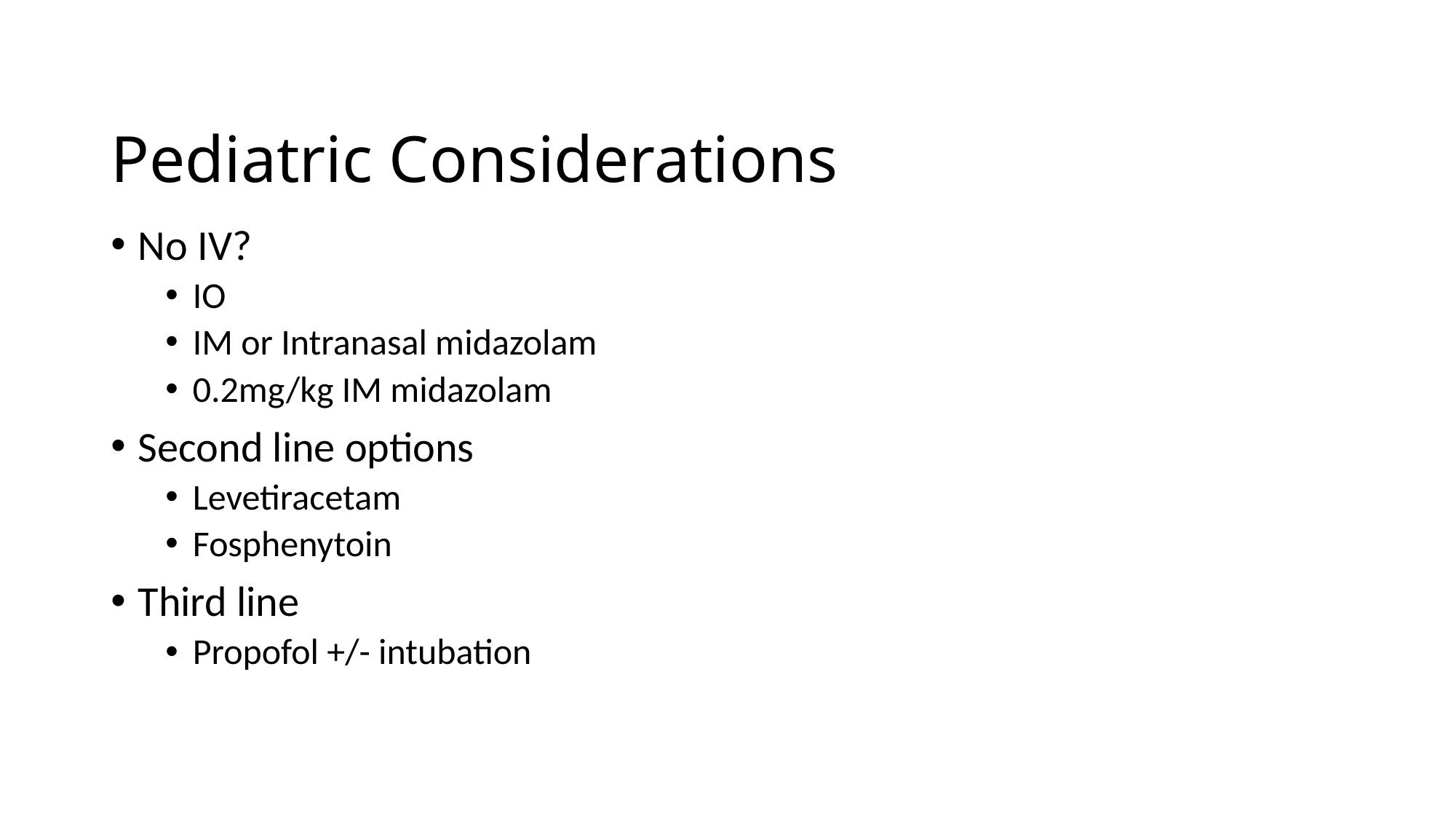

# Pediatric Considerations
No IV?
IO
IM or Intranasal midazolam
0.2mg/kg IM midazolam
Second line options
Levetiracetam
Fosphenytoin
Third line
Propofol +/- intubation

## Slide 8
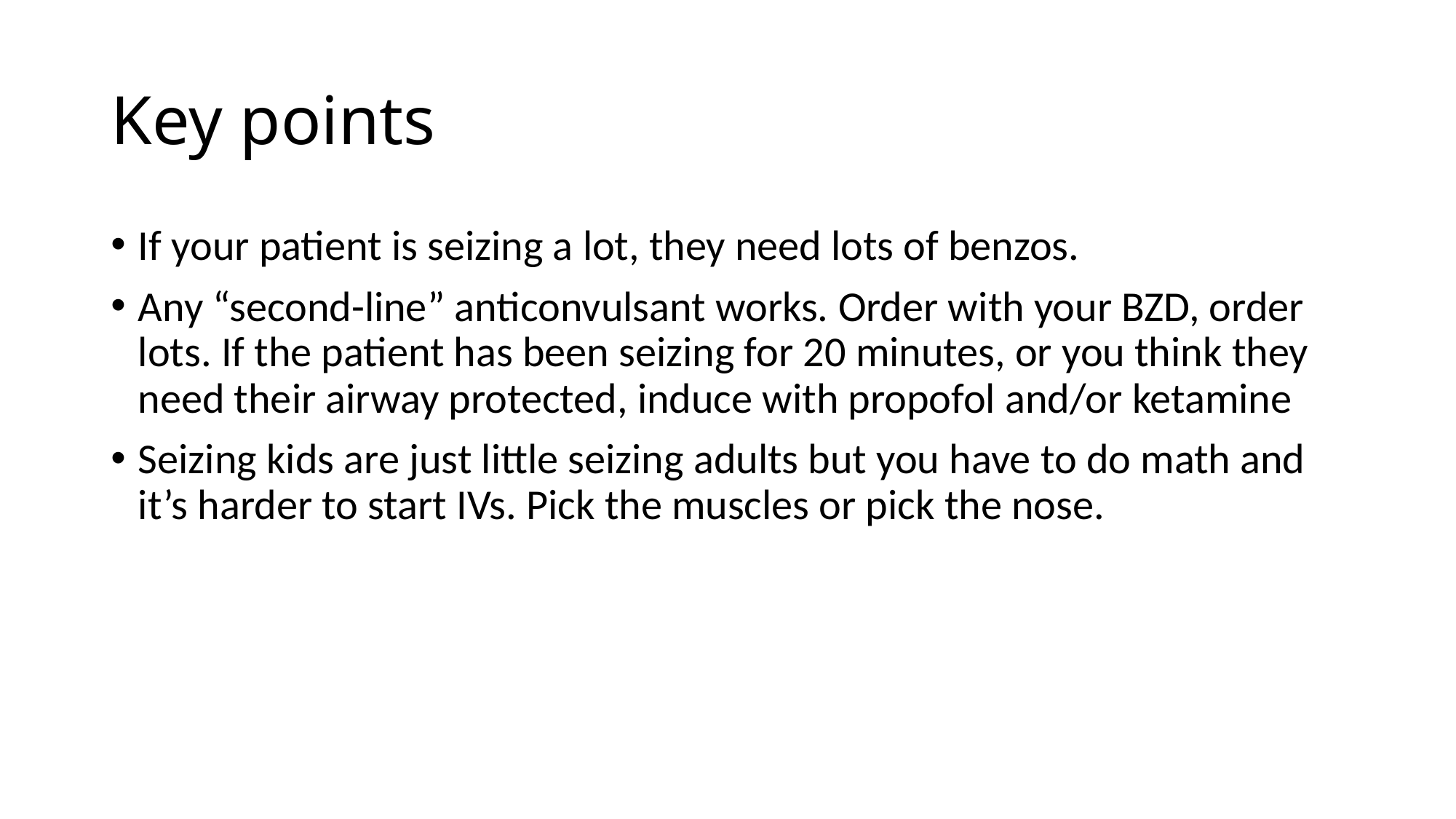

# Key points
If your patient is seizing a lot, they need lots of benzos.
Any “second-line” anticonvulsant works. Order with your BZD, order lots. If the patient has been seizing for 20 minutes, or you think they need their airway protected, induce with propofol and/or ketamine
Seizing kids are just little seizing adults but you have to do math and it’s harder to start IVs. Pick the muscles or pick the nose.

## Slide 9
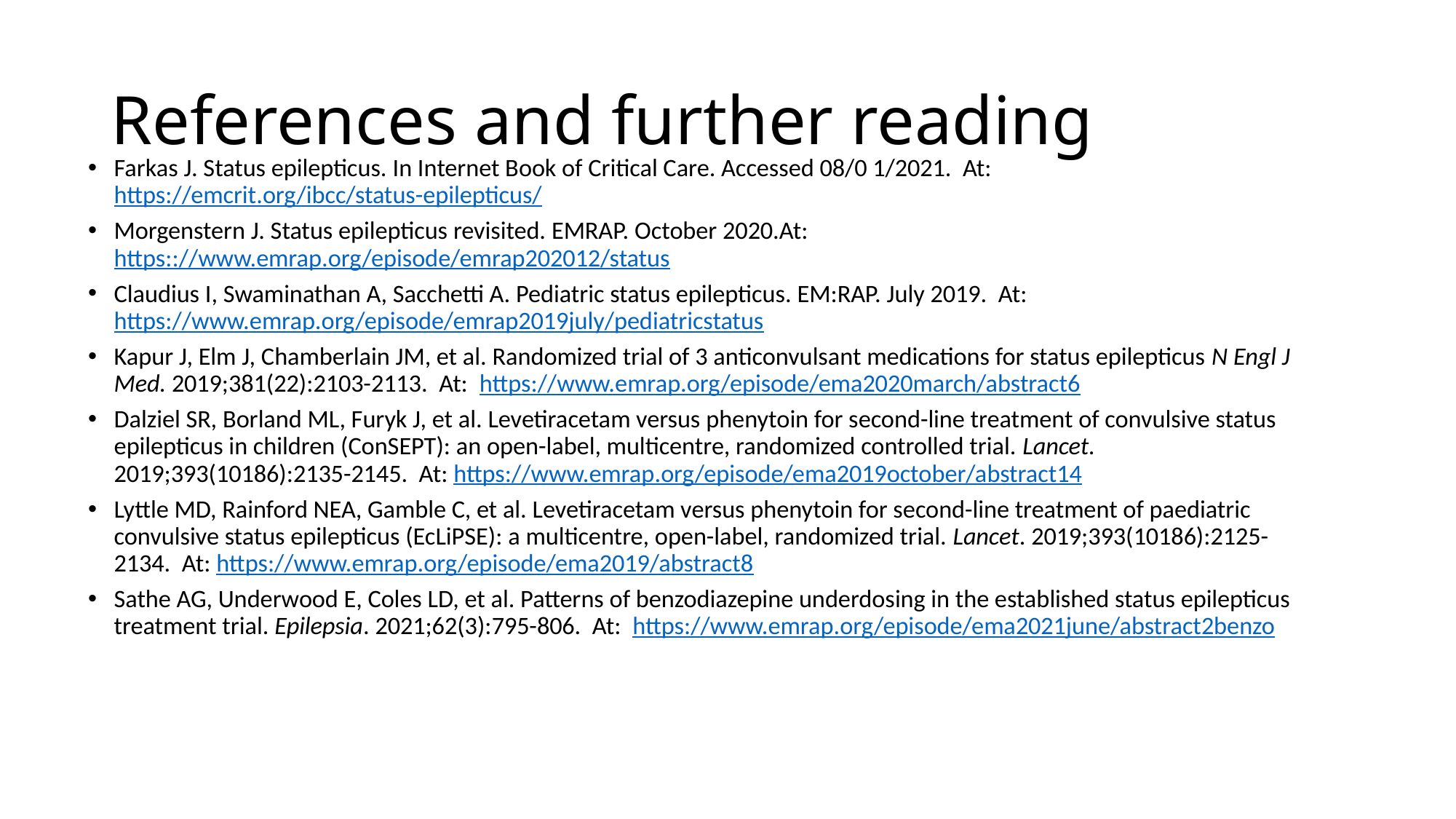

# References and further reading
Farkas J. Status epilepticus. In Internet Book of Critical Care. Accessed 08/0 1/2021. At: https://emcrit.org/ibcc/status-epilepticus/
Morgenstern J. Status epilepticus revisited. EMRAP. October 2020.At: https:://www.emrap.org/episode/emrap202012/status
Claudius I, Swaminathan A, Sacchetti A. Pediatric status epilepticus. EM:RAP. July 2019. At: https://www.emrap.org/episode/emrap2019july/pediatricstatus
Kapur J, Elm J, Chamberlain JM, et al. Randomized trial of 3 anticonvulsant medications for status epilepticus N Engl J Med. 2019;381(22):2103-2113. At: https://www.emrap.org/episode/ema2020march/abstract6
Dalziel SR, Borland ML, Furyk J, et al. Levetiracetam versus phenytoin for second-line treatment of convulsive status epilepticus in children (ConSEPT): an open-label, multicentre, randomized controlled trial. Lancet. 2019;393(10186):2135-2145. At: https://www.emrap.org/episode/ema2019october/abstract14
Lyttle MD, Rainford NEA, Gamble C, et al. Levetiracetam versus phenytoin for second-line treatment of paediatric convulsive status epilepticus (EcLiPSE): a multicentre, open-label, randomized trial. Lancet. 2019;393(10186):2125-2134. At: https://www.emrap.org/episode/ema2019/abstract8
Sathe AG, Underwood E, Coles LD, et al. Patterns of benzodiazepine underdosing in the established status epilepticus treatment trial. Epilepsia. 2021;62(3):795-806. At: https://www.emrap.org/episode/ema2021june/abstract2benzo
